# Investigating the Impact of Packing and Environmental Factors on the Luminescence of Pt(N^N^N) Chromophores

**DOI:** 10.1021/acs.inorgchem.3c04562

**Published:** 2024-01-23

**Authors:** Guillermo Romo-Islas, Sergi Burguera, Antonio Frontera, Laura Rodríguez

**Affiliations:** † Departament de Química Inorgànica i Orgànica, Secció de Química Inorgànica., Institut de Nanociència i Nanotecnologia (IN2UB). Universitat de Barcelona, Martí i Franquès 1-11, Barcelona E-08028, Spain; ‡ Departament de Química, Universitat de les Illes Balears, Palma de Mallorca 07122, Spain

## Abstract

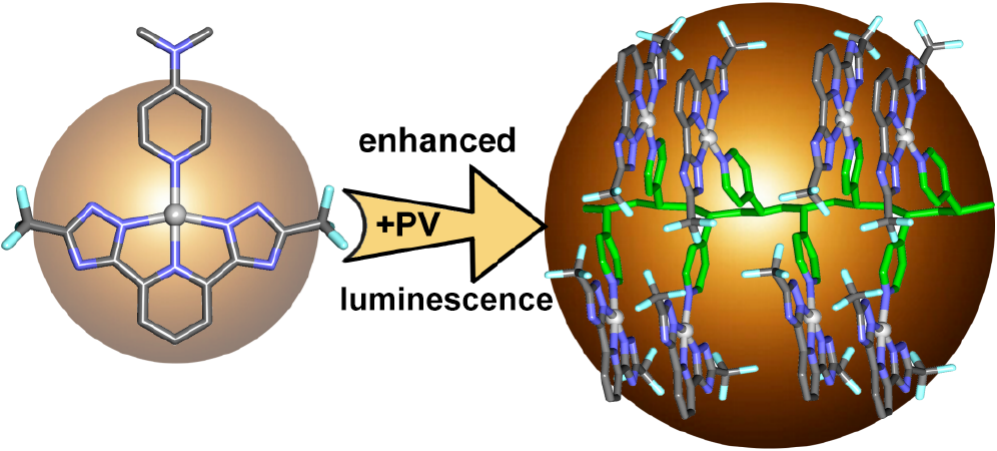

Four Pt(II)(N^N^N) compounds featuring DMSO coordination at the fourth position were synthesized. Ligands varied in terms of pyridyl central ring (hydrogen/chlorine substituent) and lateral rings (triazoles with CF_3_ substitution or tetrazoles). Coordination to pyridine yielded tetra-nitrogen coordinated Pt(II) complexes or Pt-functionalized polymers using commercial 4-pyridyl polyvinyl (PV) or dimethylaminopyridine. Luminescence behaviors exhibited remarkable environmental dependence. While some of the molecular compounds (tetrazole derivatives) in solid state displayed quenched luminescence, all the polymers exhibited ^3^MMLCT emission around 600 nm. Conversely, monomer emission was evident on poly(methyl methacrylate) or polystyrene matrices. DFT calculations were used to analyze the aggregation of the complexes both at the molecular level and coordinated to the PV polymer and their influence on the HOMO–LUMO gaps.

## Abstract 2

The structural modifications on the chemical structure of a series of (N^N^N)Pt(II) moieties have evidenced their key role on the possible establishment of intermolecular weak interactions and how these interactions can affect the resulting luminescence. The compounds have been introduced in organic matrices (favoring monomer emission) and bonded to polymers (favoring MMLCT emission). This behavior has been analyzed experimentally and through DFT calculation studies.

## Introduction

The research on metal-containing polymers and, in particular, metallopolymers, refer to a fascinating category of functional soft materials, in the field of polymer and materials science. These systems are composed of polymeric chains, metal centers, and/or metal-related segments^1−6^ with specific roles suitable to provide a new dimension in several fields such as emissive materials, photovoltaics, electrochromic devices, sensors, nanowires, memory devices, catalysts, or memory and data storage.^7−12^ The presence of metal centers into the metallopolymers is particularly relevant to attain phosphorescent materials for a variety of photonic applications.^13−16^

The design and synthesis of coordination polymers, where the metal centers coordinated to specific ligands in a polymeric chain (being the metal part of the polymeric structure), has been extensively explored.^1−4,11^ Nevertheless, the introduction of metallic chromophores as side pending chains within an organic polymer is less explored and even less, the particular use of metal centers suitable to establish metal···metal interactions, playing particular roles in the 2D and 3D structure of metallopolymers or metal coordinating polymers and also in the resulting emissive properties. Regarding this, poly(4-vinylpyridine), PV, is a very good option due to the presence of pyridyl pending groups, suitable for coordination to a large number of metals and for this reason, it has been used as support of metal-containing polymers, metal nanoparticles, or metal films with several applications such as (photo)sensing or catalysis.^16−18^ Nevertheless, the coordination of specific metals well-known to establish metallophilic contacts is scarcely explored.^17−20^ The use of these metals is of great importance regarding the development of luminescent materials since the resulting photophysical properties are strongly affected by the interplay of different noncovalent interactions such as M···M but also hydrogen bonding, π–π stacking or halogen bonding, among others.^21−25^ Platinum(II), with d^8^ configuration, is one of the most studied metals suitable for metallophilic contacts and with relevant emissive properties. The involvement of several intra- and intermolecular contacts has resulted in the modulation of the intrinsic luminescence that can be tuned on energies (emission wavelength), intensities (quantum yields), or lifetimes (from a few ns to hundreds of microseconds). The establishment of Pt···Pt interactions induces the formation of aggregates and ^3^MMLCT phosphorescence emissions or excimeric transitions.^26−30^ As expected, the establishment of this type of weak interactions is much more favorable if the Pt(II) moieties are located as pendant arms.^31−34^ For this reason, the development and understanding of how supramolecular weak interactions can affect both the packing and the resulting applications as photonic materials is of great relevance and deserves its investigation. In this sense, DFT calculations are convenient to analyze the ability of molecules to aggregate and in the particular case of Pt-complexes, the formation of metallophilic interactions.

Taking all of this into consideration, in this work we have developed new Pt(II) systems containing different tridentate N^N^N ligands as chromophoric units. These Pt(N^N^N) moieties have been coordinated to a pyridyl group included as pendant side in two different polymers (giving rise to the formation of luminescent platinum(II) metallopolymers) and to pyridyl ligands as model compounds. The chemical environment of the Pt(II) systems that differ on specific chemical modifications on the ligands (both on steric and electronic point of view) has been analyzed experimentally and theoretically to understand their role on the resulting packing and photophysical properties.

## Results and Discussion

### Synthesis and Characterization

**Figure 1:**
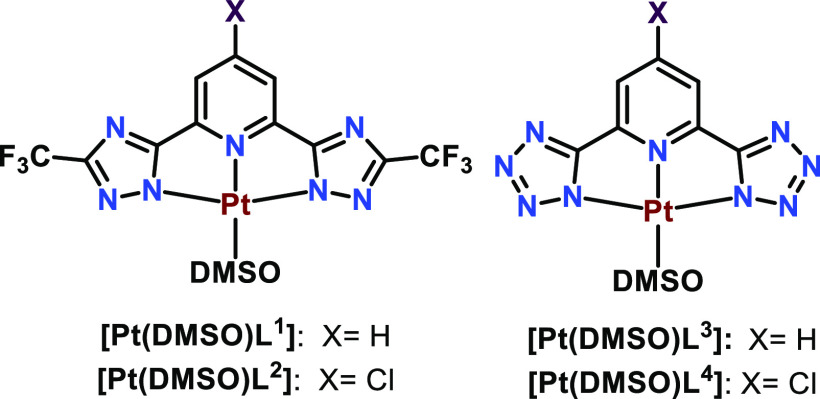
[Pt(DMSO)L] complexes used for the synthesis of the Pt(II)-functionalized polymers.

The development of the Pt(II) functionalized polymers requires the previous synthesis of the Pt(II)(N^N^N) precursors (Figure 1), containing a labile ligand at the fourth coordination position (DMSO). These compounds were obtained based on the previous synthetic procedure where the corresponding N^N^N **H**_**2**_**L** ligands (**H**_**2**_**L**^**1**^–**H**_**2**_**L**^**4**^) reacted with the PtCl_2_(DMSO)_2_ complex.^26^

**Scheme 1:**
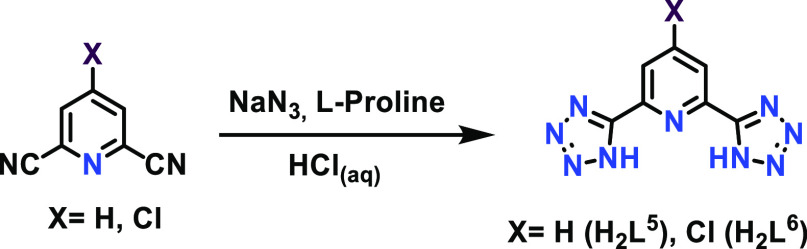
Synthesis of the H_2_L^3^ and H_2_L^4^ Ligands

As it is displayed in Figure 1, four different types of N^N^N **H**_**2**_**L** ligands have been used in this work that differ on the lateral rings being the CF_3_-substituted triazoles, previously reported in the literature,^26,35^ (Figure 1 left) and tetrazoles (Figure 1 right). At the same time, the compounds differ on the *p*-substituted group of the central pyridyl ring that can be a H atom (**H**_**2**_**L**^**1**^ and **H**_**2**_**L**^**3**^) or Cl (**H**_**2**_**L**^**2**^ and **H**_**2**_**L**^**4**^). The tetrazole ligands **H**_**2**_**L**^**3**^ and **H**_**2**_**L**^**4**^ were synthesized by a modification of the procedure described in the literature.^36,37^ That is, the corresponding dicyanopyridine and NaN_3_ (2.5 equiv) reacted in DMF at 110 °C and using l-proline (0.3 equiv) as a catalyst. After 2 h of stirring, the reaction mixture was then allowed to cool down to room temperature and was poured into ice (15 mL) under stirring and then acidified with HCl(c). The mixture was stirred vigorously to obtain the desired product as a white (**H**_**2**_**L**^**3**^) or brownish **H**_**2**_**L**^**4**^) precipitate in the reaction media (Scheme 1). The ^1^H NMR spectra of the ligands show a *ca*. 0.5 ppm downfield shift of the central pyridyl protons with respect to the 2,6-dicyanopyridine precursors. The well-defined band in the IR spectra centered between 3580 and 3415 cm^–1^ corresponding to the (N–H) vibration and the molecular peak recorded in the mass spectra were final evidence of the correct formation of the desired products.

**Scheme 2:**
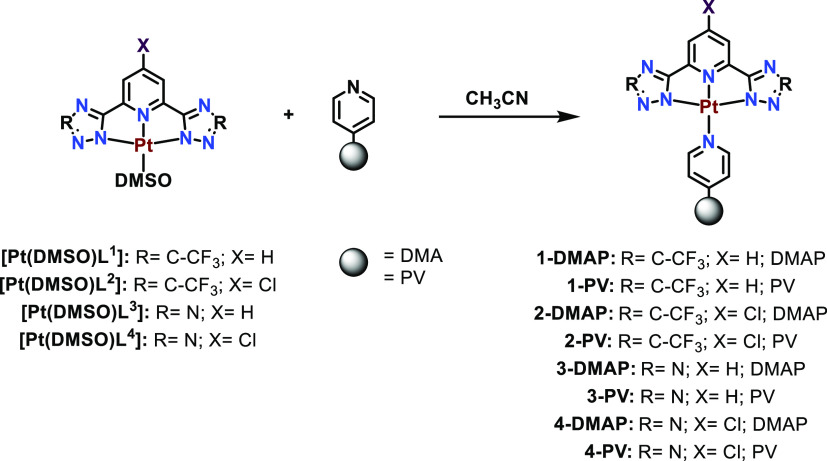
Synthesis of the Pt(N^N^N)-Functionalized Polymers

The four [Pt(DMSO)**L**] compounds were reacted with two different commercially available pyridyl-functionalized polymers 4-(dimethylamine)pyridine, dimethylaminopyridine (DMAP)-containing polymer (an average of 3.0 mmol pyridyl units per gram), and poly(4-vinylpyridine), PV (average MW ∼ 60 000) where the labile DMSO ligand leaves the Pt(II) environment in the presence of the pyridyl group of the polymer by the establishment of the new Pt-py bonds (Scheme 2). There are clear differences between the two of them: while the PV polymer is completely functionalized by pending pyridyl groups, the DMAP polymer is a ∼ 3.0 mmol/g DMAP loading, matrix cross-linked with 2% DVB. That is, the number of coordination sites on the DMAP polymer is much lower than that in the PV. This should affect the resulting luminescent properties of the resulting functionalized polymers.

The reactions were carried out in acetonitrile under reflux for 18 h. An excess of the platinum complex was required to complete the reaction in the DMAP derivatives, with lower degree of pyridyl pending functionalization. The polymer was insoluble in this medium but a color change in the solid suspended in the reaction mixture was observed in all cases after this time, being direct evidence of the formation of a new insoluble product. The purification of the resulting solids was carried out by filtration and washing with DMSO/EtOH, in order to remove the possible traces of noncoordinated Pt(II) precursors, giving the desired Pt(II)-functionalized polymers in moderate-high yields (*ca*. 65–87%).

**Scheme 3:**
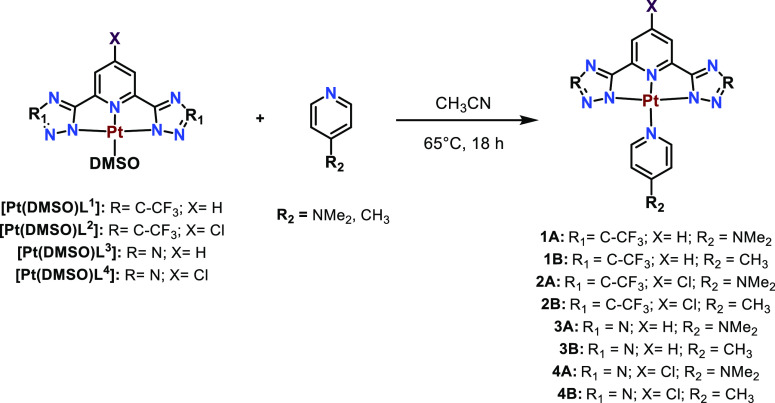
Synthesis of the Pt(N^N^N)-py Compounds

To better understand both the packing and the properties of the Pt(N^N^N) functionalized-polymers, we decided to synthesize the analogous Pt(N^N^N)-pyridyl compounds containing 4-dimethylamino pyridine and 4-methylaminopydine in the fourth coordination position, previously occupied by the pyridyl unit of the polymers. For this goal, the [Pt(DMSO)**L**] compounds were reacted with the corresponding pyridyl ligand in a 1:1 stoichiometry in acetonitrile under reflux for 18 h (Scheme 3).^38,39^ The purification by recrystallization with dichloromethane/hexane gave the desired compounds moderate-high yields (*ca*. 42–84%).

The correct formation of the desired systems has been clearly evidenced by ^1^H NMR and mass spectrometry (for the molecular compounds **1–4A,B**). IR spectroscopy and powder X-ray diffraction were carried out for all the systems since the insolubility of the polymers precludes their characterization by the previous techniques.

The ^1^H NMR spectra of the final products exhibit the presence of the corresponding protons of the pyridyl groups that are 0.12–0.89 ppm upfield shifted with respect to the free pyridine precursor. The signals corresponding to the −CF_3_ moieties are also detected in the ^19^F NMR spectra of corresponding compounds and their IR spectra with a well-defined band between 1183 and 1061 cm^–1^ corresponding to the ν(C–F). The final evidence of the correct formation of the compounds has been detected in the high-resolution mass spectra with the identification of the [M + H^+^] or [M + Na^+^] molecular peaks. The IR spectra of the Pt(II) compounds coordinated to the polymers all exhibit a similar behavior (see Supporting Information). All the functionalized polymers display shifts about 53–31 cm^–1^ of the −N=N bond from the N^N^N ligands compared to these vibrations in the [Pt(DMSO)**L**] precursors, together with the presence of the −CF_3_ vibration bands (for the triazole functionalized polymers) that are not detected in the free DMAP or 4-pyridy polyvinyl polymers. The −C=N vibration of the pyridine is observed in all cases better defined (less broad) in the functionalized-polymers compared to the polymer precursors, due to the coordination to the Pt(II) moieties.^40−42^ X-ray diffraction analyses of the powders were carried out for both the functionalized polymers and their precursors, the uncoordinated DMAP and PVP polymers, and the molecular compounds **1–4A,B**.

**Figure 2:**
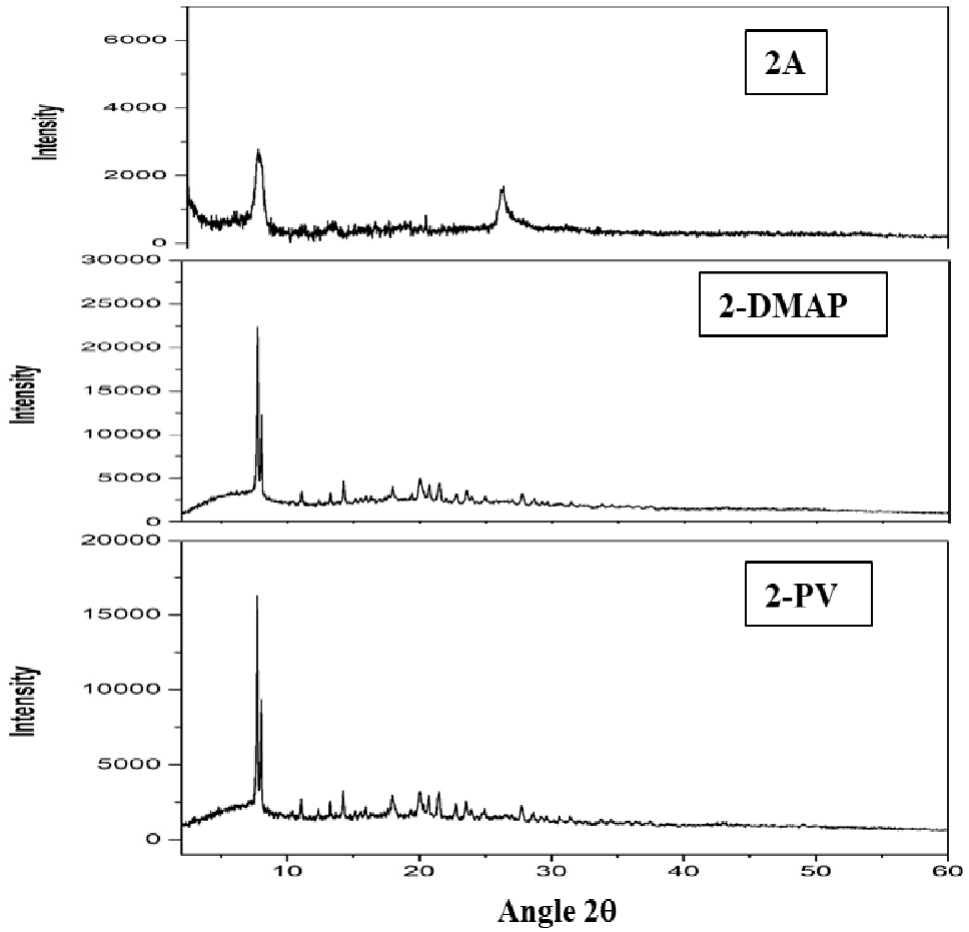
DRX spectra of **2A**, **2-DMAP**, and **2-PV** showing the better crystallinity of the functionalized polymers.

The diffraction spectra of the initial polymers, DMAP and PVP, exhibit a broad band ranging from 15 to 25°, which can be attributed to the low crystallinity of the material, consistent with prior research findings.^18,43,44^ Improved organization, indicating higher crystallinity, is evident in the functionalized polymers, particularly when compared to the powders of the isolated molecules 1–4A,B (see Figure 2). The highest level of organization is observed in systems derived from N^N^N ligands L2 and L3, primarily influenced by the presence of Pt(II) metal moieties, aligning with established literature.^45^

### Photophysical Characterization

All compounds and Pt(N^N^N)-functionalized polymers were characterized from photophysical point of view. None of the compounds were significantly luminescent in solution, and thus, all the data presented herein are based on the solid state and it is summarized in Table 1. The commercial DMAP and PV polymers were also not luminescent.

**Table 1:** Photophysical Data of Pt(N^N^N)-Functionalized Polymers and Analogous Pt(N^N^N) Complexes

complex	λ_max_ Em (nm)	ϕ	τ (μs)	*k*_r_ (×10^5^ s^–1^)	*k*_nr_ (×10^5^ s^–1^)	*k*_nr_/*k*_r_
**1-DMAP**	585	0.02	τ_1_ = 0.1 (42.19) τ_2_ = 0.5 (57.81)	0.6	31.4	52
**1-PV**	594	0.07	τ_1_ = 0.1 (67.39) τ_2_ = 0.6 (32.61)	2.4	34.9	15
**2-DMAP**	595	0.01	τ_1_ = 0.2 (45.01) τ_2_ = 0.9 (54.99)	0.2	17.2	86
**2-PV**	583	0.01	τ_1_= 0.1 (31.57) τ_2_ = 1.4 (68.43)	0.1	9.8	98
**3-DMAP**	558	0.03	τ_1_ = 0.1 (49.28) τ_2_ = 0.6 (50.72)	0.9	27.4	30
**3-PV**	568	0.03	τ_1_ = 0.1 (37.56) τ_2_ = 0.6 (64.44)	0.8	23.3	29
**4-DMAP**	572	0.01	τ_1_ = 0.1 (36.30) τ_2_ = 0.9 (63.70)	0.1	16.1	161
**4-PV**	583	0.01	τ_1_ = 0.1 (36.33) τ_2_ = 0.9 (63.67)	0.2	16.0	80
**1A**	494, 585	0.01	τ_1_ = 0.1 (21.87) τ_2_ = 0.7 (78.13)	0.2	14.3	72
**1B**	573	0.02	τ_1_ = 0.1 (45.52) τ_2_ = 0.4 (54.48)	0.5	35.2	70
**2A**	587	0.02	τ_1_ = 0.1 (35.99) τ_2_ = 0.4 (64.01)	0.5	25.8	52
**2B**	596	0.01	τ_1_ = 0.1 (42.79) τ_2_ = 0.2 (57.21)	0.9	65.8	73

The emission spectra of all the systems were recorded at room temperature upon excitation the samples at 450 nm. A broad emission band between 500 and 600 nm is recorded in all cases that is attributed to ^3^MMLCT transition based on previous reports with similar Pt(N^N^N) compounds.^26,31,46−50^ This means that some kind of aggregation/intermolecular packing should be expected in all cases. An ^3^IL contribution is also observed only in the case of **1A** with the presence of a vibronic resolution band at higher energies^51^ overlapped with the previous one, due to the possibility of several metal environments or different numbers of M···M interactions.^20^

**Figure 3:**
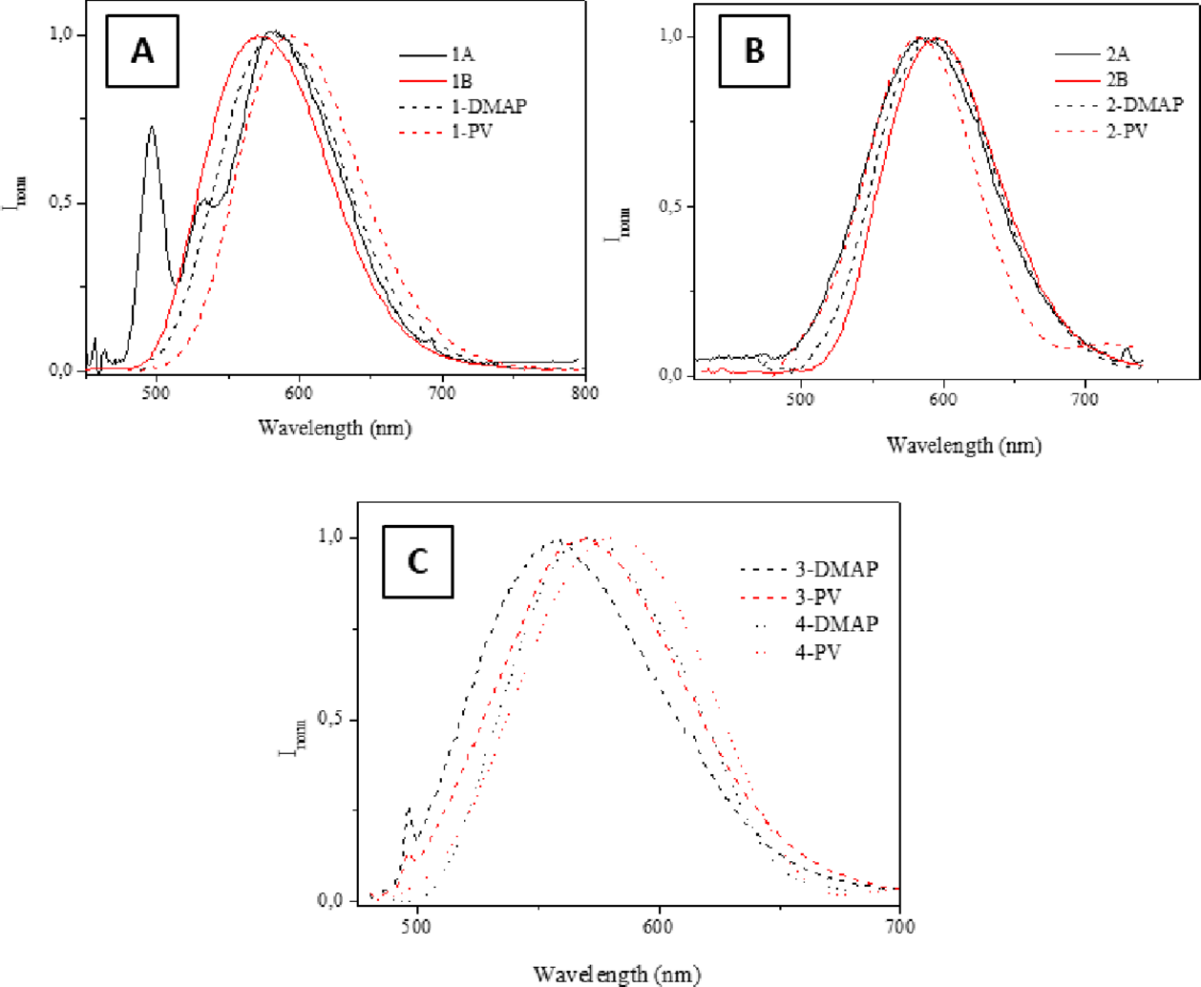
Emission spectra of the Pt(N^N^N) complexes and respective functionalized polymers in the solid state, being A) for **L**^**1**^-derivatives; B) **L**^**2**^-derivatives; C) **L**^**3**^- and **L**^**4**^-derivatives. λ_exc_ = 450 nm.

Additionally, some differences can be observed in the photophysical properties of the triazoles and tetrazoles derivatives. First of all, tetrazole derivatives (compounds **3** and **4A,B**) are not emissive in their molecular form but their analogous functionalized polymers are. This can be due to the different HOMO–LUMO transition, as predicted by TD-DFT calculations for **3A** and **4A** (see below) and to a probable more efficient packing in the solid state that can cause aggregation caused quenching (ACQ). The emission of the Pt-functionalized systems containing the triazole **L**^**1**^ and **L**^**2**^ ligands is *ca*. 20 nm red-shifted compared to those containing the tetrazole **L**^**3**^ and **L**^**4**^ ligands. On the other hand, a *ca*. 10 nm emission red-shift is observed for compounds containing X=Cl compared to their analogous with X=H (Figure 3). Additionally, the Pt-functionalized systems derived from the PV polymer also display an emission red-shift with respect to the DMAP functionalized polymers. This can be due to the very well distribution and closer proximity of the Pt(N^N^N) moieties in the former polymer that would make a more favored aggregates’ emission and corresponding red-shift (see below DFT studies).

Finally, we can observe a more efficient emission (higher quantum yield) for the compounds with X=H compared to their corresponding containing X=Cl (being **1-PV** the most efficient one) that present more favored nonradiative deactivation pathways in the polymers (larger *k*_nr/_*k*_r_ values, Table 1).

**Figure 4:**
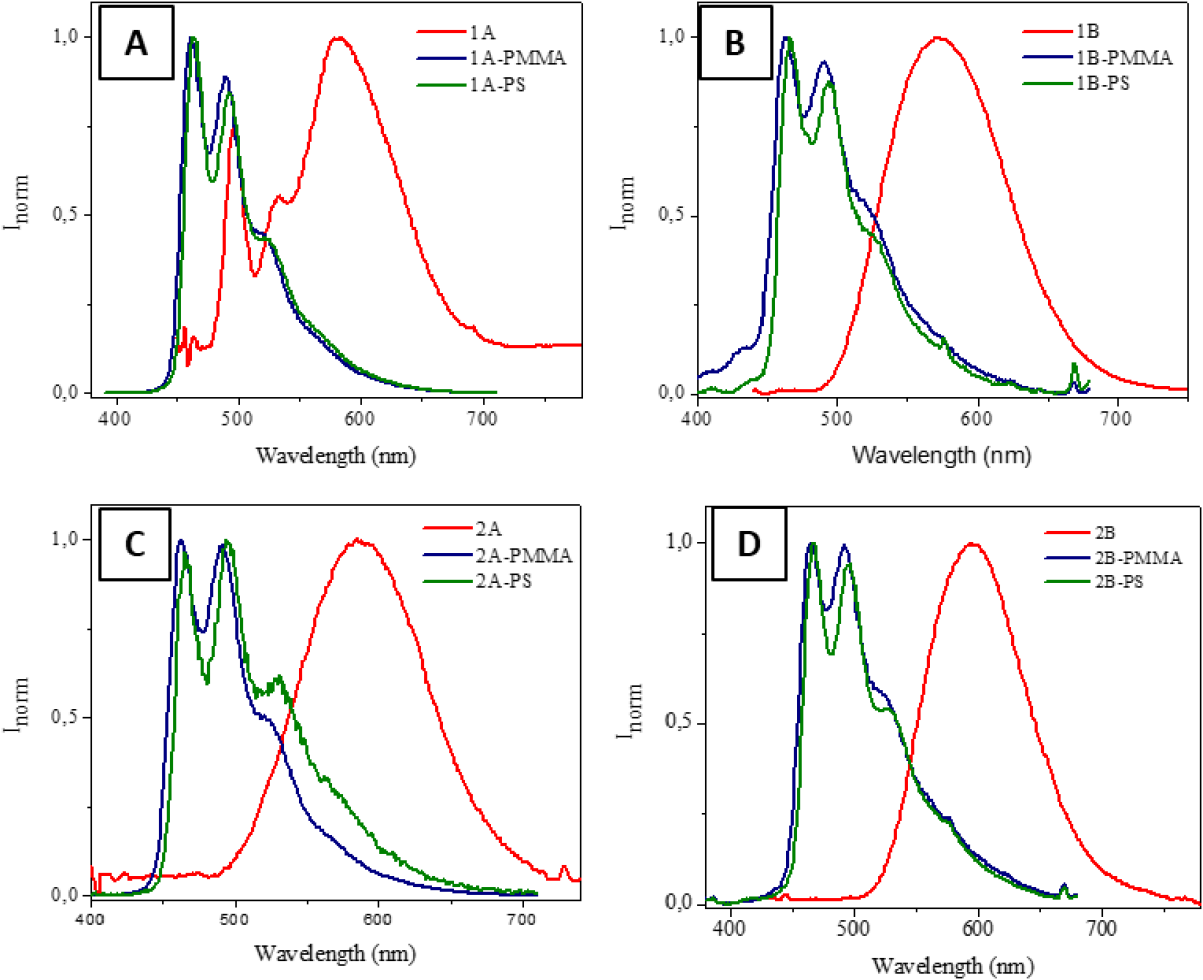
Emission spectra of the emissive Pt(N^N^N) complexes within PMMA and PS matrices and comparison with the corresponding spectra of the compound in the solid state, being A) for **1A**; B) for **1B**; C) for **1C**; C) for **1D**. λ_exc_ = 380 nm.

The recorded photophysical data let us suspect that the packing is a key point on the resulting emissive properties of our Pt(N^N^N) complexes. According to this, largest packing (solid state) produces ACQ effect while a better alignment of the molecules (as further studied below), thanks to their coordination to a polymer, improves their resulting emission. An intermediate situation would be doping organic matrices, such as poly(methyl methacrylate) (PMMA) or polystyrene (PS). Based on our previous experience, the dispersion of luminophores within this type of matrices may improve their emission properties regarding emission quantum yields and lifetimes.^23,52−54^ The recorded emission spectra indicate that the intermolecular packing, responsible for the resulting MMLCT emission is observed to be not favored in these media while the ^3^IL monomer is the emissive species instead (Figure 4 and Table 2). Vibronically structured bands are herein displayed due to a well distribution of the molecules within the thin film media, and they present, as expected, higher emission quantum yields and larger emission lifetimes.^27^ The recorded emission lifetimes show biexponential decays probably ascribed to the different environments of the molecules within the films.

**Table 2:** Photophysical Data of Compounds **1-2A,B** Dispersed in PMMA and PS

compound	λ_max_ Em (nm) PMMA	ϕ PMMA	τ (μs) PMMA	λ_max_ Em (nm) PS	ϕ PS	τ (μs) PS
**1A**	461, 488	0.20	τ_1_ = 1.0 (50.66) τ_2_ = 4.4 (49.34)	464, 493	0.33	τ_1_ = 0.9 (65.03) τ_2_ = 3.6 (34.97)
**1B**	462, 491	0.04	τ_1_ = 0.1 (45.52) τ_2_ = 0.4 (54.48)	466, 494	0.12	τ_1_ = 0.1 (45.52) τ_2_ = 0.4 (54.48)
**2A**	463, 490	0.08	τ_1_ = 0.9 (41.42) τ_2_ = 3.8 (58.58)	465, 494	0.11	τ_1_ = 0.1 (47.93) τ_2_ = 0.6 (52.07)
**2B**	464, 493	0.06	τ_1_ = 1.0 (49.84) τ_2_ = 5.2 (50.16)	467, 495	0.12	τ_1_ = 0.9 (55.23) τ_2_ = 4.3 (44.77)

### DFT Analysis of the Aggregation

The planarity and large aromatic surfaces present in the complexes may favor the formation of aggregates in solution and solid state. We have studied this theoretically by comparing the relative ability of these complexes to form self-assembled dimers and how the formation of these dimers affects the HOMO–LUMO levels and consequently their luminescent properties. In addition, the presence of metallophilic Pt···Pt interactions in the dimers has been also analyzed and correlated with the experimental findings.

**Figure 5:**
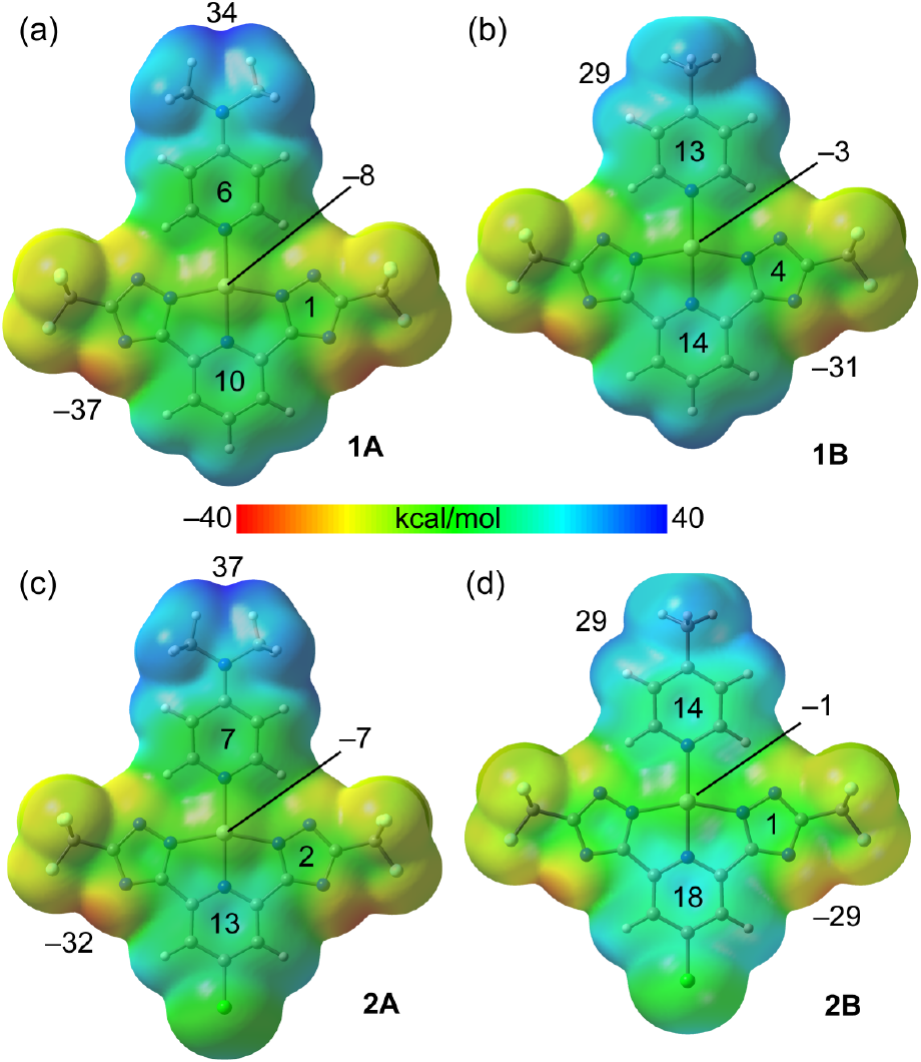
MEP surfaces of compounds **1A** (a), **1B** (b), **2A** (c), and **2B** (d) at the PBE0-D4/def2-TZVP level of theory. The values at the minimum, maximum, and selected points are given in kcal/mol. Density isovalue 0.001 au.

**Figure 6:**
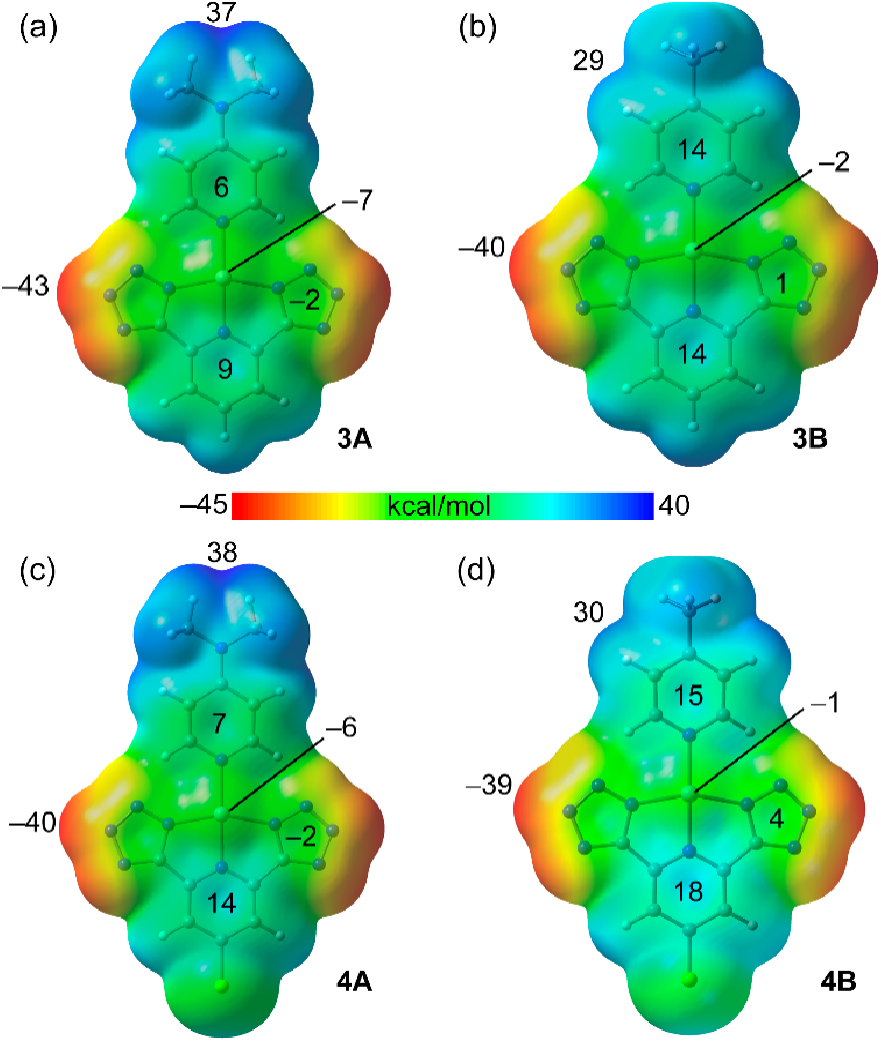
MEP surfaces of compounds **3A** (a), **3B** (b), **4A** (c), and **4B** (d) at the PBE0-D4/def2-TZVP level of theory. The values at the minimum, maximum, and selected points are given in kcal/mol. Density isovalue 0.001 au.

As starting point, the molecular electrostatic potential (MEP) surfaces of all compounds have been computed to study the most electron rich and poor regions of the molecules and the π-acidity/basicity of the π-clouds. The MEP surfaces of compounds **1A,B** and **2A,B** are given in Figure 5 and those of **3A,B** and **4A,B** in Figure 6. It can be observed that the MEP minima and maxima are located at the molecular plane, being the maximum values at the NMe_2_ group in the A-series and between the methyl group and the adjacent aromatic H atom in the B-series. The values range between 28 and 38 kcal/mol. The MEP minima are located at the N atoms of the triazole (Figure 5) or tetrazole (Figure 6) derivatives, being the MEP values more negative for the latter (−39 to −43 kcal/mol).

In all compounds, the MEP values at the aromatic rings are relatively small, thus favoring the π-stacking interactions (small electrostatic repulsion). Moreover, the MEP values at the Pt-atoms are very small, also favoring the dispersion-dominated metallophilic interactions.

**Figure 7:**
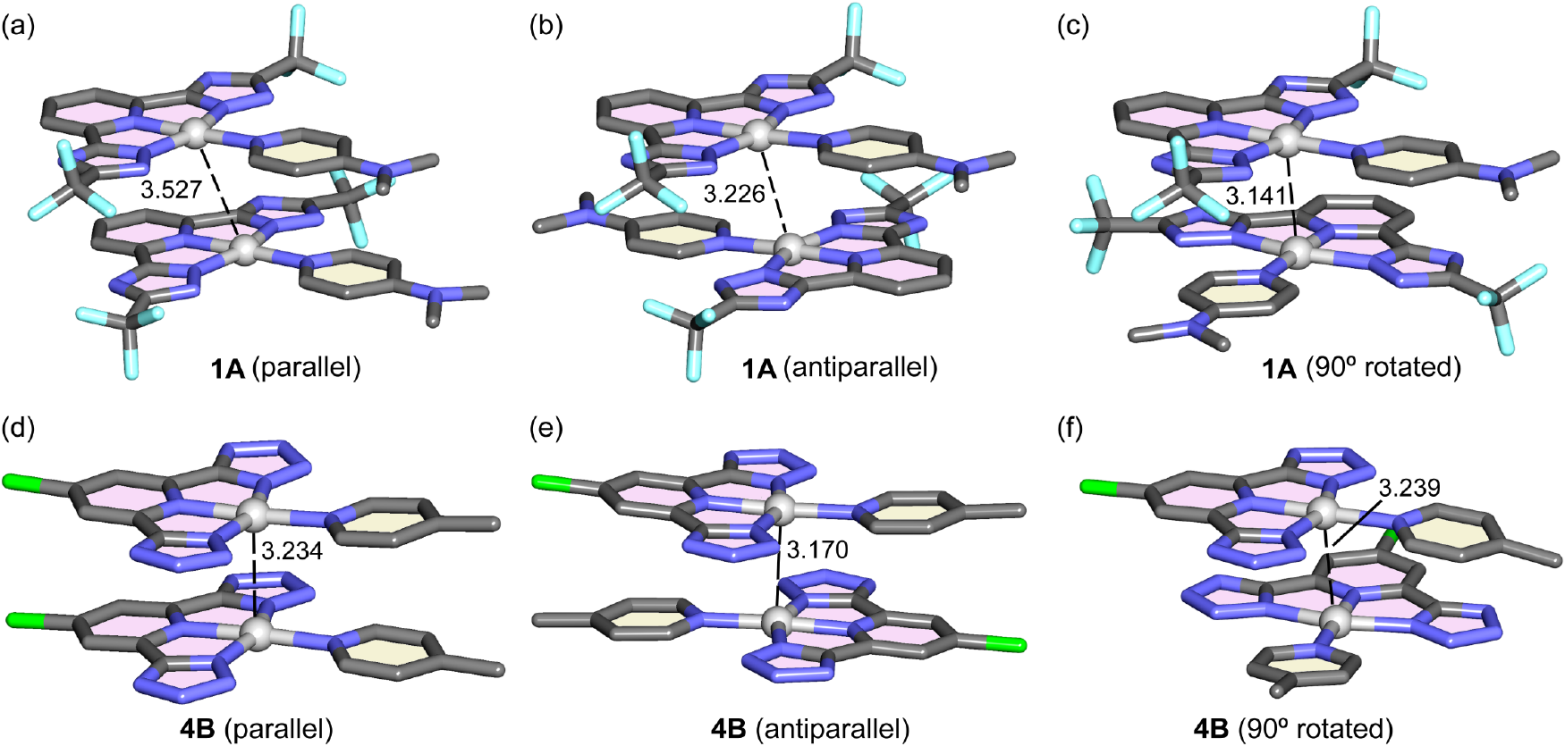
Perspective views of the PBE0-D4/def2-TZVP optimized geometries of the dimers **1A** parallel (a), antiparallel (b), and 90° rotated (c) and **4B** parallel (d), antiparallel (e), and 90° rotated (f). The Pt···Pt interactions are given in Å. Pt are represented as gray spheres and the rest of the molecule in “tube” format.

For all compounds reported herein, the π-stacked dimers have been computed by using three different arrangements, which are parallel, antiparallel, and 90° rotated. The geometries for two exemplifying dimers are given in Figure 7 (**1A** and **4B** and the rest in Figures S79 and S80). In general, the dimers of the **A** series present longer metallophilic distances in line with the more negative MEP values at the Pt(II)-atom. The triazole dimers of **2A** and **2B** (Cl-substituted pyridine in tridentate ligand) present shorter (or equivalent in some cases) distances compared to **1A** and **1B** dimers (unsubstituted pyridine in tridentate ligand) that aligns with the more efficient emission of the latter. A similar result is also observed for the tetrazole dimers, where the Cl-substituted dimers present shorter distances than the nonsubstituted ones. The metallophillic distance variation within the dimers is more significant in the triazole series, where the 90° rotated is shorter, that can be related to the steric hindrance caused by the −CF_3_ groups that is minimized in the 90° rotated dimers.

The energetic features of the dimers of all compounds are gathered in Table S1, in the gas phase and acetonitrile and water solutions and briefly commented herein. In the gas phase, the 90° rotated dimers are more favored that the other two, however the differences become much smaller in the presence of solvent, thus suggesting that the aggregation of the complexes in solution leads to a mixture of all possible conformations. Although the energy differences are small in solution, some trends can be extracted from the inspection of the results. First, the aggregation is more favored in water than in acetonitrile in all cases. Second, the triazole derivatives have more tendency to aggregate than the tetrazole ones, in spite of having the CF_3_ group likely due to additional van der Waals (C–F···F–C) or CH···F contacts. The Cl substituent does not have a noticeable influence on the interaction energies. The energies have also been computed using unrestricted calculations (UKS), providing similar tendencies but stronger binding energies see Table S2.

**Figure 8:**
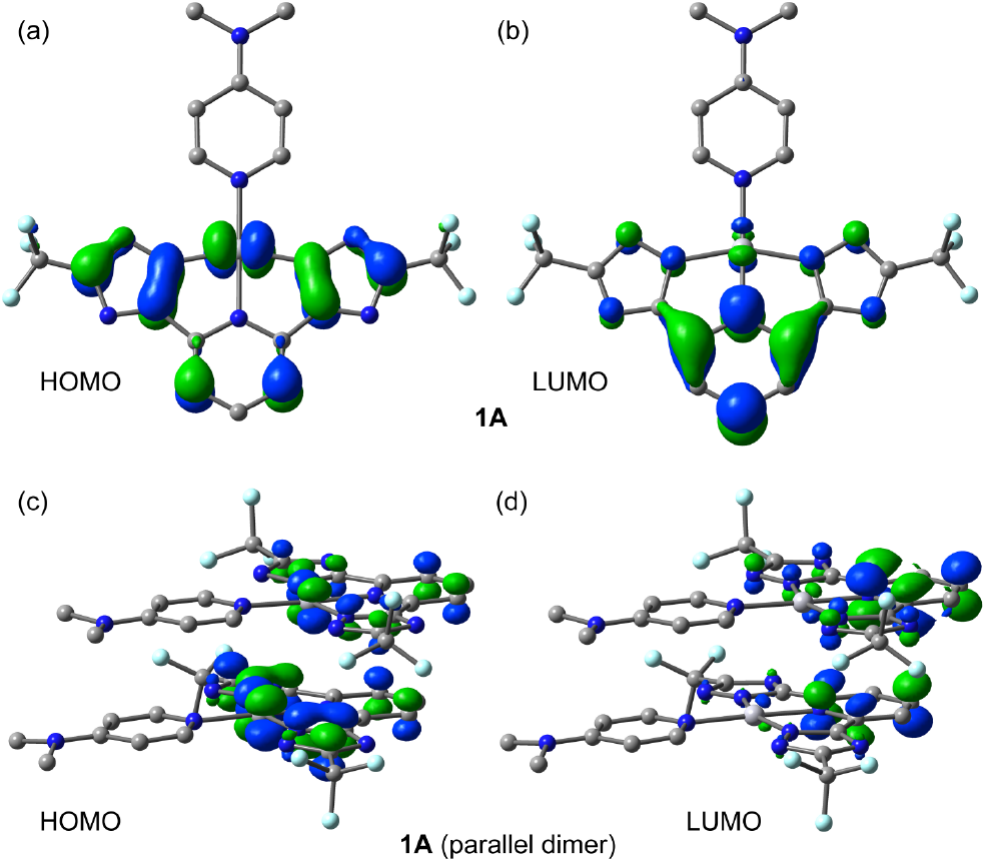
HOMO and LUMO plots of **1A** (a,b) and the parallel dimer of **1B** (c,d).

The influence of aggregation on the HOMO–LUMO gaps has also been investigated. Table S3 gathers the gaps for the monomers and the dimers in all three orientations. In all cases, the aggregation caused a reduction of the HOMO–LUMO gap, ranging from 0.1 to 0.6 eV, depending on the orientation and solvation. It is important to note that TD-DFT calculations demonstrate (see theoretical methods for further details) that the S_0_ → S_1_ excitation corresponds to a HOMO → LUMO transition (∼97%) in the monomers. A similar behavior is observed in the dimers, where the HOMO → LUMO is also dominant (∼85%). The HOMO–LUMO plots of **1A** are given in Figure 8 as a representative example and the rest in the Supporting Information (Figure S81). In **1A**, the π-system of the tridentate ligand and the metal center are important contributors to the HOMO, while in the LUMOs, the contribution of the metal is negligible. Therefore, this transition can be considered as a mixture of MLCT and intraligand CT. This explains the influence of the dimer formation on the HOMO and LUMO energies (see Table S3). The HOMO and LUMO plots are similar in the parallel dimer of **1A** (Figure 8c,d) compared to the monomer. It is interesting to highlight that for compounds **3A** and **4A**, the HOMO is located in the pyridine ligand instead of the tridentate ligand (Figure S81), as observed in **1A** and **2A**. This can be related to the lack of luminescence recorded for the tetrazole compounds.

**Figure 9:**
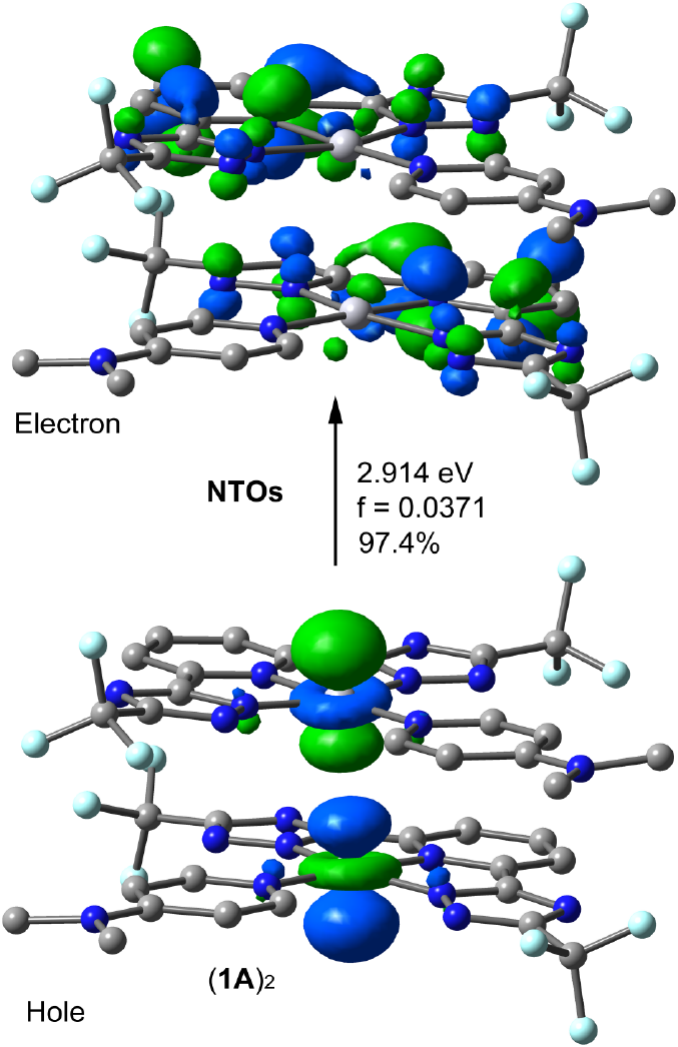
Natural transition orbitals (NTOs) for the dimer of compound 1A illustrating the nature of the singlet excited state. The transition energy (eV) and the oscillator strength are also indicated. Shown are only occupied (hole) and unoccupied (electron) NTO pair that contributes more than 97% to the S_1_ excited state.

Furthermore, Figure 9 presents the natural transition orbitals (NTOs) for the dimer of **1A**, accounting for 96% of the transition. NTOs provide a more simplified and chemically meaningful representation of orbital transitions in an excited state compared to canonical MOs. The spatial distribution depicted in the NTOs strongly suggests a metal-to-ligand charge transfer. Specifically, the occupied NTO (hole) is primarily associated with the d_*z*_2 atomic orbitals of Pt(II), while the unoccupied orbital embraces the π-system of the aromatic ring. This pronounced charge transfer could potentially enhance intersystem crossing to triplet states, consistent with the observed red-shift and the ^3^MMLCT emission in our experiments. It is noteworthy to mention that emission assignments for Pt(II) compounds are well-understood, having been rigorously investigated both experimentally and theoretically, as evidenced by several studies in the literature.^51,55−57^

**Figure 10:**
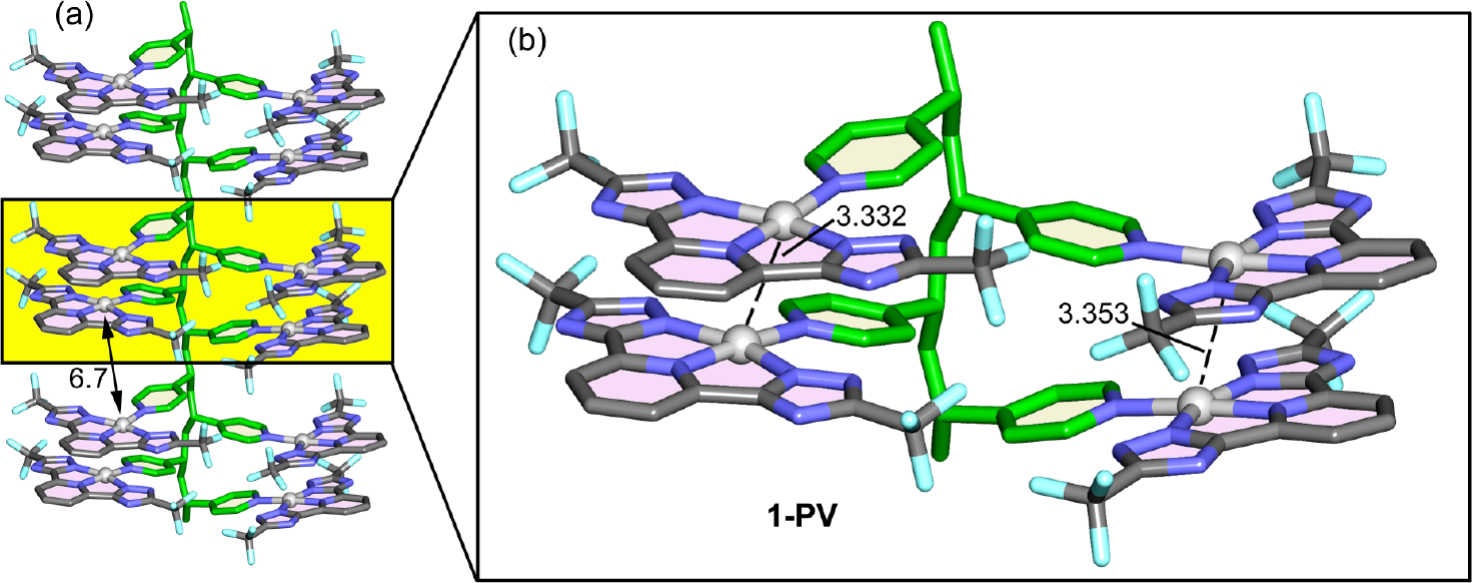
(a) Representation of the oligomer **1-PV**. (b) Detail of the tetramer showing the formation of parallel π-stacked dimers. Distances in Å.

Two functionalized PV polymers (**1-PV** and **3-PV**) have been modeled in order to rationalize the influence of the substrate immobilization on the luminescence properties. For the optimization, a tetrameric part was fully optimized at the RI-BP86-D4/def2-TZVP level of theory and then this unit was used to generate the oligomer (12 units) shown in Figure 10 for 1-PV and Figure S82 for 3-PV. For the calculations, we have considered that all the pyridine residues of the polymer are coordinated to Pt(N^N^N), although the real system most likely has vacant positions. The examination of the geometry suggests that the well-aligned parallel dimers are formed in the polymer, in line with the improved emission of the polymers. It seems that the formation of larger π–π aggregates within the polymer is difficult, since the Pt···Pt distance between two consecutive dimers is around 6.7 Å. Therefore, the effect of the coordination to the PV seems to be generation of a large number of well-defined parallel dimers. Furthermore, the initial coordination of a pyridyl group in the polymer with Pt(N^N^N) most likely assists, through π-stacking interactions, the functionalization of adjacent pyridyl moieties (specifically those in the 1, 5 relative position relative to the aliphatic chain). This mechanism could enhance the formation of dimeric assemblies within the polymer, even under the less-than-optimal functionalization observed in our experiments. Interestingly, the data in Table S3 show that the parallel dimers cause the largest reduction of HOMO–LUMO gaps in most cases. The results for the 3-PV polymer are very similar in terms of Pt···Pt distances and the formation of well-defined parallel π-stacked dimers.

## Conclusions

The structural modifications on the chemical structure of a series of (N^N^N)Pt(II) complexes have evidenced their key role on the possible establishment of intermolecular weak interactions and how these interactions can affect the resulting luminescence. Thus, we can modulate the emission wavelengths, efficiencies (quantum yields), and lifetimes of the compounds depending on the environment and the corresponding intermolecular contacts between them in a well-aligned distribution (^3^MMLCT emission in the functionalized polymers), with the organic matrix (^3^IL emission from the monomer in PMMA or PS).

These results open a wide range of new possibilities on the research based on supramolecular chemistry and luminescent materials, taking into consideration that many other parameters can be modified giving rise to an easy tune of the luminescence of this type of metallopolymers and materials.

The DFT study reveals the facility of these compounds to form aggregates via π-stacking and metallophilic interactions. Several possible orientations have been analyzed indicating that parallel and antipararallel orientations have similar binding energies and influence on HOMO–LUMO gaps. Models of 1-PV and 3-PV polymers have been computed confirming the formation of well-defined parallel dimers that likely favor the ^3^MMLCT emission.

## Experimental Section

### General Information

Solvents have been purchased from commercial sources and dried by distillation under a nitrogen atmosphere. Reagents 2,6-pyridinedicarbonitrile, 4-chloropyridine-2,6-carbonitrile, *N,N*-diisopropylethylamine (DIPEA), l-proline, poly(4-vinylpyridine) cross-linked (poly(4-VP), CAS: 9017-40-7), 4-(dimethylamino)pyridine polymer-bound (DMAP) with ∼3.0 mmol/g “DMAP” loading, matrix cross-linked with 2% DVB (CAS: 82942-26-5), hydrazine hydrate, and trifluoroacetic acid have been purchased from commercial sources and used without further purification. The precursor [PtCl_2_(DMSO)_2_] was prepared following the procedure described in the literature.^58^

### Physical Measurements

^1^H NMR (δ(TMS) = 0.0 ppm), ^19^F NMR (δ(CFCl_3_) = 0.0 ppm), and ^31^P{^1^H} NMR (δ(85% H_3_PO_4_) = 0.0 ppm) spectra have been obtained on Bruker 400 and Bruker 500 (Universitat de Barcelona) instruments. Electrospray mass spectra (+) have been recorded on a Fisons VG Quatro spectrometer (Universitat de Barcelona). Infrared spectra have been recorded on an FT-IR 520 Nicolet spectrophotometer. The emission spectra of the compounds in solution have been obtained in a fluorescence quartz cuvette of 5 mm or 10 mm path length, using a Horiba-Jobin-Yvon SPEX Nanolog spectrofluorimeter (Universitat de Barcelona). Emission quantum yields were determined with a Hamamatsu Quantaurus QY absolute photoluminescence quantum yield spectrometer C11347 with an absorbance in the range of 0.043–0.139. Luminescence lifetimes were measured on a JYF-DELTAPRO-NL equipment upon excitation of the samples with a 390 nm NanoLED and collecting the decays through a cutoff filter at the corresponding emission maxima. X-ray powder diffraction has been recorded on the PANalytical X’Pert PRO MPD θ/θ powder diffractometer of 240 mm of radius, in a configuration of convergent beam with a focalizing mirror and a transmission geometry with flat samples sandwiched between low absorbing films. With a Cu Kα radiation (λ = 1.5418 Å). Work power: 45 kV – 40 mA. Incident beam slits defining a beam height of 0.4 mm. Incident and diffracted beam 0.02 radians Soller slits. PIXcel detector: Active length = 3.347°. 2θ/θ scan from 2 to 60° 2θ with a step size of 0.0263° 2θ and a measuring time of 300 s per step (Universitat de Barcelona).

### Preparation of Matrices Doped with the Pt(II) Complexes

PMMA and PS were used as the matrix polymers. The films were made using a drop-casting technique with a combination of dopant and host (PMMA or PS). The polymer solutions were prepared as follows: PMMA, MW 120000 in 30% solution in dichloromethane and PS, MW 45000, in 35% solution in dichloromethane. The same volume of a sample solution with a concentration of 20 mg/mL was added to a polymer solution. To prevent any thermal annealing, the films were drop-cast onto a quartz substrate at ambient temperature.

### Theoretical Calculations

The geometries of the complexes and dimeric assemblies were fully optimized without symmetry constrains at the RI-PBE0-D4(COSMO)/def2-TZVP//RI-BP86-D4/def2-TZVP level of theory^59−62^ by means of the Turbomole 7.7 program.^63^ Moreover, this combination of functional and basis set has been recently used by us to explain the aggregation of Pt-^26^ and Au-complexes.^49^ The basis set includes relativistic effects for Pt in the definitionof the ECPs.^62^ The binding energies were computed by subtracting the sum of the energies of the monomers to the energy of the dimer. Solvent effects were taken into consideration using the COSMO-RS method^64^ as implemented in the Turbomole program.^63^ The TD-DFT calculations were computed using 30 excited states at the RI-PBE0-D4/def2-TZVP level of theory^65^ using ORCA 5.0 program.^66^

### Synthesis and Characterization

#### Synthesis of H_2_L^3^

0.5 g of 2,6-pyridinedicarbonitrile (3.87 mmol) was dissolved in 5 mL of DMF in a sealed Schlenk tube. 0.629 g of sodium azide (9.67 mmol) and 0.105 g of l-proline have been then added, and the solution was stirred for 3 h at 110 °C. After this, the solution was cooled to room temperature. The mixture reaction was poured into an ice–water solution (20 mL) with stirring. The mixture was acidulated with HCl 0.5 M under stirring. The mixture was filtered and washed with water. After filtration, a white solid (0.75 g, 90% yield) was obtained. ^1^H NMR (400 MHz, DMSO-*d*_*6*_) δ = 8.34 (s, 3H, *Hpy*) ppm. IR (cm^–1^): ν = 3415 (N–H), 3003 (C-Har), 2923 (C–H), 1557 (C=N), 1453 (C=C), 1093 (C–N).

#### Synthesis of H_2_L^4^

A similar procedure used for **H**_**2**_**L**^**3**^ was followed in the synthesis of **H**_**2**_**L**^**4**^ but using 4-chloropyridine-2,6-carbonitrile instead of 2,6-pyridinedicarbonitrile. After filtration, a brown solid was obtained (0.601 g, 79% yield). ^1^H NMR (400 MHz, DMSO-*d*_*6*_) δ = 7.98 (s, 2H, *HPy*) ppm. IR (cm^–1^): ν = 3415 (N–H), 3003 (C-Har), 2923 (C–H), 1557 (C=N), 1453 (C=C), 1093 (C–N).

#### Synthesis of [Pt(DMSO)L^3^]

0.20 g of **H**_**2**_**L**_**3**_ (0.92 mmol) have been dissolved in 1 mL of DMF in a Schenk flask equipped with reflux and 406 μL of DIPEA (2.32 mmol) have been added to the solution and stirred for 15 min. Then, 0.339 g of [Pt(DMSO)_2_Cl_2_] (0.51 mmol) was added to the mixture and heated at 75 °C for 24 h. The reaction was cooled to room temperature, and an excess of diethyl ether was used to precipitate the product. After filtration, a red solid was obtained (0.388 g, 86% yield). ^1^H NMR (400 MHz, DMSO-*d*_*6*_) δ = 8.22 (t, *J* = 8.6 Hz, 1H, *H4-py*), 8.12 (d, *J* = 7.6 Hz, 2H, *H3,5-py*), 2.70 (s, 6H, *CH*_*3*_) ppm. IR (cm^–1^): ν = 3081 (C-Har), 2945 (C–H), 1619 (C=N), 1457 (C=C), 1118 (S=O), 823 (Pt–N).

#### Synthesis of [Pt(DMSO)L^4^]

A similar procedure used for [Pt(DMSO)L^3^] was followed in the synthesis of [Pt(DMSO)L^4^] but using **H**_**2**_**L**^**4**^ instead of **H**_**2**_**L**^**3**^. A brown solid was obtained after filtration (0.3378 g, 70% yield) 1H NMR (400 MHz, DMSO- *d*_*6*_) δ = 8.51 (s, 3H, *Hpy*), 2.69 (s, 6H, CH_3_) ppm. IR (cm^–1^): ν = 3019 (C-Har), 2993 (C–H), 1667 (C=N), 1496 (C=C), 1385 (CH_3_), 1023 (S=O), 916 (C–N), 746 (CCl).

#### Synthesis of [Pt(L^1^)(4-DMAP)], 1-DMAP

Twenty mg (0.032 mmol) of [Pt(DMSO)L^1^] and 5.3 mg (0.016 mmol of pyridyl groups) of 4-(dimethylamine)pyridine polymer bounded have been suspended in 5 mL of dry acetonitrile under a nitrogen atmosphere and reflux. The reaction was stirred overnight at 65 °C. All the volatiles have been then evaporated under vacuum, and the crude product was washed with DMSO and EtOH. After filtration, a pale-yellow solid was obtained (12.2 mg, 79% yield). IR (cm^–1^): ν = 3461 (N–H), 3083 (C-Har), 2923 (C–H), 2853 (N–CH_3_), 1650 (C=C), 1490(−C=N), 1384 (−CH_3_), 1160 (−CF_3_), 520 (Pt–N). Anal. Calcd (For (C_213_H_178_F_30_N_45_Pt_5_) unit): C: 52.07%, H: 3.65%, N: 12.83%, Pt: 19.85%; Found: C: 52.21%, H: 3.57%, N: 12.40%, Pt: 19.74%.

#### Synthesis of [Pt(L^1^)(4-Vynylpyridine)], 1-PV

A similar procedure used for **1-DMAP** was followed in the synthesis of **1-PV** but using 3.5 mg (0.032 mmol) of poly(4-vinylpyridine) instead of 4-(dimethylamine)pyridine polymer-bounded. A yellow solid was obtained after filtration (16.7 mg, 80% yield). IR (cm^–1^): ν = 3421 (N–H), 3092 (C–H_ar_), 2923 (C–H), 2851 (−CH_2_), 1650 (C=C), 1481 (−C=N), 1152 (−CF_3_), 555 (Pt–N). Anal. Calcd (For (C_18_H_10_F_6_N_8_Pt) unit): C: 33.39%, H: 1.56%, N: 17.31%, Pt: 30.13%; Found: C: 33.45%, H: 1.60%, N: 17.58%, Pt: 30.18%.

#### Synthesis of [Pt(L^2^)(4-DMAP)], 2-DMAP

A similar procedure used for **1-DMAP** was followed in the synthesis of **DMAP** (5.1 mg, 0.015 mmol of DMAP) but using **[Pt(DMSO)L**^**2**^**]** instead of **[Pt(DMSO)L**^**1**^**]**. A brown solid was obtained after filtration (11.7 mg, 77% yield). IR (cm^–1^): ν= 3412 (N–H), 3087 (CH_ar_), 2980 (C–H), 2851 (N–CH_3_), 1610 (C=C), 1472 (−C=N), 1152 (−CF_3_), 546 (Pt–N). Anal. Calcd (For (C_213_H_173_Cl_5_F_30_N_45_Pt_5_) unit): C: 50.30%, H: 3.43%, N: 12.39%, Pt: 19.18%; Found: C: 50.14%, H: 3.40%, N: 12.33%, Pt: 19.12%.

#### Synthesis of [Pt(L^2^)(4-Vynylpyridine)], 2-PV

A similar procedure used for **1-PV** was followed in the synthesis of **2-PV** (3.3 mg, 0.030 mmol of PV) but using **[Pt(DMSO)L**^**2**^**]** instead of **[Pt(DMSO)L**^**1**^**]**. A brown solid was obtained after filtration (14.3 mg, 69% yield). IR (cm^–1^): ν = 3421 (N–H), 3092 (CH_ar_), 2985 (C–H), 2705 (−CH_2_), 1618 (C=C), 1476 (−C=N), 1156 (−CF_3_), 559 (Pt–N). Anal. Calcd (For (C_18_H_9_ClF_6_N_8_Pt) unit): C: 31.71%, H: 1.33%, N: 16.43%, Pt: 28.61%; Found: C: 31.65%, H: 1.39%, N: 16.62%, Pt: 28.81%.

#### Synthesis of [Pt(L^3^)(4-DMAP)], 3-DMAP

A similar procedure used for **1-DMAP** was followed in the synthesis of **3-DMAP** (6.8 mg, 0.02 mmol of DMAP) but using **[Pt(DMSO)L**^**3**^**]** instead of **[Pt(DMSO)L**^**1**^**]**. A yellow solid was obtained after filtration (12.2 mg, 72% yield). IR (cm^–1^): ν = 3412 (N–H), 3083 (C-Har), 2918 (C–H), 2843 (NCH_3_), 1628 (C–C), 1459 (−C–N). Anal. Calcd (For (C_193_H_178_N_55_Pt_5_) unit): C: 54.63%, H: 4.23%, N: 18.16%, Pt: 22.99%; Found: C: 54.60%, H: 4.19%, N: 18.12%, Pt: 22.94%.

#### Synthesis of [Pt(L^3^)(4-Vynylpyridine)], 3-PV

A similar procedure used for **1-PV** was followed in the synthesis of **3-PV** (4.4 mg, 0.041 mmol of PV) but using **[Pt(DMSO)L**^**3**^**]** instead of **[Pt(DMSO)L**^**1**^**]**. An orange solid was obtained after filtration (19.6 mg, 93% yield). IR (cm^–1^): ν = 3412 (N–H), 3083 (C–H_ar_), 2918 (C–H), 1628 (C=C), 1459 (−C=N). Anal. Calcd (For (C_14_H_10_N_10_Pt) unit): C: 32.75%, H: 1.96%, N: 27.28%, Pt: 38.00%; Found: C: 32.81%, H: 2.01%, N: 27.42%, Pt: 38.14%.

#### Synthesis of [Pt(L^4^)(4-DMAP)], 4-DMAP

A similar procedure used for **1-DMAP** was followed in the synthesis of **4-DMAP** (6.4 mg, 0.02 mmol of DMAP) but using **[Pt(DMSO)L**^**4**^**]** instead of **[Pt(DMSO)L**^**1**^**]**. A pale-brown solid was obtained after filtration (13.4 mg, 81% yield). IR (cm^–1^): ν = 3333 (N–H), 3053 (C-Har), 2921 (C–H), 2852 (N–CH_3_), 1638 (C=C), 1459 (−C=N). Anal. Calcd (For (C_193_H_173_Cl_5_N_55_Pt_5_) unit): C: 52.50%, H: 3.95%, N: 17.45%, Pt: 22.09%; Found: C: 52.45%, H: 3.92%, N: 17.43%, Pt: 22.04%.

#### Synthesis of [Pt(L^4^)(4-vynylpyridine)], 4-PV

A similar procedure used for **1-PV** was followed in the synthesis of **4-PV** (4.2 mg, 0.038 mmol of PV) but using **[Pt(DMSO)L**^**4**^**]** instead of **[Pt(DMSO)L**^**1**^**]**. An orange solid was obtained after filtration (18.5 mg, 88% yield). IR (cm^–1^): ν = 3425 (N–H), 2923 (C–H), 2754 (−CH_2_), 1641 (C=C), 1454 (−C=N), 555 (Pt–N). Anal. Calcd (For (C_14_H_9_ClN_10_Pt) unit): C: 30.69%, H: 1.66%, N: 25.57%, Pt: 35.61%; Found: C: 30.74%, H: 1.69%, N: 25.62%, Pt: 35.66%.

#### Synthesis of [Pt(L^3^)(4-Dimethylaminopyridine)], 1A

A similar procedure used for **1-DMAP** was followed in the synthesis of **1A** but using 4-dimethylaminopyridine instead of 4-(dimethylamine)pyridine polymer. A pale-yellow solid was obtained after filtration (18.2 mg, 84% yield). ^1^H NMR (400 MHz, DMSO-d^6^) δ= 8.87–8.83 (m, 2H, *H*_*3,5-Py*_), 8.33–8.25 (m, 1H, *H*_*4-Py*_), 7.98 (d, *J* = 8.0 Hz, 2H, *H*_*2,6-PyNMe*_), 6.99–6.87 (m, 2H, *H*_*3,5-PyNMe*_), 3.13 (s, 6H, *H*). ^19^F NMR (471 MHz, DMSO-d^6^) δ = −64.6 ppm. ESI (*m*/*z*): 666.2 [**1A** + H^+^]^+^, 655.1 [**1A** – CH_3_]^+^, 635.2 [**1A** – 2 CH_3_]^+^. IR (cm^–1^): ν = 3068 (C–H_ar_), 2927 (C–H), 2358 (N–CH), 1633 (C–N), 1486 (−CH_3_), 1145 (−CF_3_), 811 (Pt–N).

#### Synthesis of [Pt(L^1^)(4-Methylpyridine)], 1B

A similar procedure used for **1-PV** was followed in the synthesis of **1B** but using 4-methylpyridine instead of poly(4-vinylpyridine) polymer. A pale brown solid was obtained after filtration (8.7 mg, 43% yield). ^1^H NMR (500 MHz, CDCl_3_) δ= 9.53–9.47 (m, 2H, *H*_*3,5-Py*_), 8.06 (t, *J* = 7.9 Hz, 1H, *H*_*4-Py*_), 7.87 (d, *J* = 7.9 Hz, 2H, *H*_*2,6-PyMe*_), 7.49–7.43 (m, 2H, *H*_*3,5-PyMe*_), 2.54 (s, 3H, ) ppm. ^19^F NMR (471 MHz, CDCl_3_) δ= −64.3 ppm. ESI (*m*/*z*): 659.11 [**1B** + Na^+^]^+^, 636.06 [**1B** + H^+^]^+^. IR (cm^–1^): ν = 3034 (C–H_ar_), 2948 (C–H), 2372 (N–CH), 1634 (C–N), 1482 (−CH_3_), 1125 (−CF_3_), 814 (Pt–N).

#### Synthesis of [Pt(L^4^)(4-Dimethylaminopyridine)], 2A

A similar procedure used for **2-DMAP** was followed in the synthesis of **2A** but using 4-dimethylaminopyridine instead of 4-(dimethylamine)pyridine polymer. A pale-yellow solid was obtained after filtration (17.8 mg, 83% yield). ^1^H 0 NMR (400 MHz, DMSO-d^6^) δ = 8.76–8.70 (m, 2H, H*_3, 5-Py_*), 8.19 (d, *J* = 6.4 Hz, 1H, H*_2-PyNMe_*), 8.02 (d, *J* = 1.2 Hz, 1H, H*_6-PyNMe_*), 6.91 (d, *J* = 6.6 Hz, 1H, H*_3-PyNMe_*), 6.77 (d, *J* = 6.8 Hz, 1H, H*_5-PyNMe_*), 3.12 (d, *J* = 11.7 Hz, 6H, H). ^19^F NMR (471 MHz, DMSO-d^6^) δ = −63.4 ppm. ESI (*m*/*z*): 693.1[**2A** + H^+^]^+^, 664.1 [**2A** – Cl + H^+^]^+^, 655.1 [**2A -** Cl – CH_3_]^+^, 635.2 [**2A**–Cl–2 CH_3_]^+^. IR (cm^–1^): ν = 3085 (C–H_ar_), 2939 (C–H), 2346 (N–CH), 1625 (C–N), 1444 (C–H), 1153 (−CF_3_), 817 (Pt–N), 746 (C–Cl).

#### Synthesis of [Pt(L^4^)(4-Methylpyridine)], 2B

A similar procedure used for **2-PV** was followed in the synthesis of **2B** but using 4-methylpyridine instead of poly(4-vinylpyridine) polymer. A pale-yellow solid was obtained after filtration (9.5 mg, 47% yield). ^1^H NMR (500 MHz, CDCl_3_) δ= 9.51 (d, *J* = 6.1 Hz, 2H, H*_3,5-Py_*), 7.90 (s, 2H, H*_2,6-PyMe_*), 7.50–7.45 (m, 2H, H*_3,5-PyMe_*), 2.55 (s, 3H, ) ppm. ^19^F NMR (471 MHz, CDCl_3_) δ = −64.4 ppm. ESI (*m*/*z*): 636.06 [**2B** – Cl + H^+^]^+^, 510.10 [**2B**– CF_3_ – C_6_H_7_N + H^+^]^+^. IR (cm^–1^): ν = 3088 (C–H_ar_), 2948 (C–H), 2486 (N–CH), 1631 (C–N), 1444 (C–H), 1121 (−CF3), 814 (Pt–N), 748 (C–Cl).

#### Synthesis of [Pt(L^5^)(4-Methylpyridine)], 3A

A similar procedure used for **3-DMAP** was followed in the synthesis of **3A** but using 4-dimethylaminopyridine instead of 4-(dimethylamine)pyridine polymer. A pale-orange solid was obtained after filtration (16.3 mg, 74% yield). ^1^H NMR (400 MHz, DMSO-d^6^) δ = 8.22 (d, *J* = 6.2 Hz, 2H, H*_2,6-PyNMe_*), 6.99 (d, *J* = 6.5 Hz, 2H, H*_3,5-PyNMe_*), 3.19 (s, 6H, H). ESI (*m*/*z*): 532.1[**3B** + H^+^]^+^. IR (cm^–1^): ν = 3079 (C–H_ar_), 2937 (C–H), 2366 (N–CH), 1646 (C–N), 1456 (C–H), 813 (Pt–N).

#### Synthesis of [Pt(L^5^)(4-Dimethylaminopyridine)], 3B

A similar procedure used for **3-PV** was followed in the synthesis of **3B** but using 4-methylpyridine instead of poly(4-vinylpyridine) polymer. A pale-orange solid was obtained after filtration (15.9 mg, 77% yield). ^1^H NMR (400 MHz, DMSO-d^6^) δ= 8.77–8.64 (m, 2H, H*_2,6-PyMe_*), 7.87–7.79 (m, 2H, H*_3,5-PyMe_*), 2.58 (s, 3H, ). ESI (*m*/*z*): 502.1 [**3A** + H^+^]^+^, 477.1 [**3A** – N_2_ + H^+^]^+^. IR (cm^–1^): ν = 3077 (C–H_ar_), 2927 (C–H), 2422 (N–CH), 1619 (C–N), 1384 (C–H), 819 (Pt–N).

#### Synthesis of [Pt(L^6^)(4-Methylpyridine)], 4A

A similar procedure used for **4-DMAP** was followed in the synthesis of **4A** but using 4-dimethylaminopyridine instead of 4-(dimethylamine)pyridine polymer. A pale-orange solid was obtained after filtration (17.3 mg, 79% yield). ^1^H NMR (400 MHz, DMSO-d^6^) δ= 8.19 (d, *J* = 7.0 Hz, 2H, H*_2,6-PyNMe_*), 6.94 (dd, *J* = 14.8, 6.8 Hz, 2H, H*_3,5-PyNMe_*), 3.16 (s, 6H, H). ESI (*m*/*z*): 584.0 [**4B** + NH_4_^+^]^+^, 568.0 [**4B** + H^+^]^+^. IR (cm^–1^): ν = 3085 (C–H_ar_), 2954 (C–H), 2416 (N–CH), 1645 (C–N), 1442 (C–H), 809 (Pt–N), 620 (C–Cl).

#### Synthesis of [Pt(L^6^)(4-Dimethylaminopyridine)], 4B

A similar procedure used for **4-PV** was followed in the synthesis of **4B** but using 4-methylpyridine instead of poly(4-vinylpyridine) polymer. A pale-orange solid was obtained after filtration (15.4 mg, 75% yield). ^1^H NMR (400 MHz, DMSO-d^6^) δ = 8.70 (d, *J* = 6.0 Hz, 2H, H*_2,6-PyMe_*), 7.75 (d, *J* = 5.7 Hz, 2H, H*_3,5-PyMe_*), 2.53 (s, 3H, ). ESI (*m*/*z*): 555.0 [**4A** + NH_4_^+^]^+^, 539.1 [**4A** + H^+^]^+^. IR (cm^–1^): ν = 3060 (C–H_ar_), 2983 (C–H), 2424 (N–CH), 1641 (C–N), 1384 (C–H), 809 (Pt–N), 620 (C–Cl).

## Supporting Information Available

Characterization data of the compounds, emission lifetime decays; optimized geometries calculated for the dimers at different disposition; HOMO–LUMO plots of the compounds, representation of the calculated oligomer **3-PV**; Interaction energies (Eint, kcal/mol) computed for the dimers of compounds, binding energies using the unrestricted UKS-PBE0-d4/def2-TZVP level of theory, and Cartesian coordinates of the optimized geometries (PDF)
