# Supplementary material for: Investigating the Impact of Packing and Environmental Factors on the Luminescence of Pt(N^N^N) Chromophores
Source: Inorg Chem. 2024 Jan 23;63(5):2821–32. doi: 10.1021/acs.inorgchem.3c04562 (PMC10848268; doi:10.1021/acs.inorgchem.3c04562)
Supplement: Supplementary file 1 — ic3c04562_si_001.pdf [file ic3c04562_si_001.pdf]

# Investigating the Impact of Packing and Environmental Factors on the Luminescence of Pt(N^N^N) Chromophores

Guillermo Romo-Islas,[a] Sergi Burguera,[b] Antonio Frontera[b,*] and Laura Rodríguez[a,*]

[a] *Departament de Química Inorgànica i Orgànica, Secció de Química Inorgànica. Institut de Nanociència i Nanotecnologia (IN2UB). Universitat de Barcelona, Martí i Franquès 1-11, E-08028 Barcelona, Spain. e-mail: laurarodriguezr@ub.edu*

[b] *Departament de Química, Universitat de les Illes Balears, 07122 Palma de Mallorca, Spain. e-mail: toni.frontera@uib.es*

# Supporting Information

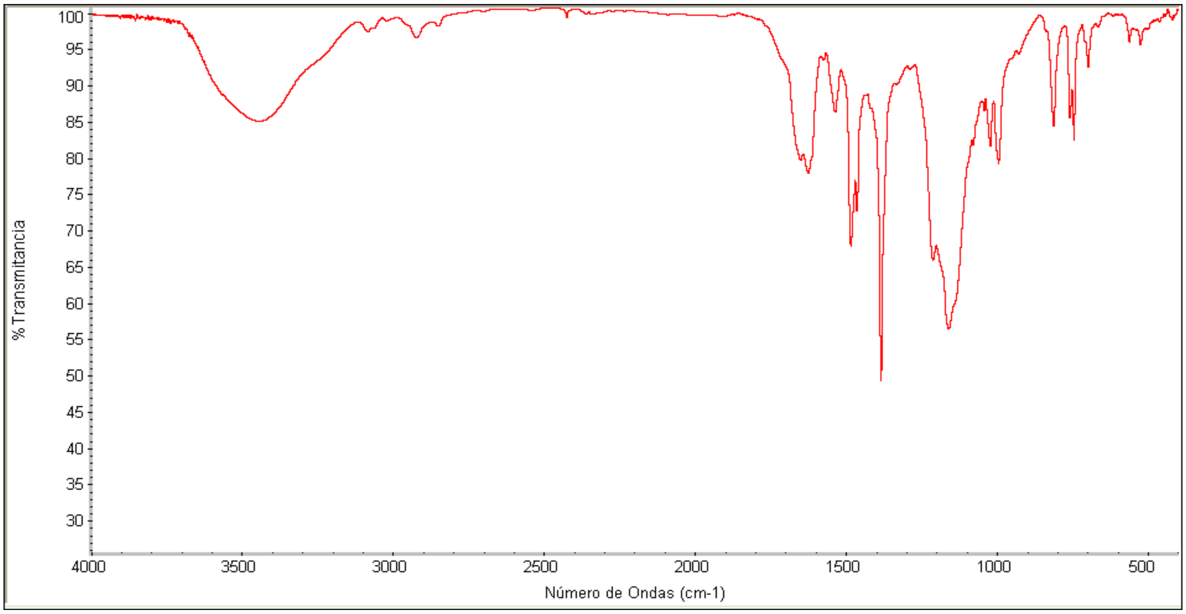


**Figure S1**. ATR IR spectrum of complex **1-DMAP** in KBr pellets.

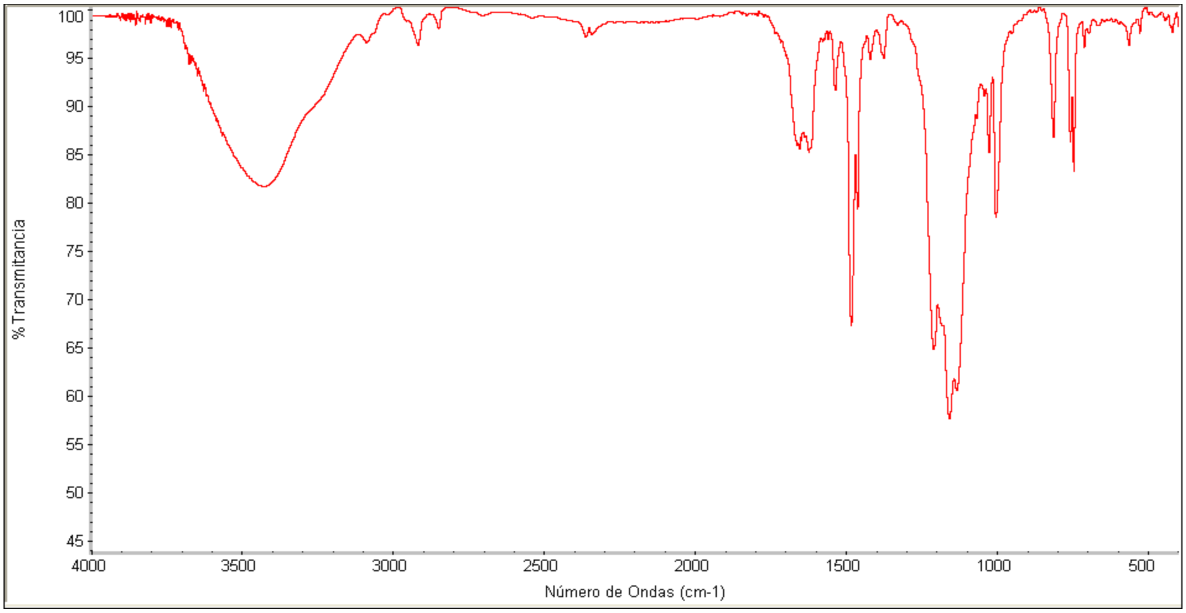


**Figure S2**. ATR IR spectrum of complex **1-PV** in KBr pellets.

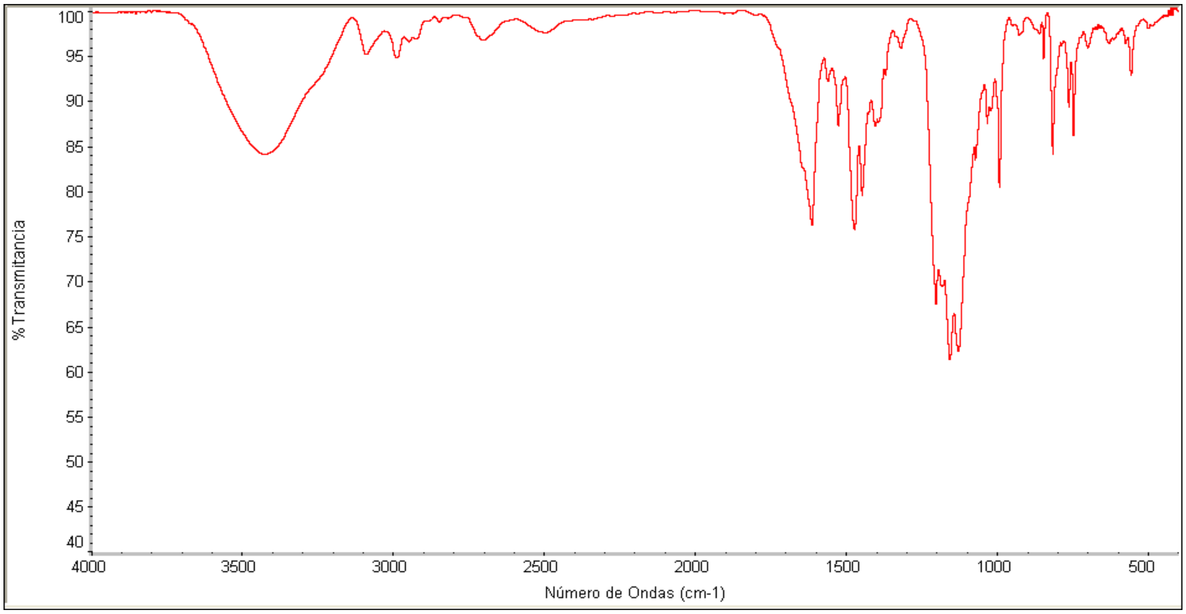


**Figure S3**. ATR IR spectrum of complex **2-DMAP** in KBr pellets.

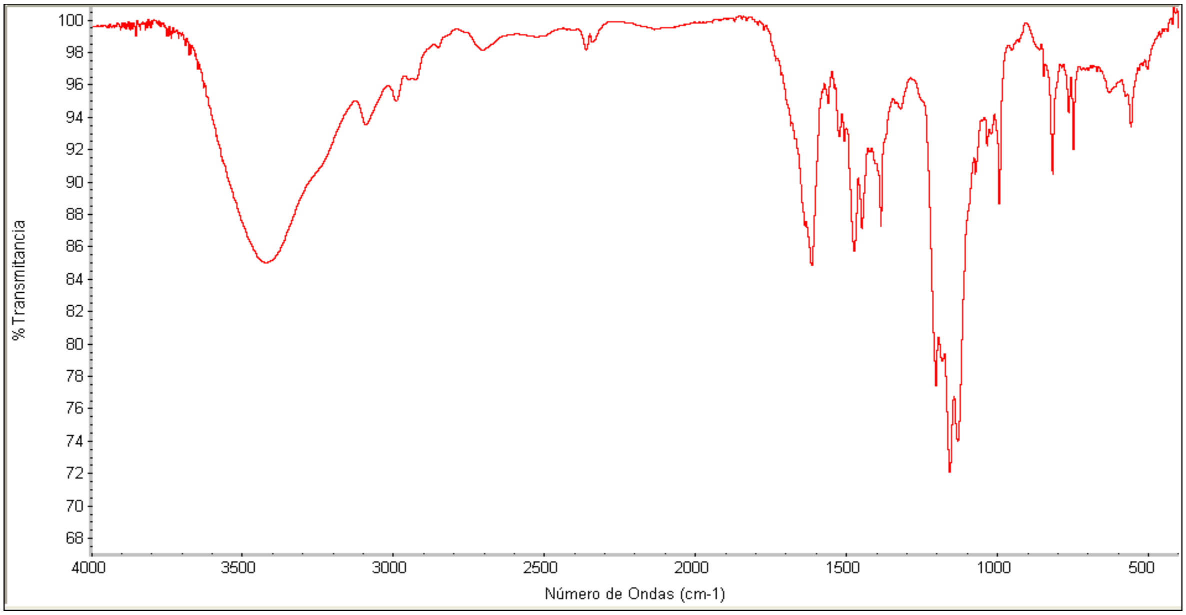


**Figure S4**. ATR IR spectrum of complex **2-PV** in KBr pellets.

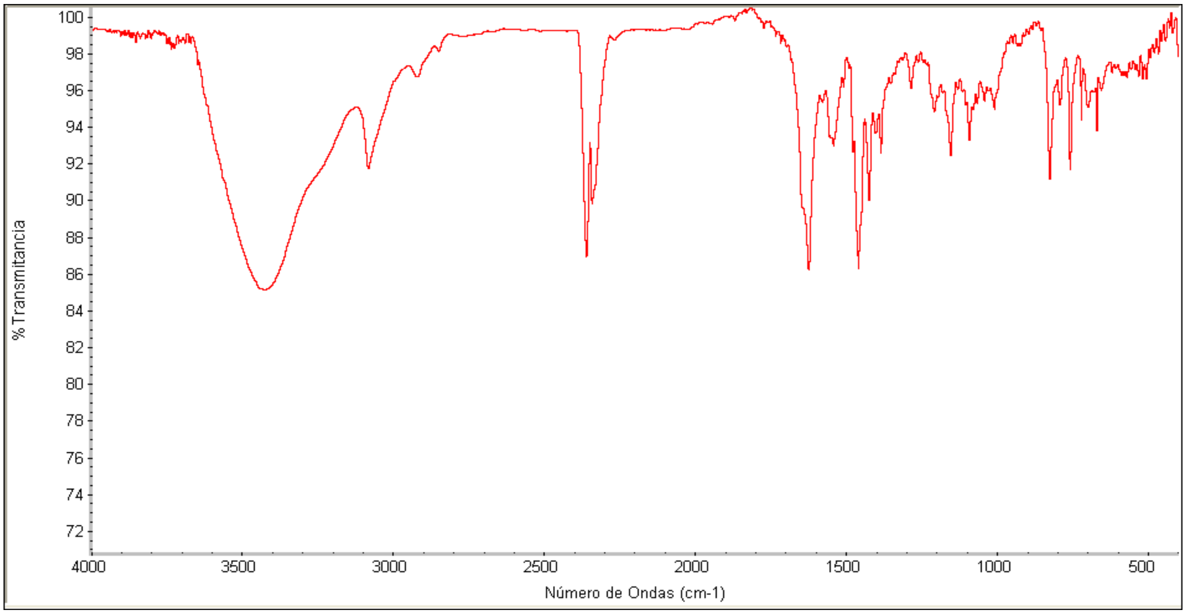


**Figure S5**. ATR IR spectrum of complex **3-DMAP** in KBr pellets.

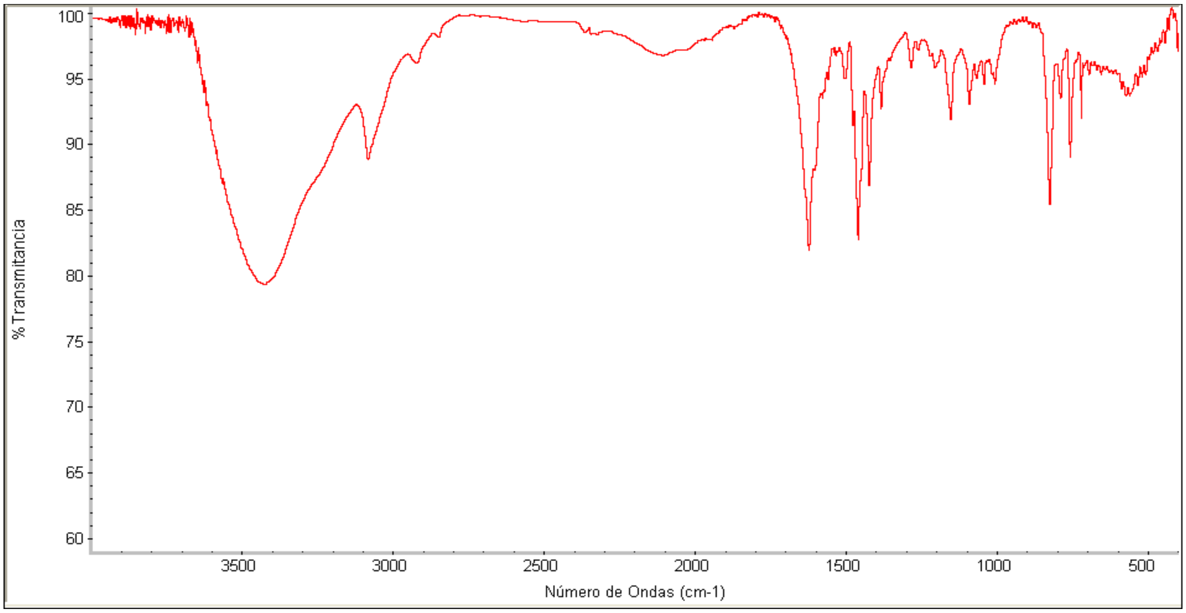


**Figure S6**. ATR IR spectrum of complex **3-PV** in KBr pellets.

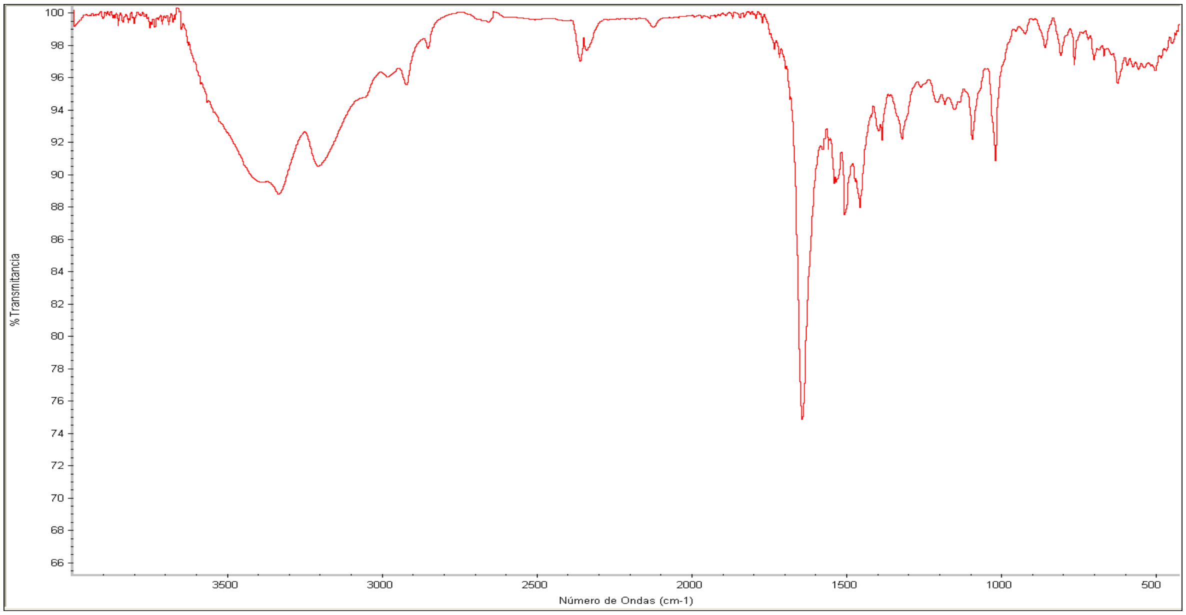


**Figure S7**. ATR IR spectrum of complex **4-DMAP** in KBr pellets.

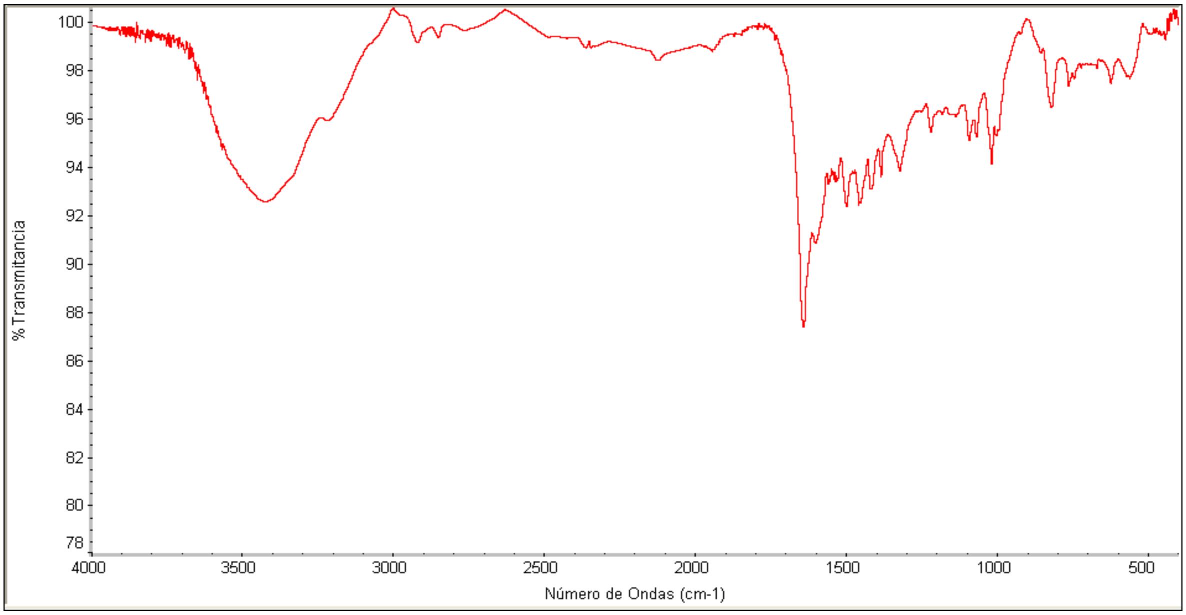


**Figure S8**. ATR IR spectrum of complex **4-PV** in KBr pellets.

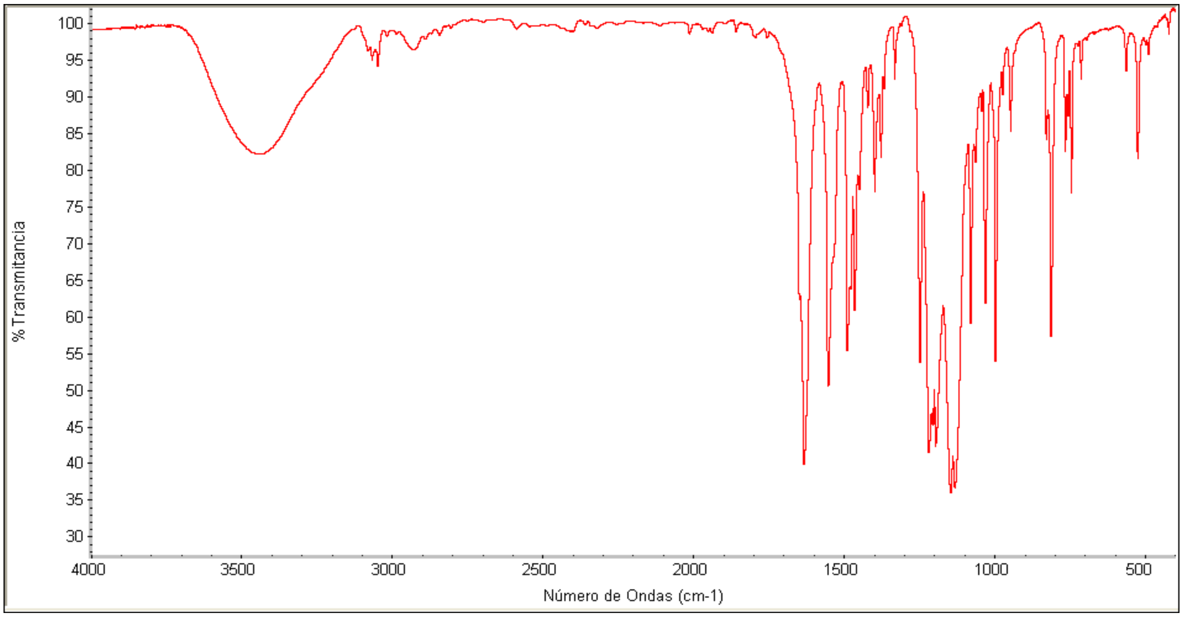


**Figure S9**. ATR IR spectrum of complex **1A** in KBr pellets.

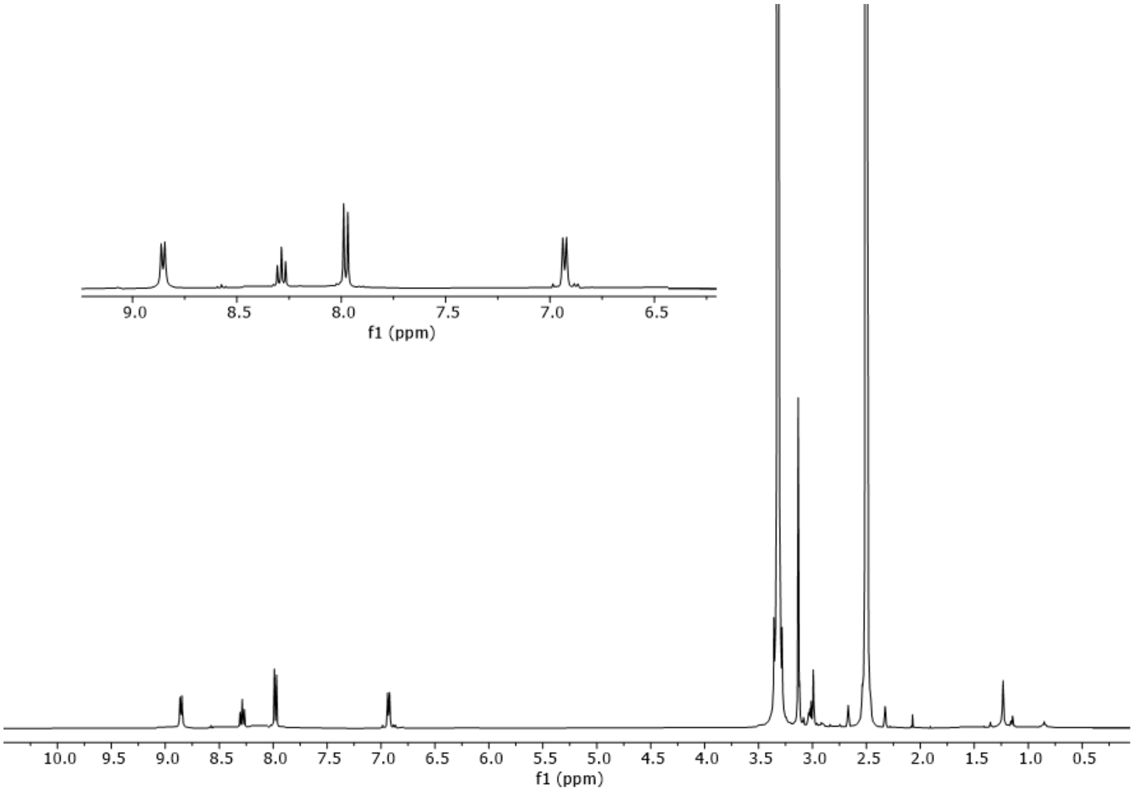


**Figure S10**. $^{1}$H NMR spectrum of complex **1A** in DMSO-d$^{6}$.

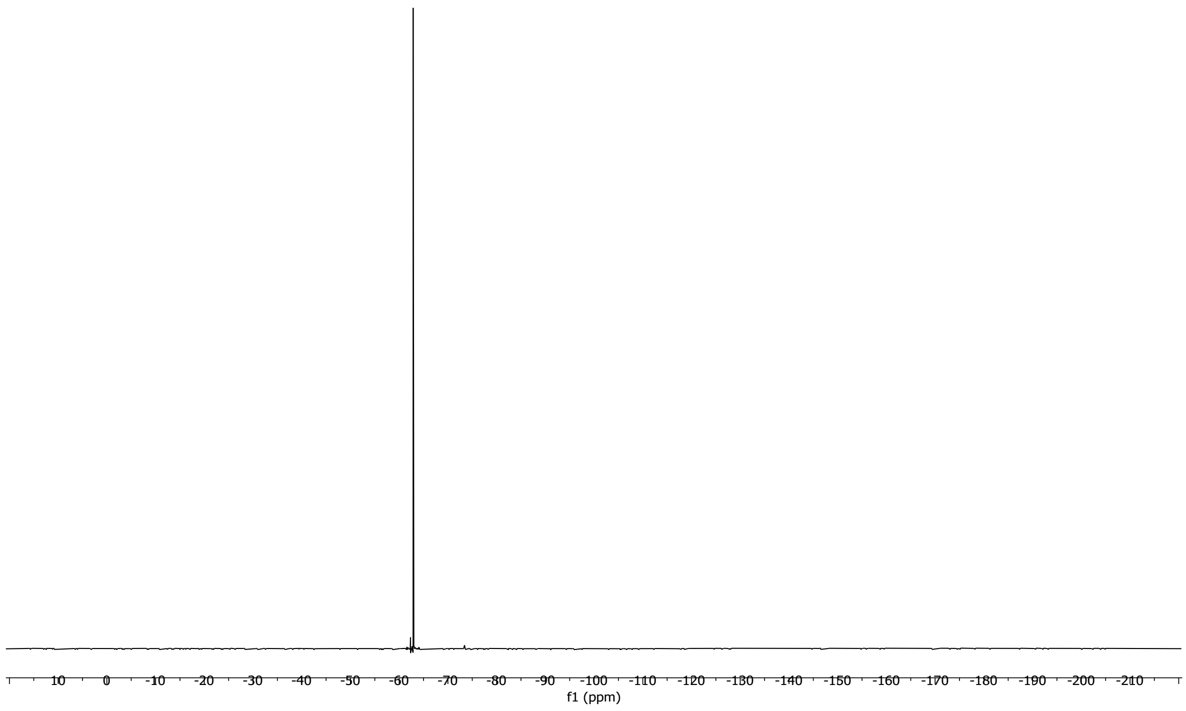


**Figure S11**. $^{19}F$ NMR spectrum of complex **1A** in DMSO-d[6].

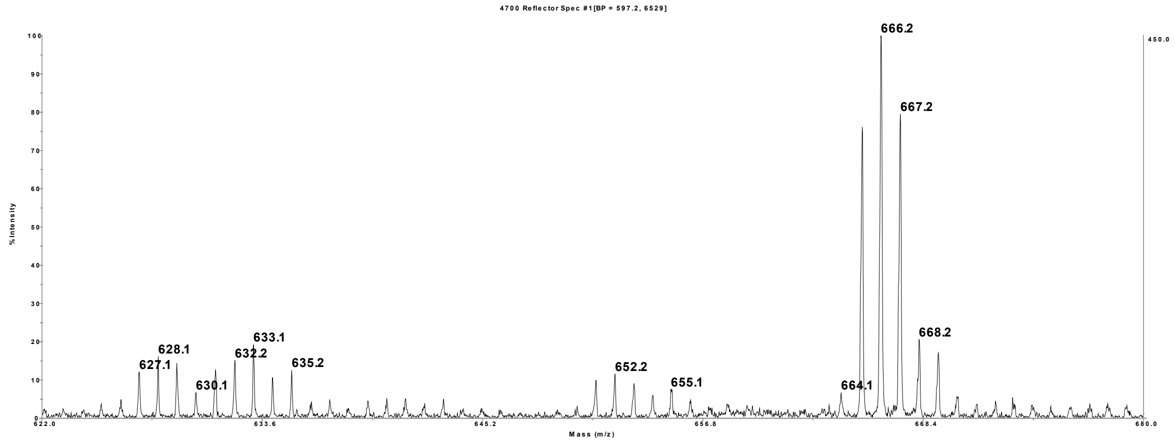


**Figure S12**. MALDI-TOF(+) of complex **1A**.

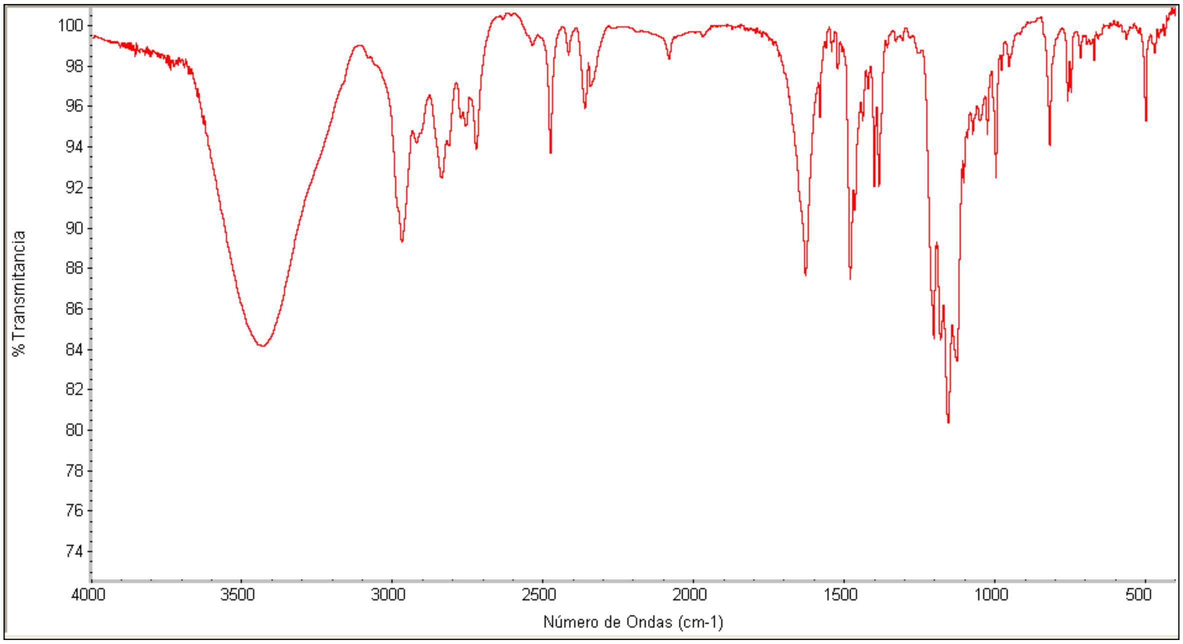


**Figure S13**. ATR IR spectrum of complex **1B** in KBr pellets.

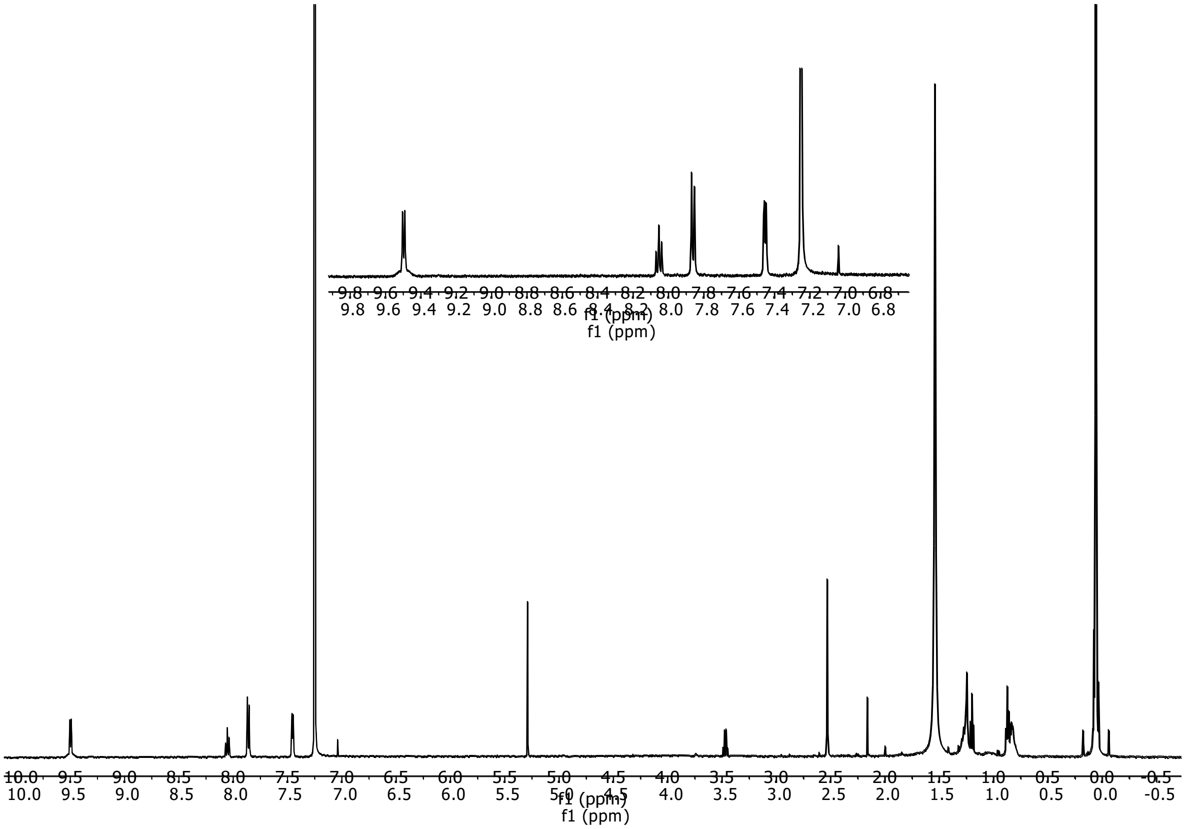


**Figure S14**. $^{1}$H NMR spectrum of complex **1B** in $CDCl_3$.

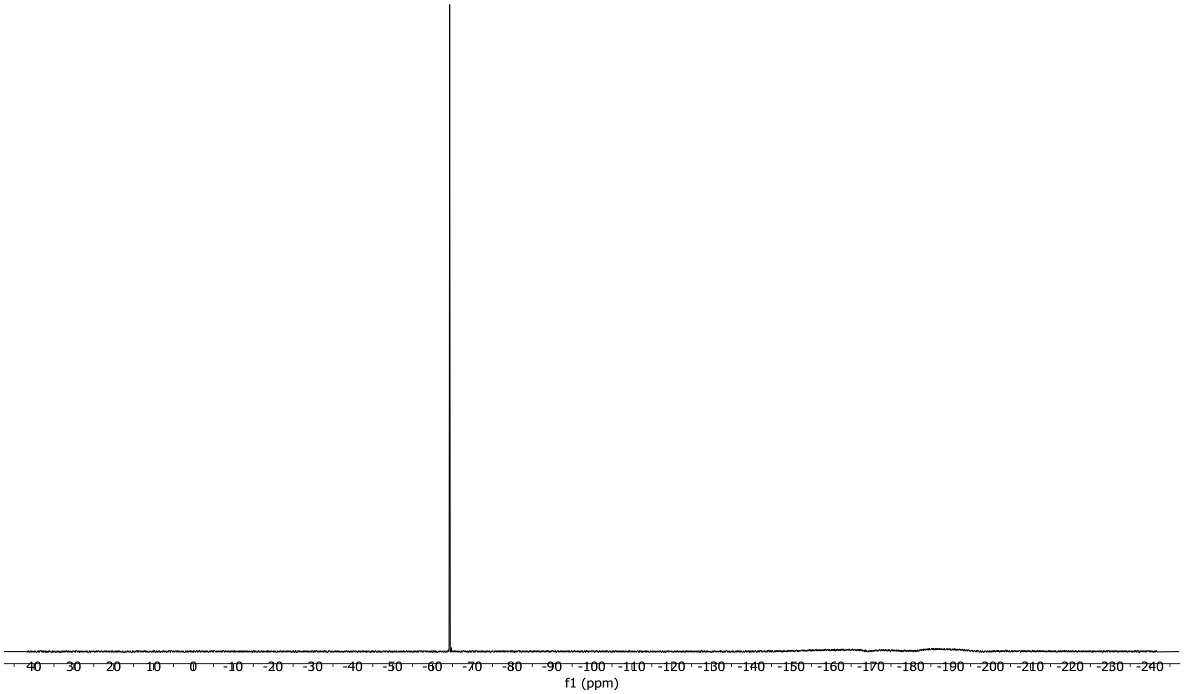


**Figure S15**. $^{19}F$ NMR spectrum of complex **1B** in $CDCl_3$.

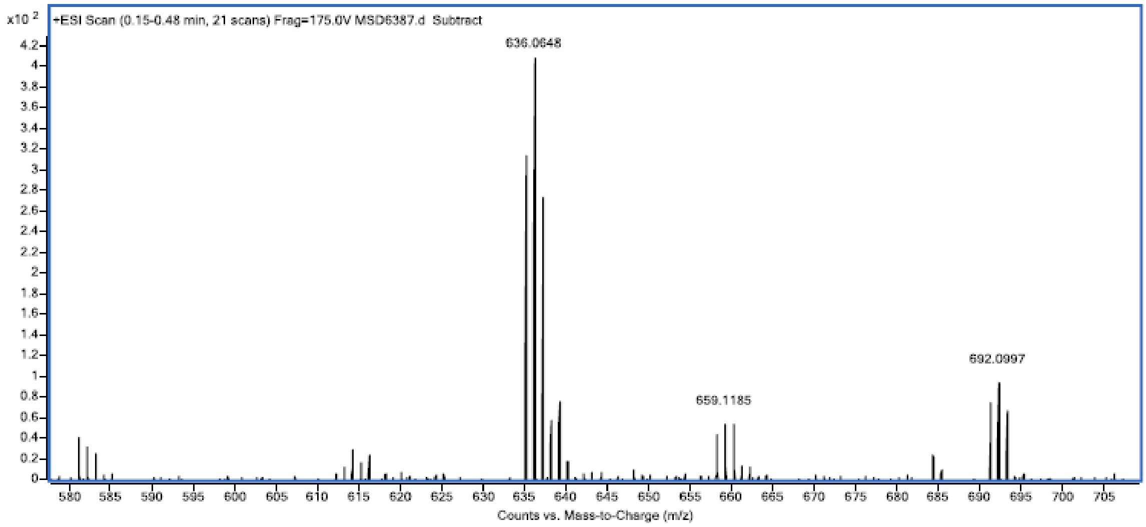


**Figure S16**. ESI-TOF(+) of complex **1B**.

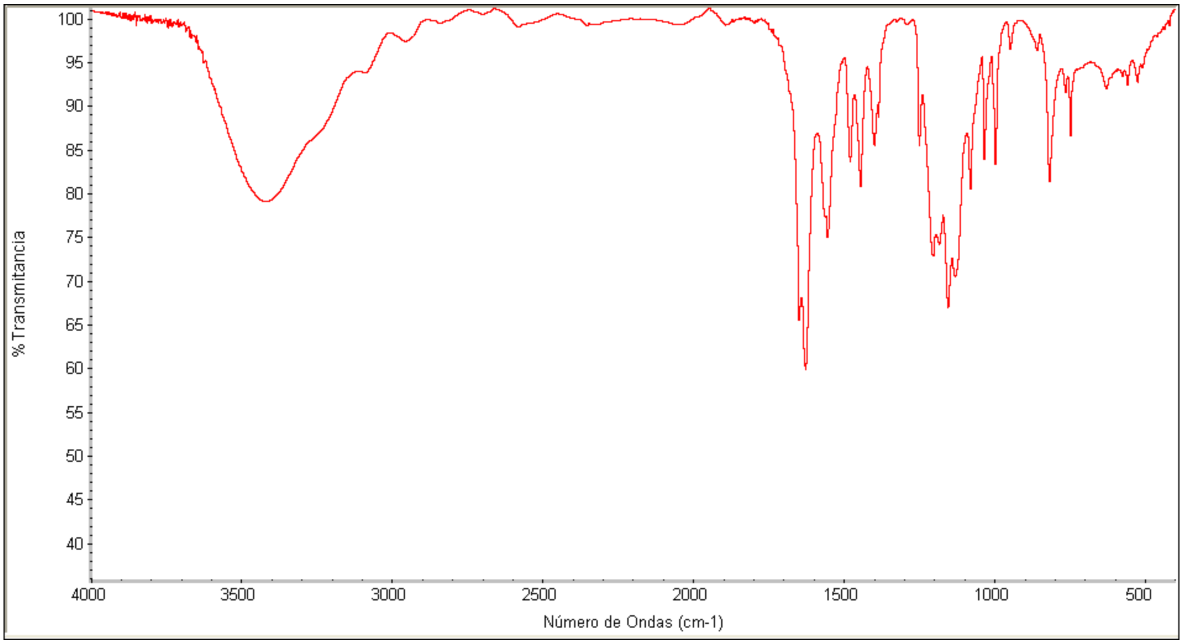


**Figure S17**. ATR IR spectrum of complex **2A** in KBr pellets.

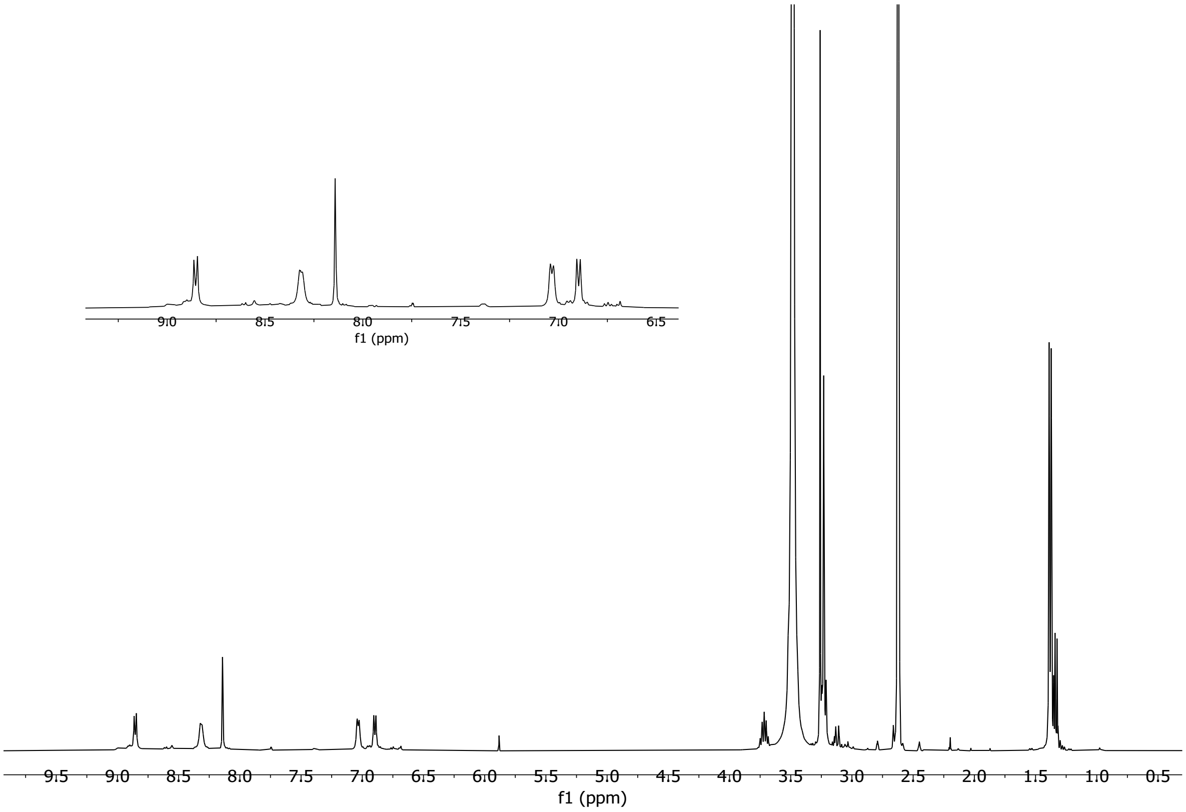


**Figure S18**. $^{1}$H NMR spectrum of complex **2A** in DMSO-d$^{6}$.

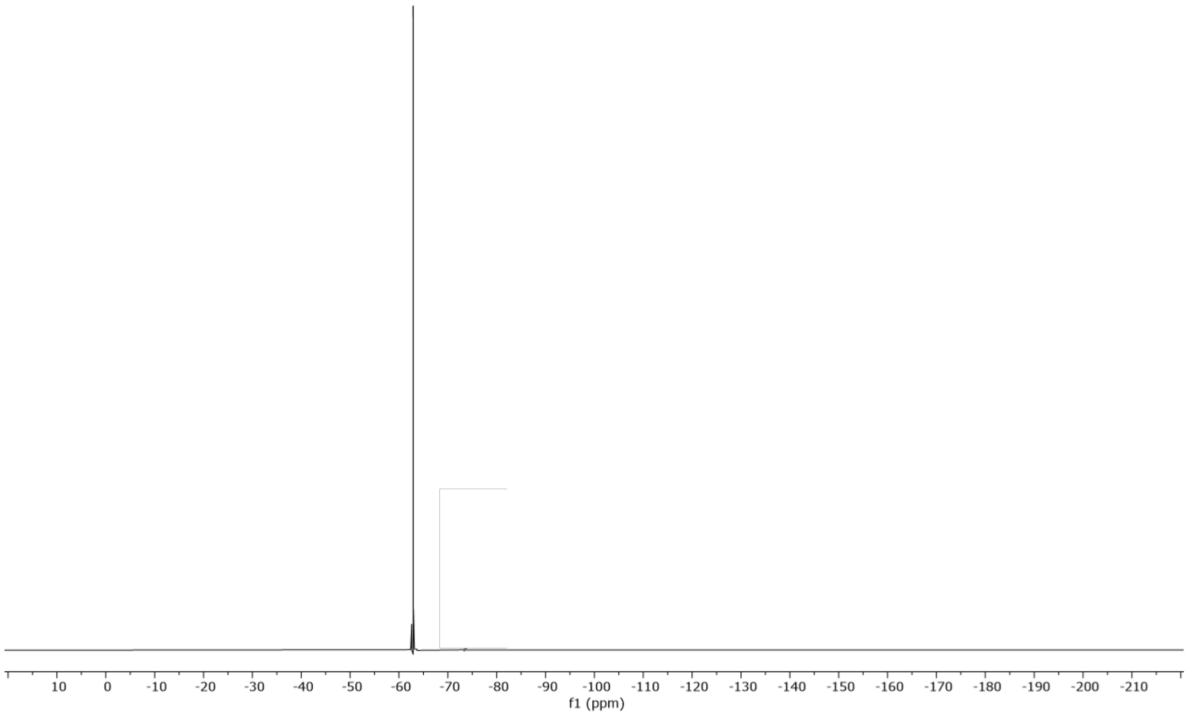


**Figure S19**. $^{19}$F NMR spectrum of complex **2A** in DMSO-d$^{6}$.

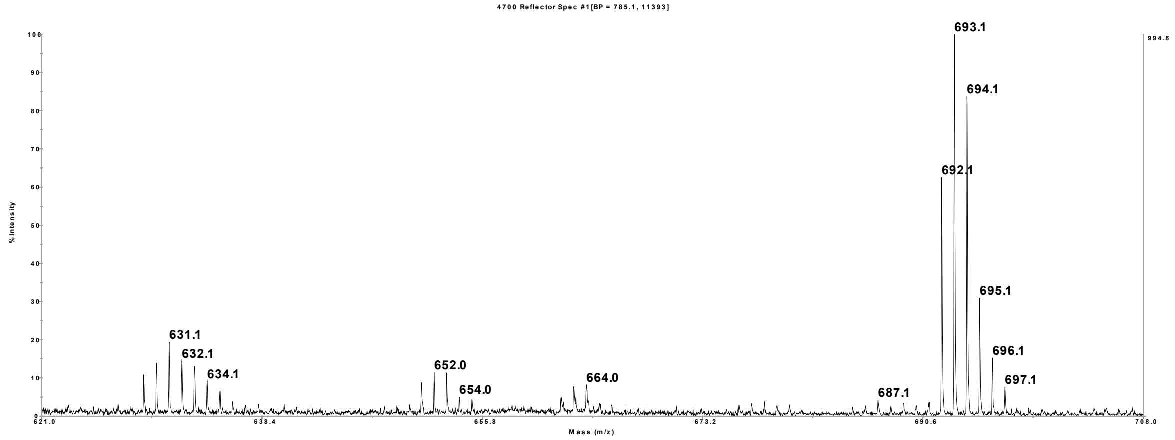


**Figure S20**. MALDI-TOF(+) of complex **2A**.

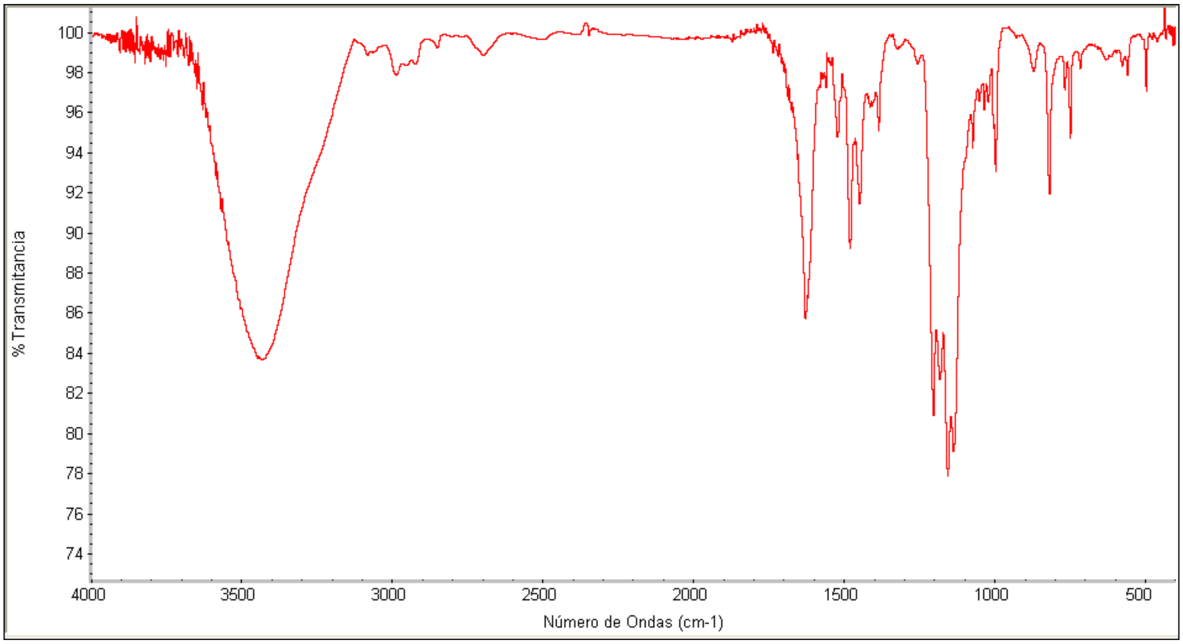


**Figure S21**. ATR IR spectrum of complex **2B** in KBr pellets.

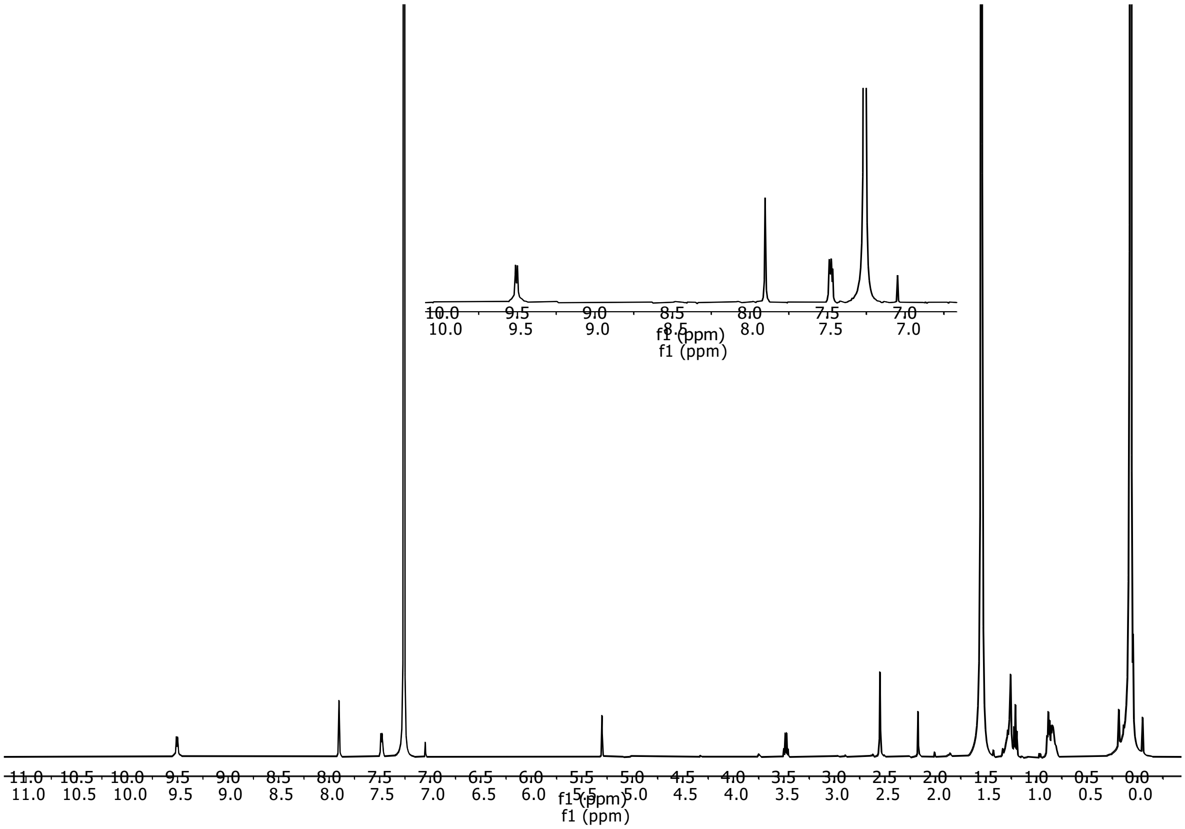


**Figure S22**. $^{1}$H NMR spectrum of complex **2B** in $CDCl_3$.

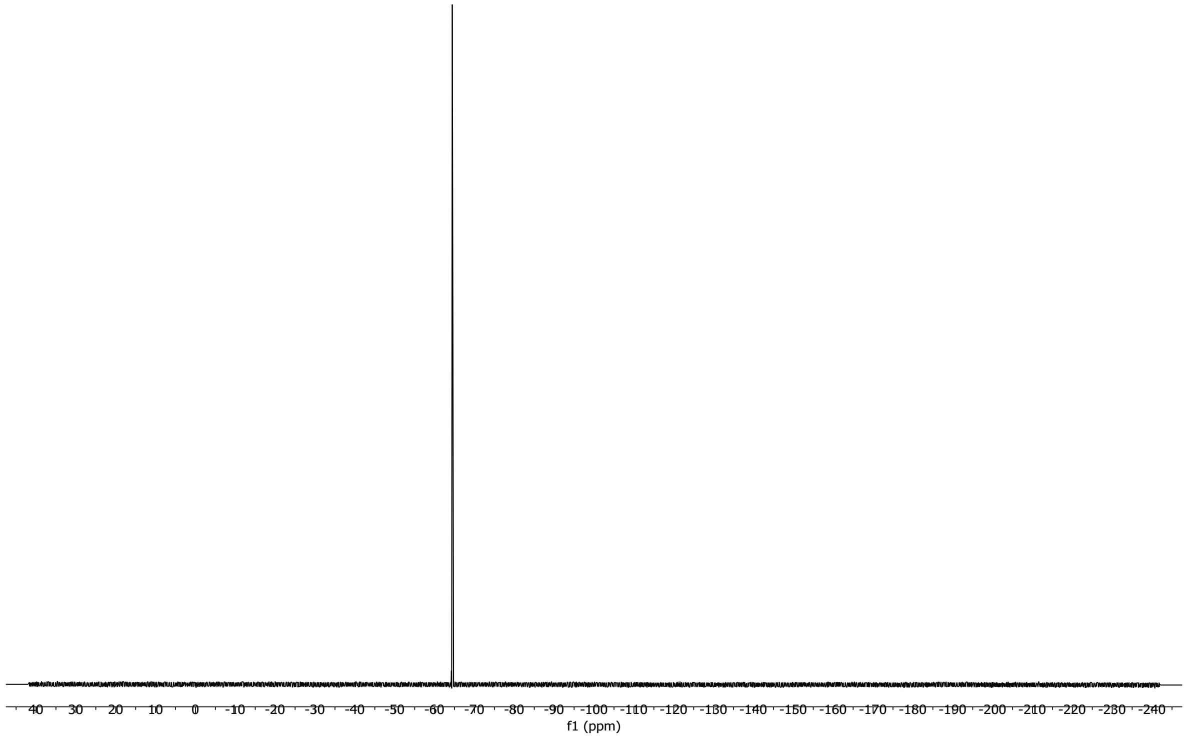


**Figure S23**. $^{19}$F NMR spectrum of complex **2B** in $CDCl_3$.

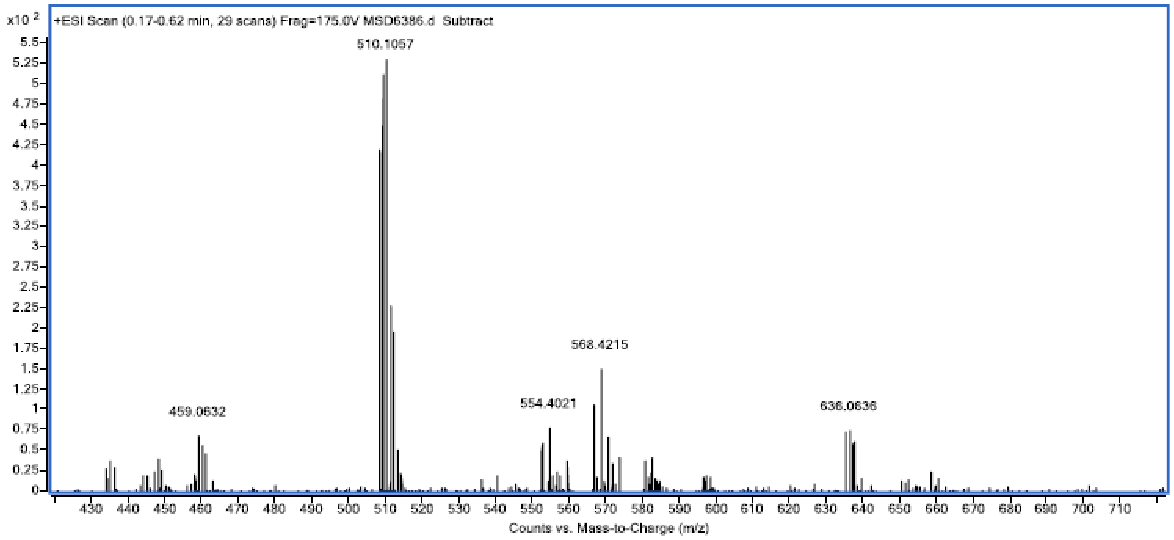


**Figure S24**. ESI-TOF(+) of complex **2B**.

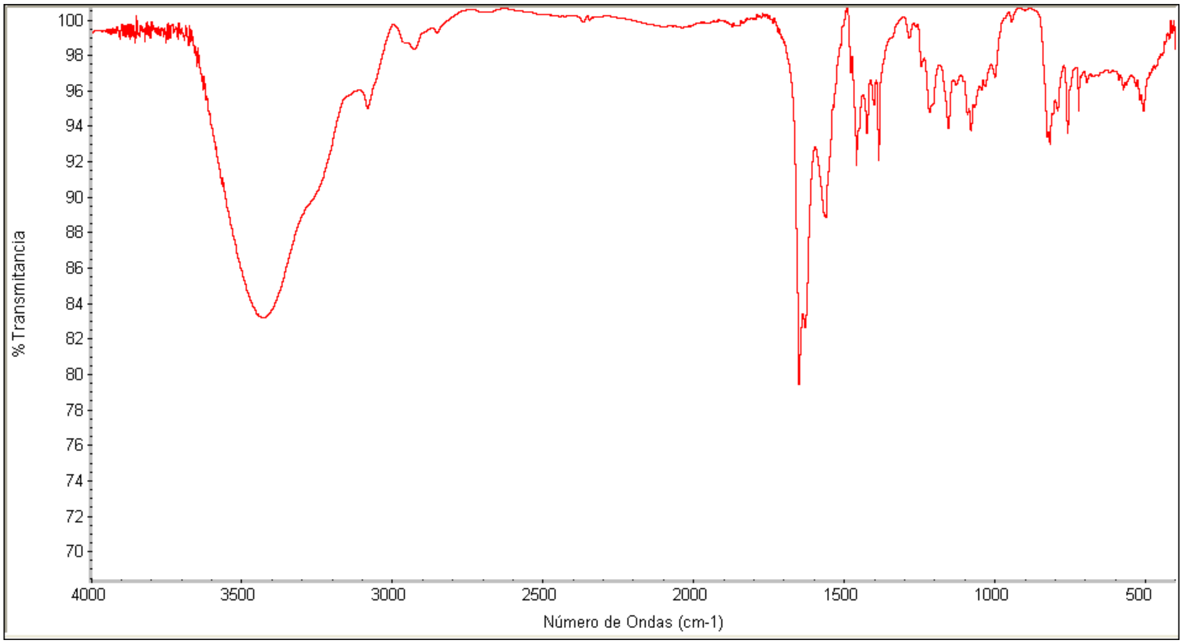


**Figure S25**. ATR IR spectrum of complex **3A** in KBr pellets.

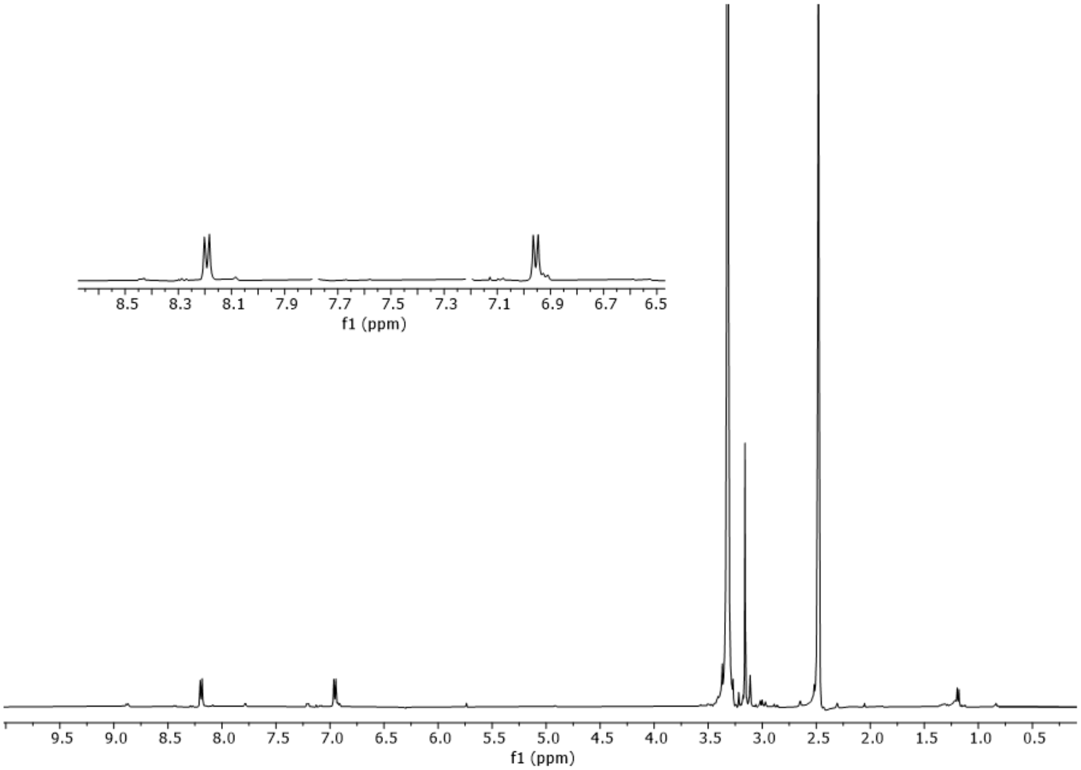


**Figure S26**. $^{1}H$ NMR spectrum of complex **3A** in DMSO-$d^6$.

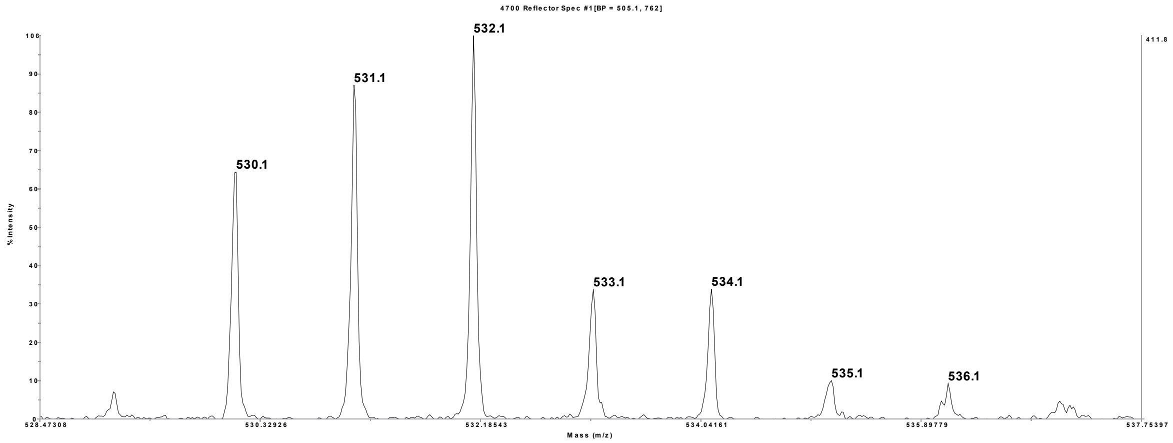


**Figure S27**. MALDI-TOF(+) of complex **3A**.

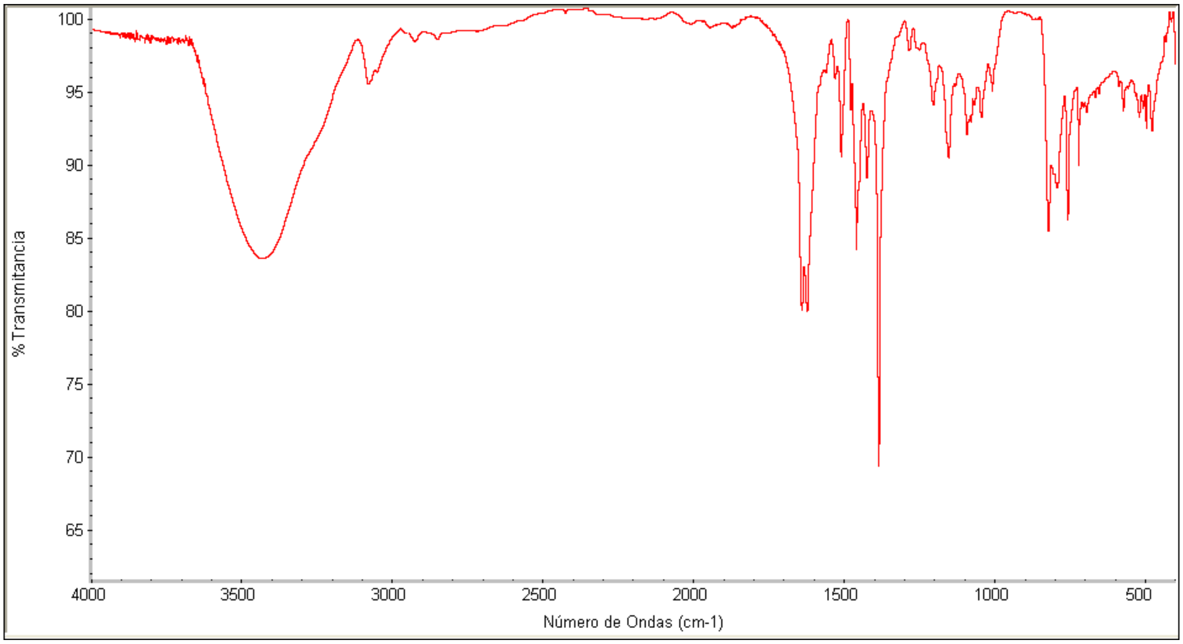


**Figure S28**. ATR IR spectrum of complex **3B** in KBr pellets.

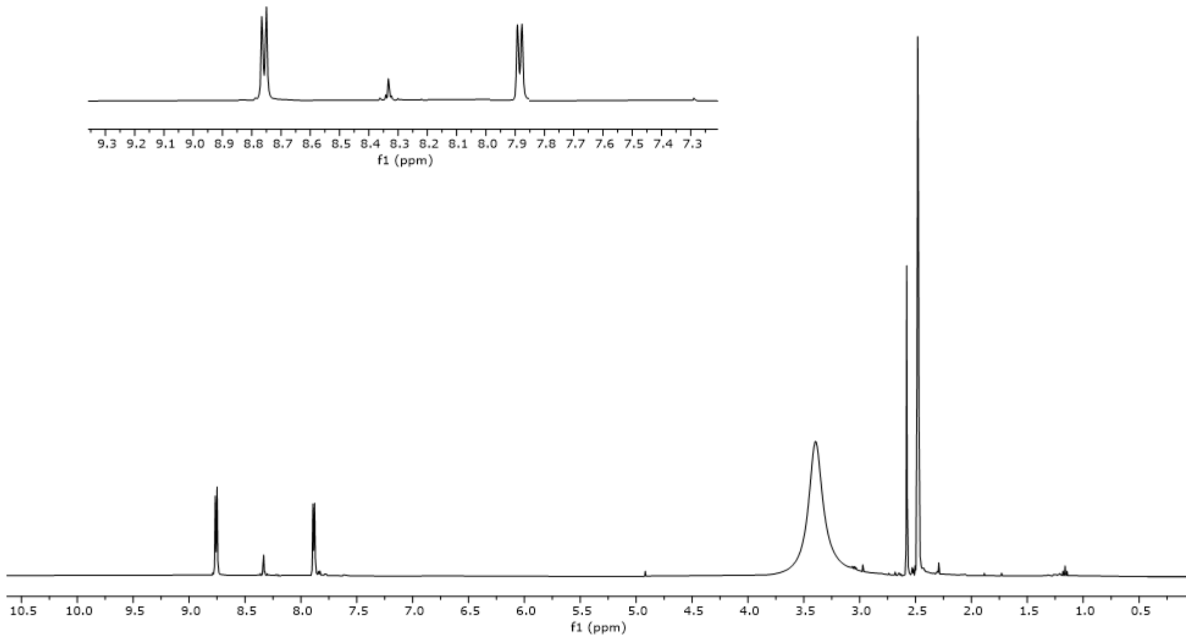


**Figure S29**. $^{1}$H NMR spectrum of complex **3B** in DMSO-d$^{6}$.

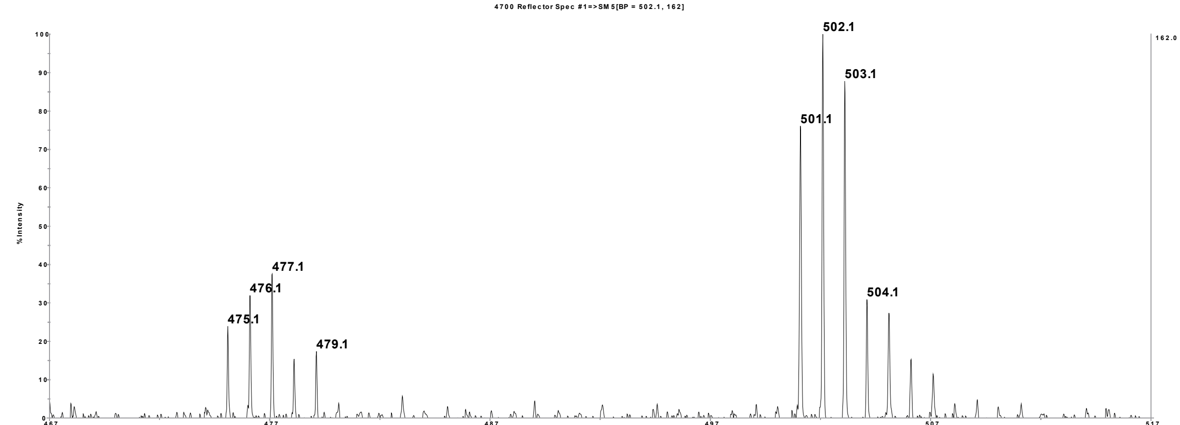


**Figure S30**. MALDI-TOF(+) of complex **3B**.

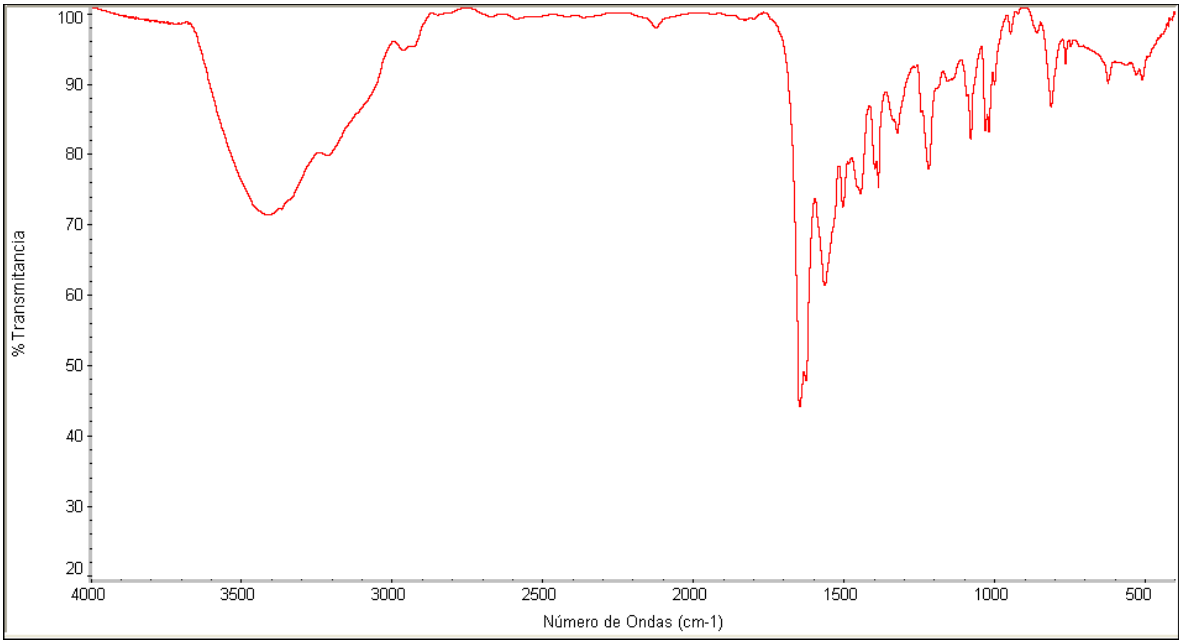


**Figure S31**. ATR IR spectrum of complex **4A** in KBr pellets.

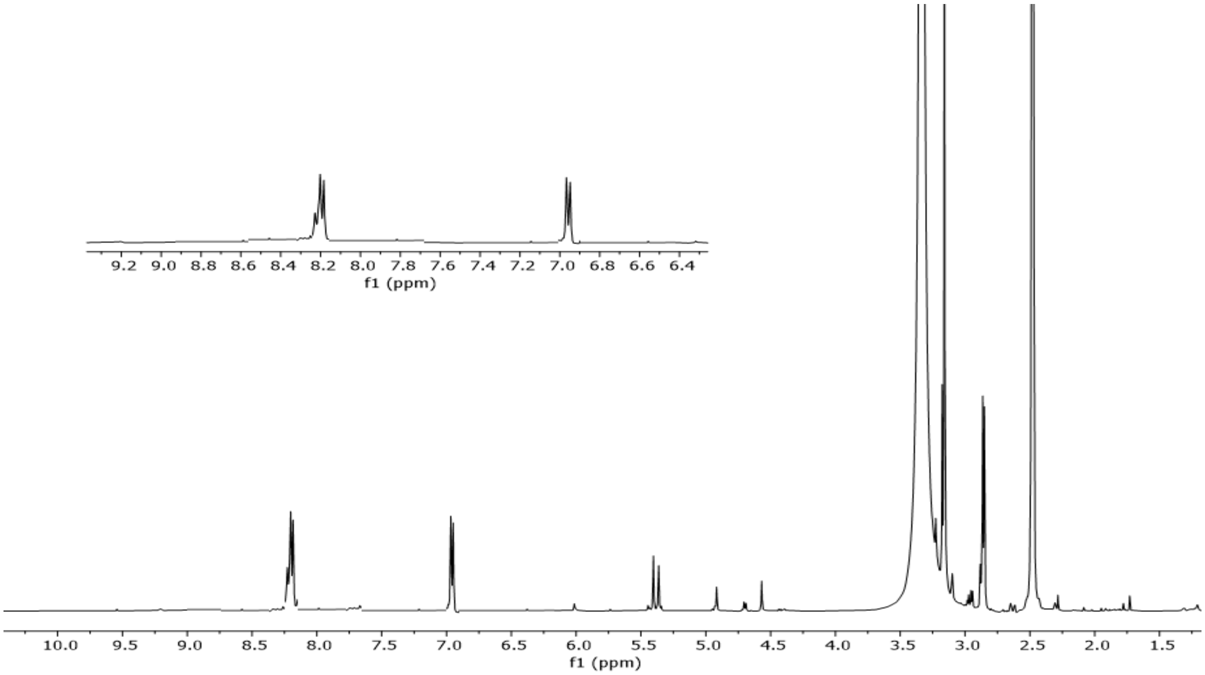


**Figure S32**. $^{1}H$ NMR spectrum of complex **4A** in DMSO-$d^6$.

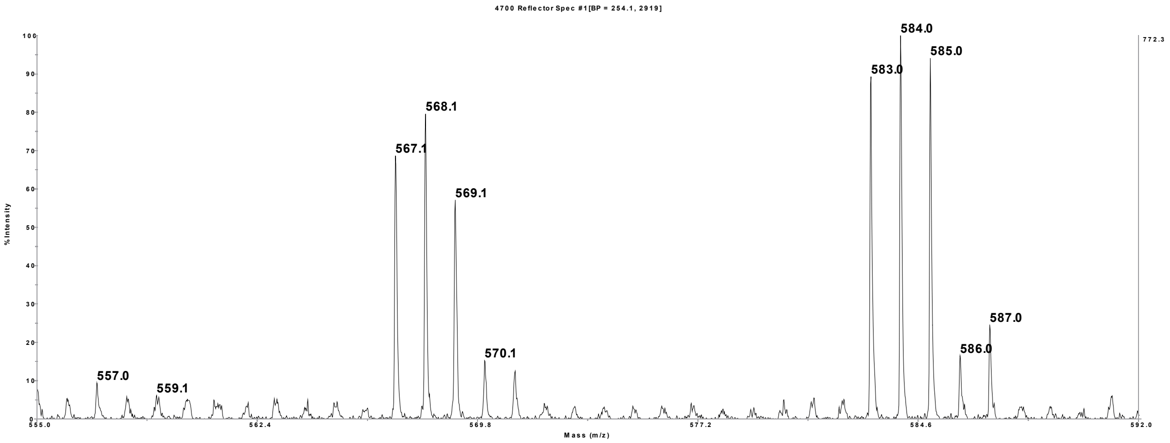


**Figure S33**. MALDI-TOF(+) of complex **4A**.

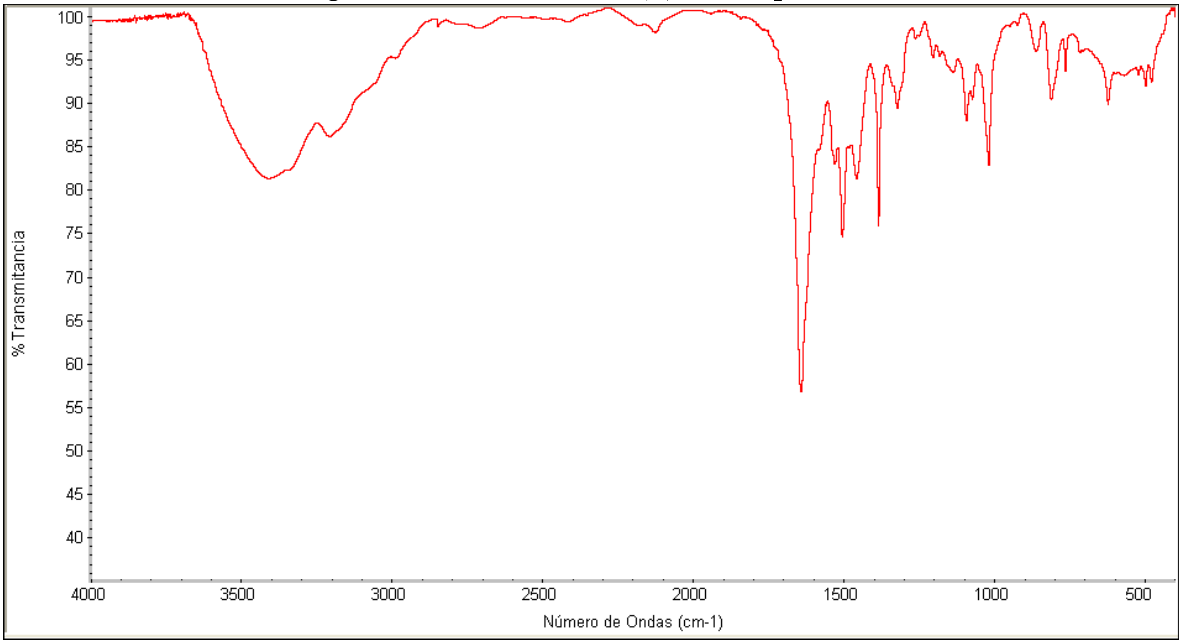


**Figure S34**. ATR IR spectrum of complex **4B** in KBr pellets.

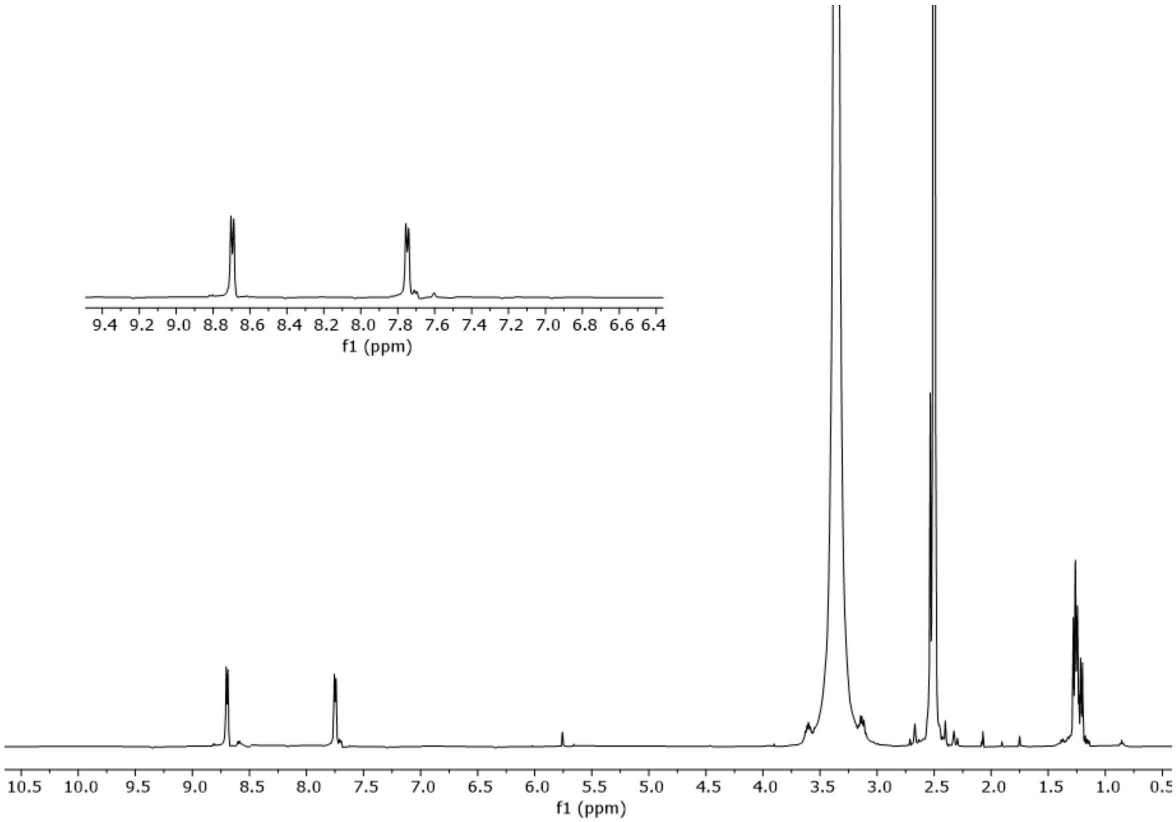


**Figure S35**. $^{1}H$ NMR spectrum of complex **4B** in DMSO-d$^{6}$.

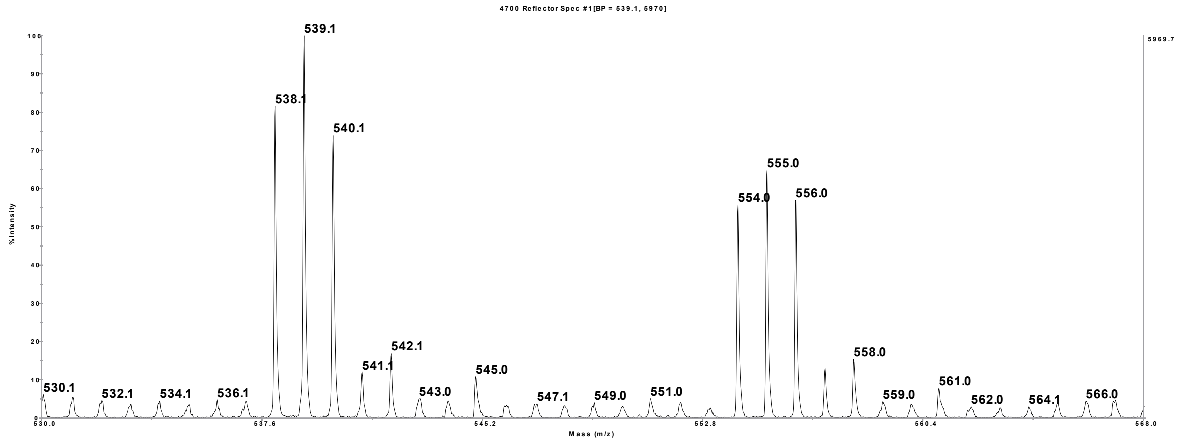


**Figure S36**. MALDI-TOF(+) of complex **4B**.

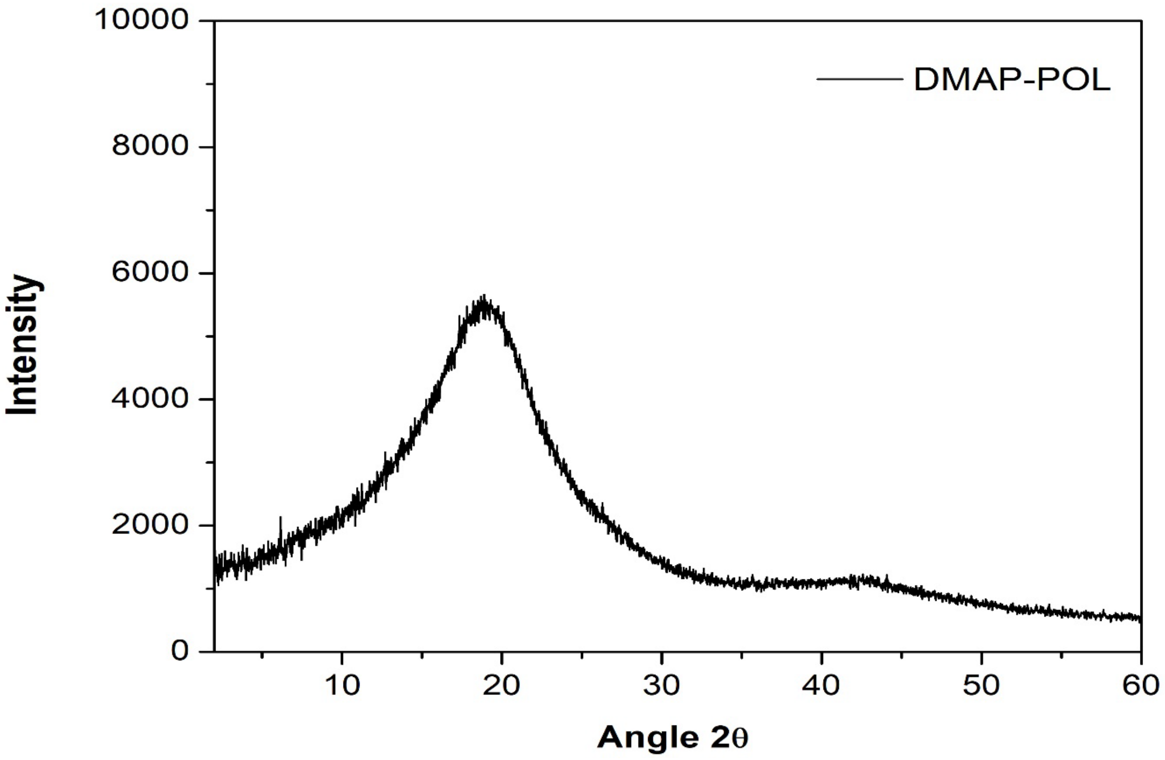


**Figure S37**. Powder X-ray diffraction pattern of 4-(dimethylamine)pyridine polymer bounded.

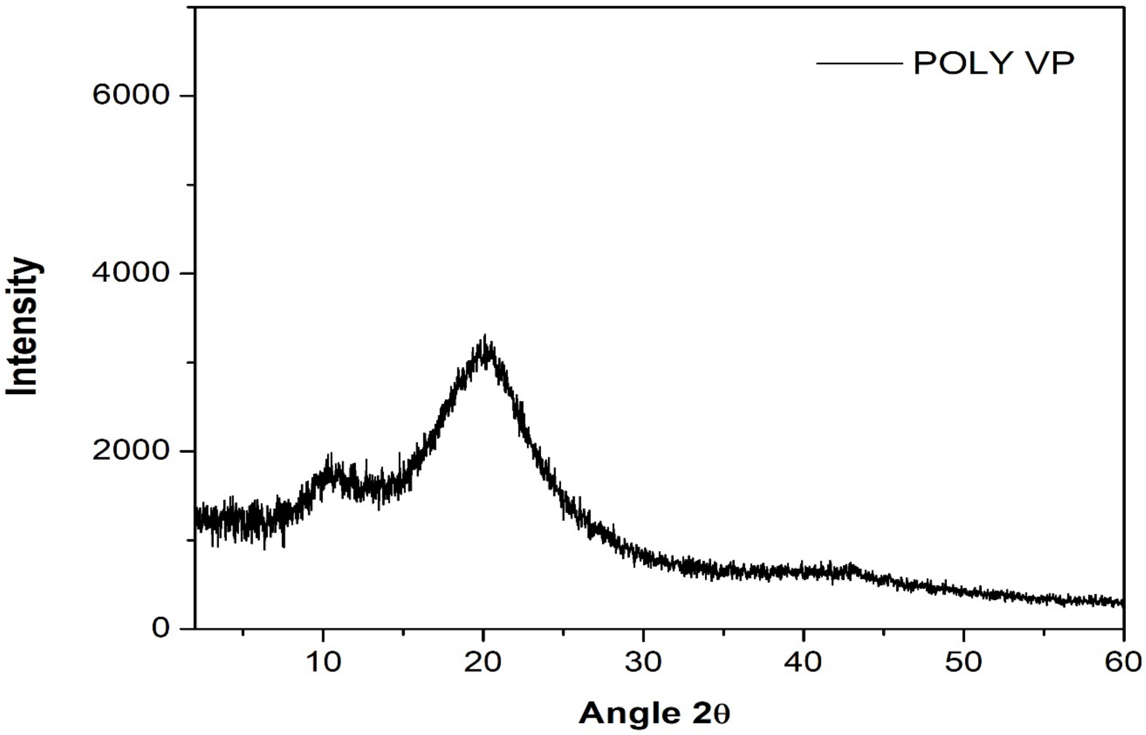


**Figure S38**. Powder X-ray diffraction pattern of Poly (4-vinyl pyridine).

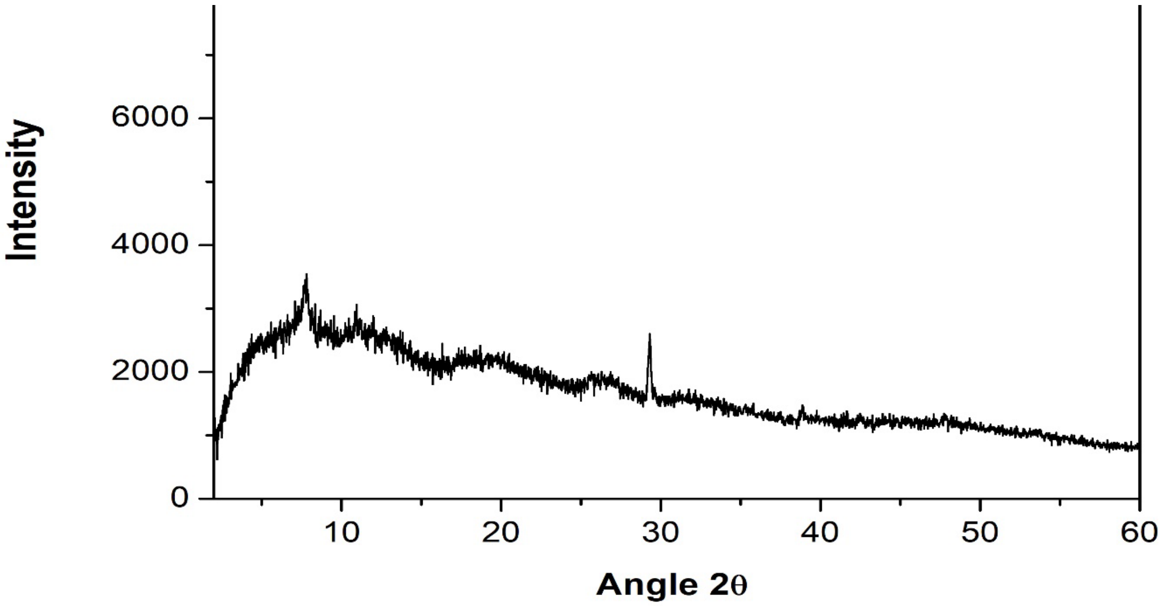


**Figure S39**. Powder X-ray diffraction pattern of polymer **1-DMAP**.

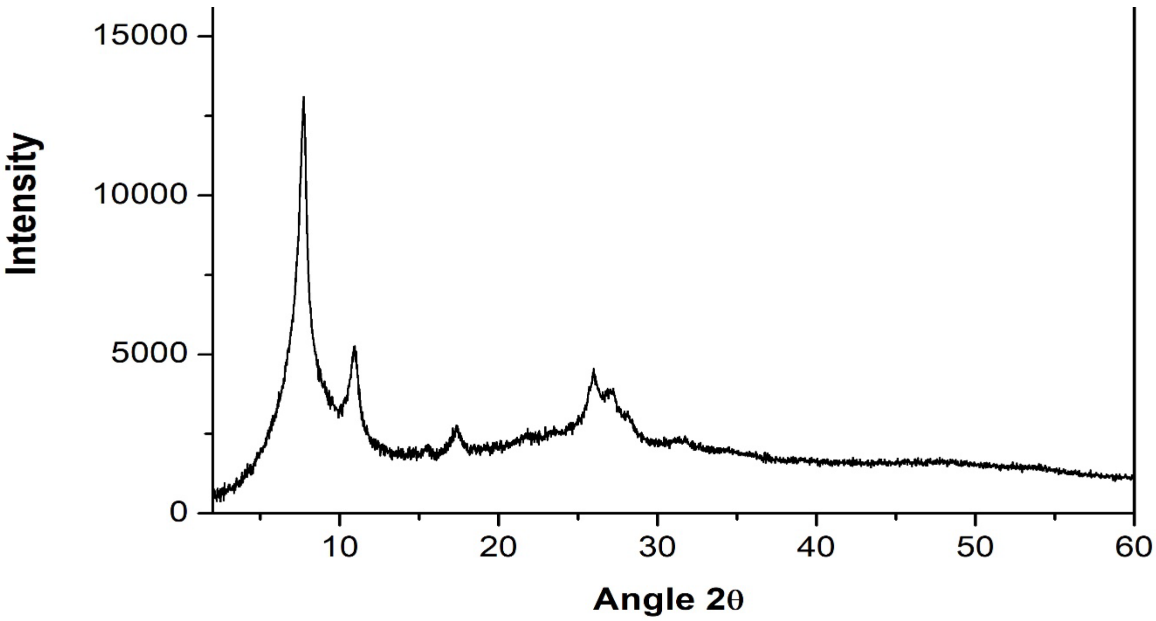


**Figure S40**. Powder X-ray diffraction pattern of polymer **1-PV**.

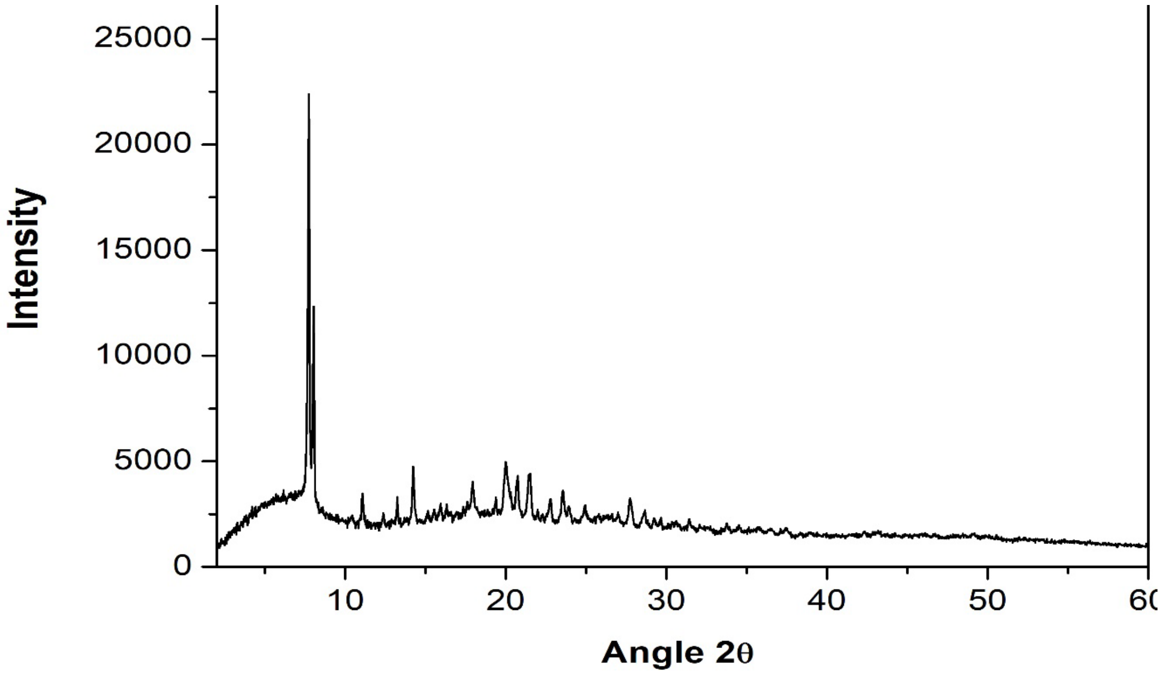


**Figure S41**. Powder X-ray diffraction pattern of polymer **2-DMAP**.

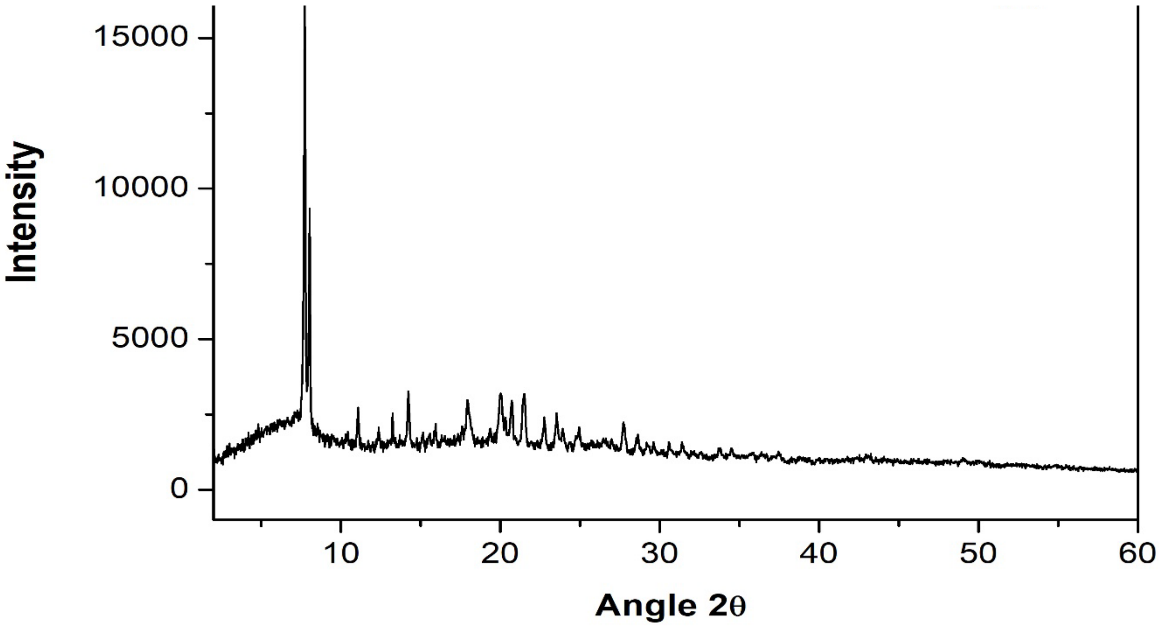


**Figure S42**. Powder X-ray diffraction pattern of polymer **2-PV**.

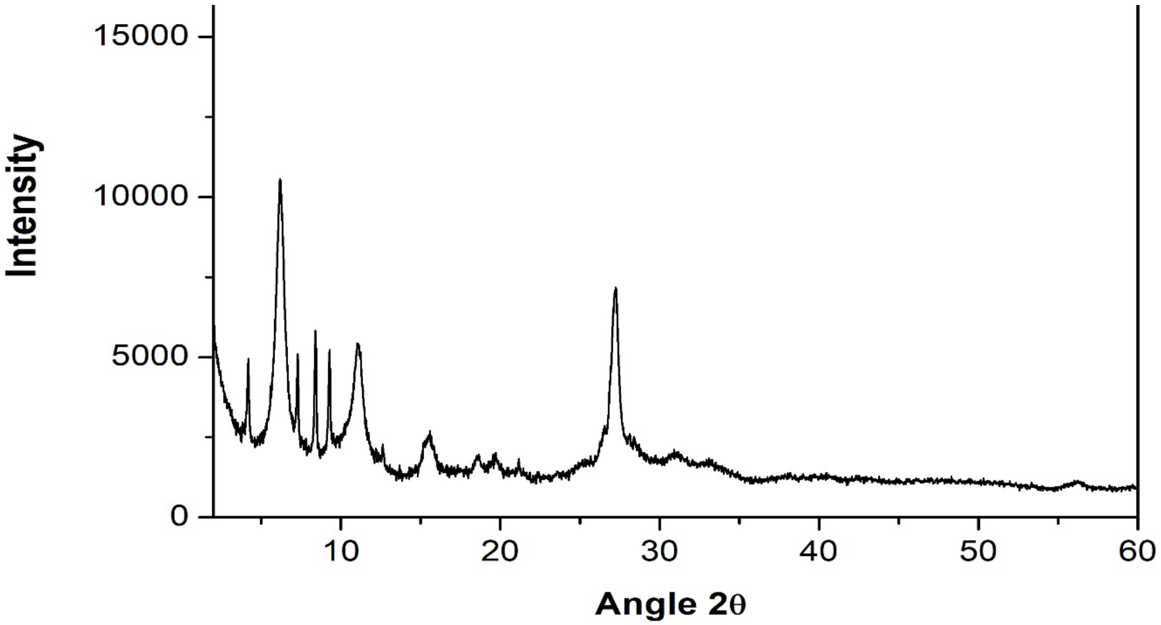


**Figure S43**. Powder X-ray diffraction pattern of polymer **3-DMAP**.

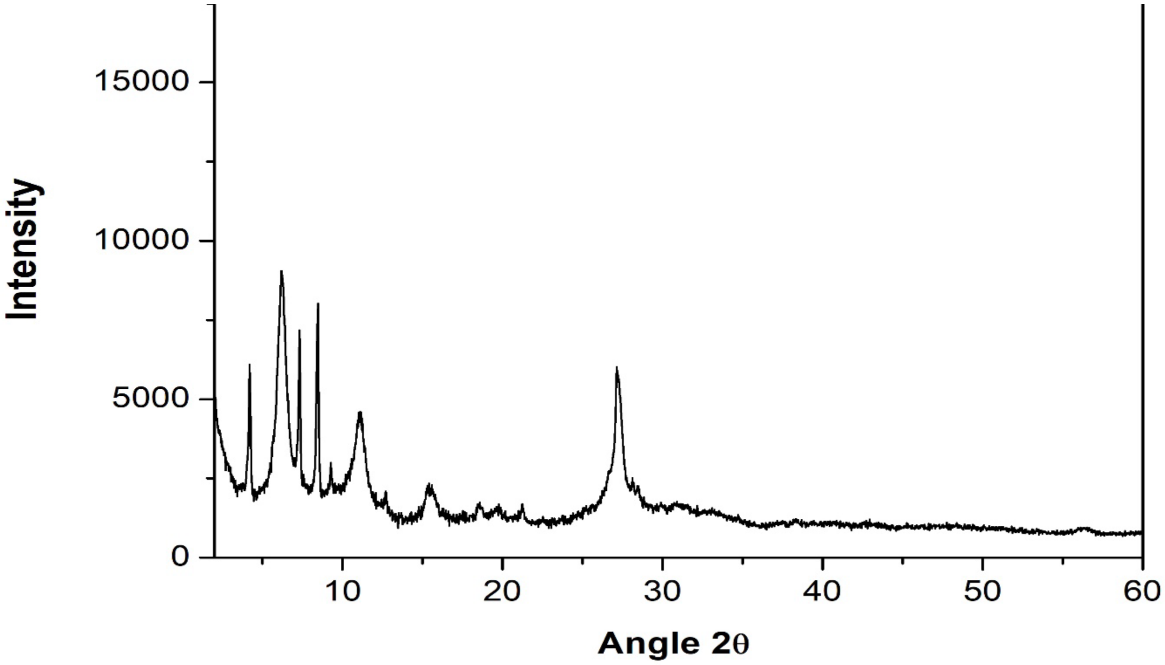


**Figure S44**. Powder X-ray diffraction pattern of polymer **3-PV**.

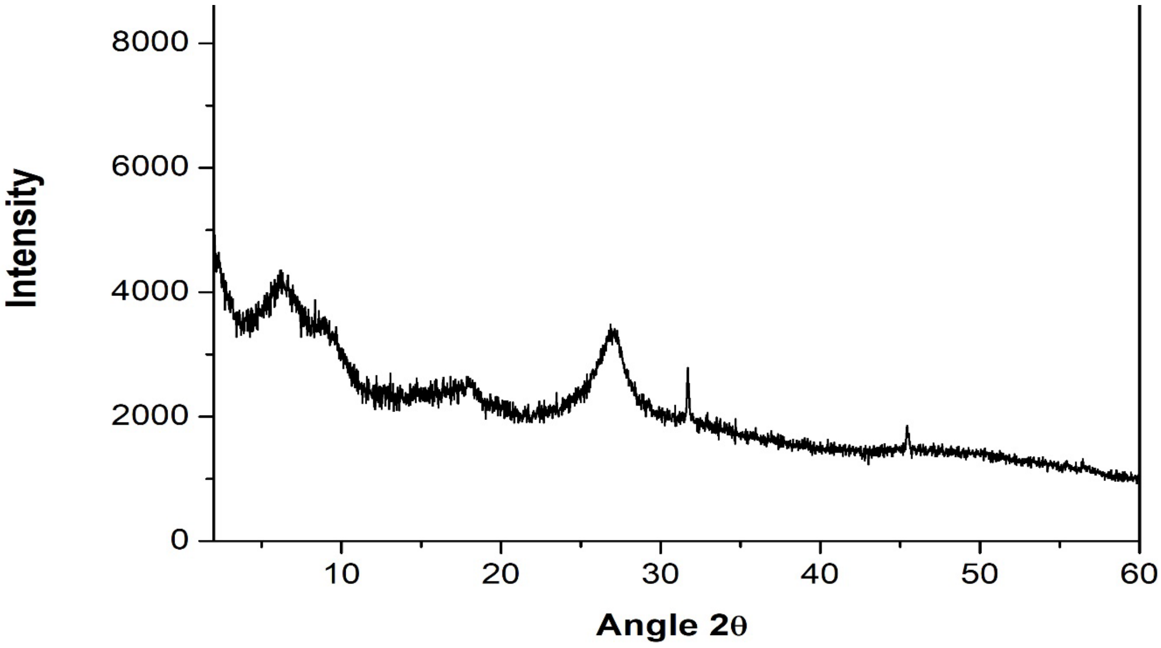


**Figure S45**. Powder X-ray diffraction pattern of polymer **4-DMAP**.

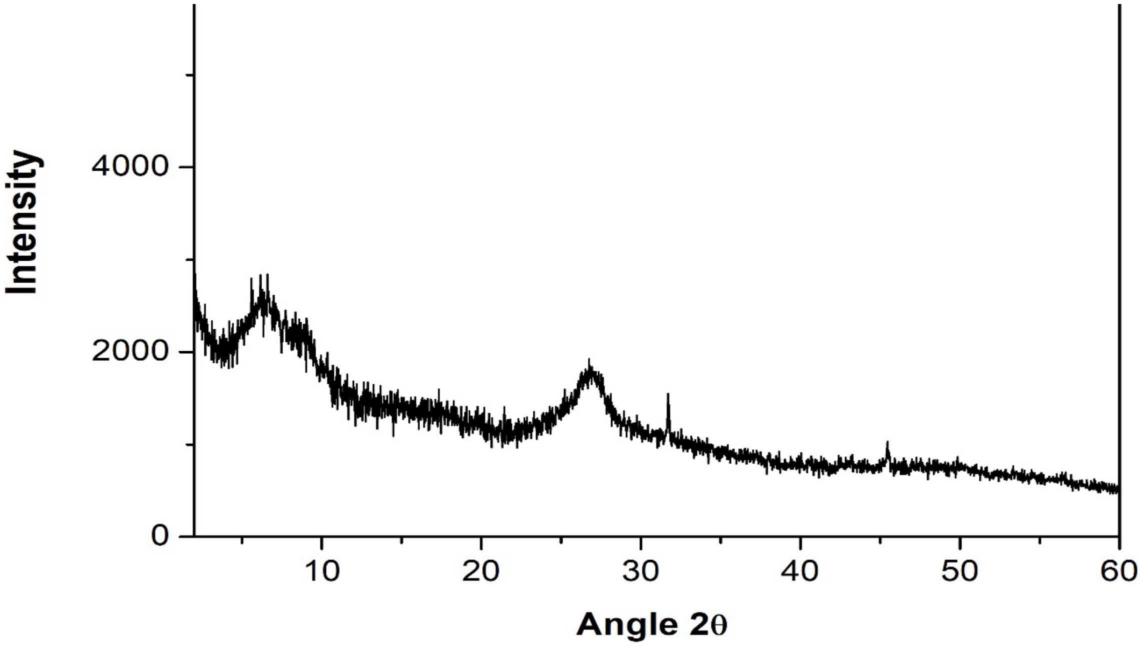


**Figure S46**. Powder X-ray diffraction pattern of polymer **4-PV**.

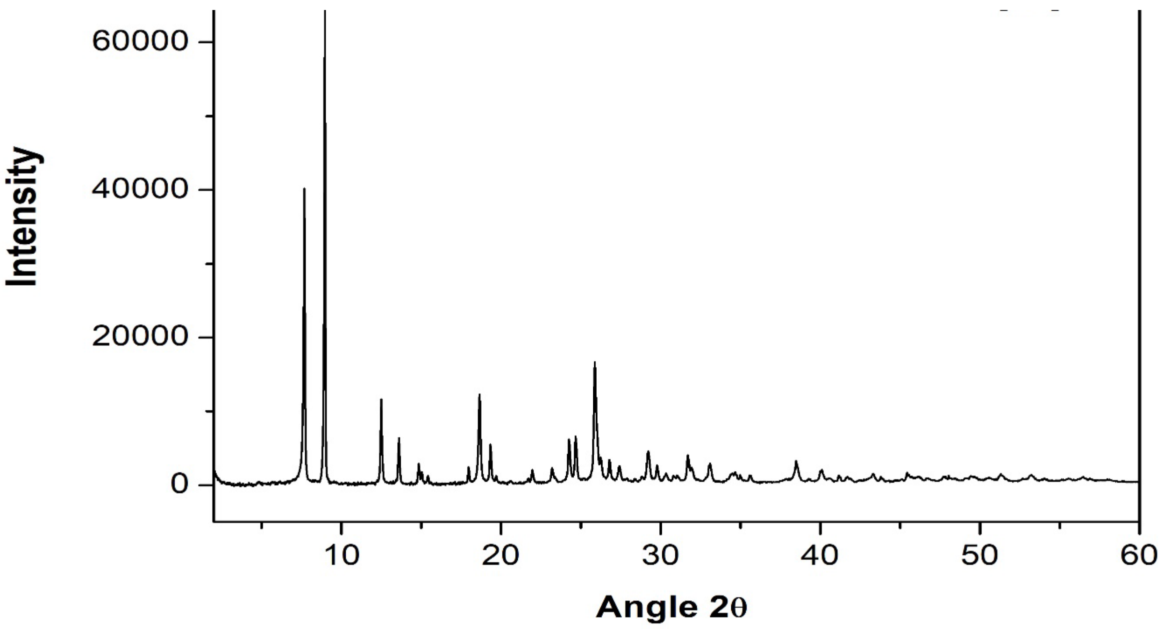


**Figure S47**. Powder X-ray diffraction pattern of compound **1A**.

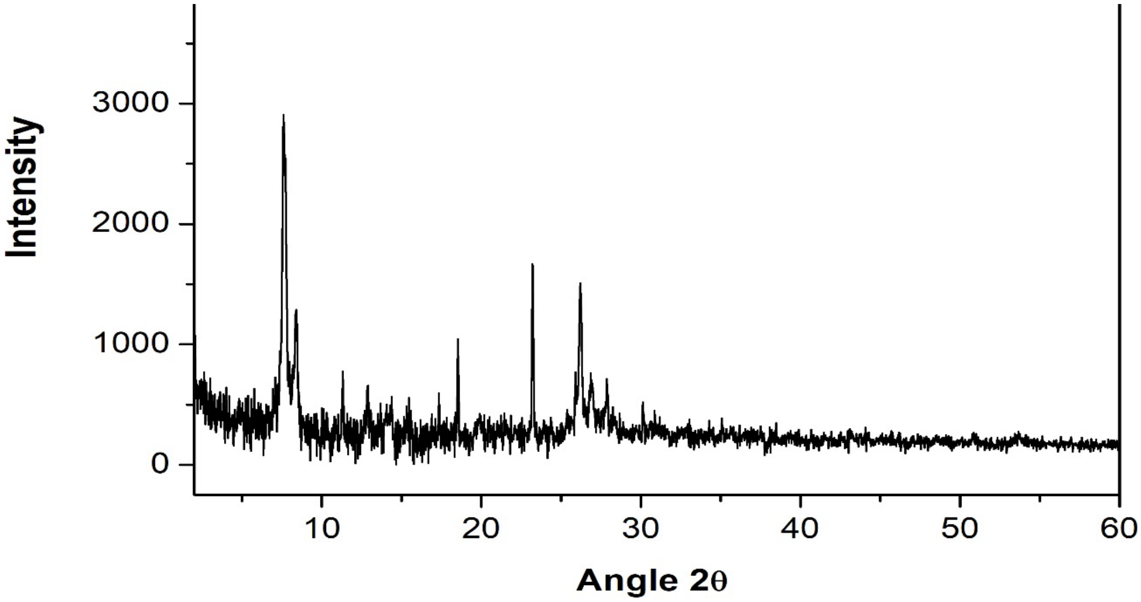


**Figure S48**. Powder X-ray diffraction pattern of compound **1B**.

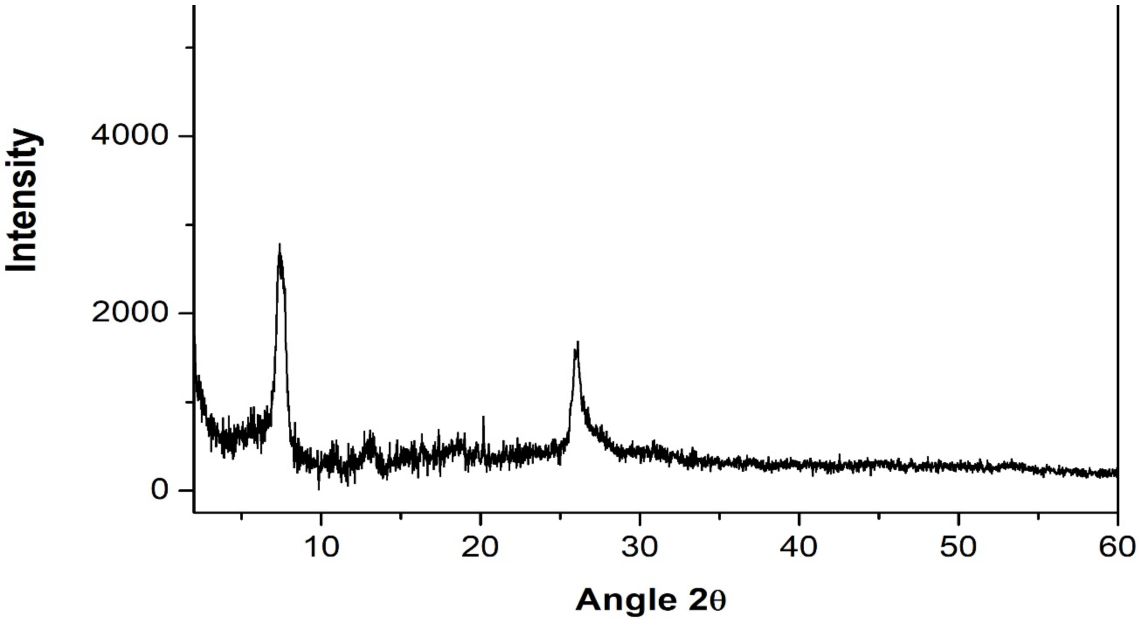


**Figure S49**. Powder X-ray diffraction pattern of compound **2A**.

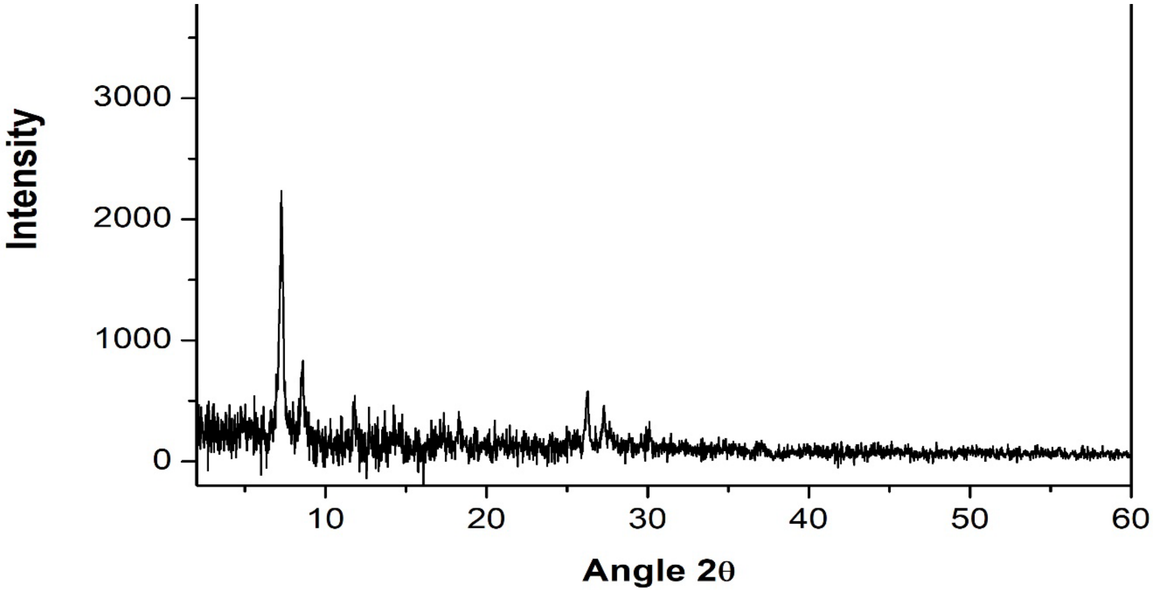


**Figure S50**. Powder X-ray diffraction pattern of compound **2B**.

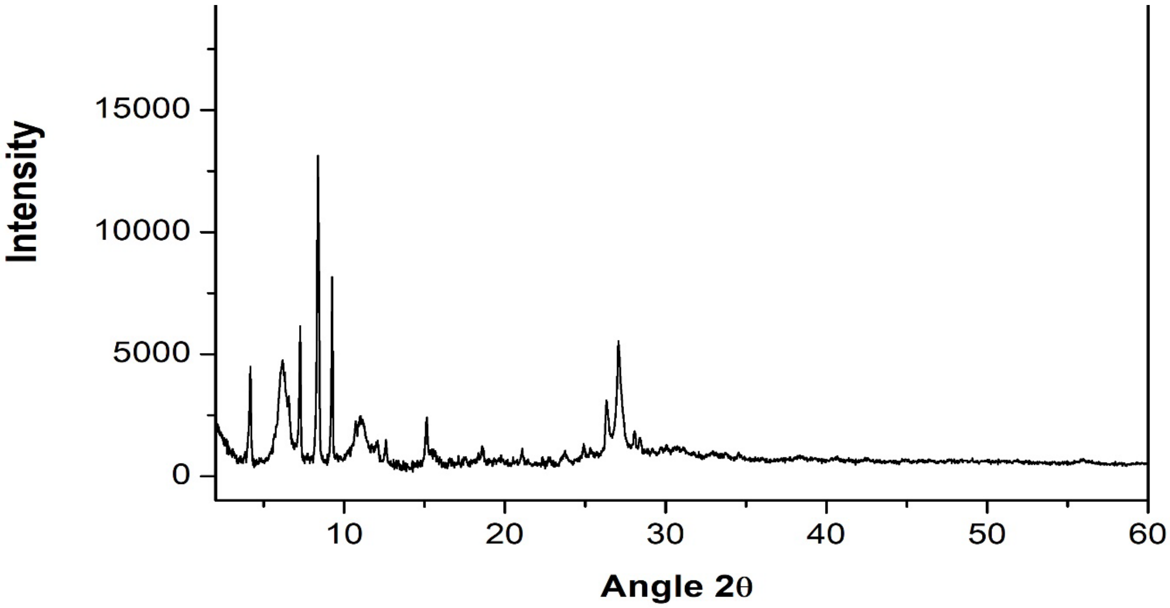


**Figure S51**. Powder X-ray diffraction pattern of compound **3A**.

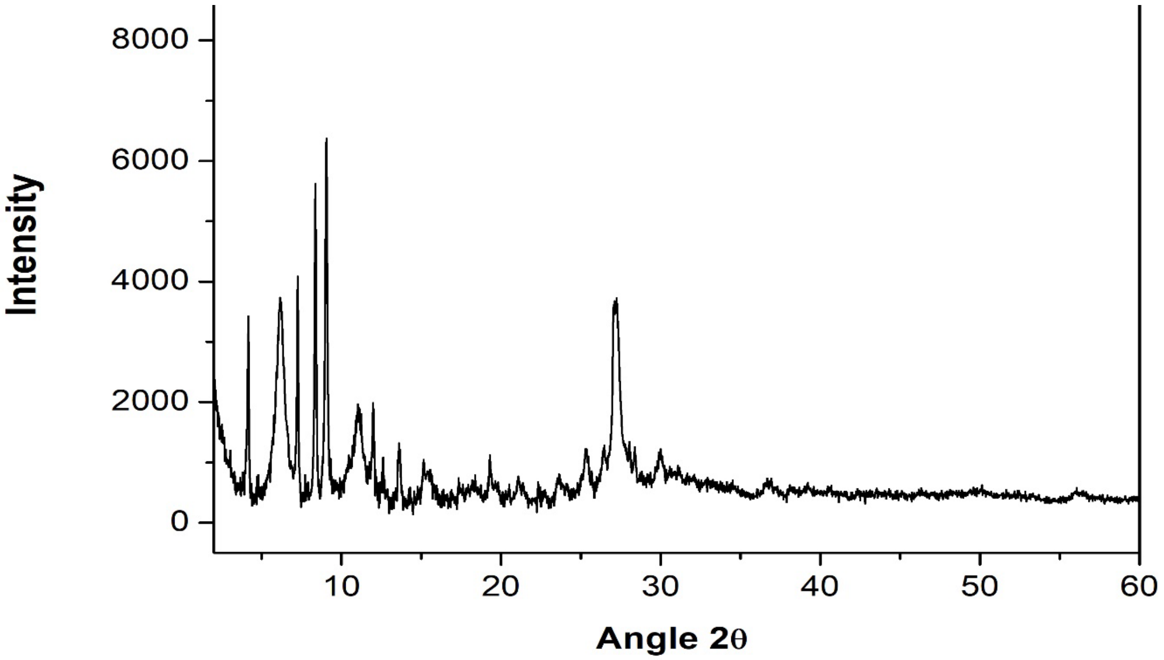


**Figure S52**. Powder X-ray diffraction pattern of compound **3B**.

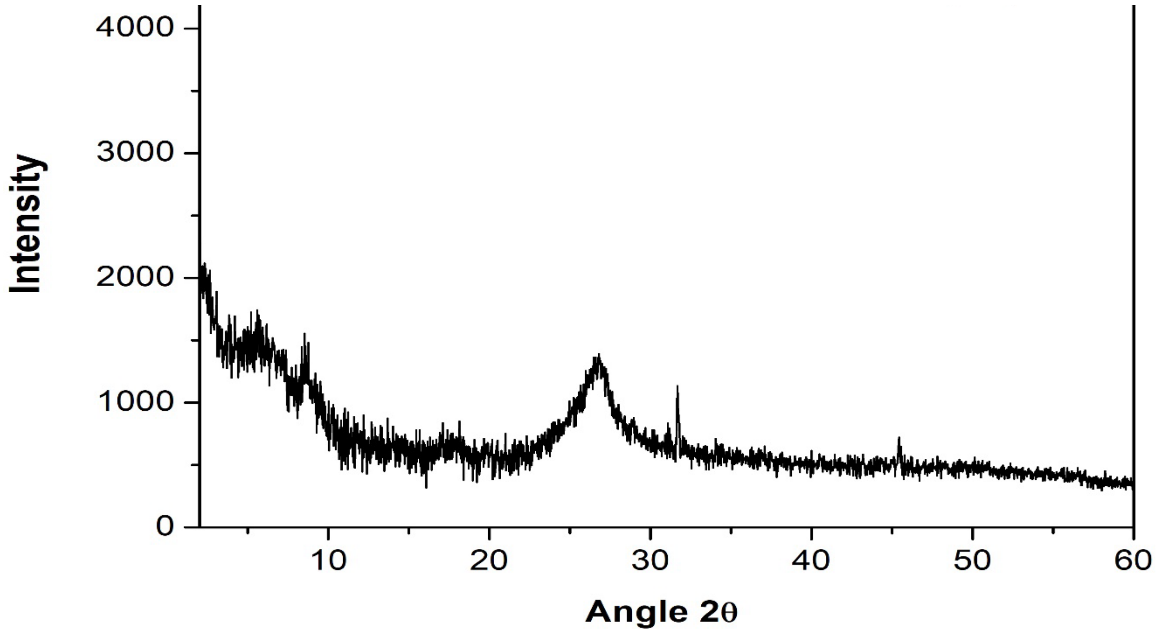


**Figure S53**. Powder X-ray diffraction pattern of compound **4A**.

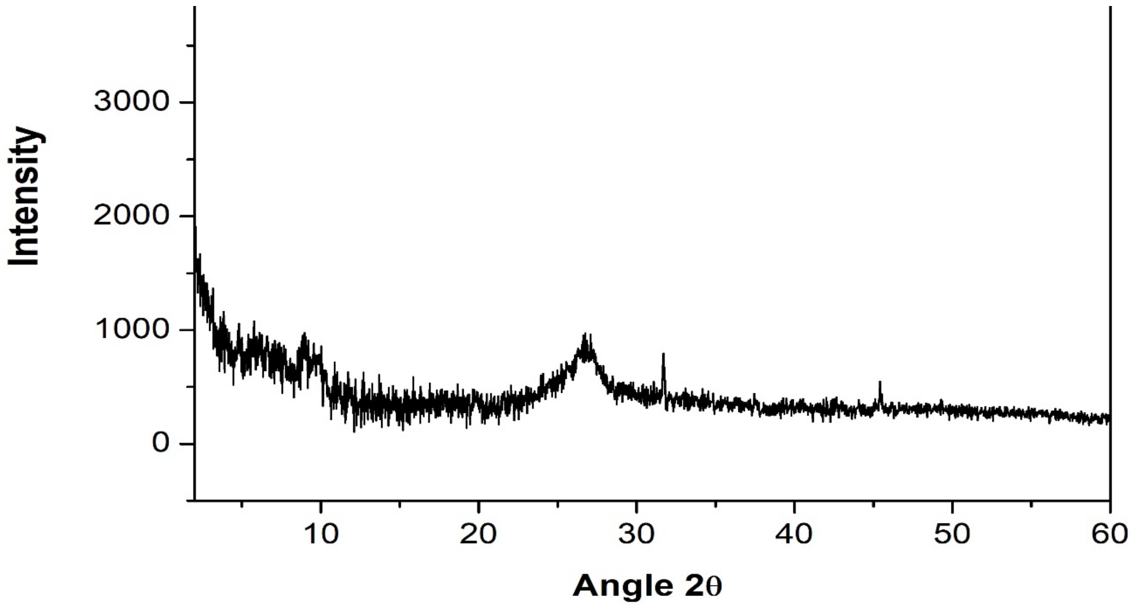


**Figure S54**. Powder X-ray diffraction pattern of compound **4B**.

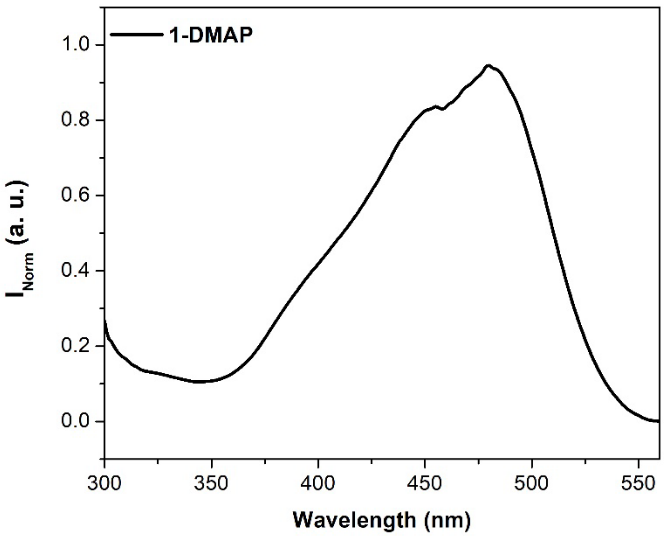


**Figure S55**. Normalized excitation spectra of **1-DMAP** ($\lambda_{em}$ = 600 nm) in the solid state.

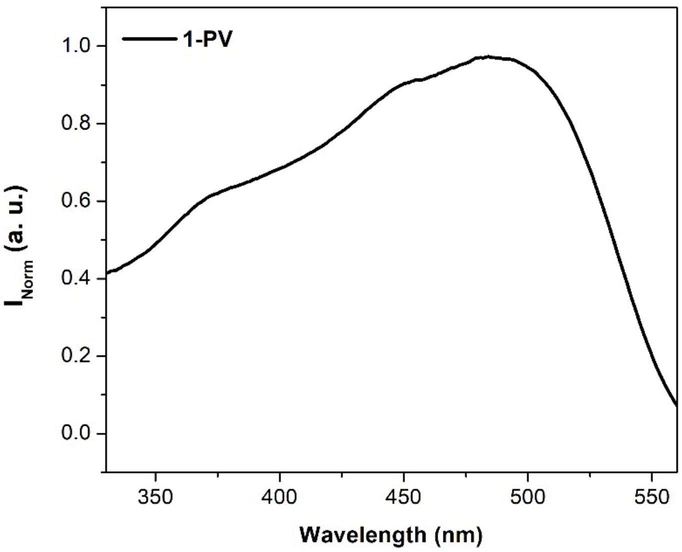


**Figure S56**. Normalized excitation spectra of **1-PV** ($\lambda_{em}$ = 600 nm) in the solid state.

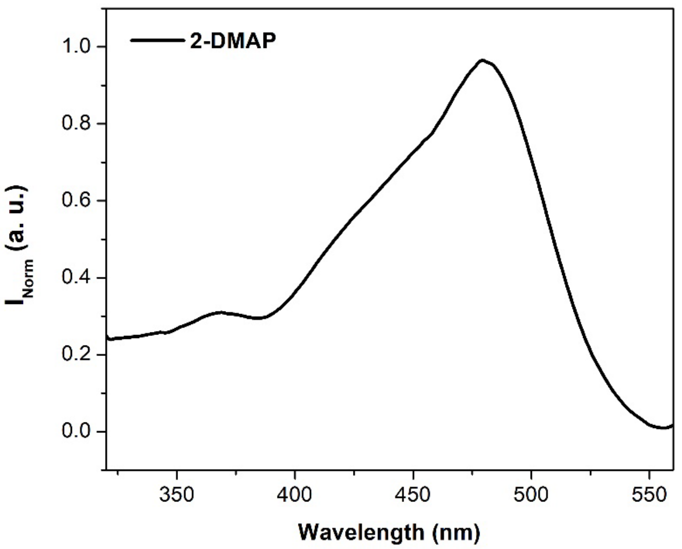


**Figure S57**. Normalized excitation spectra of **2-DMAP** ($\lambda_{em}$ = 600 nm) in the solid state.

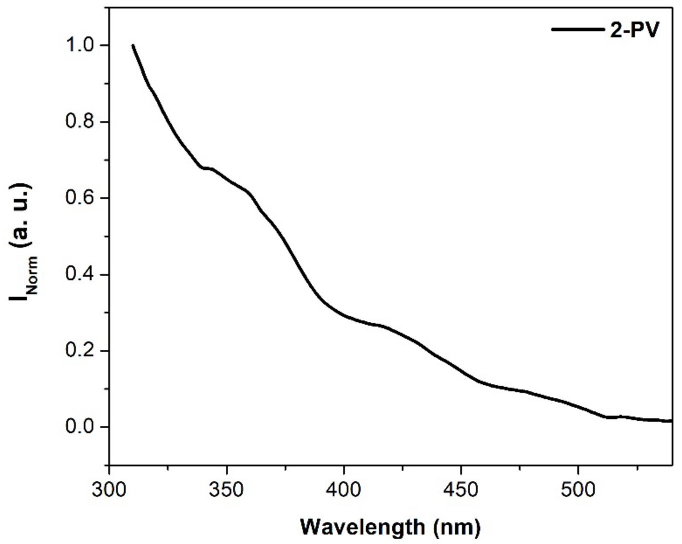


**Figure S58**. Normalized excitation spectra of **2-PV** ($\lambda_{em}$ = 550 nm) in the solid state.

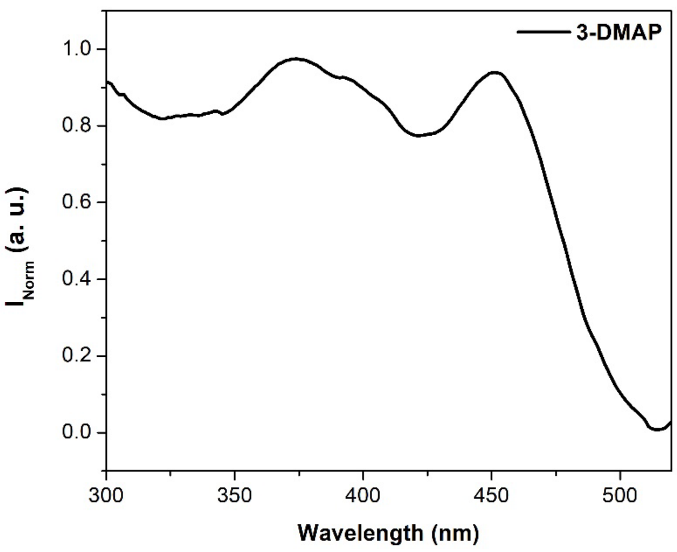


**Figure S59**. Normalized excitation spectra of **3-DMAP** ($\lambda_{em}$ = 550 nm) in the solid state.

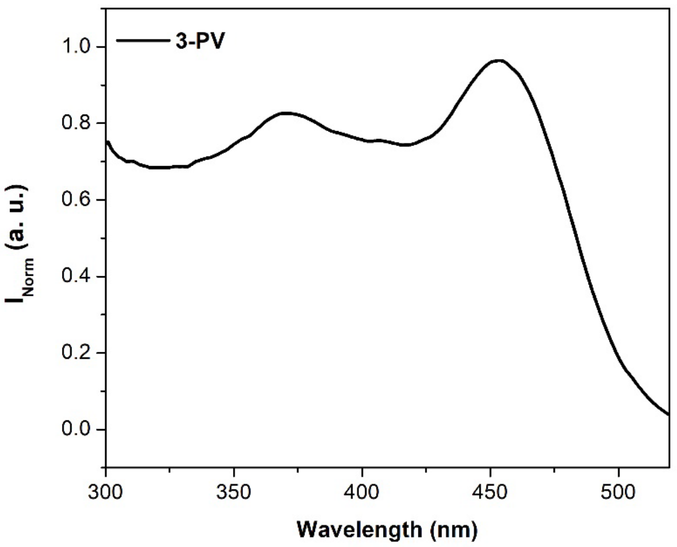


**Figure S60**. Normalized excitation spectra of **3-PV** ($\lambda_{em}$ = 550 nm) in the solid state.

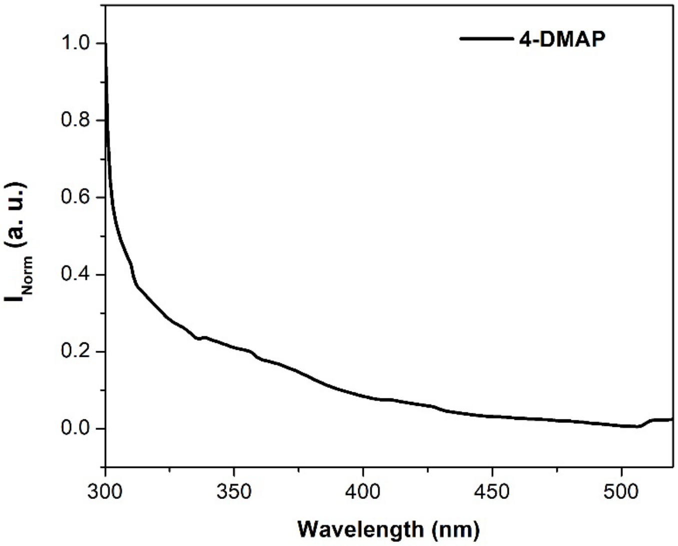


**Figure S61**. Normalized excitation spectra of **4-DMAP** ($\lambda_{em}$ = 550 nm) in the solid state.

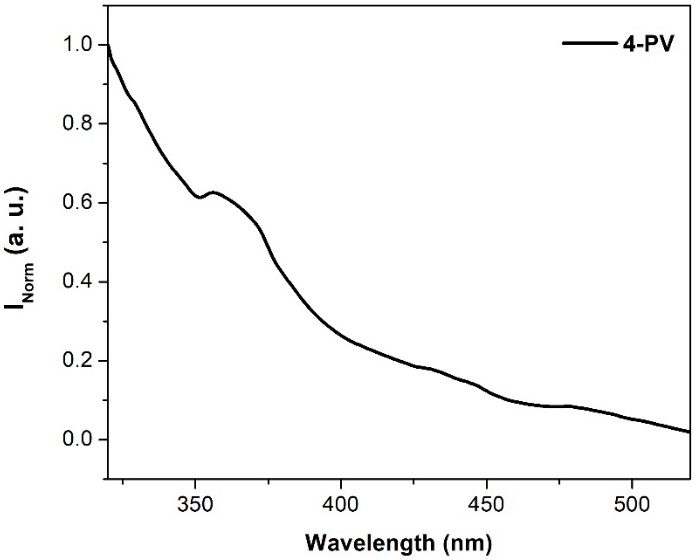


**Figure S62**. Normalized excitation spectra of **4-PV** ($\lambda_{em}$ = 550 nm) in the solid state.

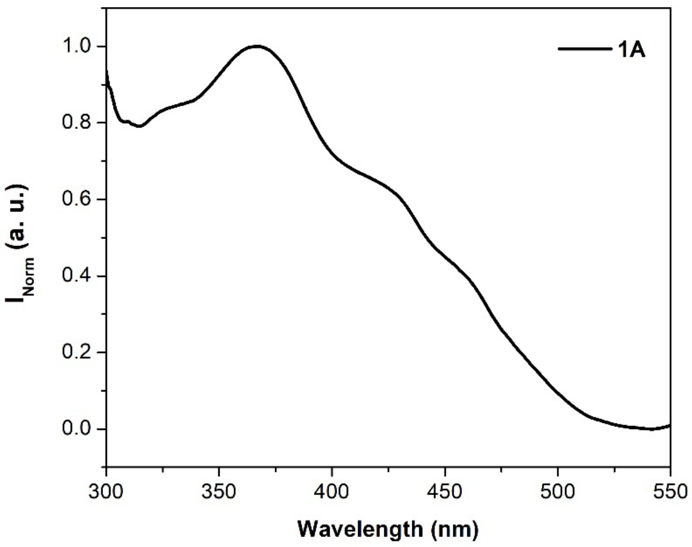


**Figure S63**. Normalized excitation spectra of **1A** ($\lambda_{em}$ = 550 nm) in the solid state.

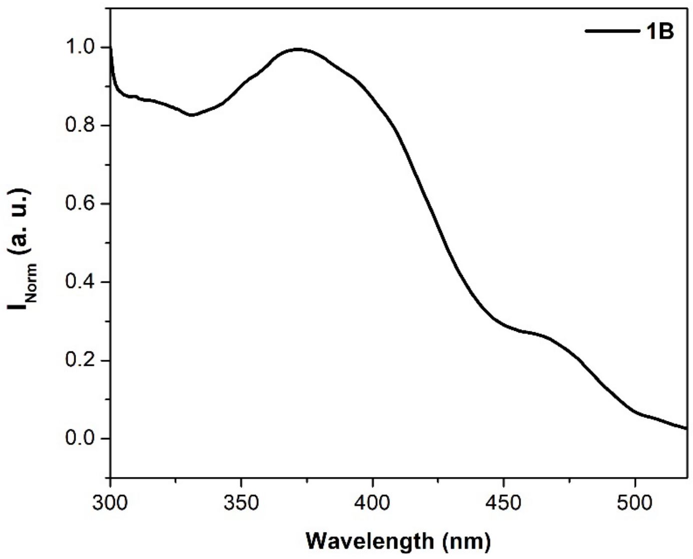


**Figure S64**. Normalized excitation spectra of **1B** ($\lambda_{em}$ = 550 nm) in the solid state.

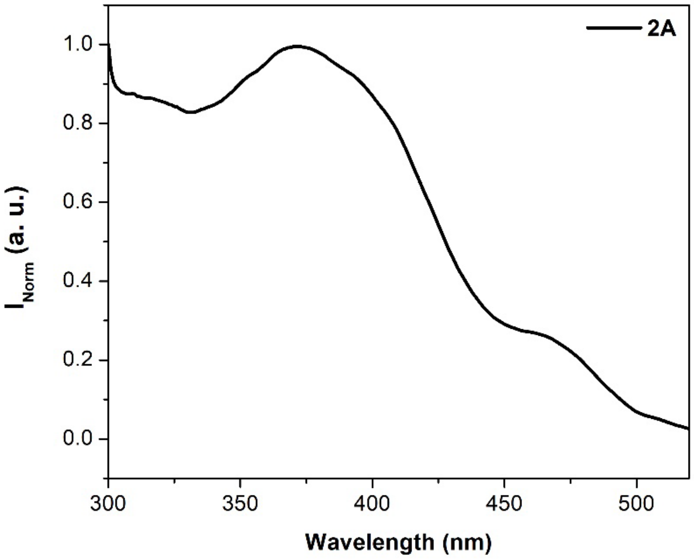


**Figure S65**. Normalized excitation spectra of **2A** ($\lambda_{em}$ = 550 nm) in the solid state.

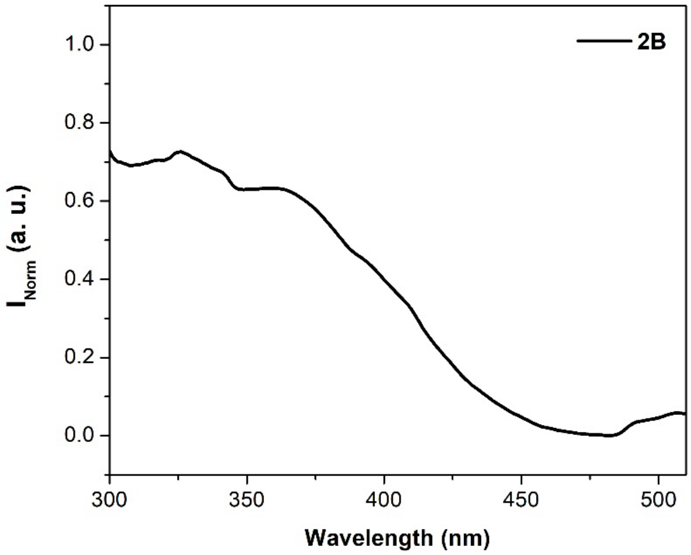


**Figure S66**. Normalized excitation spectra of **2B** ($\lambda_{em}$ = 550 nm) in the solid state.

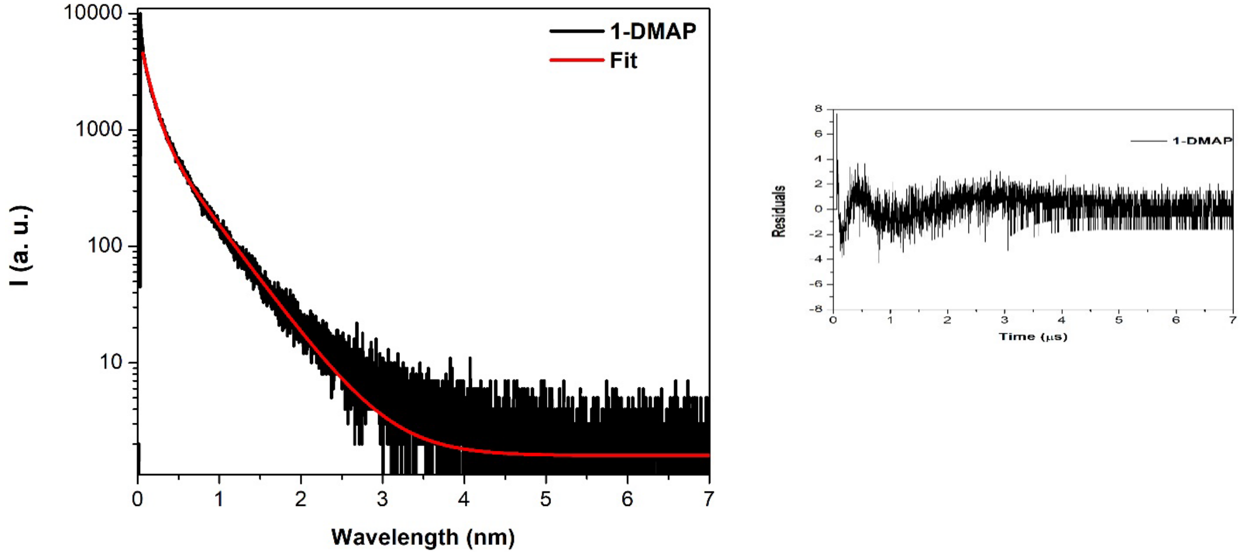


**Figure S67**. Lifetime decay and residual plots of complex **1-DMAP** recorded in the solid state.

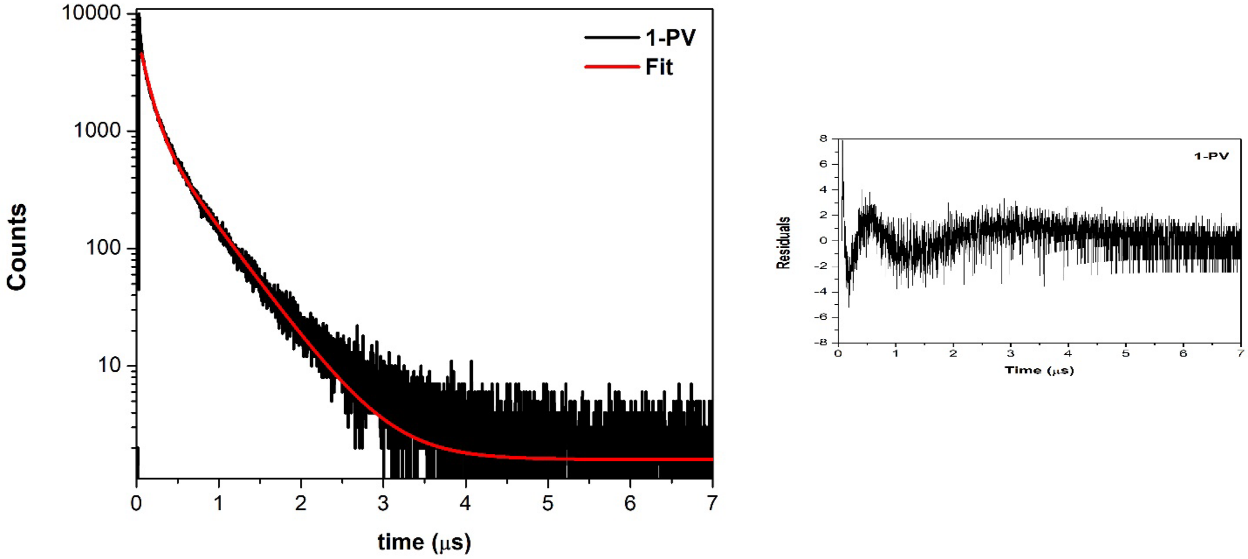


**Figure S68**. Lifetime decay and residual plots of complex **1-PV** recorded in the solid state.

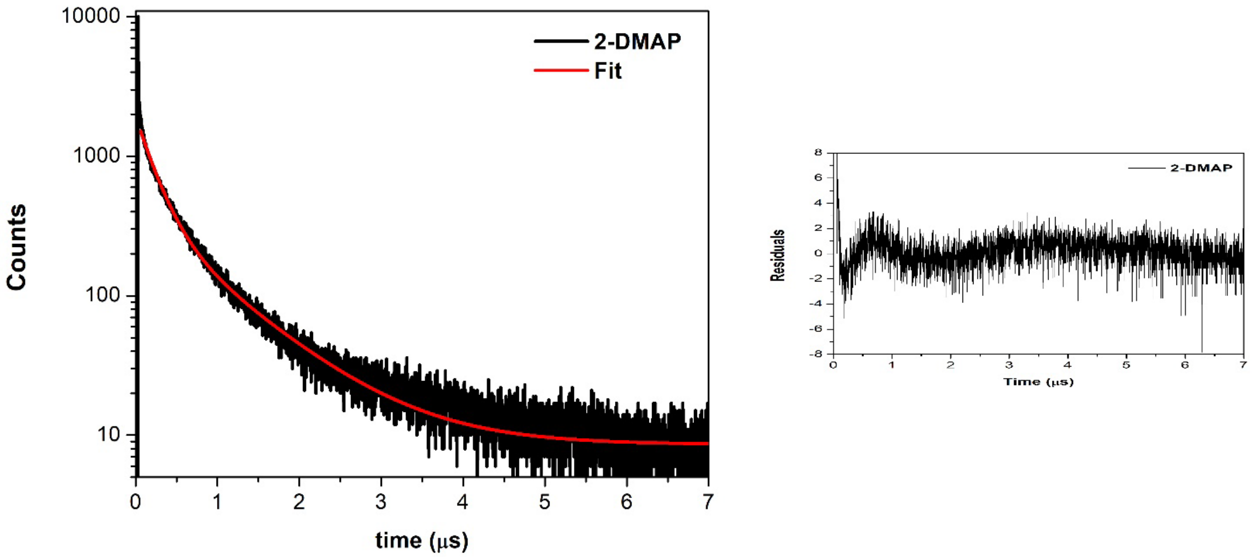


**Figure S69**. Lifetime decay and residual plots of complex **2-DMAP** recorded in the solid state.

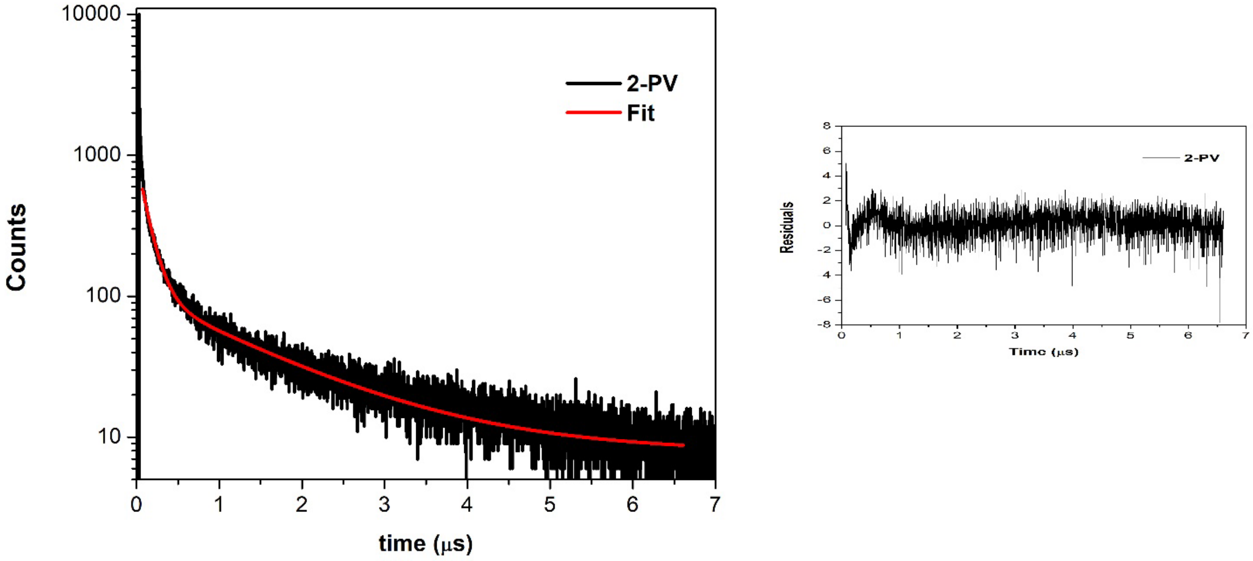


**Figure S70**. Lifetime decay and residual plots of complex **2-PV** recorded in the solid state.

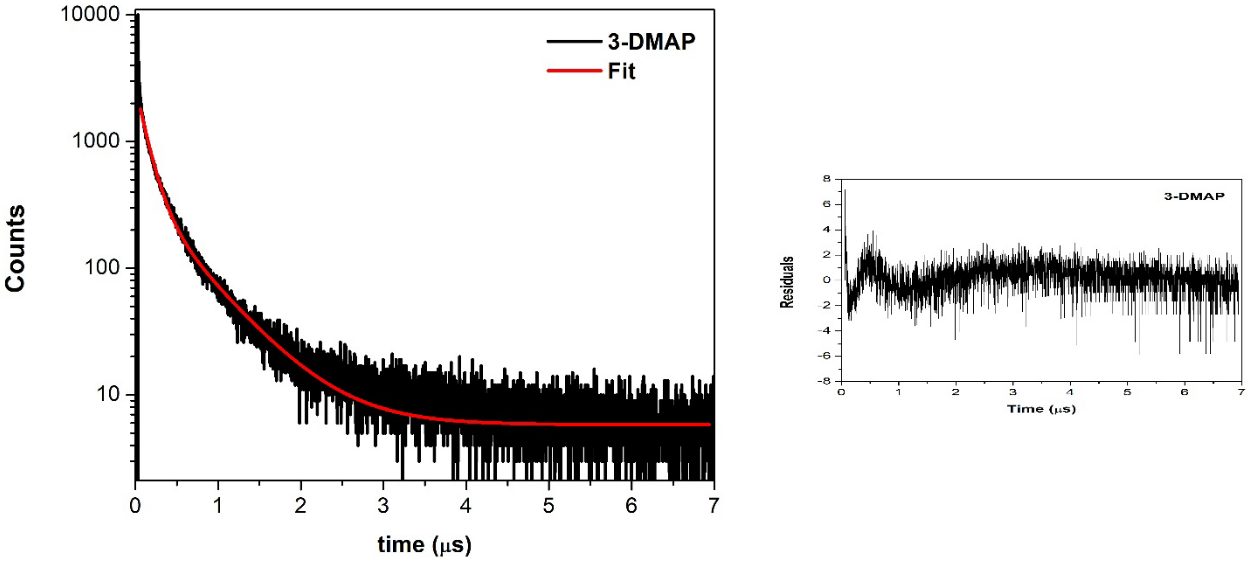


**Figure S71**. Lifetime decay and residual plots of complex **3-DMAP** recorded in the solid state.

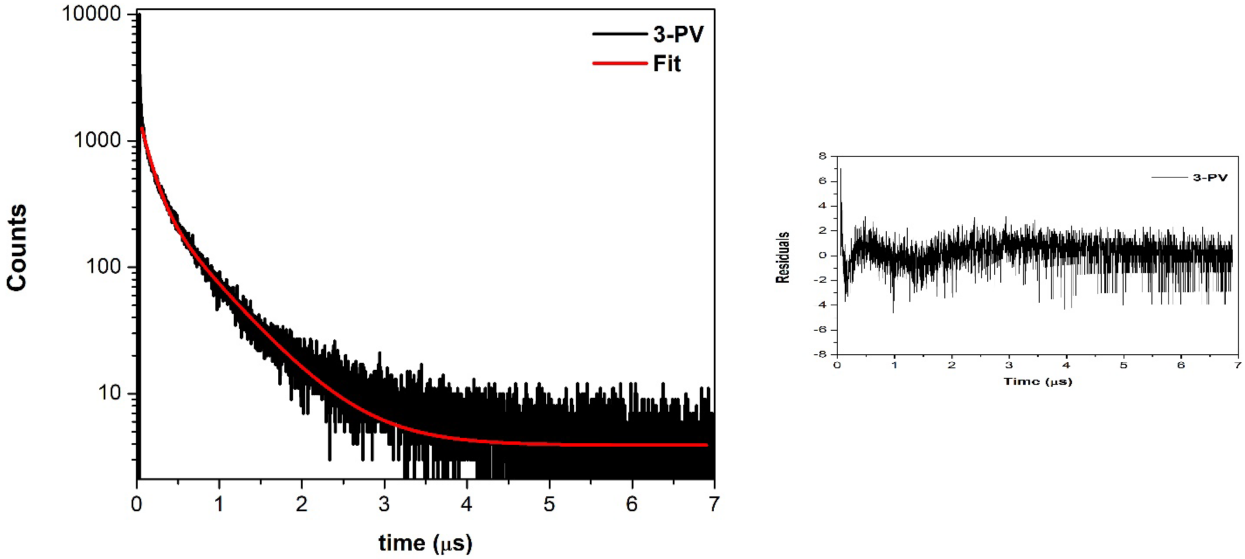


**Figure S72**. Lifetime decay and residual plots of complex **3-PV** recorded in the solid state.

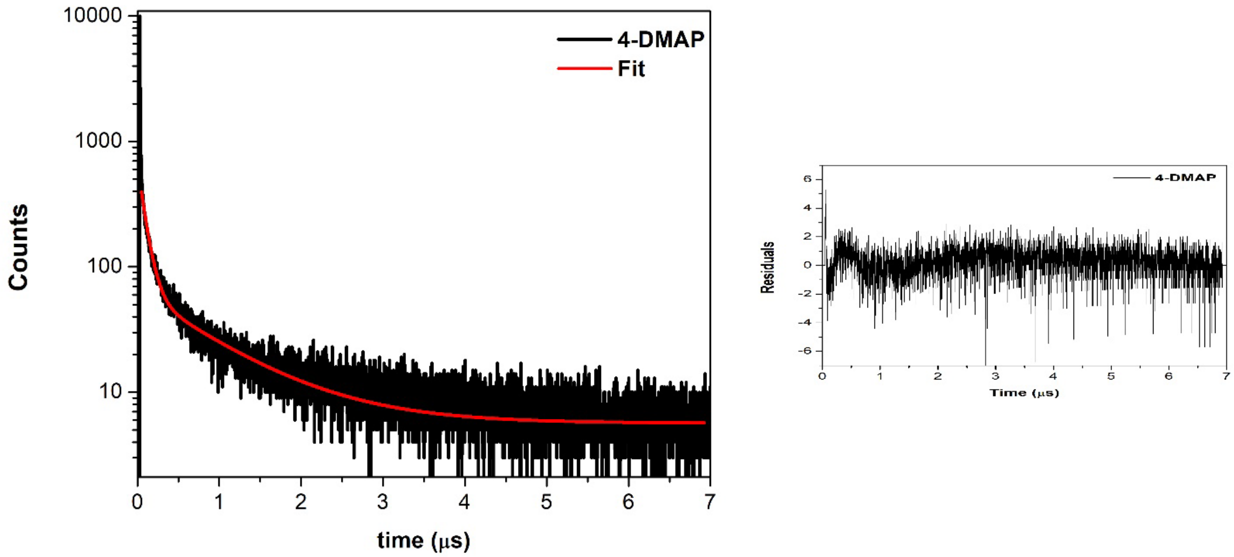


**Figure S73**. Lifetime decay and residual plots of complex **4-DMAP** recorded in the solid state.

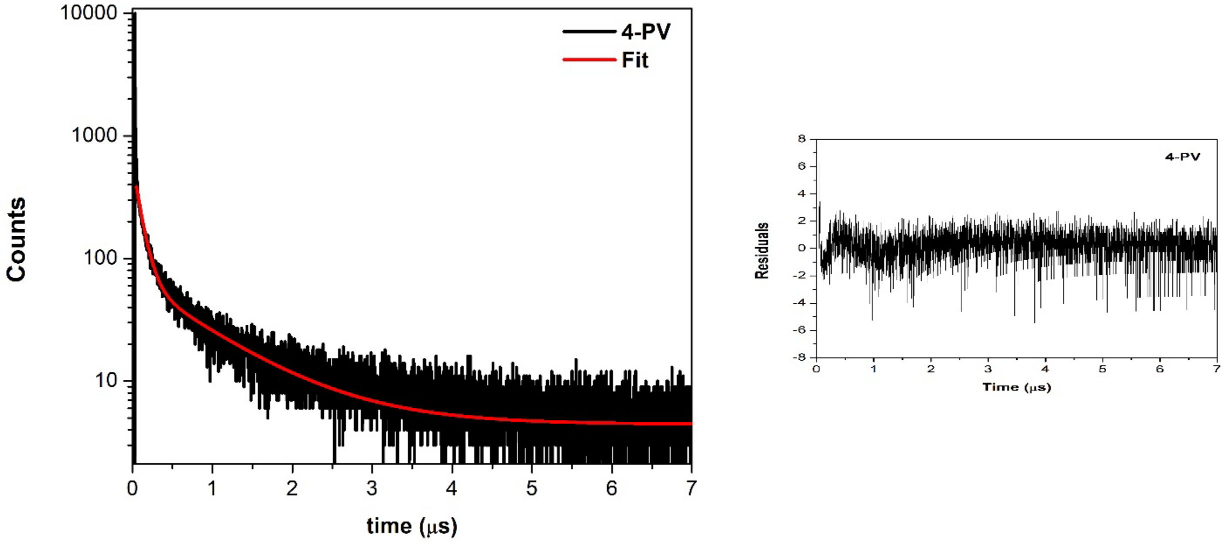


**Figure S74**. Lifetime decay and residual plots of complex **4-PV** recorded in the solid state.

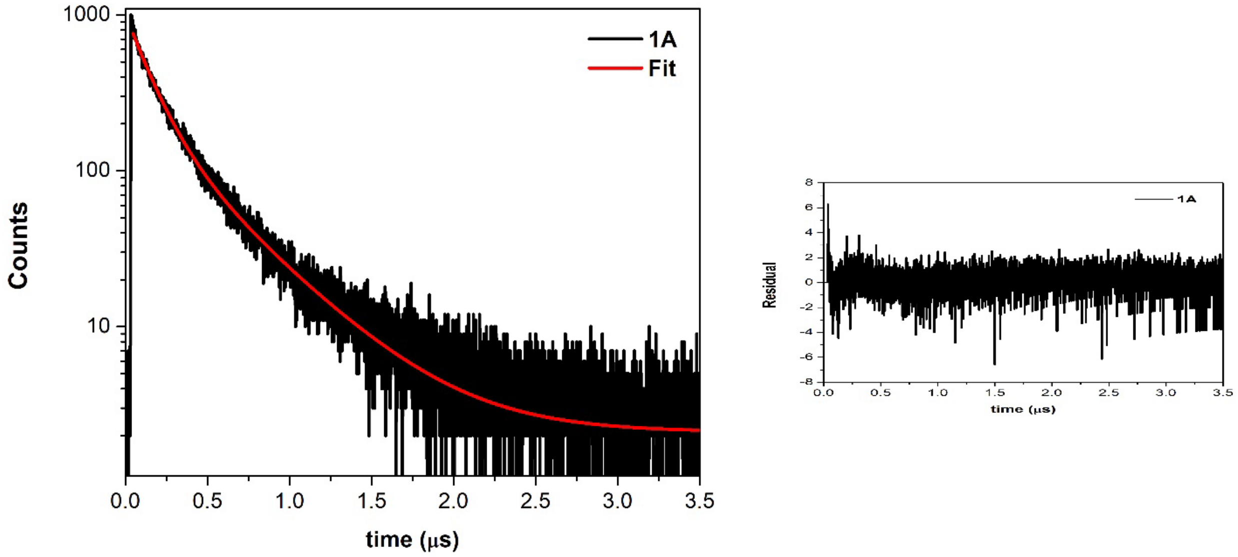


**Figure S75**. Lifetime decay and residual plots of complex **1A** recorded in the solid state.

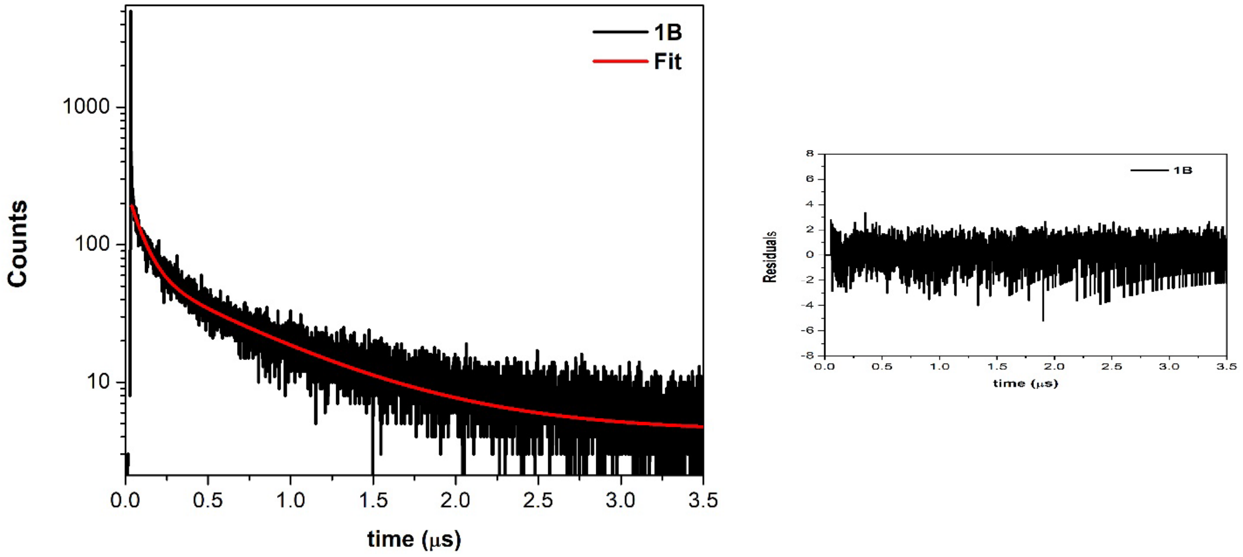


**Figure S76**. Lifetime decay and residual plots of complex **1B** recorded in the solid state.

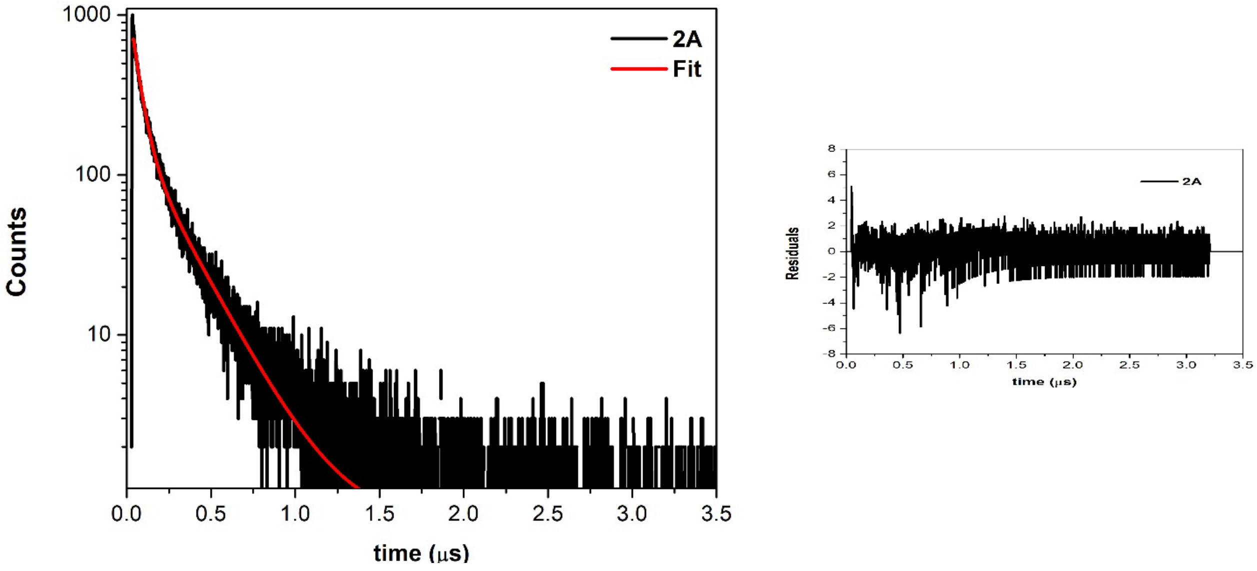


**Figure S77**. Lifetime decay and residual plots of complex **2A** recorded in the solid state.

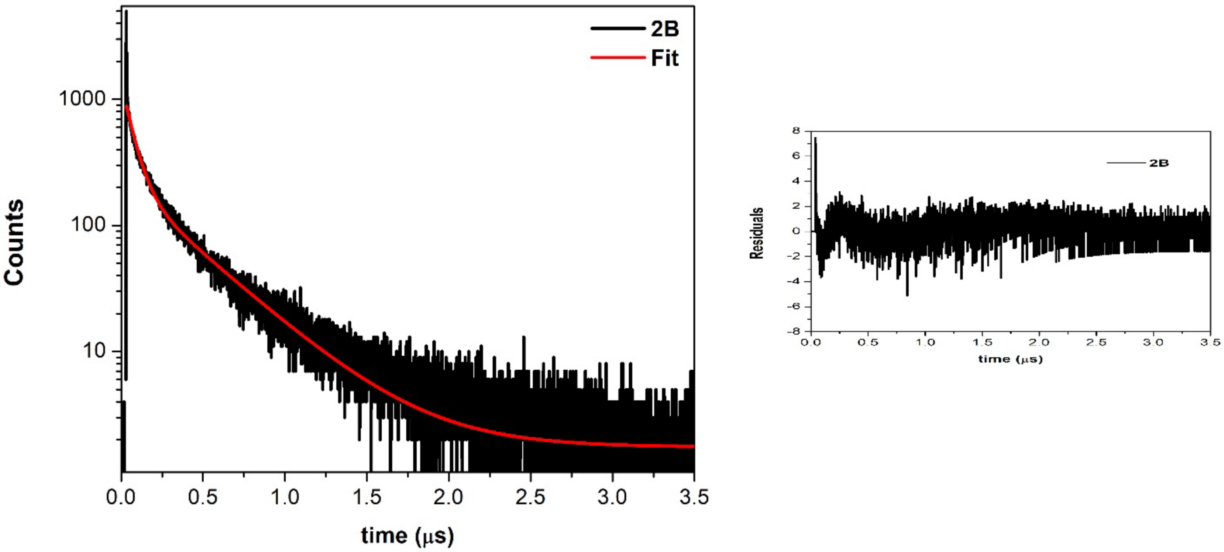


**Figure S78**. Lifetime decay and residual plots of complex **1B** recorded in the solid state.

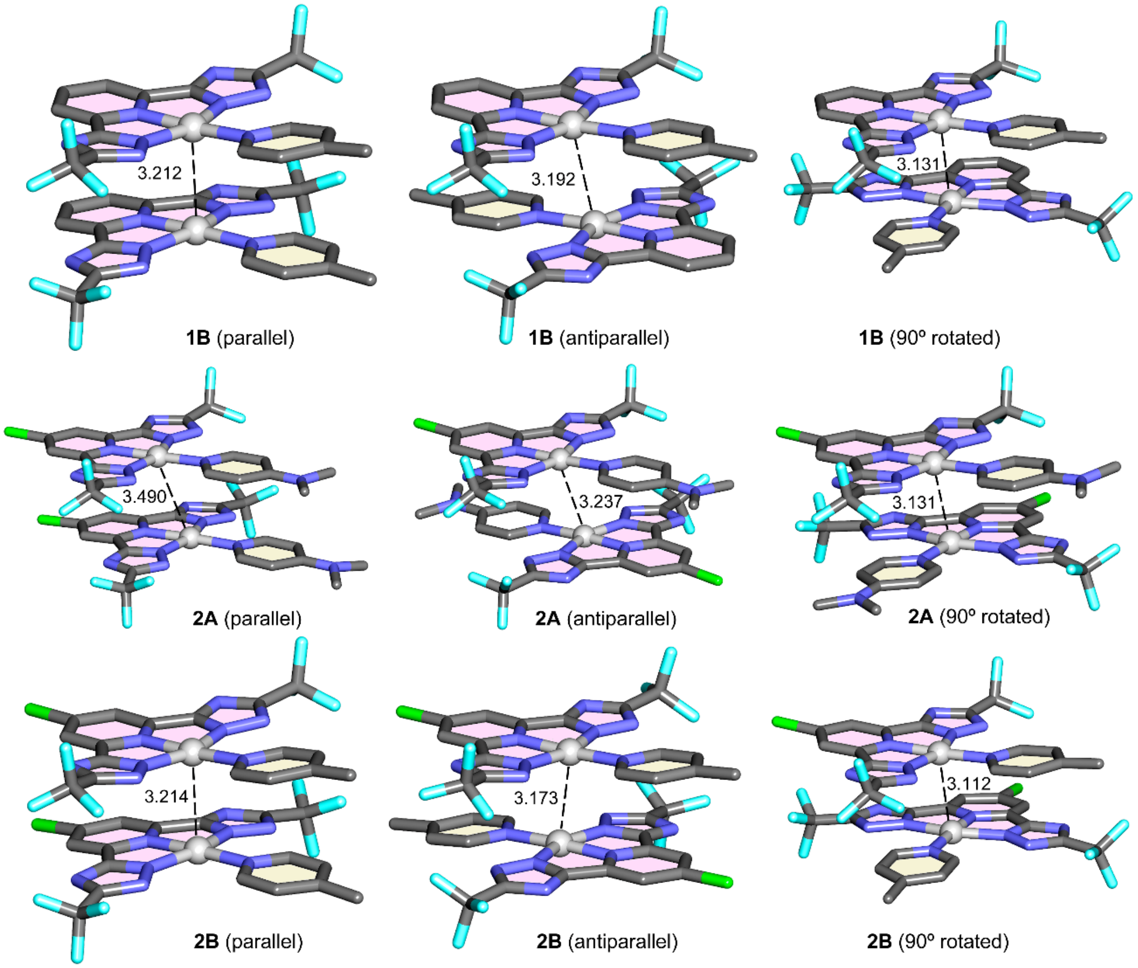


**Figure S79.** Perspective views of the PBE0-D4/def2-TZVP optimized geometries of the dimers **1B** (top), **A2** (middle) and **2B** (bottom) in parallel, antiparallel and 90º rotated binding modes. The Pt···Pt interactions are given in Å. The Pt atoms are represented as grey spheres and the rest of the molecule in "tube" format.

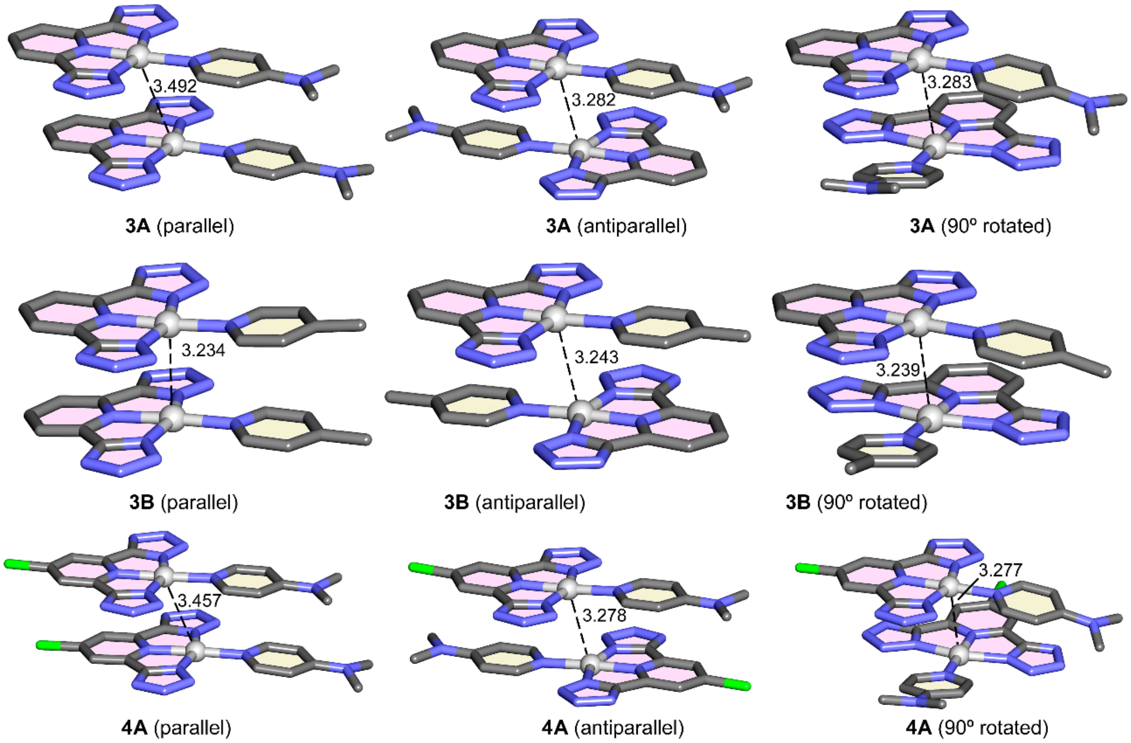


**Figure S80.** Perspective views of the PBE0-D4/def2-TZVP optimized geometries of the dimers **3A** (top), **3B** (middle) and **4A** (bottom) in parallel, antiparallel and 90º rotated binding modes. The Pt···Pt interactions are given in Å. The Pt atoms are represented as grey spheres and the rest of the molecule in "tube" format.

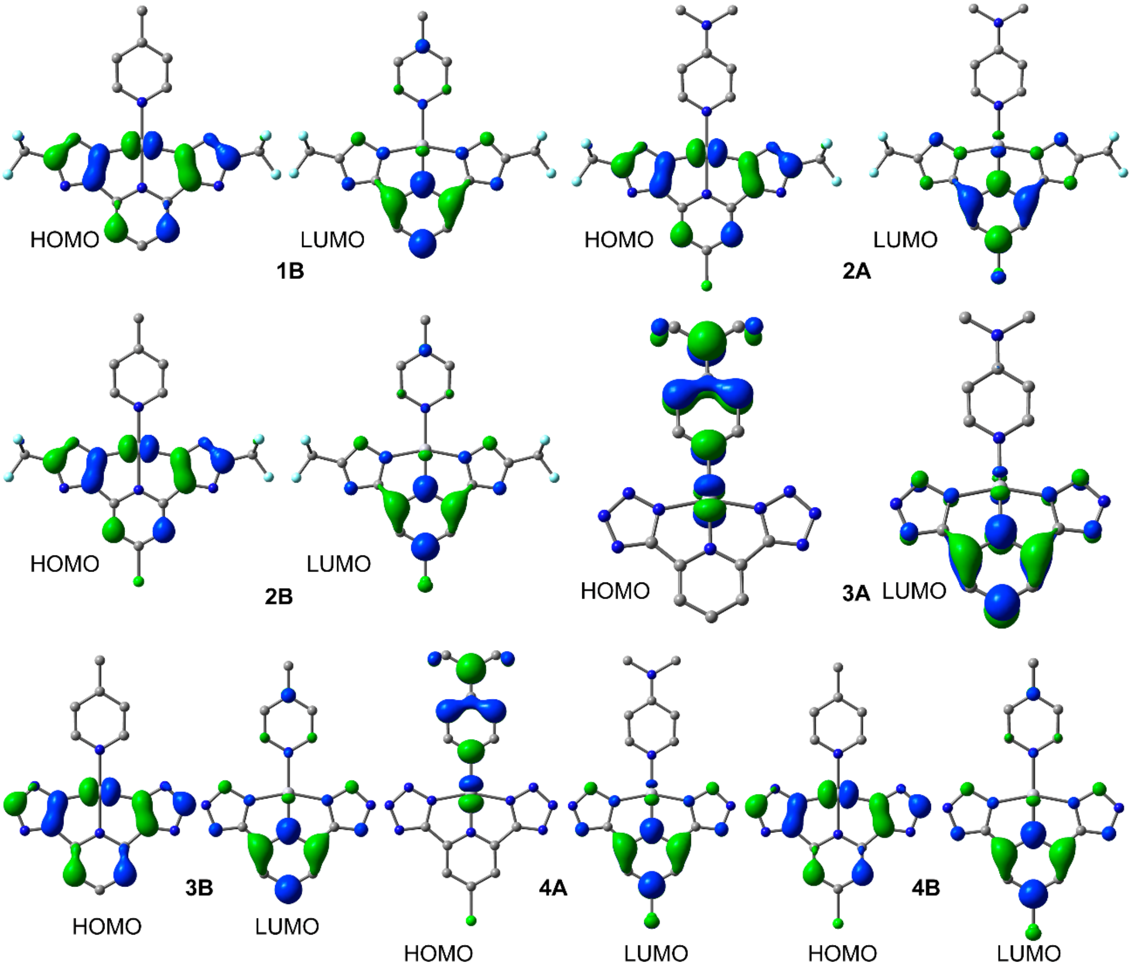


**Figure S81.** Plots of the HOMO and LUMO of compounds **1B, 2A,B-4A,B**.

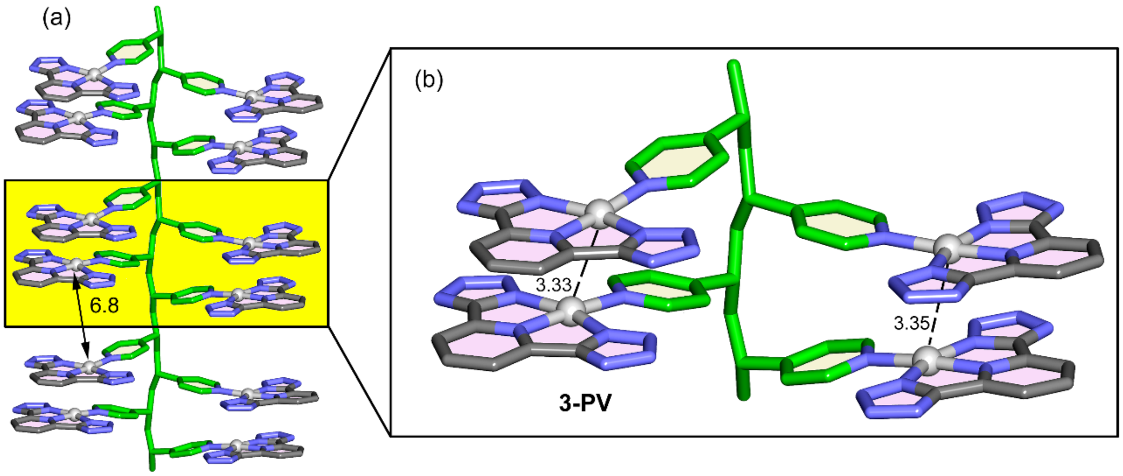


**Figure S82.** (a) Representation of the oligomer **3-PV**. (b) Detail of the tetramer showing the formation of parallel π-stacked dimers. Distances in Å

**Table S1.** Interaction energies ($E_{int}$, kcal/mol) computed for the dimers of compounds **1A,B–4A,B** (three different orientations) in the gas phase and two different solvents (acetonitrile and water) at the PBE0-D4/def2-TZVP (COSMO-RS) level of theory.

| Dimer | Orientation | $E_{int}$ (gas phase) | acetonitrile | $H_2O$ |
|---|---|---|---|---|
| **1A** | 90 | –36.5 | –23.9 | –32.9 |
| **1A** | parallel | –24.7 | –21.2 | –30.0 |
| **1A** | antiparallel | –26.0 | –22.0 | –31.6 |
| **1B** | 90 | –35.0 | –23.6 | –31.6 |
| **1B** | parallel | –19.1 | –17.3 | –25.1 |
| **1B** | antiparallel | –23.4 | –19.6 | –27.9 |
| **2A** | 90 | –36.5 | –24.7 | –33.7 |
| **2A** | parallel | –26.2 | –22.0 | –31.0 |
| **2A** | antiparallel | –29.6 | –23.8 | –33.3 |
| **2B** | 90 | –34.5 | –23.8 | –31.9 |
| **2B** | parallel | –20.5 | –18.0 | –25.7 |
| **2B** | antiparallel | –24.6 | –20.2 | –28.8 |
| **3A** | 90 | –35.4 | –17.9 | –23.7 |
| **3A** | parallel | –18.7 | –18.0 | –25.8 |
| **3A** | antiparallel | –20.8 | –18.8 | –27.1 |
| **3B** | 90 | –33.1 | –18.2 | –22.9 |
| **3B** | parallel | –14.3 | –14.2 | –20.8 |
| **3B** | antiparallel | –18.2 | –16.1 | –23.0 |
| **4A** | 90 | –35.8 | –18.7 | –23.9 |
| **4A** | parallel | –24.4 | –20.5 | –29.2 |
| **4A** | antiparallel | –20.3 | –18.6 | –26.4 |
| **4B** | 90 | –33.4 | –18.7 | –23.0 |
| **4B** | parallel | –15.7 | –14.6 | –21.3 |
| **4B** | antiparallel | –19.3 | –16.6 | –23.9 |

**Table S2.** Binding energies using the unrestricted UKS-PBE0-d4/def2-TZVP level of theory.

| | | **gas phase** | **acetonitrile** | **$H_2O$** |
|---|---|---|---|---|
| **1A** | 90 | -47.7 | -35.5 | -44.4 |
| | parallel | -37.6 | -33.3 | -42.7 |
| | 180 | -35.2 | -31.6 | -40.4 |
| **1B** | 90 | -45.6 | -34.9 | -42.7 |
| | parallel | -29.5 | -27.6 | -35.2 |
| | 180 | -34.0 | -29.9 | -38.0 |
| **2A** | 90 | -47.7 | -36.2 | -45.1 |
| | parallel | -37.3 | -33.3 | -42.2 |
| | 180 | -41.6 | -35.2 | -44.6 |
| **2B** | 90 | -45.2 | -35.0 | -43.0 |
| | parallel | -35.0 | -30.7 | -38.5 |
| | 180 | -35.6 | -31.1 | -39.5 |
| **3A** | 90 | -46.2 | -29.0 | -34.6 |
| | parallel | -29.4 | -28.6 | -36.2 |
| | 180 | -32.5 | -30.1 | -38.2 |
| **3B** | 90 | -43.3 | -28.8 | -33.3 |
| | parallel | -24.6 | -24.2 | -30.4 |
| | 180 | -28.9 | -26.5 | -33.1 |
| **4A** | 90 | -46.8 | -29.9 | -35.2 |
| | parallel | -31.5 | -29.8 | -37.8 |
| | 180 | -36.6 | -32.3 | -41.1 |
| **4B** | 90 | -43.8 | -29.5 | -33.6 |
| | parallel | -26.4 | -25.1 | -31.5 |
| | 180 | -30.4 | -27.5 | -34.5 |

**Table S3.** HOMO-LUMO gaps (eV) computed for the monomers and dimers of compounds **1A,B–4A,B** (three different orientations) in the gas phase and two different solvents (acetonitrile and water) at the PBE0-D4/def2-TZVP (COSMO-RS) level of theory.

| **DIMER** | **gas phase** | **acetonitrile** | **$H_2O$** |
|---|---|---|---|
| **1A,** monomer | 4.12 | 3.93 | 3.86 |
| **1A**, dimer 90° | 3.84 | 3.69 | 3.63 |
| **1A**, dimer parallel | 3.84 | 3.77 | 3.68 |
| **1A**, dimer antiparallel | 3.94 | 3.81 | 3.74 |
| **1B,** monomer | 4.08 | 4.25 | 4.26 |
| **1B**, dimer 90° | 3.89 | 3.96 | 3.94 |
| **1B**, dimer parallel | 3.42 | 3.62 | 3.64 |
| **1B**, dimer antiparallel | 3.76 | 3.95 | 3.97 |
| **2A,** monomer | 4.12 | 3.80 | 3.75 |
| **2A**, dimer 90° | 3.82 | 3.59 | 3.55 |
| **2A**, dimer parallel | 3.86 | 3.63 | 3.56 |
| **2A**, dimer antiparallel | 3.93 | 3.70 | 3.64 |
| **2B,** monomer | 4.09 | 4.23 | 4.24 |
| **2B**, dimer 90° | 3.87 | 3.89 | 3.88 |
| **2B**, dimer parallel | 3.45 | 3.60 | 3.62 |
| **2B**, dimer antiparallel | 3.69 | 3.86 | 3.85 |
| **3A,** monomer | 4.33 | 3.91 | 3.79 |
| **3A**, dimer 90° | 4.00 | 3.75 | 3.63 |
| **3A**, dimer parallel | 4.05 | 3.70 | 3.52 |
| **3A**, dimer antiparallel | 4.12 | 3.80 | 3.67 |
| **3B,** monomer | 4.34 | 4.56 | 4.61 |
| **3B**, dimer 90° | 4.16 | 4.23 | 4.20 |
| **3B**, dimer parallel | 3.68 | 3.92 | 3.90 |
| **3B**, dimer antiparallel | 3.97 | 4.15 | 4.15 |
| **4A,** monomer | 4.25 | 3.78 | 3.68 |
| **4A**, dimer 90° | 3.93 | 3.61 | 3.53 |

| | | | |
|---|---|---|---|
| **4A**, dimer parallel | 4.07 | 3.55 | 3.40 |
| **4A**, dimer antiparallel | 4.01 | 3.68 | 3.57 |
| **4B,** monomer | 4.35 | 4.53 | 4.58 |
| **4B**, dimer 90° | 4.13 | 4.16 | 4.14 |
| **4B**, dimer parallel | 3.71 | 3.84 | 3.83 |
| **4B**, dimer antiparallel | 3.73 | 3.79 | 3.89 |

## Cartesian coordinates of the optimized geometries

**1A monomer.**

| | | | |
|---|---|---|---|
| C | 1.2169036 | -4.5488832 | 0.0970458 |
| C | 1.1907869 | -3.1541736 | 0.0476061 |
| C | -1.1908344 | -3.1546575 | 0.0481618 |
| C | -1.2163205 | -4.5493862 | 0.0976632 |
| C | 0.0004471 | -5.2365147 | 0.1214902 |
| H | 2.1757010 | -5.0631725 | 0.1151413 |
| H | -2.1748620 | -5.0641133 | 0.1162784 |
| Pt | -0.0005114 | -0.5145180 | -0.0470331 |
| C | 1.1608011 | 2.2278907 | -0.1425328 |
| C | -1.1629410 | 2.2276360 | -0.1409130 |
| C | 1.1999339 | 3.6052283 | -0.1868289 |
| H | 2.0836572 | 1.6438670 | -0.1243201 |
| C | -1.2024479 | 3.6049801 | -0.1852685 |
| H | -2.0856503 | 1.6433908 | -0.1215249 |
| C | -0.0013431 | 4.3580263 | -0.2094966 |
| H | 2.1749774 | 4.0856757 | -0.2028993 |
| H | -2.1776121 | 4.0852240 | -0.2004620 |
| C | 2.2984062 | -2.2221296 | 0.0153797 |
| N | 3.6225338 | -2.4163398 | 0.0242299 |
| C | 4.0793537 | -1.1453579 | -0.0217384 |
| C | -2.2988336 | -2.2230080 | 0.0163622 |
| N | -1.9844386 | -0.8887458 | -0.0324826 |
| C | -4.0800821 | -1.1466852 | -0.0202152 |
| N | -0.0001498 | -2.5052403 | 0.0242659 |
| N | -0.0009674 | 1.5246327 | -0.1186004 |
| N | 1.9836229 | -0.8879867 | -0.0333453 |
| N | 3.1273477 | -0.1906502 | -0.0574412 |
| N | -3.1283605 | -0.1917174 | -0.0562261 |
| N | -3.6229172 | -2.4175426 | 0.0256325 |
| N | -0.0014670 | 5.7185138 | -0.2515486 |
| H | 0.0006916 | -6.3258984 | 0.1599560 |
| C | -5.5370608 | -0.7816401 | -0.0233414 |
| C | 5.5362973 | -0.7801586 | -0.0252241 |
| F | -5.8690849 | -0.0410072 | 1.0719681 |
| F | -6.3212823 | -1.8782434 | -0.0298604 |
| F | -5.8620337 | -0.0306342 | -1.1125007 |
| F | 6.3205656 | -1.8767396 | -0.0317272 |
| F | 5.8685003 | -0.0393577 | 1.0699060 |
| F | 5.8610364 | -0.0293223 | -1.1145634 |
| C | -1.2620531 | 6.4493393 | -0.2754042 |
| H | -1.8654959 | 6.2425374 | 0.6227367 |
| H | -1.0512063 | 7.5226613 | -0.3047379 |
| H | -1.8614461 | 6.1925974 | -1.1635698 |
| C | 1.2589833 | 6.4494247 | -0.2789625 |
| H | 1.8626398 | 6.2472221 | 0.6201270 |
| H | 1.8582421 | 6.1882480 | -1.1658854 |
| H | 1.0480596 | 7.5225697 | -0.3139664 |

**1B monomer**

| | | | |
|---|---|---|---|
| C | 1.2166053 | -4.5452618 | 0.0154598 |
| C | 1.1910599 | -3.1497543 | 0.0097155 |
| C | -1.1910774 | -3.1497546 | 0.0096774 |
| C | -1.2165968 | -4.5452685 | 0.0154110 |
| C | -0.0000121 | -5.2325115 | 0.0181335 |
| H | 2.1743387 | -5.0613025 | 0.0176082 |
| H | -2.1743454 | -5.0612996 | 0.0175166 |
| H | 0.0000013 | -6.3224462 | 0.0225111 |
| Pt | -0.0000045 | -0.5081767 | -0.0024854 |
| C | 1.1650700 | 2.2283595 | -0.0140104 |
| C | -1.1650706 | 2.2283559 | -0.0139334 |
| C | 1.1907934 | 3.6148709 | -0.0192852 |
| H | 2.0849312 | 1.6381515 | -0.0144806 |
| C | -1.1907913 | 3.6148696 | -0.0192044 |
| H | -2.0849302 | 1.6381545 | -0.0143555 |
| C | -0.0000023 | 4.3521738 | -0.0194239 |
| H | 2.1589836 | 4.1163104 | -0.0260515 |
| H | -2.1589828 | 4.1163084 | -0.0259056 |
| C | 2.2991204 | -2.2174212 | 0.0055901 |
| N | 3.6228931 | -2.4115870 | 0.0062058 |

| | | | |
|---|---|---|---|
| C | 4.0802331 | -1.1399697 | -0.0000378 |
| C | -2.2991123 | -2.2174180 | 0.0055361 |
| N | -1.9845255 | -0.8827264 | -0.0007519 |
| C | -4.0802389 | -1.1399675 | -0.0000806 |
| C | -0.0000014 | 5.8525779 | 0.0042582 |
| N | 0.0000144 | -2.5013930 | 0.0068209 |
| N | 0.0000020 | 1.5357441 | -0.0103113 |
| N | 1.9845262 | -0.8827214 | -0.0007193 |
| N | 3.1278981 | -0.1847943 | -0.0043675 |
| N | -3.1278975 | -0.1847940 | -0.0043934 |
| N | -3.6228862 | -2.4115933 | 0.0061452 |
| H | 0.8925343 | 6.2629208 | -0.4851256 |
| H | 0.0000941 | 6.2166254 | 1.0440729 |
| H | -0.8926293 | 6.2629153 | -0.4849604 |
| C | -5.5378319 | -0.7742806 | -0.0015903 |
| C | 5.5378374 | -0.7742731 | -0.0015642 |
| F | -5.8651143 | -0.0277512 | 1.0897765 |
| F | -6.3213967 | -1.8708114 | -0.0009559 |
| F | -5.8634833 | -0.0299906 | -1.0948637 |
| F | 5.8651137 | -0.0276907 | 1.0897651 |
| F | 5.8634796 | -0.0300320 | -1.0948720 |
| F | 6.3214016 | -1.8707964 | -0.0008812 |

**2A monomer**

| | | | |
|---|---|---|---|
| C | 1.2230721 | -4.5465899 | 0.0909195 |
| C | 1.1893507 | -3.1538934 | 0.0444274 |
| C | -1.1894863 | -3.1543096 | 0.0449233 |
| C | -1.2226568 | -4.5470274 | 0.0914771 |
| C | 0.0003396 | -5.2252726 | 0.1135542 |
| H | 2.1752949 | -5.0711329 | 0.1084097 |
| H | -2.1746785 | -5.0719380 | 0.1094362 |
| Pt | -0.0005291 | -0.5130062 | -0.0454303 |
| C | 1.1610537 | 2.2283468 | -0.1382781 |
| C | -1.1628851 | 2.2281015 | -0.1358043 |
| C | 1.2001717 | 3.6055788 | -0.1820702 |
| H | 2.0838897 | 1.6444361 | -0.1204287 |
| C | -1.2023700 | 3.6053156 | -0.1795871 |
| H | -2.0855323 | 1.6439912 | -0.1162154 |
| C | -0.0012054 | 4.3584874 | -0.2043402 |
| H | 2.1751875 | 4.0860699 | -0.1982991 |
| H | -2.1774922 | 4.0856213 | -0.1940019 |
| C | 2.2980108 | -2.2239685 | 0.0139780 |
| N | 3.6216572 | -2.4191124 | 0.0224584 |
| C | 4.0791454 | -1.1487641 | -0.0210665 |
| C | -2.2984435 | -2.2247089 | 0.0148906 |
| N | -1.9840800 | -0.8896064 | -0.0313579 |
| C | -4.0799008 | -1.1500020 | -0.0195302 |
| N | -0.0001783 | -2.5030354 | 0.0224070 |
| N | -0.0008275 | 1.5250883 | -0.1143838 |
| N | 1.9832215 | -0.8889310 | -0.0322541 |
| N | 3.1271642 | -0.1931898 | -0.0550190 |
| N | -3.1281743 | -0.1942061 | -0.0537054 |
| N | -3.6220647 | -2.4202621 | 0.0237636 |
| Cl | 0.0006672 | -6.9561062 | 0.1707840 |
| N | -0.0014019 | 5.7186849 | -0.2466492 |
| C | -5.5375194 | -0.7855505 | -0.0225468 |
| C | 5.5366829 | -0.7840079 | -0.0244649 |
| F | -5.8620586 | -0.0361458 | -1.1125726 |
| F | -5.8682818 | -0.0440036 | 1.0720428 |
| F | -6.3204702 | -1.8825756 | -0.0276863 |
| F | 6.3198186 | -1.8808881 | -0.0296787 |
| F | 5.8608460 | -0.0346461 | -1.1146393 |
| F | 5.8675611 | -0.0423028 | 1.0699828 |
| C | -1.2621135 | 6.4495738 | -0.2707832 |
| H | -1.8651555 | 6.2436020 | 0.6277676 |
| H | -1.0512918 | 7.5228486 | -0.3012612 |
| H | -1.8615613 | 6.1919159 | -1.1585880 |
| C | 1.2590640 | 6.4498681 | -0.2740059 |
| H | 1.8620787 | 6.2488029 | 0.6257266 |
| H | 1.8587607 | 6.1878685 | -1.1603115 |
| H | 1.0478969 | 7.5228840 | -0.3103754 |

**2B monomer**

| | | | |
|---|---|---|---|
| C | 1.2230275 | -4.5432358 | 0.0155091 |
| C | 1.1897247 | -3.1500107 | 0.0097203 |
| C | -1.1897112 | -3.1500093 | 0.0096817 |
| C | -1.2230495 | -4.5432389 | 0.0154596 |
| C | 0.0000132 | -5.2223349 | 0.0181120 |
| H | 2.1756599 | -5.0672763 | 0.0176457 |
| H | -2.1756504 | -5.0672746 | 0.0175537 |
| Pt | 0.0000007 | -0.5074164 | -0.0023757 |
| C | 1.1649817 | 2.2287721 | -0.0139499 |
| C | -1.1649720 | 2.2287726 | -0.0138740 |
| C | 1.1907447 | 3.6152092 | -0.0192739 |
| H | 2.0848464 | 1.6390480 | -0.0145858 |
| C | -1.1907458 | 3.6152040 | -0.0191935 |
| H | -2.0848440 | 1.6390455 | -0.0144621 |
| C | 0.0000020 | 4.3525851 | -0.0195271 |
| H | 2.1591155 | 4.1163903 | -0.0259922 |
| H | -2.1591151 | 4.1163862 | -0.0258458 |
| C | 2.2990045 | -2.2197881 | 0.0055891 |
| N | 3.6225108 | -2.4143222 | 0.0062195 |
| C | 4.0800916 | -1.1432029 | -0.0000168 |
| C | -2.2990136 | -2.2197802 | 0.0055335 |
| N | -1.9840951 | -0.8844176 | -0.0007417 |
| C | -4.0800841 | -1.1432104 | -0.0000608 |
| C | -0.0000017 | 5.8528956 | 0.0040989 |
| N | -0.0000061 | -2.4996309 | 0.0068018 |
| N | -0.0000019 | 1.5358683 | -0.0102653 |
| N | 1.9840905 | -0.8844170 | -0.0007080 |
| N | 3.1273658 | -0.1876649 | -0.0043554 |
| N | -3.1273635 | -0.1876696 | -0.0043825 |
| N | -3.6225098 | -2.4143225 | 0.0061577 |
| H | 0.8927382 | 6.2629914 | -0.4850855 |
| H | 0.0000943 | 6.2166232 | 1.0440738 |
| H | -0.8928335 | 6.2629873 | -0.4849213 |
| Cl | 0.0000011 | -6.9526208 | 0.0249196 |
| C | -5.5383333 | -0.7778249 | -0.0015812 |
| C | 5.5383278 | -0.7778197 | -0.0015549 |
| F | -5.8648386 | -0.0315371 | 1.0897695 |
| F | -6.3207011 | -1.8748013 | -0.0009306 |
| F | -5.8632010 | -0.0338165 | -1.0948690 |
| F | 5.8648354 | -0.0314729 | 1.0897576 |
| F | 6.3207043 | -1.8747896 | -0.0008516 |
| F | 5.8631943 | -0.0338621 | -1.0948823 |

**3A monomer**

| | | | |
|---|---|---|---|
| C | 1.2175225 | -4.5319466 | 0.0809753 |
| C | 1.1922711 | -3.1377621 | 0.0376697 |
| C | -1.1921016 | -3.1385279 | 0.0399854 |
| C | -1.2163395 | -4.5327366 | 0.0835601 |
| C | 0.0008415 | -5.2196239 | 0.1035321 |
| H | 2.1755341 | -5.0477963 | 0.0960486 |
| H | -2.1739644 | -5.0492462 | 0.1007848 |
| Pt | -0.0008120 | -0.5019249 | -0.0456842 |
| C | 1.1606184 | 2.2407043 | -0.1327633 |
| C | -1.1634523 | 2.2402640 | -0.1376768 |
| C | 1.1996925 | 3.6178852 | -0.1771522 |
| H | 2.0839790 | 1.6585862 | -0.1117132 |
| C | -1.2028842 | 3.6174874 | -0.1818384 |
| H | -2.0866098 | 1.6576865 | -0.1205947 |
| C | -0.0016682 | 4.3703726 | -0.2034263 |
| H | 2.1746724 | 4.0983951 | -0.1901275 |
| H | -2.1779804 | 4.0976044 | -0.2003592 |
| C | 2.2966305 | -2.2003608 | 0.0074943 |
| N | 3.6180800 | -2.3632241 | 0.0132150 |
| C | -2.2971066 | -2.2017949 | 0.0114527 |
| N | -1.9862376 | -0.8733947 | -0.0318772 |
| N | -0.0001399 | -2.4899931 | 0.0185540 |
| N | -0.0013277 | 1.5366599 | -0.1124124 |
| N | 1.9848861 | -0.8721577 | -0.0348520 |
| N | 3.1456681 | -0.2102300 | -0.0555849 |

| | | | |
|---|---|---|---|
| N | -3.1474316 | -0.2121752 | -0.0513878 |
| N | -3.6184566 | -2.3654335 | 0.0190242 |
| N | -0.0015373 | 5.7305968 | -0.2465451 |
| H | 0.0012493 | -6.3090828 | 0.1373008 |
| N | -4.1240937 | -1.1109000 | -0.0207129 |
| N | 4.1229100 | -1.1083796 | -0.0265972 |
| C | -1.2621372 | 6.4614968 | -0.2655689 |
| H | -1.8620316 | 6.2537468 | 0.6347429 |
| H | -1.0517134 | 7.5348738 | -0.2952113 |
| H | -1.8647234 | 6.2050054 | -1.1515711 |
| C | 1.2594918 | 6.4604390 | -0.2750176 |
| H | 1.8626412 | 6.2602692 | 0.6248948 |
| H | 1.8586218 | 6.1955425 | -1.1608137 |
| H | 1.0498154 | 7.5336275 | -0.3138546 |

**3B monomer**

| | | | |
|---|---|---|---|
| C | 1.2171767 | -4.5289361 | 0.0147236 |
| C | 1.1923826 | -3.1338874 | 0.0091611 |
| C | -1.1923754 | -3.1338923 | 0.0091009 |
| C | -1.2171640 | -4.5289414 | 0.0146547 |
| C | 0.0000083 | -5.2161157 | 0.0174449 |
| H | 2.1744930 | -5.0464649 | 0.0168128 |
| H | -2.1744776 | -5.0464740 | 0.0166859 |
| Pt | -0.0000034 | -0.4956080 | -0.0013795 |
| C | 1.1650463 | 2.2421427 | -0.0133143 |
| C | -1.1650696 | 2.2421426 | -0.0132266 |
| C | 1.1909137 | 3.6285408 | -0.0202861 |
| H | 2.0855592 | 1.6545218 | -0.0133051 |
| C | -1.1909356 | 3.6285429 | -0.0201962 |
| H | -2.0855838 | 1.6545234 | -0.0131609 |
| C | -0.0000114 | 4.3655550 | -0.0212806 |
| H | 2.1592353 | 4.1300738 | -0.0274643 |
| H | -2.1592568 | 4.1300778 | -0.0273034 |
| C | 2.2967548 | -2.1957872 | 0.0054186 |
| N | 3.6179150 | -2.3580110 | 0.0058877 |
| C | -2.2967521 | -2.1957972 | 0.0053203 |
| N | -1.9849087 | -0.8670730 | 0.0000038 |
| N | 0.0000020 | -2.4864563 | 0.0065994 |
| N | -0.0000121 | 1.5483387 | -0.0089670 |
| N | 1.9849060 | -0.8670644 | 0.0000786 |
| N | 3.1449572 | -0.2042801 | -0.0028642 |
| N | -3.1449620 | -0.2042928 | -0.0029610 |
| N | -3.6179118 | -2.3580257 | 0.0057523 |
| N | -4.1224404 | -1.1023904 | 0.0005257 |
| N | 4.1224389 | -1.1023739 | 0.0006589 |
| C | -0.0000021 | 5.8658839 | 0.0006375 |
| H | 0.0002732 | 6.2308957 | 1.0401169 |
| H | 0.8925172 | 6.2755479 | -0.4892961 |
| H | -0.8927625 | 6.2755602 | -0.4888414 |
| H | 0.0000104 | -6.3062052 | 0.0217638 |

**4A monomer**

| | | | |
|---|---|---|---|
| C | 1.2236714 | -4.5311031 | 0.0749824 |
| C | 1.1908830 | -3.1389423 | 0.0346807 |
| C | -1.1908114 | -3.1396114 | 0.0367795 |
| C | -1.2226784 | -4.5317953 | 0.0772810 |
| C | 0.0007166 | -5.2102773 | 0.0956943 |
| H | 2.1750822 | -5.0573671 | 0.0894652 |
| H | -2.1737185 | -5.0586550 | 0.0936055 |
| Pt | -0.0007377 | -0.5015717 | -0.0434818 |
| C | 1.1608397 | 2.2402309 | -0.1259199 |
| C | -1.1634094 | 2.2398667 | -0.1299346 |
| C | 1.1998810 | 3.6173666 | -0.1685661 |
| H | 2.0841994 | 1.6582230 | -0.1062008 |
| C | -1.2027907 | 3.6170410 | -0.1720297 |
| H | -2.0865730 | 1.6574489 | -0.1137260 |
| C | -0.0015093 | 4.3701542 | -0.1934166 |
| H | 2.1748604 | 4.0978899 | -0.1810704 |
| H | -2.1778743 | 4.0972446 | -0.1883639 |
| C | 2.2962181 | -2.2033062 | 0.0065335 |
| N | 3.6174088 | -2.3669440 | 0.0110901 |

| | | | |
|---|---|---|---|
| C | -2.2966930 | -2.2045413 | 0.0102054 |
| N | -1.9857309 | -0.8754104 | -0.0306239 |
| N | -0.0001598 | -2.4891835 | 0.0165272 |
| N | -0.0011966 | 1.5361152 | -0.1063812 |
| N | 1.9844947 | -0.8743516 | -0.0334832 |
| N | 3.1452525 | -0.2137745 | -0.0538819 |
| N | -3.1468563 | -0.2154544 | -0.0499110 |
| N | -3.6177924 | -2.3688585 | 0.0163716 |
| N | -0.0014282 | 5.7301816 | -0.2350046 |
| N | -4.1236278 | -1.1151705 | -0.0215833 |
| N | 4.1225350 | -1.1129763 | -0.0270012 |
| Cl | 0.0013450 | -6.9406787 | 0.1456604 |
| C | -1.2620774 | 6.4614657 | -0.2555287 |
| H | -1.8627442 | 6.2552121 | 0.6445858 |
| H | -1.0511055 | 7.5346535 | -0.2865205 |
| H | -1.8639546 | 6.2044237 | -1.1418005 |
| C | 1.2594210 | 6.4607921 | -0.2639380 |
| H | 1.8625178 | 6.2615508 | 0.6361644 |
| H | 1.8586712 | 6.1961235 | -1.1497673 |
| H | 1.0487559 | 7.5338063 | -0.3029453 |

**4B monomer**

| | | | |
|---|---|---|---|
| C | 1.2234186 | -4.5274438 | 0.0147143 |
| C | 1.1910981 | -3.1348103 | 0.0092369 |
| C | -1.1910918 | -3.1348144 | 0.0091786 |
| C | -1.2234075 | -4.5274506 | 0.0146479 |
| C | 0.0000089 | -5.2067386 | 0.0173943 |
| H | 2.1746043 | -5.0544468 | 0.0168348 |
| H | -2.1745886 | -5.0544565 | 0.0167127 |
| Pt | -0.0000038 | -0.4957351 | -0.0015785 |
| C | 1.1651497 | 2.2406627 | -0.0133462 |
| C | -1.1651694 | 2.2406609 | -0.0132606 |
| C | 1.1907904 | 3.6269099 | -0.0202744 |
| H | 2.0858618 | 1.6533830 | -0.0132916 |
| C | -1.1908110 | 3.6269110 | -0.0201845 |
| H | -2.0858818 | 1.6533822 | -0.0131523 |
| C | -0.0000106 | 4.3638689 | -0.0211698 |
| H | 2.1585285 | 4.1289711 | -0.0274530 |
| H | -2.1585485 | 4.1289723 | -0.0272897 |
| C | 2.2967903 | -2.1987339 | 0.0054492 |
| N | 3.6178267 | -2.3612370 | 0.0058909 |
| C | -2.2967871 | -2.1987458 | 0.0053526 |
| N | -1.9845783 | -0.8694929 | -0.0000090 |
| N | 0.0000014 | -2.4854024 | 0.0066499 |
| N | -0.0000100 | 1.5469628 | -0.0089310 |
| N | 1.9845738 | -0.8694838 | 0.0000641 |
| N | 3.1443684 | -0.2076374 | -0.0028345 |
| N | -3.1443762 | -0.2076522 | -0.0029308 |
| N | -3.6178244 | -2.3612545 | 0.0057539 |
| N | -4.1222067 | -1.1063217 | 0.0005503 |
| N | 4.1222033 | -1.1063021 | 0.0006860 |
| Cl | 0.0000117 | -6.9365475 | 0.0243090 |
| C | -0.0000026 | 5.8639178 | 0.0004739 |
| H | 0.0002725 | 6.2282673 | 1.0401792 |
| H | 0.8928846 | 6.2730511 | -0.4892763 |
| H | -0.8931309 | 6.2730622 | -0.4888220 |

**1A dimer parallel**

| | | | |
|---|---|---|---|
| C | 1.2116021 | -3.9264127 | 0.6161159 |
| C | 1.1872245 | -2.5363063 | 0.4999378 |
| C | -1.1990492 | -2.5359495 | 0.4994483 |
| C | -1.2233689 | -3.9261224 | 0.6161532 |
| C | -0.0059249 | -4.6098013 | 0.6752817 |
| H | 2.1700187 | -4.4398972 | 0.6588016 |
| H | -2.1816347 | -4.4397992 | 0.6596234 |
| Pt | -0.0048530 | 0.0914809 | 0.3067961 |
| C | 1.1560530 | 2.8128234 | -0.0277278 |
| C | -1.1644659 | 2.8139112 | -0.0268289 |
| C | 1.1955074 | 4.1754239 | -0.2414444 |
| H | 2.0786343 | 2.2310408 | 0.0461197 |
| C | -1.2029133 | 4.1765712 | -0.2405864 |

| | | | |
|---|---|---|---|
| H | -2.0875800 | 2.2330052 | 0.0474308 |
| C | -0.0034160 | 4.9213674 | -0.3452874 |
| H | 2.1715914 | 4.6418340 | -0.3461814 |
| H | -2.1785093 | 4.6441093 | -0.3452236 |
| C | 2.2950220 | -1.6065309 | 0.4077851 |
| N | 3.6191419 | -1.7944537 | 0.3444857 |
| C | 4.0669417 | -0.5247560 | 0.2156580 |
| C | -2.3062718 | -1.6053117 | 0.4062343 |
| N | -1.9832278 | -0.2756669 | 0.3169488 |
| C | -4.0763694 | -0.5206652 | 0.2119398 |
| N | -0.0057365 | -1.8927708 | 0.4535069 |
| N | -0.0044728 | 2.1194532 | 0.0793741 |
| N | 1.9738985 | -0.2765363 | 0.3179378 |
| N | 3.1083837 | 0.4230155 | 0.1962185 |
| N | -3.1163141 | 0.4254978 | 0.1939841 |
| N | -3.6307570 | -1.7911391 | 0.3410925 |
| N | -0.0027209 | 6.2761097 | -0.5355485 |
| H | -0.0059328 | -5.6953560 | 0.7698612 |
| C | -5.5119193 | -0.1587151 | -0.0388601 |
| C | 5.5040082 | -0.1677293 | -0.0334256 |
| F | -5.8157213 | 1.0589613 | 0.4776822 |
| F | -6.3626384 | -1.0660526 | 0.4771306 |
| F | -5.7662633 | -0.0882635 | -1.3827565 |
| F | 6.3505052 | -1.0765574 | 0.4871933 |
| F | 5.8111035 | 1.0503982 | 0.4797951 |
| F | 5.7613214 | -0.1027349 | -1.3769931 |
| C | 1.2236881 | -5.0791785 | 4.0629798 |
| C | 1.1960900 | -3.6928299 | 3.9020381 |
| C | -1.1833333 | -3.6950608 | 3.9005953 |
| C | -1.2084637 | -5.0814385 | 4.0617531 |
| C | 0.0082197 | -5.7638002 | 4.1408385 |
| H | 2.1835660 | -5.5870770 | 4.1318066 |
| H | -2.1674168 | -5.5911996 | 4.1296505 |
| Pt | 0.0041869 | -1.0640828 | 3.6396991 |
| C | 1.1648117 | 1.6642366 | 3.4235177 |
| C | -1.1601179 | 1.6629942 | 3.4230023 |
| C | 1.2024487 | 3.0328424 | 3.2642950 |
| H | 2.0876115 | 1.0829927 | 3.4867752 |
| C | -1.1989863 | 3.0315101 | 3.2636025 |
| H | -2.0823949 | 1.0808224 | 3.4853940 |
| C | 0.0013710 | 3.7756723 | 3.1590745 |
| H | 2.1778595 | 3.5100633 | 3.2192066 |
| H | -2.1747882 | 3.5077570 | 3.2170102 |
| C | 2.3023695 | -2.7657336 | 3.8227140 |
| N | 3.6239534 | -2.9550293 | 3.8914207 |
| C | 4.0832769 | -1.6884851 | 3.7882025 |
| C | -2.2911809 | -2.7699956 | 3.8190665 |
| N | -1.9798370 | -1.4403098 | 3.6830778 |
| C | -4.0740022 | -1.6960247 | 3.7795240 |
| N | 0.0058131 | -3.0483256 | 3.8226632 |
| N | 0.0027085 | 0.9688929 | 3.4982294 |
| N | 1.9888948 | -1.4366414 | 3.6861238 |
| N | 3.1328952 | -0.7390393 | 3.6634760 |
| N | -3.1250861 | -0.7448291 | 3.6573126 |
| N | -3.6125959 | -2.9617453 | 3.8841163 |
| N | 0.0002400 | 5.1248943 | 2.9606380 |
| H | 0.0091819 | -6.8460842 | 4.2721117 |
| C | -5.5295117 | -1.3498163 | 3.8987388 |
| C | 5.5377258 | -1.3393531 | 3.9121156 |
| F | -5.9129354 | -1.3050095 | 5.2111143 |
| F | -6.3156685 | -2.2624214 | 3.2900052 |
| F | -5.7927964 | -0.1319866 | 3.3684192 |
| F | 6.3279080 | -2.2520476 | 3.3087672 |
| F | 5.9160710 | -1.2905771 | 5.2258576 |
| F | 5.8009119 | -0.1224042 | 3.3797270 |
| C | 1.2535287 | 5.8620222 | 2.9514096 |
| H | 1.0535299 | 6.8998738 | 2.6657068 |
| H | 1.7461545 | 5.8610658 | 3.9385185 |
| H | 1.9508450 | 5.4326408 | 2.2166461 |
| C | -1.2548057 | 5.8591115 | 2.9541204 |
| H | -1.9500550 | 5.4328541 | 2.2154976 |

| | | | |
|---|---|---|---|
| H | -1.7487801 | 5.8501152 | 3.9404613 |
| H | -1.0569728 | 6.8993630 | 2.6759000 |
| C | 1.2470451 | 6.9614044 | -0.8401307 |
| H | 1.0626790 | 8.0403526 | -0.8703269 |
| H | 1.9982343 | 6.7702098 | -0.0616035 |
| H | 1.6690229 | 6.6516213 | -1.8118856 |
| C | -1.2518838 | 6.9640952 | -0.8365608 |
| H | -1.6762631 | 6.6574750 | -1.8082919 |
| H | -2.0020843 | 6.7726514 | -0.0571584 |
| H | -1.0655712 | 8.0428001 | -0.8646019 |

**1A dimer 90° rotated**

| | | | |
|---|---|---|---|
| C | 1.1578442 | -4.5927254 | 0.1613803 |
| C | 1.1252955 | -3.2002712 | 0.2164467 |
| C | -1.2510950 | -3.2069779 | 0.1147240 |
| C | -1.2699906 | -4.5994631 | 0.0517842 |
| C | -0.0534144 | -5.2838682 | 0.0774384 |
| H | 2.1166005 | -5.1053464 | 0.1935988 |
| H | -2.2242734 | -5.1178086 | -0.0131556 |
| Pt | -0.0751947 | -0.5636483 | 0.2855494 |
| C | 1.0799468 | 2.1741775 | 0.3476151 |
| C | -1.2416538 | 2.1799086 | 0.3102822 |
| C | 1.1238574 | 3.5497431 | 0.3576362 |
| H | 2.0007979 | 1.5884439 | 0.3587436 |
| C | -1.2772377 | 3.5569858 | 0.3248938 |
| H | -2.1639098 | 1.5971978 | 0.2766337 |
| C | -0.0745633 | 4.3066704 | 0.3441883 |
| H | 2.0991053 | 4.0277984 | 0.3851989 |
| H | -2.2498035 | 4.0416030 | 0.3130731 |
| C | 2.2324022 | -2.2663300 | 0.2738848 |
| N | 3.5571611 | -2.4537177 | 0.2614878 |
| C | 4.0105385 | -1.1796092 | 0.3067977 |
| C | -2.3623999 | -2.2778485 | 0.0668987 |
| N | -2.0534281 | -0.9427265 | 0.1352910 |
| C | -4.1349776 | -1.1953556 | -0.0951248 |
| N | -0.0662835 | -2.5537135 | 0.1988359 |
| N | -0.0830329 | 1.4751219 | 0.3151988 |
| N | 1.9138661 | -0.9332711 | 0.3219381 |
| N | 3.0559014 | -0.2294945 | 0.3465957 |
| N | -3.1910915 | -0.2423741 | 0.0328808 |
| N | -3.6790986 | -2.4689265 | -0.0824006 |
| N | -0.0654728 | 5.6660996 | 0.3466116 |
| H | -0.0475192 | -6.3724219 | 0.0420903 |
| C | -5.5680044 | -0.8421283 | -0.3473911 |
| C | 5.4682573 | -0.8334535 | 0.2360242 |
| F | -5.8702724 | 0.3963341 | 0.1251673 |
| F | -6.4185790 | -1.7266833 | 0.2380275 |
| F | -5.8642927 | -0.8400353 | -1.6765742 |
| F | 6.2178542 | -1.6369894 | 1.0359528 |
| F | 5.6952568 | 0.4497811 | 0.6120284 |
| F | 5.9568335 | -0.9747718 | -1.0272923 |
| C | 4.0722910 | 0.8152778 | 3.6936150 |
| C | 2.6808022 | 0.7945134 | 3.6127613 |
| C | 2.6769628 | -1.5830027 | 3.5449037 |
| C | 4.0686144 | -1.6138314 | 3.6193925 |
| C | 4.7578260 | -0.4009172 | 3.6948737 |
| H | 4.5890119 | 1.7708533 | 3.7508177 |
| H | 4.5821374 | -2.5725549 | 3.6079710 |
| Pt | 0.0409083 | -0.3849378 | 3.4196221 |
| C | -2.7028708 | 0.7805668 | 3.3383360 |
| C | -2.6962992 | -1.5411449 | 3.3293841 |
| C | -4.0798700 | 0.8156850 | 3.3060921 |
| H | -2.1214369 | 1.7037626 | 3.3680572 |
| C | -4.0716497 | -1.5854952 | 3.3029686 |
| H | -2.1103742 | -2.4618322 | 3.3366150 |
| C | -4.8292008 | -0.3873635 | 3.2944821 |
| H | -4.5648937 | 1.7882455 | 3.3006238 |
| H | -4.5483764 | -2.5614895 | 3.2792560 |
| C | 1.7506293 | 1.9058140 | 3.6324689 |
| N | 1.9394294 | 3.2245006 | 3.7661228 |
| C | 0.6658465 | 3.6801455 | 3.7538598 |

| | | | |
|---|---|---|---|
| C | 1.7441941 | -2.6911749 | 3.4909269 |
| N | 0.4118938 | -2.3738899 | 3.4181219 |
| C | 0.6580776 | -4.4697690 | 3.4728622 |
| N | 2.0295093 | -0.3918313 | 3.5366518 |
| N | -1.9976220 | -0.3777607 | 3.3574263 |
| N | 0.4167294 | 1.5954945 | 3.5488720 |
| N | -0.2851367 | 2.7343387 | 3.6249624 |
| N | -0.2914613 | -3.5163110 | 3.4026591 |
| N | 1.9313603 | -4.0153568 | 3.5286428 |
| N | -6.1885740 | -0.3982319 | 3.2799240 |
| H | 5.8458961 | -0.4053741 | 3.7435831 |
| C | 0.3117070 | -5.9259685 | 3.5656665 |
| C | 0.3089156 | 5.1168195 | 3.9781882 |
| F | 0.4524028 | -6.3947039 | 4.8365593 |
| F | 1.1156281 | -6.6880360 | 2.7776108 |
| F | -0.9709939 | -6.1584405 | 3.1923221 |
| F | 1.2023864 | 5.9585302 | 3.3935133 |
| F | 0.2853834 | 5.4342458 | 5.3022167 |
| F | -0.9218740 | 5.4108116 | 3.4808292 |
| C | -6.9104221 | -1.6563980 | 3.4282082 |
| H | -6.7148992 | -2.1236715 | 4.4081224 |
| H | -7.9838318 | -1.4604081 | 3.3454823 |
| H | -6.6355246 | -2.3659200 | 2.6363217 |
| C | -6.9299780 | 0.8546313 | 3.2946905 |
| H | -6.8088733 | 1.3967509 | 4.2482726 |
| H | -6.6054000 | 1.5081536 | 2.4730436 |
| H | -7.9934342 | 0.6397524 | 3.1511420 |
| C | -1.3187988 | 6.4049343 | 0.2959207 |
| H | -1.1084800 | 7.4702316 | 0.4325543 |
| H | -1.8393705 | 6.2719271 | -0.6681932 |
| H | -1.9894213 | 6.0878793 | 1.1064925 |
| C | 1.1930257 | 6.3892129 | 0.2106983 |
| H | 1.8968947 | 6.1089969 | 1.0053499 |
| H | 1.6669876 | 6.2015601 | -0.7675915 |
| H | 0.9962829 | 7.4617999 | 0.3013621 |

**1A dimer 180° rotated**

| | | | |
|---|---|---|---|
| C | 1.2154919 | -4.9423893 | 0.1070798 |
| C | 1.1895040 | -3.5510047 | 0.2184707 |
| C | -1.1902460 | -3.5513187 | 0.2180441 |
| C | -1.2157902 | -4.9427097 | 0.1065959 |
| C | -0.0000435 | -5.6288842 | 0.0512553 |
| H | 2.1756757 | -5.4498634 | 0.0393603 |
| H | -2.1757584 | -5.4505082 | 0.0384025 |
| Pt | -0.0006750 | -0.9090701 | 0.2992969 |
| C | 1.1616914 | 1.8249858 | 0.2130806 |
| C | -1.1633909 | 1.8251324 | 0.2138491 |
| C | 1.2007281 | 3.2016233 | 0.2575320 |
| H | 2.0837607 | 1.2397352 | 0.1919207 |
| C | -1.2021926 | 3.2017546 | 0.2584145 |
| H | -2.0854843 | 1.2398175 | 0.1931671 |
| C | -0.0006704 | 3.9507133 | 0.3136833 |
| H | 2.1759799 | 3.6811813 | 0.2593295 |
| H | -2.1773726 | 3.6815677 | 0.2607542 |
| C | 2.2959584 | -2.6212487 | 0.2335939 |
| N | 3.6176374 | -2.8146770 | 0.1531714 |
| C | 4.0753211 | -1.5429244 | 0.1361897 |
| C | -2.2969706 | -2.6217614 | 0.2329674 |
| N | -1.9845368 | -1.2856766 | 0.2638753 |
| C | -4.0765205 | -1.5436150 | 0.1361350 |
| N | -0.0004387 | -2.9027123 | 0.2717173 |
| N | -0.0008991 | 1.1268232 | 0.2130814 |
| N | 1.9834355 | -1.2852195 | 0.2643589 |
| N | 3.1243238 | -0.5882356 | 0.2006371 |
| N | -3.1255244 | -0.5887816 | 0.2005541 |
| N | -3.6185442 | -2.8153165 | 0.1527797 |
| N | -0.0005707 | 5.3075916 | 0.4290691 |
| H | 0.0001395 | -6.7136604 | -0.0566300 |
| C | -5.5239093 | -1.2011938 | -0.0702568 |
| C | 5.5226724 | -1.2004872 | -0.0713293 |
| F | -5.8083411 | 0.0453128 | 0.3719397 |

| | | | |
|---|---|---|---|
| F | -6.3428626 | -2.0762951 | 0.5534773 |
| F | -5.8454606 | -1.2350672 | -1.3995499 |
| F | 6.3420776 | -2.0757080 | 0.5515857 |
| F | 5.8075022 | 0.0459420 | 0.3707250 |
| F | 5.8430035 | -1.2342706 | -1.4009252 |
| C | -1.2159979 | 3.9640534 | 3.6099225 |
| C | -1.1899376 | 2.5728674 | 3.4967962 |
| C | 1.1898445 | 2.5732848 | 3.4972099 |
| C | 1.2152887 | 3.9645654 | 3.6104386 |
| C | -0.0005206 | 4.6505246 | 3.6666346 |
| H | -2.1762385 | 4.4713857 | 3.6784611 |
| H | 2.1752256 | 4.4723013 | 3.6794639 |
| Pt | 0.0003999 | -0.0689130 | 3.4136733 |
| C | -1.1617987 | -2.8029894 | 3.5008668 |
| C | 1.1631833 | -2.8030039 | 3.5000157 |
| C | -1.2008744 | -4.1795516 | 3.4570883 |
| H | -2.0839370 | -2.2177635 | 3.5216515 |
| C | 1.2021033 | -4.1796734 | 3.4561165 |
| H | 2.0852511 | -2.2177211 | 3.5202653 |
| C | 0.0005346 | -4.9285690 | 3.4008036 |
| H | -2.1761213 | -4.6591722 | 3.4556576 |
| H | 2.1772256 | -4.6594405 | 3.4540700 |
| C | -2.2964038 | 1.6429208 | 3.4806166 |
| N | -3.6181079 | 1.8360601 | 3.5605249 |
| C | -4.0757250 | 0.5641771 | 3.5758826 |
| C | 2.2965821 | 1.6437172 | 3.4812019 |
| N | 1.9841290 | 0.3076970 | 3.4490422 |
| C | 4.0761592 | 0.5653426 | 3.5757641 |
| N | 0.0000375 | 1.9246941 | 3.4425842 |
| N | 0.0006950 | -2.1047649 | 3.5003268 |
| N | -1.9835738 | 0.3069979 | 3.4486048 |
| N | -3.1243877 | -0.3902861 | 3.5109175 |
| N | 3.1251005 | -0.3893216 | 3.5109184 |
| N | 3.6182942 | 1.8371201 | 3.5608050 |
| N | 0.0004914 | -6.2853009 | 3.2848765 |
| H | -0.0007715 | 5.7351915 | 3.7762096 |
| C | 5.5235273 | 0.2220520 | 3.7807102 |
| C | -5.5227931 | 0.2206429 | 3.7819893 |
| F | 5.8452696 | 0.2509147 | 5.1100691 |
| F | 6.3425158 | 1.0992934 | 3.1600549 |
| F | 5.8076489 | -1.0228648 | 3.3338514 |
| F | -6.3424903 | 1.0980440 | 3.1624920 |
| F | -5.8432569 | 0.2490078 | 5.1116887 |
| F | -5.8072876 | -1.0241414 | 3.3349865 |
| C | 1.2577445 | -7.0199559 | 3.3095511 |
| H | 1.0587088 | -8.0746560 | 3.0943266 |
| H | 1.9482039 | -6.6395709 | 2.5424024 |
| H | 1.7573181 | -6.9533264 | 4.2906012 |
| C | -1.2566320 | -7.0200919 | 3.3122047 |
| H | -1.7531167 | -6.9556267 | 4.2949646 |
| H | -1.9495786 | -6.6382370 | 2.5481215 |
| H | -1.0584388 | -8.0743656 | 3.0942420 |
| C | -1.2577309 | 6.0423868 | 0.4012334 |
| H | -1.9496287 | 5.6628390 | 1.1674220 |
| H | -1.0591309 | 7.0972912 | 0.6159348 |
| H | -1.7554859 | 5.9749535 | -0.5807136 |
| C | 1.2564660 | 6.0424554 | 0.3986870 |
| H | 1.9507447 | 5.6614449 | 1.1619134 |
| H | 1.7512388 | 5.9770815 | -0.5849085 |
| H | 1.0587323 | 7.0969545 | 0.6160364 |

**1B dimer parallel**

| | | | |
|---|---|---|---|
| C | 1.2167161 | -4.5340567 | -0.0797564 |
| C | 1.1916146 | -3.1411646 | -0.1581612 |
| C | -1.1915934 | -3.1411704 | -0.1581729 |
| C | -1.2167411 | -4.5340402 | -0.0797668 |
| C | 0.0000082 | -5.2195427 | -0.0472145 |
| H | 2.1752589 | -5.0472099 | -0.0379999 |
| H | -2.1752560 | -5.0472024 | -0.0380194 |
| H | -0.0000048 | -6.3078557 | 0.0139030 |
| Pt | 0.0000068 | -0.5031374 | -0.2733571 |

| | | | |
|---|---|---|---|
| C | 1.1652503 | 2.2279438 | -0.1747196 |
| C | -1.1652257 | 2.2279234 | -0.1746464 |
| C | 1.1909261 | 3.6104295 | -0.0758150 |
| H | 2.0848476 | 1.6372075 | -0.2136014 |
| C | -1.1909190 | 3.6104470 | -0.0757332 |
| H | -2.0848210 | 1.6372180 | -0.2134770 |
| C | 0.0000363 | 4.3434361 | -0.0140209 |
| H | 2.1591387 | 4.1110277 | -0.0440152 |
| H | -2.1590939 | 4.1110279 | -0.0438741 |
| C | 2.2985357 | -2.2090364 | -0.1510406 |
| N | 3.6173000 | -2.3957533 | -0.0306532 |
| C | 4.0715795 | -1.1224803 | -0.0223767 |
| C | -2.2985425 | -2.2090217 | -0.1510609 |
| N | -1.9833460 | -0.8754278 | -0.2102049 |
| C | -4.0715560 | -1.1224690 | -0.0224135 |
| C | 0.0000237 | 5.8354050 | 0.1449646 |
| N | -0.0000068 | -2.4958023 | -0.2123069 |
| N | 0.0000053 | 1.5390277 | -0.2160893 |
| N | 1.9833544 | -0.8754352 | -0.2101896 |
| N | 3.1192690 | -0.1725155 | -0.1287729 |
| N | -3.1192469 | -0.1725021 | -0.1287999 |
| N | -3.6172978 | -2.3957296 | -0.0306888 |
| H | 0.8943738 | 6.2898574 | -0.3005805 |
| H | 0.0000477 | 6.1013287 | 1.2138253 |
| H | -0.8943319 | 6.2898641 | -0.3005463 |
| C | -5.5127925 | -0.7669814 | 0.2086190 |
| C | 5.5127967 | -0.7670142 | 0.2086729 |
| F | -5.7956171 | -0.7284158 | 1.5460616 |
| F | -6.3476527 | -1.6694230 | -0.3461408 |
| F | -5.8075124 | 0.4559162 | -0.2933815 |
| F | 5.7955906 | -0.7283584 | 1.5461221 |
| F | 5.8075652 | 0.4558442 | -0.2934050 |
| F | 6.3476621 | -1.6695089 | -0.3460006 |
| C | 1.2166674 | -4.5534443 | -3.6380479 |
| C | 1.1914858 | -3.1599047 | -3.5734537 |
| C | -1.1915354 | -3.1599011 | -3.5734633 |
| C | -1.2166878 | -4.5534523 | -3.6380560 |
| C | -0.0000219 | -5.2392663 | -3.6638306 |
| H | 2.1752734 | -5.0668700 | -3.6749337 |
| H | -2.1753066 | -5.0668774 | -3.6749505 |
| H | -0.0000127 | -6.3281269 | -3.7142644 |
| Pt | -0.0000298 | -0.5207115 | -3.4858646 |
| C | 1.1652674 | 2.2089724 | -3.6102268 |
| C | -1.1653273 | 2.2089679 | -3.6101455 |
| C | 1.1907719 | 3.5909189 | -3.7158694 |
| H | 2.0847872 | 1.6180309 | -3.5740687 |
| C | -1.1908524 | 3.5909210 | -3.7157870 |
| H | -2.0848462 | 1.6180224 | -3.5739400 |
| C | -0.0000334 | 4.3243021 | -3.7736369 |
| H | 2.1588860 | 4.0907900 | -3.7598764 |
| H | -2.1589550 | 4.0907807 | -3.7597237 |
| C | 2.2984469 | -2.2280893 | -3.5899147 |
| N | 3.6171660 | -2.4165561 | -3.7077567 |
| C | 4.0719769 | -1.1437412 | -3.7287791 |
| C | -2.2984800 | -2.2280985 | -3.5899327 |
| N | -1.9836326 | -0.8938534 | -3.5445997 |
| C | -4.0720221 | -1.1437693 | -3.7288174 |
| C | -0.0000474 | 5.8214741 | -3.8715708 |
| N | -0.0000074 | -2.5139224 | -3.5257080 |
| N | -0.0000227 | 1.5206462 | -3.5612657 |
| N | 1.9835876 | -0.8938427 | -3.5445835 |
| N | 3.1199374 | -0.1922540 | -3.6325373 |
| N | -3.1199890 | -0.1922649 | -3.6325613 |
| N | -3.6172072 | -2.4165731 | -3.7077912 |
| H | 0.8920275 | 6.1898544 | -4.3940659 |
| H | 0.0000476 | 6.2722242 | -2.8666321 |
| H | -0.8922082 | 6.1898582 | -4.3939058 |
| C | -5.5134433 | -0.7910578 | -3.9632620 |
| C | 5.5133993 | -0.7910256 | -3.9632066 |
| F | -5.8139499 | 0.4282843 | -3.4557472 |
| F | -6.3474826 | -1.6989290 | -3.4166139 |

```
F    -5.7912690   -0.7457883   -5.3016301
F     5.8138606    0.4283635   -3.4557723
F     5.7912677   -0.7458485   -5.3015675
F     6.3474403   -1.6988330   -3.4164641
```

**1B dimer 90° rotated**

```
C     4.1649101    0.6303907   -0.0525087
C     2.7742608    0.6575334   -0.1418165
C     2.6847569   -1.7181139   -0.1849218
C     4.0741696   -1.7978052   -0.1053118
C     4.8057284   -0.6096947   -0.0351594
H     4.7150813    1.5674651   -0.0050877
H     4.5536537   -2.7741530   -0.1071805
H     5.8925369   -0.6526193    0.0208757
Pt    0.0911203   -0.4287397   -0.3125124
C    -2.5968222    0.8408306   -0.3191234
C    -2.6890203   -1.4846458   -0.3694996
C    -3.9792806    0.9214900   -0.2970952
H    -1.9707810    1.7353462   -0.3027008
C    -4.0752698   -1.4566604   -0.3528333
H    -2.1369948   -2.4262791   -0.3951848
C    -4.7629727   -0.2388271   -0.2961172
H    -4.4433932    1.9078094   -0.2734232
H    -4.6157197   -2.4024541   -0.3828160
C     1.8854698    1.8014795   -0.1476546
N     2.1253545    3.1139432   -0.0561292
C     0.8705046    3.6189531   -0.0725530
C     1.7129723   -2.7921134   -0.2362794
N     0.3927492   -2.4280890   -0.3057783
C     0.5647121   -4.5310853   -0.2692318
C    -6.2580269   -0.1751943   -0.2102472
N     2.0813058   -0.5047778   -0.2049058
N    -1.9521381   -0.3495858   -0.3403991
N     0.5405601    1.5397422   -0.2147688
N    -0.1170822    2.7073588   -0.1674823
N    -0.3504150   -3.5439636   -0.3313961
N     1.8530908   -4.1219151   -0.2096199
H    -6.6593196    0.6204659   -0.8512962
H    -6.5659258    0.0499294    0.8231280
H    -6.7194410   -1.1251959   -0.5020145
C     0.1667231   -5.9751012   -0.1895199
C     0.5876358    5.0826994    0.0805770
F     0.2488825   -6.4465270    1.0846041
F     0.9722467   -6.7603787   -0.9514902
F    -1.1093724   -6.1632211   -0.6072427
F     0.7919392    5.5060690    1.3565210
F    -0.6972770    5.3763161   -0.2405320
F     1.3948673    5.8347876   -0.7155028
C     1.2850376   -4.5939958   -3.6004501
C     1.2056916   -3.2039037   -3.5330267
C    -1.1695317   -3.2921844   -3.5944073
C    -1.1426914   -4.6834955   -3.6724580
C     0.0970004   -5.3252704   -3.6744260
H     2.2610125   -5.0740654   -3.5863926
H    -2.0797706   -5.2333617   -3.7229640
H     0.1396205   -6.4125146   -3.7210561
Pt   -0.0826309   -0.6085888   -3.4338733
C     0.9722798    2.1729811   -3.3982751
C    -1.3532741    2.0781533   -3.4359228
C     0.9426984    3.5590439   -3.4225331
H     1.9145234    1.6219683   -3.3723904
C    -1.4355533    3.4604412   -3.4643269
H    -2.2473676    1.4514675   -3.4458977
C    -0.2763200    4.2452948   -3.4761301
H     1.8881091    4.1005633   -3.4007723
H    -2.4225012    3.9233141   -3.4841430
C     2.2799408   -2.2324750   -3.4816074
N     3.6096654   -2.3740001   -3.4991683
C     4.0197456   -1.0855315   -3.4480675
C    -2.3128527   -2.4026419   -3.6034719
N    -2.0505745   -1.0575486   -3.5428181
```

C    -4.1289753   -1.3869103   -3.6971936
C    -0.3418564    5.7397420   -3.5707821
N    -0.0072597   -2.5995114   -3.5276446
N    -0.1622564    1.4347495   -3.4176745
N     1.9166838   -0.9114085   -3.4251856
N     3.0331078   -0.1689663   -3.3993226
N    -3.2173405   -0.3994641   -3.6020538
N    -3.6248473   -2.6422165   -3.7025482
H    -0.5301374    6.0419062   -4.6133418
H     0.5959338    6.2055955   -3.2482132
H    -1.1611414    6.1416088   -2.9608235
C    -5.5912419   -1.1033703   -3.8622697
C     5.4648424   -0.6906477   -3.5241677
F    -5.8864025    0.1824414   -3.5457644
F    -6.3498878   -1.9084477   -3.0705743
F    -6.0044810   -1.3095451   -5.1412731
F     5.6530481    0.5893692   -3.1190168
F     5.9429129   -0.7873637   -4.7948552
F     6.2448077   -1.4896590   -2.7499285

**1B dimer 180° rotated**
C    -1.2165821    3.8570586    0.0026245
C    -1.1910313    2.4698413   -0.1516541
C     1.1910107    2.4698213   -0.1516558
C     1.2166055    3.8570103    0.0026666
C     0.0000160    4.5389743    0.0854397
H    -2.1754473    4.3658599    0.0774771
H     2.1754822    4.3657827    0.0775883
H     0.0000370    5.6192234    0.2334450
Pt   -0.0000316   -0.1678703   -0.3072819
C    -1.1658098   -2.8964945   -0.2614812
C     1.1656929   -2.8964982   -0.2614693
C    -1.1915777   -4.2817928   -0.2836973
H    -2.0853125   -2.3057549   -0.2577295
C     1.1914540   -4.2817936   -0.2836787
H     2.0851890   -2.3057494   -0.2577084
C    -0.0000637   -5.0165316   -0.3008745
H    -2.1598567   -4.7826075   -0.2992916
H     2.1597287   -4.7826188   -0.2992538
C    -2.2977445    1.5398115   -0.1940491
N    -3.6196077    1.7272457   -0.1050961
C    -4.0747419    0.4547511   -0.1227923
C     2.2977207    1.5397533   -0.1940964
N     1.9830318    0.2059625   -0.2624711
C     4.0747263    0.4546881   -0.1228951
C    -0.0000500   -6.5156926   -0.3194596
N    -0.0000105    1.8252817   -0.2264629
N    -0.0000604   -2.2085711   -0.2606790
N    -1.9830562    0.2060143   -0.2624128
N    -3.1213257   -0.4952230   -0.2161440
N     3.1212895   -0.4952687   -0.2162217
N     3.6195781    1.7272009   -0.1051751
H    -0.8934700   -6.9103472   -0.8199622
H    -0.0000483   -6.9107141    0.7090828
H     0.8933979   -6.9102927   -0.8199496
C     5.5198831    0.0972513    0.0783907
C    -5.5199012    0.0972831    0.0784754
F     5.8280429    0.0640833    1.4099659
F     6.3465548    0.9969269   -0.4951711
F     5.8000054   -1.1279095   -0.4231336
F    -5.8280729    0.0640455    1.4100454
F    -5.7999905   -1.1278573   -0.4231135
F    -6.3465719    0.9969799   -0.4950523
C     1.2162119   -4.8796419   -3.7313003
C     1.1908201   -3.4916081   -3.5852131
C    -1.1907944   -3.4916425   -3.5851297
C    -1.2161556   -4.8796954   -3.7311635
C     0.0000365   -5.5629052   -3.8048367
H     2.1752210   -5.3886601   -3.8029095
H    -2.1751509   -5.3887628   -3.8026174
H     0.0000572   -6.6454377   -3.9323884

| | | | |
|---|---|---|---|
| Pt | -0.0000048 | -0.8535377 | -3.4253560 |
| C | 1.1658530 | 1.8760928 | -3.4736486 |
| C | -1.1659308 | 1.8761076 | -3.4735538 |
| C | 1.1916562 | 3.2616613 | -3.4749544 |
| H | 2.0853565 | 1.2852937 | -3.4743429 |
| C | -1.1917183 | 3.2616828 | -3.4748619 |
| H | -2.0854542 | 1.2853396 | -3.4741912 |
| C | -0.0000237 | 3.9965288 | -3.4740955 |
| H | 2.1595709 | 3.7634050 | -3.4775683 |
| H | -2.1596302 | 3.7634271 | -3.4773760 |
| C | 2.2975479 | -2.5615552 | -3.5466111 |
| N | 3.6186376 | -2.7497069 | -3.6430823 |
| C | 4.0748361 | -1.4775498 | -3.6263815 |
| C | -2.2975360 | -2.5616117 | -3.5465070 |
| N | -1.9838107 | -1.2278016 | -3.4743557 |
| C | -4.0748096 | -1.4775906 | -3.6261985 |
| C | 0.0000123 | 5.4945397 | -3.4478558 |
| N | -0.0000001 | -2.8468552 | -3.5103418 |
| N | -0.0000443 | 1.1881023 | -3.4668190 |
| N | 1.9838325 | -1.2277528 | -3.4744384 |
| N | 3.1225730 | -0.5269975 | -3.5262478 |
| N | -3.1225395 | -0.5270411 | -3.5261184 |
| N | -3.6186255 | -2.7497491 | -3.6429097 |
| H | 0.8934104 | 5.9059817 | -3.9344855 |
| H | 0.0000663 | 5.8517009 | -2.4055978 |
| H | -0.8933749 | 5.9060801 | -3.9344289 |
| C | -5.5197780 | -1.1239251 | -3.8365236 |
| C | 5.5198108 | -1.1239519 | -3.8368217 |
| F | -5.8043002 | 0.1054052 | -3.3481646 |
| F | -6.3474472 | -2.0194871 | -3.2575606 |
| F | -5.8225764 | -1.1048257 | -5.1696082 |
| F | 5.8044443 | 0.1053998 | -3.3485814 |
| F | 5.8225060 | -1.1049718 | -5.1699339 |
| F | 6.3474874 | -2.0195132 | -3.2578563 |

**2A dimer parallel**

| | | | |
|---|---|---|---|
| C | 1.2204171 | -3.9813715 | 0.5887449 |
| C | 1.1916545 | -2.5924003 | 0.4805874 |
| C | -1.1919032 | -2.5866987 | 0.4839697 |
| C | -1.2271579 | -3.9754912 | 0.5918918 |
| C | -0.0049232 | -4.6537186 | 0.6395075 |
| H | 2.1709085 | -4.5083767 | 0.6220868 |
| H | -2.1801645 | -4.4977887 | 0.6278640 |
| Pt | 0.0059043 | 0.0383165 | 0.2794127 |
| C | 1.1707520 | 2.7536270 | -0.0876467 |
| C | -1.1497933 | 2.7558507 | -0.0946290 |
| C | 1.2116487 | 4.1119269 | -0.3266857 |
| H | 2.0928940 | 2.1731722 | -0.0011622 |
| C | -1.1868927 | 4.1139883 | -0.3345262 |
| H | -2.0736223 | 2.1775859 | -0.0117302 |
| C | 0.0133473 | 4.8564925 | -0.4495801 |
| H | 2.1884911 | 4.5754587 | -0.4374374 |
| H | -2.1622038 | 4.5792128 | -0.4514205 |
| C | 2.3020878 | -1.6677209 | 0.3819327 |
| N | 3.6247912 | -1.8605431 | 0.3113852 |
| C | 4.0757529 | -0.5936820 | 0.1703053 |
| C | -2.2981804 | -1.6566856 | 0.3900306 |
| N | -1.9736538 | -0.3271817 | 0.2934842 |
| C | -4.0674405 | -0.5733781 | 0.1893051 |
| N | 0.0013998 | -1.9442838 | 0.4384643 |
| N | 0.0095035 | 2.0626026 | 0.0281151 |
| N | 1.9837411 | -0.3366785 | 0.2846437 |
| N | 3.1194336 | 0.3572703 | 0.1505156 |
| N | -3.1064755 | 0.3726717 | 0.1666470 |
| N | -3.6222717 | -1.8427756 | 0.3256357 |
| Cl | -0.0089593 | -6.3803445 | 0.7556148 |
| N | 0.0151101 | 6.2066900 | -0.6666946 |
| C | -5.5037423 | -0.2149405 | -0.0654442 |
| C | 5.5130011 | -0.2450340 | -0.0933985 |
| F | -5.7571926 | -0.1637535 | -1.4098149 |
| F | -5.8071815 | 1.0091158 | 0.4346544 |

| | | | |
|---|---|---|---|
| F | -6.3527230 | -1.1159422 | 0.4637054 |
| F | 6.3593167 | -1.1440789 | 0.4438607 |
| F | 5.7622324 | -0.2116065 | -1.4390919 |
| F | 5.8245071 | 0.9831553 | 0.3907852 |
| C | 1.2259080 | -5.0429902 | 4.0533430 |
| C | 1.1889802 | -3.6607936 | 3.8790948 |
| C | -1.1875942 | -3.6661887 | 3.8809291 |
| C | -1.2180813 | -5.0484945 | 4.0553115 |
| C | 0.0054990 | -5.7190762 | 4.1417911 |
| H | 2.1795465 | -5.5601657 | 4.1235500 |
| H | -2.1693018 | -5.5698662 | 4.1269921 |
| Pt | -0.0056530 | -1.0342852 | 3.6012051 |
| C | 1.1504862 | 1.6936431 | 3.3734454 |
| C | -1.1747153 | 1.6877347 | 3.3647138 |
| C | 1.1860363 | 3.0598825 | 3.1965060 |
| H | 2.0741202 | 1.1151932 | 3.4478323 |
| C | -1.2157285 | 3.0537950 | 3.1871107 |
| H | -2.0959825 | 1.1048597 | 3.4335436 |
| C | -0.0162652 | 3.7983917 | 3.0752493 |
| H | 2.1605903 | 3.5386894 | 3.1492927 |
| H | -2.1922417 | 3.5276933 | 3.1320328 |
| C | 2.2953157 | -2.7345201 | 3.7934146 |
| N | 3.6167701 | -2.9215179 | 3.8657347 |
| C | 4.0744287 | -1.6552123 | 3.7566052 |
| C | -2.2982598 | -2.7450089 | 3.7969119 |
| N | -1.9889043 | -1.4150992 | 3.6523928 |
| C | -4.0824990 | -1.6741614 | 3.7625161 |
| N | -0.0008370 | -3.0165347 | 3.7948595 |
| N | -0.0106675 | 0.9969567 | 3.4506294 |
| N | 1.9794503 | -1.4061191 | 3.6488244 |
| N | 3.1222987 | -0.7077765 | 3.6243639 |
| N | -3.1351294 | -0.7221262 | 3.6295834 |
| N | -3.6186903 | -2.9383307 | 3.8706415 |
| Cl | 0.0094664 | -7.4344133 | 4.3681317 |
| N | -0.0187979 | 5.1439198 | 2.8556297 |
| C | -5.5378065 | -1.3276402 | 3.8905074 |
| C | 5.5277186 | -1.3000619 | 3.8840146 |
| F | -5.8056226 | -0.1162058 | 3.3475244 |
| F | -5.9087262 | -1.2671510 | 5.2052460 |
| F | -6.3281181 | -2.2474421 | 3.2993207 |
| F | 6.3242324 | -2.2223974 | 3.3057543 |
| F | 5.7906397 | -0.0936149 | 3.3273317 |
| F | 5.8950816 | -1.2218435 | 5.1989648 |
| C | 1.2341514 | 5.8814707 | 2.8363953 |
| H | 1.9309234 | 5.4437977 | 2.1058719 |
| H | 1.0337776 | 6.9160521 | 2.5396330 |
| H | 1.7277680 | 5.8914564 | 3.8228585 |
| C | -1.2750953 | 5.8756783 | 2.8305144 |
| H | -1.9647919 | 5.4379855 | 2.0932439 |
| H | -1.7754667 | 5.8787674 | 3.8136184 |
| H | -1.0772757 | 6.9125711 | 2.5400126 |
| C | 1.2661621 | 6.8882194 | -0.9743998 |
| H | 1.0810417 | 7.9664431 | -1.0209362 |
| H | 2.0130263 | 6.7085981 | -0.1890992 |
| H | 1.6935677 | 6.5653450 | -1.9393681 |
| C | -1.2326437 | 6.8902956 | -0.9832497 |
| H | -1.6533666 | 6.5681020 | -1.9514106 |
| H | -1.9853833 | 6.7115288 | -0.2033855 |
| H | -1.0452191 | 7.9682531 | -1.0280316 |

**2A dimer 90° rotated**

| | | | |
|---|---|---|---|
| C | 1.0891729 | -4.6376112 | 0.0730628 |
| C | 1.0638270 | -3.2496223 | 0.1835000 |
| C | -1.3092138 | -3.2251675 | 0.0801440 |
| C | -1.3509498 | -4.6113230 | -0.0360899 |
| C | -0.1355089 | -5.3021626 | -0.0492262 |
| H | 2.0369355 | -5.1698520 | 0.0739306 |
| H | -2.3041589 | -5.1254635 | -0.1287358 |
| Pt | -0.1036152 | -0.5979120 | 0.2905466 |
| C | 1.0886741 | 2.1219659 | 0.3624247 |
| C | -1.2322809 | 2.1626886 | 0.2988641 |

| | | | |
|---|---|---|---|
| C | 1.1536230 | 3.4964720 | 0.3666630 |
| H | 2.0004249 | 1.5227079 | 0.3869268 |
| C | -1.2469816 | 3.5401877 | 0.3057060 |
| H | -2.1630335 | 1.5939782 | 0.2601544 |
| C | -0.0329916 | 4.2715361 | 0.3330315 |
| H | 2.1355809 | 3.9597108 | 0.4050987 |
| H | -2.2117966 | 4.0396890 | 0.2809123 |
| C | 2.1821636 | -2.3320452 | 0.2657824 |
| N | 3.5038860 | -2.5361446 | 0.2608193 |
| C | 3.9728775 | -1.2693622 | 0.3292487 |
| C | -2.4108224 | -2.2845600 | 0.0503423 |
| N | -2.0859962 | -0.9534753 | 0.1338255 |
| C | -4.1713288 | -1.1815295 | -0.0924848 |
| N | -0.1183348 | -2.5865792 | 0.1849134 |
| N | -0.0843915 | 1.4403949 | 0.3192731 |
| N | 1.8791593 | -0.9953463 | 0.3309672 |
| N | 3.0296142 | -0.3074780 | 0.3758645 |
| N | -3.2160778 | -0.2404118 | 0.0439433 |
| N | -3.7296210 | -2.4596401 | -0.0970343 |
| Cl | -0.1482717 | -7.0191297 | -0.2420787 |
| N | -0.0015728 | 5.6300768 | 0.3240467 |
| C | -5.6022972 | -0.8092076 | -0.3308807 |
| C | 5.4346076 | -0.9388398 | 0.2661761 |
| F | -5.9072612 | -0.7889065 | -1.6572379 |
| F | -5.8847542 | 0.4275018 | 0.1580448 |
| F | -6.4584821 | -1.6894328 | 0.2515880 |
| F | 6.1748389 | -1.7959879 | 1.0107939 |
| F | 5.9074539 | -1.0115256 | -1.0096118 |
| F | 5.6764457 | 0.3196816 | 0.7108919 |
| C | 4.0744120 | 0.8853934 | 3.7781356 |
| C | 2.6899616 | 0.8418815 | 3.6435255 |
| C | 2.7159477 | -1.5322500 | 3.5708215 |
| C | 4.1023138 | -1.5559452 | 3.7004212 |
| C | 4.7651363 | -0.3296203 | 3.8158822 |
| H | 4.5871751 | 1.8397525 | 3.8662846 |
| H | 4.6344895 | -2.5035491 | 3.7194554 |
| Pt | 0.0660847 | -0.3668347 | 3.4096613 |
| C | -2.6922440 | 0.7635574 | 3.3362752 |
| C | -2.6550691 | -1.5582362 | 3.3259780 |
| C | -4.0694807 | 0.7804315 | 3.3127115 |
| H | -2.1224849 | 1.6940507 | 3.3620148 |
| C | -4.0297201 | -1.6209247 | 3.3072911 |
| H | -2.0571743 | -2.4712021 | 3.3279074 |
| C | -4.8029247 | -0.4324896 | 3.3053062 |
| H | -4.5673038 | 1.7464680 | 3.3096641 |
| H | -4.4944077 | -2.6028107 | 3.2854766 |
| C | 1.7489967 | 1.9435174 | 3.6468866 |
| N | 1.9215926 | 3.2639317 | 3.7824272 |
| C | 0.6436840 | 3.7051311 | 3.7529226 |
| C | 1.7994344 | -2.6515374 | 3.4910279 |
| N | 0.4637918 | -2.3496713 | 3.4022858 |
| C | 0.7376858 | -4.4429923 | 3.4369939 |
| N | 2.0529911 | -0.3503392 | 3.5446276 |
| N | -1.9717952 | -0.3855825 | 3.3508671 |
| N | 0.4194074 | 1.6173905 | 3.5463915 |
| N | -0.2950355 | 2.7481298 | 3.6123253 |
| N | -0.2234613 | -3.5006242 | 3.3632903 |
| N | 2.0033282 | -3.9730036 | 3.5172886 |
| Cl | 6.4792904 | -0.3137477 | 4.0319107 |
| N | -6.1615697 | -0.4607288 | 3.3002342 |
| C | 0.4060064 | -5.9034497 | 3.5164540 |
| C | 0.2663494 | 5.1379061 | 3.9709788 |
| F | -0.8409709 | -6.1535921 | 3.0442935 |
| F | 0.4459243 | -6.3541704 | 4.8016932 |
| F | 1.2820588 | -6.6552020 | 2.8064316 |
| F | 1.1561322 | 5.9897126 | 3.3968824 |
| F | -0.9615082 | 5.4141605 | 3.4567927 |
| F | 0.2215924 | 5.4548384 | 5.2939573 |
| C | -6.8653270 | -1.7288394 | 3.4513278 |
| H | -6.6397091 | -2.2038149 | 4.4207550 |
| H | -7.9425109 | -1.5444049 | 3.3986699 |

| | | | |
|---|---|---|---|
| H | -6.6023596 | -2.4266414 | 2.6448108 |
| C | -6.9198455 | 0.7823100 | 3.3186056 |
| H | -6.8037349 | 1.3239955 | 4.2730387 |
| H | -6.6055379 | 1.4411000 | 2.4974379 |
| H | -7.9805175 | 0.5531029 | 3.1764833 |
| C | 1.2716305 | 6.3292139 | 0.1956763 |
| H | 1.7655790 | 6.0989787 | -0.7631779 |
| H | 1.0897324 | 7.4071173 | 0.2417889 |
| H | 1.9519427 | 6.0666222 | 1.0169554 |
| C | -1.2418340 | 6.3904026 | 0.2616472 |
| H | -1.7576198 | 6.2624250 | -0.7056516 |
| H | -1.9233715 | 6.0882191 | 1.0686100 |
| H | -1.0141692 | 7.4524662 | 0.3956803 |

**2A dimer 180° rotated**

| | | | |
|---|---|---|---|
| C | 2.4061864 | -3.8392236 | 0.3237844 |
| C | 2.0022554 | -2.5055735 | 0.3776985 |
| C | -0.2875054 | -3.1408196 | 0.4451852 |
| C | 0.0523985 | -4.4931324 | 0.3984328 |
| C | 1.4096985 | -4.8181940 | 0.3355283 |
| H | 3.4619865 | -4.0858886 | 0.2453961 |
| H | -0.7275780 | -5.2503995 | 0.3824598 |
| Pt | 0.1512869 | -0.2754814 | 0.3681103 |
| C | 0.5263883 | 2.6698762 | 0.1509263 |
| C | -1.7062624 | 2.0230229 | 0.1013188 |
| C | 0.1804229 | 4.0029609 | 0.0981297 |
| H | 1.5742473 | 2.3647549 | 0.1840382 |
| C | -2.1267338 | 3.3321331 | 0.0358871 |
| H | -2.4301031 | 1.2052916 | 0.1179272 |
| C | -1.1829797 | 4.3895316 | 0.0601124 |
| H | 0.9841518 | 4.7344678 | 0.0953684 |
| H | -3.1955061 | 3.5202131 | -0.0174917 |
| C | 2.8198893 | -1.3165033 | 0.3270897 |
| N | 4.1443707 | -1.1563784 | 0.2376056 |
| C | 4.2479429 | 0.1889937 | 0.1561386 |
| C | -1.6027047 | -2.5428986 | 0.4524295 |
| N | -1.6574607 | -1.1718082 | 0.4072662 |
| C | -3.6065490 | -1.9827041 | 0.3721823 |
| N | 0.6836891 | -2.1952016 | 0.4366614 |
| N | -0.3965469 | 1.6760252 | 0.1731875 |
| N | 2.1622773 | -0.1111839 | 0.3029192 |
| N | 3.0774725 | 0.8596036 | 0.1912437 |
| N | -2.9435632 | -0.8080479 | 0.3548841 |
| N | -2.8260162 | -3.0842346 | 0.4320207 |
| Cl | 1.8726002 | -6.4895871 | 0.2425441 |
| N | -1.5686272 | 5.6937536 | 0.0605137 |
| C | -5.0992351 | -2.0494179 | 0.2255021 |
| C | 5.5631758 | 0.8815197 | -0.0590117 |
| F | -5.4467220 | -2.4032124 | -1.0472380 |
| F | -5.6731908 | -0.8513408 | 0.4782768 |
| F | -5.6444898 | -2.9679656 | 1.0537722 |
| F | 6.5215533 | 0.4035319 | 0.7685646 |
| F | 6.0103444 | 0.6880136 | -1.3354266 |
| F | 5.4597324 | 2.2148493 | 0.1310314 |
| C | -2.8800412 | 3.9778489 | 3.3808137 |
| C | -2.3733244 | 2.6777016 | 3.3809807 |
| C | -0.1394712 | 3.4893873 | 3.3298303 |
| C | -0.5827574 | 4.8108633 | 3.3474421 |
| C | -1.9615756 | 5.0306928 | 3.3682591 |
| H | -3.9535149 | 4.1447174 | 3.4240060 |
| H | 0.1354648 | 5.6266472 | 3.3636165 |
| Pt | -0.3493867 | 0.6020184 | 3.4438477 |
| C | -0.5135462 | -2.3516609 | 3.7035392 |
| C | 1.6746603 | -1.5681696 | 3.6194593 |
| C | -0.0857345 | -3.6609541 | 3.7172640 |
| H | -1.5784819 | -2.1118638 | 3.7371843 |
| C | 2.1762245 | -2.8510444 | 3.6305884 |
| H | 2.3439649 | -0.7060410 | 3.5813991 |
| C | 1.2974615 | -3.9636925 | 3.6509181 |
| H | -0.8409525 | -4.4408196 | 3.7683832 |
| H | 3.2556524 | -2.9746688 | 3.6116717 |

| | | | |
|---|---|---|---|
| C | -3.0948858 | 1.4297082 | 3.4796595 |
| N | -4.3968545 | 1.1687873 | 3.6412837 |
| C | -4.3863688 | -0.1744006 | 3.7868505 |
| C | 1.2182054 | 2.9982298 | 3.3400635 |
| N | 1.3854355 | 1.6373506 | 3.4009601 |
| C | 3.2618007 | 2.6064805 | 3.4105527 |
| N | -1.0337306 | 2.4709480 | 3.3454018 |
| N | 0.3439863 | -1.3031025 | 3.6334239 |
| N | -2.3407504 | 0.2828927 | 3.5263353 |
| N | -3.1673801 | -0.7502829 | 3.7231850 |
| N | 2.6985963 | 1.3812099 | 3.4487700 |
| N | 2.3919003 | 3.6393272 | 3.3421584 |
| Cl | -2.5499389 | 6.6644920 | 3.4010862 |
| N | 1.7516906 | -5.2443129 | 3.5968751 |
| C | 4.7437941 | 2.8075994 | 3.5407627 |
| C | -5.6160584 | -0.9623707 | 4.1390367 |
| F | 5.4261982 | 1.6788755 | 3.2466041 |
| F | 5.0756896 | 3.1572044 | 4.8193242 |
| F | 5.1860701 | 3.7989060 | 2.7338620 |
| F | -6.6797623 | -0.6102620 | 3.3831798 |
| F | -5.4112207 | -2.2906483 | 3.9958903 |
| F | -5.9658665 | -0.7450551 | 5.4424067 |
| C | 0.8156188 | -6.3552356 | 3.7000086 |
| H | 1.3602954 | -7.2935607 | 3.5620853 |
| H | 0.3154293 | -6.3801844 | 4.6822496 |
| H | 0.0438713 | -6.2943627 | 2.9182455 |
| C | 3.1833220 | -5.5133177 | 3.5893102 |
| H | 3.6787185 | -4.9758292 | 2.7676468 |
| H | 3.6629969 | -5.2183657 | 4.5375848 |
| H | 3.3429862 | -6.5844930 | 3.4361251 |
| C | -0.5702863 | 6.7535730 | 0.0677248 |
| H | -1.0714723 | 7.7129900 | 0.2270640 |
| H | 0.1477348 | 6.6064269 | 0.8867972 |
| H | -0.0099369 | 6.8029839 | -0.8812309 |
| C | -2.9735212 | 6.0386323 | -0.1143935 |
| H | -3.3554999 | 5.7083216 | -1.0942602 |
| H | -3.5941472 | 5.5867589 | 0.6732346 |
| H | -3.0835253 | 7.1246487 | -0.0457495 |

**2B dimer parallel**

| | | | |
|---|---|---|---|
| C | 1.2229694 | -4.5309144 | -0.0938233 |
| C | 1.1903405 | -3.1401179 | -0.1670895 |
| C | -1.1903888 | -3.1401225 | -0.1670939 |
| C | -1.2230392 | -4.5308911 | -0.0938281 |
| C | -0.0000302 | -5.2083605 | -0.0609675 |
| H | 2.1750497 | -5.0541958 | -0.0512220 |
| H | -2.1751194 | -5.0541784 | -0.0512318 |
| Pt | -0.0000149 | -0.5013408 | -0.2731705 |
| C | 1.1652304 | 2.2279053 | -0.1731628 |
| C | -1.1652431 | 2.2279095 | -0.1730598 |
| C | 1.1909598 | 3.6103458 | -0.0748905 |
| H | 2.0848640 | 1.6375078 | -0.2117448 |
| C | -1.1909483 | 3.6103501 | -0.0747942 |
| H | -2.0848704 | 1.6375165 | -0.2115870 |
| C | 0.0000031 | 4.3433677 | -0.0133834 |
| H | 2.1591431 | 4.1110116 | -0.0427993 |
| H | -2.1591317 | 4.1110164 | -0.0426126 |
| C | 2.2986773 | -2.2100338 | -0.1563837 |
| N | 3.6173860 | -2.3971744 | -0.0372348 |
| C | 4.0713373 | -1.1242019 | -0.0235657 |
| C | -2.2987298 | -2.2100058 | -0.1563909 |
| N | -1.9827979 | -0.8756376 | -0.2103765 |
| C | -4.0713823 | -1.1241626 | -0.0235619 |
| C | 0.0000130 | 5.8354001 | 0.1444943 |
| N | -0.0000233 | -2.4927144 | -0.2190000 |
| N | -0.0000036 | 1.5390316 | -0.2143783 |
| N | 1.9827556 | -0.8756575 | -0.2103784 |
| N | 3.1182838 | -0.1738817 | -0.1263613 |
| N | -3.1183030 | -0.1738615 | -0.1263577 |
| N | -3.6174246 | -2.3971546 | -0.0372416 |
| H | 0.8946488 | 6.2887839 | -0.3016569 |

| | | | |
|---|---|---|---|
| H | 0.0000166 | 6.1027448 | 1.2131200 |
| H | -0.8946232 | 6.2887882 | -0.3016518 |
| Cl | -0.0000434 | -6.9347326 | 0.0368733 |
| C | -5.5121401 | -0.7657203 | 0.2093021 |
| C | 5.5121046 | -0.7657485 | 0.2093135 |
| F | -5.7919334 | -0.7270845 | 1.5467255 |
| F | -6.3494000 | -1.6652310 | -0.3455066 |
| F | -5.8028544 | 0.4584258 | -0.2919487 |
| F | 5.7918572 | -0.7270238 | 1.5467426 |
| F | 6.3493674 | -1.6653078 | -0.3454114 |
| F | 5.8028416 | 0.4583578 | -0.2920162 |
| C | 1.2230596 | -4.5573570 | -3.6260308 |
| C | 1.1904601 | -3.1659230 | -3.5655656 |
| C | -1.1903124 | -3.1659535 | -3.5655675 |
| C | -1.2228950 | -4.5573597 | -3.6260396 |
| C | 0.0000870 | -5.2350832 | -3.6528579 |
| H | 2.1751395 | -5.0810033 | -3.6640685 |
| H | -2.1749717 | -5.0810321 | -3.6640854 |
| Pt | 0.0000591 | -0.5261395 | -3.4866898 |
| C | 1.1653210 | 2.2019389 | -3.6111511 |
| C | -1.1652393 | 2.2019151 | -3.6110544 |
| C | 1.1908801 | 3.5839062 | -3.7147714 |
| H | 2.0849053 | 1.6113661 | -3.5758877 |
| C | -1.1908246 | 3.5839167 | -3.7146546 |
| H | -2.0848099 | 1.6113601 | -3.5757099 |
| C | 0.0000422 | 4.3173839 | -3.7717180 |
| H | 2.1590152 | 4.0838727 | -3.7582771 |
| H | -2.1589412 | 4.0838603 | -3.7580780 |
| C | 2.2987858 | -2.2359820 | -3.5850997 |
| N | 3.6176934 | -2.4242048 | -3.7014114 |
| C | 4.0714750 | -1.1514280 | -3.7276294 |
| C | -2.2986594 | -2.2359961 | -3.5851009 |
| N | -1.9826223 | -0.9011443 | -3.5449418 |
| C | -4.0713514 | -1.1514642 | -3.7276316 |
| C | 0.0000229 | 5.8145255 | -3.8684829 |
| N | 0.0000716 | -2.5180264 | -3.5197485 |
| N | 0.0000499 | 1.5135512 | -3.5631756 |
| N | 1.9827378 | -0.9011197 | -3.5449443 |
| N | 3.1182193 | -0.2002242 | -3.6352258 |
| N | -3.1180918 | -0.2002599 | -3.6352159 |
| N | -3.6175512 | -2.4242455 | -3.7014198 |
| H | 0.8922144 | 6.1833990 | -4.3906046 |
| H | 0.0001193 | 6.2632443 | -2.8625967 |
| H | -0.8922488 | 6.1834049 | -4.3904455 |
| Cl | 0.0000957 | -6.9622653 | -3.7358757 |
| C | -5.5117157 | -0.7935451 | -3.9640346 |
| C | 5.5118403 | -0.7935050 | -3.9640278 |
| F | -5.8066127 | 0.4265266 | -3.4548699 |
| F | -6.3499217 | -1.6982787 | -3.4199260 |
| F | -5.7853685 | -0.7447571 | -5.3025000 |
| F | 5.8067194 | 0.4265996 | -3.4549370 |
| F | 6.3500470 | -1.6981986 | -3.4198406 |
| F | 5.7855254 | -0.7448059 | -5.3024909 |

**2B dimer 90° rotated**

| | | | |
|---|---|---|---|
| C | 4.1580881 | 0.7072450 | 0.0231372 |
| C | 2.7733502 | 0.7067359 | -0.1119474 |
| C | 2.7218960 | -1.6662230 | -0.1721362 |
| C | 4.1071707 | -1.7338332 | -0.0489462 |
| C | 4.8095237 | -0.5294146 | 0.0659818 |
| H | 4.7010599 | 1.6454595 | 0.1032426 |
| H | 4.6089556 | -2.6979385 | -0.0315570 |
| Pt | 0.1076860 | -0.4192338 | -0.3132437 |
| C | -2.5982125 | 0.8098331 | -0.2870505 |
| C | -2.6564246 | -1.5166320 | -0.3442526 |
| C | -3.9807647 | 0.8698634 | -0.2382120 |
| H | -1.9857946 | 1.7137630 | -0.2809697 |
| C | -4.0425941 | -1.5092022 | -0.3017484 |
| H | -2.0910607 | -2.4498588 | -0.3834783 |
| C | -4.7471648 | -0.3020618 | -0.2254635 |
| H | -4.4585300 | 1.8492116 | -0.2023282 |

| | | | |
|---|---|---|---|
| H | -4.5696314 | -2.4627698 | -0.3244416 |
| C | 1.8689323 | 1.8381191 | -0.1284856 |
| N | 2.0881223 | 3.1540828 | -0.0399860 |
| C | 0.8262778 | 3.6397618 | -0.0743306 |
| C | 1.7701863 | -2.7556342 | -0.2448113 |
| N | 0.4436769 | -2.4133278 | -0.3189192 |
| C | 0.6545821 | -4.5137560 | -0.3012324 |
| C | -6.2409695 | -0.2590346 | -0.1098142 |
| N | 2.0977577 | -0.4642135 | -0.1987095 |
| N | -1.9362057 | -0.3709036 | -0.3231278 |
| N | 0.5284660 | 1.5550605 | -0.2090695 |
| N | -0.1462002 | 2.7124828 | -0.1764488 |
| N | -0.2785296 | -3.5421633 | -0.3600787 |
| N | 1.9343053 | -4.0821088 | -0.2291515 |
| H | -6.6681325 | 0.5004974 | -0.7777430 |
| H | -6.5316318 | 0.0093002 | 0.9180661 |
| H | -6.6926963 | -1.2281116 | -0.3492611 |
| Cl | 6.5212962 | -0.5692404 | 0.2846708 |
| C | 0.2822485 | -5.9647846 | -0.2231764 |
| C | 0.5167046 | 5.0990949 | 0.0710344 |
| F | 0.2478220 | -6.3994071 | 1.0669929 |
| F | 1.1739923 | -6.7423660 | -0.8814374 |
| F | -0.9464049 | -6.1890599 | -0.7531567 |
| F | 0.6723750 | 5.5244820 | 1.3522470 |
| F | 1.3392458 | 5.8625578 | -0.6992540 |
| F | -0.7607136 | 5.3747991 | -0.2934987 |
| C | 1.2087302 | -4.6145111 | -3.6796455 |
| C | 1.1415032 | -3.2294302 | -3.5533736 |
| C | -1.2313204 | -3.2803636 | -3.6158669 |
| C | -1.2323334 | -4.6646769 | -3.7544841 |
| C | 0.0041005 | -5.3162750 | -3.7974588 |
| H | 2.1726029 | -5.1167617 | -3.6975596 |
| H | -2.1710341 | -5.2063378 | -3.8380932 |
| Pt | -0.1058067 | -0.6149133 | -3.4123058 |
| C | 0.9907607 | 2.1523871 | -3.3915722 |
| C | -1.3347421 | 2.0886879 | -3.4676143 |
| C | 0.9829912 | 3.5356056 | -3.4664505 |
| H | 1.9236713 | 1.5881419 | -3.3359806 |
| C | -1.3951212 | 3.4712753 | -3.5519924 |
| H | -2.2386540 | 1.4761136 | -3.4705003 |
| C | -0.2257308 | 4.2378027 | -3.5754814 |
| H | 1.9370844 | 4.0624381 | -3.4543424 |
| H | -2.3736729 | 3.9473106 | -3.6161979 |
| C | 2.2308428 | -2.2776269 | -3.4798819 |
| N | 3.5572586 | -2.4417266 | -3.4977193 |
| C | 3.9891231 | -1.1619953 | -3.4271512 |
| C | -2.3628408 | -2.3766769 | -3.6009062 |
| N | -2.0809482 | -1.0360841 | -3.5207496 |
| C | -4.1654508 | -1.3360907 | -3.6607007 |
| C | -0.2600031 | 5.7298156 | -3.7133769 |
| N | -0.0604569 | -2.6051341 | -3.5262453 |
| N | -0.1553630 | 1.4293529 | -3.4091665 |
| N | 1.8886321 | -0.9510108 | -3.4057805 |
| N | 3.0176473 | -0.2287380 | -3.3670423 |
| N | -3.2392477 | -0.3625581 | -3.5576150 |
| N | -3.6783040 | -2.5973038 | -3.6923664 |
| H | 0.4773753 | 6.0716249 | -4.4528977 |
| H | -0.0063850 | 6.2126959 | -2.7592047 |
| H | -1.2531246 | 6.0809765 | -4.0183387 |
| Cl | 0.0436924 | -7.0274415 | -4.0204792 |
| C | -5.6264787 | -1.0312997 | -3.8026070 |
| C | 5.4401825 | -0.7905345 | -3.5097729 |
| F | -5.9007293 | 0.2531492 | -3.4623548 |
| F | -6.3836762 | -1.8392245 | -3.0135637 |
| F | -6.0577962 | -1.2116823 | -5.0790330 |
| F | 5.6676653 | 0.4366709 | -2.9777281 |
| F | 6.2195569 | -1.6845810 | -2.8568084 |
| F | 5.8698861 | -0.7532581 | -4.8015524 |

**2B dimer 180° rotated**

| | | | |
|---|---|---|---|
| C | -1.2230172 | 3.3674339 | -0.2708473 |

| | | | |
|---|---|---|---|
| C | -1.1894711 | 1.9742997 | -0.2701962 |
| C | 1.1894514 | 1.9743874 | -0.2700942 |
| C | 1.2228931 | 3.3675025 | -0.2707471 |
| C | -0.0000805 | 4.0425600 | -0.2793843 |
| H | -2.1752703 | 3.8918909 | -0.2616654 |
| H | 2.1751185 | 3.8919963 | -0.2614487 |
| Pt | 0.0000572 | -0.6665676 | -0.2828651 |
| C | -1.1649868 | -3.3957204 | -0.1039394 |
| C | 1.1652366 | -3.3956536 | -0.1039118 |
| C | -1.1904809 | -4.7745989 | 0.0357971 |
| H | -2.0852975 | -2.8081758 | -0.1609257 |
| C | 1.1908185 | -4.7745353 | 0.0358462 |
| H | 2.0854603 | -2.8079830 | -0.1608282 |
| C | 0.0001849 | -5.5068892 | 0.1183061 |
| H | -2.1586263 | -5.2741276 | 0.0798459 |
| H | 2.1589970 | -5.2739925 | 0.0799210 |
| C | -2.2969310 | 1.0479032 | -0.2391834 |
| N | -3.6163893 | 1.2443994 | -0.1445973 |
| C | -4.0754755 | -0.0244886 | -0.1004324 |
| C | 2.2969672 | 1.0480580 | -0.2390008 |
| N | 1.9846739 | -0.2879031 | -0.2458068 |
| C | 4.0755245 | -0.0243031 | -0.1001692 |
| C | 0.0002125 | -6.9936647 | 0.3128817 |
| N | 0.0000110 | 1.3261571 | -0.2835621 |
| N | 0.0001073 | -2.7068188 | -0.1688368 |
| N | -1.9846081 | -0.2880734 | -0.2459862 |
| N | -3.1246427 | -0.9825149 | -0.1617483 |
| N | 3.1247000 | -0.9823340 | -0.1615779 |
| N | 3.6164284 | 1.2445774 | -0.1443299 |
| H | -0.8919645 | -7.4560286 | -0.1273042 |
| H | 0.0002533 | -7.2328374 | 1.3881739 |
| H | 0.8923946 | -7.4559658 | -0.1274000 |
| Cl | -0.0001055 | 5.7759661 | -0.2968987 |
| C | 5.5179543 | -0.3764657 | 0.1315121 |
| C | -5.5179241 | -0.3766638 | 0.1311448 |
| F | 5.7415986 | -0.6547101 | 1.4515470 |
| F | 6.3388975 | 0.6360035 | -0.2080672 |
| F | 5.8775716 | -1.4792090 | -0.5677558 |
| F | -5.7416084 | -0.6551820 | 1.4511188 |
| F | -6.3388880 | 0.6358526 | -0.2082313 |
| F | -5.8774854 | -1.4792597 | -0.5683873 |
| C | 1.2225550 | -4.3889535 | -3.4532199 |
| C | 1.1891735 | -2.9960292 | -3.4588782 |
| C | -1.1893168 | -2.9959850 | -3.4589755 |
| C | -1.2227478 | -4.3889062 | -3.4532829 |
| C | -0.0001106 | -5.0645208 | -3.4384394 |
| H | 2.1748881 | -4.9132307 | -3.4574322 |
| H | -2.1750983 | -4.9131515 | -3.4576349 |
| Pt | -0.0000283 | -0.3549718 | -3.4409365 |
| C | 1.1651737 | 2.3724325 | -3.6388931 |
| C | -1.1651365 | 2.3724387 | -3.6390631 |
| C | 1.1907708 | 3.7463018 | -3.8215463 |
| H | 2.0854321 | 1.7860467 | -3.5709944 |
| C | -1.1906852 | 3.7463232 | -3.8217449 |
| H | -2.0854626 | 1.7861287 | -3.5712999 |
| C | 0.0000523 | 4.4754586 | -3.9292915 |
| H | 2.1590321 | 4.2425556 | -3.8919105 |
| H | -2.1589140 | 4.2426335 | -3.8922518 |
| C | 2.2966886 | -2.0698501 | -3.4886787 |
| N | 3.6159057 | -2.2670156 | -3.5841003 |
| C | 4.0757843 | -0.9983606 | -3.6266569 |
| C | -2.2968247 | -2.0698266 | -3.4888214 |
| N | -1.9851363 | -0.7337568 | -3.4791854 |
| C | -4.0758707 | -0.9982794 | -3.6269900 |
| C | 0.0001207 | 5.9612444 | -4.1336101 |
| N | -0.0000555 | -2.3476400 | -3.4455251 |
| N | 0.0000010 | 1.6853365 | -3.5573044 |
| N | 1.9850467 | -0.7337862 | -3.4790444 |
| N | 3.1255887 | -0.0397996 | -3.5631360 |
| N | -3.1256519 | -0.0397500 | -3.5633555 |
| N | -3.6160256 | -2.2669609 | -3.5844455 |

| | | | |
|---|---|---|---|
| H | 0.8926415 | 6.2899200 | -4.6812499 |
| H | 0.0005744 | 6.4769323 | -3.1611491 |
| H | -0.8927721 | 6.2900563 | -4.6805603 |
| Cl | -0.0001775 | -6.7960741 | -3.3893270 |
| C | -5.5184563 | -0.6481495 | -3.8609381 |
| C | 5.5183953 | -0.6482616 | -3.8605047 |
| F | -5.8819548 | 0.4530666 | -3.1613931 |
| F | -6.3379902 | -1.6627334 | -3.5240337 |
| F | -5.7398729 | -0.3690189 | -5.1812095 |
| F | 5.8817195 | 0.4530788 | -3.1610850 |
| F | 6.3379553 | -1.6627495 | -3.5233504 |
| F | 5.7399857 | -0.3693697 | -5.1808004 |

**3A dimer parallel**

| | | | |
|---|---|---|---|
| C | 1.2233447 | -3.9507456 | 0.5088492 |
| C | 1.1997537 | -2.5589629 | 0.4236181 |
| C | -1.1872975 | -2.5577716 | 0.4333804 |
| C | -1.2117402 | -3.9494657 | 0.5190133 |
| C | 0.0056414 | -4.6347360 | 0.5567864 |
| H | 2.1812367 | -4.4659702 | 0.5399363 |
| H | -2.1699097 | -4.4636725 | 0.5577938 |
| Pt | 0.0069831 | 0.0698281 | 0.3045786 |
| C | 1.1669378 | 2.7987534 | 0.0241318 |
| C | -1.1534814 | 2.7984153 | 0.0264167 |
| C | 1.2052392 | 4.1640838 | -0.1726401 |
| H | 2.0904669 | 2.2188076 | 0.0932453 |
| C | -1.1925954 | 4.1636304 | -0.1705734 |
| H | -2.0766996 | 2.2183540 | 0.0981046 |
| C | 0.0060504 | 4.9110377 | -0.2657710 |
| H | 2.1811475 | 4.6319036 | -0.2718067 |
| H | -2.1688594 | 4.6309689 | -0.2681171 |
| C | 2.3031419 | -1.6226886 | 0.3674135 |
| N | 3.6248657 | -1.7820801 | 0.3451848 |
| C | -2.2902131 | -1.6204000 | 0.3863501 |
| N | -1.9756504 | -0.2941478 | 0.3275358 |
| N | 0.0064119 | -1.9150884 | 0.3955872 |
| N | 0.0069414 | 2.1023319 | 0.1231881 |
| N | 1.9892780 | -0.2960726 | 0.3116950 |
| N | 3.1461850 | 0.3678726 | 0.2596101 |
| N | -3.1324623 | 0.3708619 | 0.2857767 |
| N | -3.6122195 | -1.7786093 | 0.3762217 |
| N | 0.0057023 | 6.2691422 | -0.4348986 |
| H | 0.0053293 | -5.7220729 | 0.6269668 |
| N | -4.1130269 | -0.5248029 | 0.3134482 |
| N | 4.1261746 | -0.5287366 | 0.2782342 |
| C | 1.2104552 | -5.0618856 | 3.9414992 |
| C | 1.1855957 | -3.6727254 | 3.8126023 |
| C | -1.1986652 | -3.6723330 | 3.8216064 |
| C | -1.2230052 | -5.0615093 | 3.9507192 |
| C | -0.0061614 | -5.7460449 | 4.0088618 |
| H | 2.1688370 | -5.5745376 | 3.9940318 |
| H | -2.1811624 | -5.5737657 | 4.0107484 |
| Pt | -0.0068555 | -1.0429204 | 3.6147008 |
| C | 1.1551062 | 1.6884947 | 3.4481209 |
| C | -1.1702560 | 1.6877755 | 3.4498922 |
| C | 1.1928353 | 3.0590980 | 3.3082655 |
| H | 2.0784648 | 1.1079644 | 3.5012168 |
| C | -1.2087750 | 3.0583580 | 3.3098530 |
| H | -2.0933407 | 1.1069870 | 3.5051277 |
| C | -0.0082643 | 3.8029488 | 3.2129874 |
| H | 2.1682222 | 3.5368972 | 3.2691875 |
| H | -2.1845168 | 3.5354471 | 3.2713429 |
| C | 2.2887314 | -2.7393476 | 3.7469898 |
| N | 3.6097105 | -2.8990231 | 3.7958877 |
| C | -2.3019055 | -2.7385567 | 3.7649052 |
| N | -1.9915580 | -1.4130273 | 3.6616443 |
| N | -0.0066294 | -3.0272941 | 3.7533383 |
| N | -0.0073557 | 0.9918795 | 3.5124303 |
| N | 1.9781013 | -1.4137279 | 3.6458574 |
| N | 3.1378376 | -0.7518316 | 3.6327353 |
| N | -3.1510159 | -0.7506355 | 3.6582779 |

| | | | |
|---|---|---|---|
| N | -3.6224860 | -2.8976361 | 3.8247919 |
| N | -0.0092221 | 5.1541810 | 3.0295020 |
| H | -0.0059581 | -6.8310347 | 4.1153275 |
| N | -4.1273375 | -1.6461892 | 3.7566242 |
| N | 4.1145460 | -1.6477522 | 3.7232867 |
| C | -1.2643436 | 5.8879592 | 3.0277255 |
| H | -1.7641108 | 5.8614759 | 4.0107320 |
| H | -1.0654464 | 6.9330259 | 2.7691588 |
| H | -1.9556199 | 5.4740050 | 2.2781616 |
| C | 1.2448274 | 5.8895499 | 3.0178656 |
| H | 1.7455836 | 5.8769233 | 4.0006835 |
| H | 1.9355630 | 5.4665279 | 2.2731458 |
| H | 1.0445877 | 6.9307604 | 2.7449427 |
| C | 1.2530432 | 6.9609940 | -0.7342927 |
| H | 1.0706542 | 8.0408238 | -0.7263339 |
| H | 2.0137276 | 6.7434237 | 0.0274196 |
| H | 1.6614163 | 6.6829759 | -1.7214303 |
| C | -1.2424265 | 6.9611196 | -0.7305647 |
| H | -1.6506956 | 6.6877768 | -1.7190920 |
| H | -2.0026340 | 6.7387429 | 0.0301305 |
| H | -1.0612691 | 8.0411307 | -0.7167965 |

**3A dimer 90° rotated**

| | | | |
|---|---|---|---|
| C | 1.0711086 | -4.6891919 | 0.3441200 |
| C | 1.0316880 | -3.2979867 | 0.2884743 |
| C | -1.3473007 | -3.3218949 | 0.2411377 |
| C | -1.3584467 | -4.7141750 | 0.2950600 |
| C | -0.1372383 | -5.3890219 | 0.3517316 |
| H | 2.0325197 | -5.1938245 | 0.4062615 |
| H | -2.3101594 | -5.2414614 | 0.2986151 |
| Pt | -0.1834930 | -0.6713299 | 0.2264974 |
| C | 0.9620525 | 2.0706803 | 0.2559624 |
| C | -1.3589098 | 2.0595903 | 0.3371460 |
| C | 1.0038384 | 3.4323148 | 0.4415836 |
| H | 1.8840065 | 1.4972819 | 0.1551136 |
| C | -1.3954634 | 3.4207958 | 0.5397072 |
| H | -2.2800172 | 1.4797247 | 0.2734847 |
| C | -0.1928628 | 4.1657007 | 0.6427927 |
| H | 1.9778920 | 3.9097256 | 0.4937914 |
| H | -2.3674194 | 3.8939063 | 0.6501046 |
| C | 2.1291658 | -2.3505745 | 0.2827232 |
| N | 3.4521513 | -2.5009659 | 0.2967919 |
| C | -2.4617064 | -2.3968579 | 0.1613779 |
| N | -2.1659813 | -1.0644954 | 0.1342137 |
| N | -0.1646303 | -2.6601532 | 0.2456860 |
| N | -0.1987200 | 1.3643700 | 0.2257120 |
| N | 1.8068085 | -1.0251466 | 0.2625239 |
| N | 2.9616924 | -0.3509715 | 0.2675076 |
| N | -3.3293088 | -0.4168148 | 0.0238375 |
| N | -3.7781745 | -2.5761231 | 0.0722593 |
| N | -0.1842931 | 5.4917271 | 0.9322045 |
| H | -0.1259761 | -6.4764175 | 0.4149484 |
| N | -4.2935682 | -1.3272201 | -0.0138980 |
| N | 3.9456023 | -1.2403120 | 0.2852252 |
| C | 4.1954914 | 0.9050759 | 3.4608247 |
| C | 2.8024379 | 0.8940677 | 3.4855511 |
| C | 2.7802106 | -1.4854160 | 3.4868288 |
| C | 4.1722789 | -1.5249572 | 3.4616015 |
| C | 4.8717354 | -0.3165363 | 3.4435913 |
| H | 4.7224916 | 1.8568519 | 3.4487754 |
| H | 4.6783839 | -2.4870619 | 3.4303068 |
| Pt | 0.1525563 | -0.2710916 | 3.4676466 |
| C | -2.5753507 | 0.9009676 | 3.2780732 |
| C | -2.5886979 | -1.4178896 | 3.4066930 |
| C | -3.9327337 | 0.9334999 | 3.0506459 |
| H | -1.9963013 | 1.8231409 | 3.3330130 |
| C | -3.9468713 | -1.4633268 | 3.1979149 |
| H | -2.0175271 | -2.3375693 | 3.5363783 |
| C | -4.6761706 | -0.2710158 | 2.9595437 |
| H | -4.4030911 | 1.9031114 | 2.9115388 |
| H | -4.4237783 | -2.4381813 | 3.1568528 |

| | | | |
|---|---|---|---|
| C | 1.8755856 | 2.0091907 | 3.5238751 |
| N | 2.0524149 | 3.3272198 | 3.5918686 |
| C | 1.8331319 | -2.5831783 | 3.4955872 |
| N | 0.5075106 | -2.2613694 | 3.4802121 |
| N | 2.1413088 | -0.2889265 | 3.4910886 |
| N | -1.8827338 | -0.2567154 | 3.4262122 |
| N | 0.5430726 | 1.7130284 | 3.5288248 |
| N | -0.1072562 | 2.8776344 | 3.6036729 |
| N | -0.1662120 | -3.4164600 | 3.4856822 |
| N | 1.9840464 | -3.9060311 | 3.5136002 |
| N | -5.9968992 | -0.2858360 | 2.6472599 |
| H | 5.9602090 | -0.3283389 | 3.4035330 |
| N | 0.7235370 | -4.3999801 | 3.5085668 |
| N | 0.8017516 | 3.8431300 | 3.6422580 |
| C | -1.4200669 | 6.1469398 | 1.3383749 |
| H | -1.2178734 | 7.2080806 | 1.5138447 |
| H | -2.1841478 | 6.0747301 | 0.5499799 |
| H | -1.8219640 | 5.7088282 | 2.2667268 |
| C | 1.0830613 | 6.1740865 | 1.1777541 |
| H | 1.5876579 | 5.7673223 | 2.0678007 |
| H | 1.7476441 | 6.0832820 | 0.3067120 |
| H | 0.8836952 | 7.2384744 | 1.3381270 |
| C | -6.6759795 | -1.5580487 | 2.4181453 |
| H | -6.6018660 | -2.2027575 | 3.3055654 |
| H | -7.7369975 | -1.3617386 | 2.2336220 |
| H | -6.2537280 | -2.0832116 | 1.5472281 |
| C | -6.6449945 | 0.9419074 | 2.2065504 |
| H | -6.5896544 | 1.7196140 | 2.9830159 |
| H | -6.1884670 | 1.3283087 | 1.2804876 |
| H | -7.7020871 | 0.7352587 | 2.0129076 |

**3A dimer 180° rotated**

| | | | |
|---|---|---|---|
| C | 1.2163501 | -4.9656108 | 0.1034020 |
| C | 1.1915698 | -3.5739818 | 0.2038660 |
| C | -1.1913318 | -3.5741383 | 0.2035293 |
| C | -1.2159367 | -4.9657871 | 0.1030535 |
| C | 0.0002689 | -5.6518887 | 0.0511594 |
| H | 2.1758013 | -5.4756210 | 0.0452136 |
| H | -2.1753095 | -5.4758774 | 0.0446349 |
| Pt | -0.0000147 | -0.9359933 | 0.2695602 |
| C | 1.1625692 | 1.7985977 | 0.2040695 |
| C | -1.1631583 | 1.7979635 | 0.2022283 |
| C | 1.2009108 | 3.1750184 | 0.2532223 |
| H | 2.0859456 | 1.2161367 | 0.1872210 |
| C | -1.2023662 | 3.1743050 | 0.2513534 |
| H | -2.0862469 | 1.2150981 | 0.1839447 |
| C | -0.0010031 | 3.9234066 | 0.3068207 |
| H | 2.1765105 | 3.6537589 | 0.2601173 |
| H | -2.1782195 | 3.6524832 | 0.2568582 |
| C | 2.2937658 | -2.6391691 | 0.2406129 |
| N | 3.6156181 | -2.8027665 | 0.2351163 |
| C | -2.2936717 | -2.6394807 | 0.2400835 |
| N | -1.9847609 | -1.3096276 | 0.2528335 |
| N | 0.0000727 | -2.9265364 | 0.2448356 |
| N | -0.0001057 | 1.0993684 | 0.1979601 |
| N | 1.9847031 | -1.3093456 | 0.2533742 |
| N | 3.1440260 | -0.6493831 | 0.2510357 |
| N | -3.1441907 | -0.6498361 | 0.2502817 |
| N | -3.6154766 | -2.8032722 | 0.2344428 |
| N | -0.0015091 | 5.2804790 | 0.4199554 |
| H | 0.0003377 | -6.7375922 | -0.0471972 |
| N | -4.1213851 | -1.5493324 | 0.2401470 |
| N | 4.1213440 | -1.5487463 | 0.2409759 |
| C | -1.2174887 | 3.9897247 | 3.6040869 |
| C | -1.1925886 | 2.5985300 | 3.4976636 |
| C | 1.1903132 | 2.5988223 | 3.4982887 |
| C | 1.2148608 | 3.9900153 | 3.6047601 |
| C | -0.0014159 | 4.6758056 | 3.6598990 |
| H | -2.1769327 | 4.4994516 | 3.6645585 |
| H | 2.1741689 | 4.4999562 | 3.6657246 |
| Pt | -0.0008394 | -0.0392126 | 3.4269136 |

| | | | |
|---|---|---|---|
| C | -1.1632880 | -2.7738003 | 3.4948779 |
| C | 1.1624578 | -2.7730049 | 3.4965433 |
| C | -1.2015375 | -4.1502787 | 3.4478460 |
| H | -2.0867411 | -2.1914335 | 3.5104476 |
| C | 1.2017496 | -4.1494194 | 3.4495394 |
| H | 2.0855426 | -2.1901239 | 3.5134025 |
| C | 0.0004290 | -4.8986834 | 3.3952748 |
| H | -2.1771040 | -4.6290546 | 3.4411953 |
| H | 2.1776343 | -4.6274843 | 3.4441369 |
| C | -2.2947219 | 1.6638215 | 3.4563746 |
| N | -3.6165632 | 1.8274396 | 3.4569502 |
| C | 2.2926973 | 1.6644026 | 3.4573746 |
| N | 1.9838979 | 0.3345430 | 3.4424465 |
| N | -0.0010294 | 1.9512801 | 3.4547280 |
| N | -0.0006416 | -2.0744990 | 3.4996647 |
| N | -1.9856027 | 0.3340437 | 3.4416262 |
| N | -3.1449551 | -0.3259222 | 3.4376953 |
| N | 3.1434396 | -0.3251072 | 3.4388082 |
| N | 3.6144975 | 1.8283596 | 3.4583083 |
| N | 0.0009939 | -6.2558004 | 3.2836188 |
| H | -0.0015581 | 5.7609344 | 3.7642058 |
| N | 4.1205385 | 0.5744711 | 3.4474469 |
| N | -4.1222809 | 0.5734198 | 3.4460099 |
| C | 1.2553923 | 6.0153746 | 0.3898228 |
| H | 1.0565910 | 7.0709090 | 0.6012936 |
| H | 1.9476475 | 5.6380241 | 1.1567742 |
| H | 1.7532531 | 5.9452041 | -0.5918578 |
| C | -1.2586359 | 6.0149368 | 0.3860707 |
| H | -1.7527161 | 5.9457180 | -0.5976885 |
| H | -1.9532656 | 5.6359598 | 1.1500120 |
| H | -1.0611176 | 7.0701835 | 0.6002919 |
| C | -1.2560635 | -6.9905354 | 3.3114690 |
| H | -1.9458493 | -6.6150590 | 2.5412845 |
| H | -1.0566260 | -8.0466413 | 3.1034912 |
| H | -1.7570496 | -6.9177045 | 4.2913269 |
| C | 1.2583268 | -6.9900277 | 3.3153597 |
| H | 1.9506490 | -6.6127664 | 2.5483961 |
| H | 1.7553038 | -6.9182947 | 4.2974390 |
| H | 1.0602549 | -8.0458331 | 3.1043935 |

**3B dimer parallel**

| | | | |
|---|---|---|---|
| C | 1.2173648 | -4.5194423 | -0.0651590 |
| C | 1.1933745 | -3.1269793 | -0.1426443 |
| C | -1.1933603 | -3.1269751 | -0.1426887 |
| C | -1.2173808 | -4.5194477 | -0.0652036 |
| C | 0.0000158 | -5.2044060 | -0.0311551 |
| H | 2.1750133 | -5.0345004 | -0.0254225 |
| H | -2.1750019 | -5.0345059 | -0.0255026 |
| Pt | 0.0000021 | -0.4943770 | -0.2602333 |
| C | 1.1652681 | 2.2370240 | -0.1788229 |
| C | -1.1652400 | 2.2370263 | -0.1787257 |
| C | 1.1908203 | 3.6203059 | -0.0941503 |
| H | 2.0855671 | 1.6485049 | -0.2161505 |
| C | -1.1908541 | 3.6202893 | -0.0940488 |
| H | -2.0855838 | 1.6484949 | -0.2159972 |
| C | 0.0000184 | 4.3537940 | -0.0409996 |
| H | 2.1591075 | 4.1210647 | -0.0707359 |
| H | -2.1591024 | 4.1210571 | -0.0705523 |
| C | 2.2962904 | -2.1898742 | -0.1514731 |
| N | 3.6157932 | -2.3474769 | -0.0693738 |
| C | -2.2962917 | -2.1898868 | -0.1515554 |
| N | -1.9846693 | -0.8624651 | -0.2083035 |
| N | -0.0000101 | -2.4832896 | -0.1934763 |
| N | -0.0000267 | 1.5469584 | -0.2142672 |
| N | 1.9846674 | -0.8624596 | -0.2082332 |
| N | 3.1400268 | -0.1971097 | -0.1612643 |
| N | -3.1400339 | -0.1971198 | -0.1613674 |
| N | -3.6157955 | -2.3474868 | -0.0694972 |
| N | -4.1177995 | -1.0930387 | -0.0777955 |
| N | 4.1177941 | -1.0930248 | -0.0776607 |
| C | 0.0000030 | 5.8480185 | 0.0949313 |

| | | | |
|---|---|---|---|
| H | 0.0001215 | 6.1319163 | 1.1592003 |
| H | 0.8942765 | 6.2945554 | -0.3589480 |
| H | -0.8943620 | 6.2945489 | -0.3587581 |
| H | 0.0000033 | -6.2926915 | 0.0314349 |
| C | 1.2174479 | -4.5361398 | -3.6546678 |
| C | 1.1934370 | -3.1430644 | -3.5879486 |
| C | -1.1932729 | -3.1430696 | -3.5879932 |
| C | -1.2172750 | -4.5361418 | -3.6547129 |
| C | 0.0000854 | -5.2213309 | -3.6835402 |
| H | 2.1750696 | -5.0514883 | -3.6907166 |
| H | -2.1748974 | -5.0514951 | -3.6907974 |
| Pt | 0.0000782 | -0.5094442 | -3.4943881 |
| C | 1.1653818 | 2.2208937 | -3.6038456 |
| C | -1.1652093 | 2.2208910 | -3.6037529 |
| C | 1.1907623 | 3.6033342 | -3.7007364 |
| H | 2.0857537 | 1.6324491 | -3.5670580 |
| C | -1.1906641 | 3.6033234 | -3.7006380 |
| H | -2.0856182 | 1.6324391 | -3.5669106 |
| C | 0.0000821 | 4.3369643 | -3.7536322 |
| H | 2.1590612 | 4.1033212 | -3.7377696 |
| H | -2.1589275 | 4.1033147 | -3.7375943 |
| C | 2.2963477 | -2.2059388 | -3.5869885 |
| N | 3.6159168 | -2.3643013 | -3.6667822 |
| C | -2.2961888 | -2.2059466 | -3.5870717 |
| N | -1.9845594 | -0.8780480 | -3.5425135 |
| N | 0.0000793 | -2.4989884 | -3.5421415 |
| N | 0.0000527 | 1.5313563 | -3.5589761 |
| N | 1.9847140 | -0.8780413 | -3.5424430 |
| N | 3.1401309 | -0.2131957 | -3.5947325 |
| N | -3.1399772 | -0.2132051 | -3.5948365 |
| N | -3.6157514 | -2.3643124 | -3.6669065 |
| N | -4.1177801 | -1.1098467 | -3.6696827 |
| N | 4.1179398 | -1.1098329 | -3.6695466 |
| C | 0.0000563 | 5.8348636 | -3.8365395 |
| H | 0.0003137 | 6.2736765 | -2.8264538 |
| H | 0.8924068 | 6.2087989 | -4.3545995 |
| H | -0.8925210 | 6.2088267 | -4.3541714 |
| H | 0.0000894 | -6.3100718 | -3.7382869 |

**3B dimer 90° rotated**

| | | | |
|---|---|---|---|
| C | 4.2305765 | 0.8212708 | -0.3468843 |
| C | 2.8378969 | 0.8302201 | -0.3052955 |
| C | 2.7816790 | -1.5487794 | -0.2748227 |
| C | 4.1725197 | -1.6085049 | -0.3166496 |
| C | 4.8884394 | -0.4100894 | -0.3572357 |
| H | 4.7703961 | 1.7651647 | -0.3814234 |
| H | 4.6651986 | -2.5777112 | -0.3435978 |
| Pt | 0.1705137 | -0.2966530 | -0.2684132 |
| C | -2.5380521 | 0.9258869 | -0.3443883 |
| C | -2.5870207 | -1.3998220 | -0.2441540 |
| C | -3.9184593 | 0.9740713 | -0.4619024 |
| H | -1.9340774 | 1.8342556 | -0.3252745 |
| C | -3.9660588 | -1.4039246 | -0.3525990 |
| H | -2.0196466 | -2.3278694 | -0.1643125 |
| C | -4.6731993 | -0.2029464 | -0.4952158 |
| H | -4.4017558 | 1.9473725 | -0.5493101 |
| H | -4.4854092 | -2.3615650 | -0.3661385 |
| C | 1.9274041 | 1.9587884 | -0.2838704 |
| N | 2.1222414 | 3.2756406 | -0.2713000 |
| C | 1.8187875 | -2.6326625 | -0.2489732 |
| N | 0.4979623 | -2.2921265 | -0.2419332 |
| N | 2.1611596 | -0.3431281 | -0.2746546 |
| N | -1.8710351 | -0.2481990 | -0.2575538 |
| N | 0.5912669 | 1.6811041 | -0.2525512 |
| N | -0.0426222 | 2.8562932 | -0.2181688 |
| N | -0.1921830 | -3.4368400 | -0.2378784 |
| N | 1.9505466 | -3.9573071 | -0.2457825 |
| N | 0.6830317 | -4.4332691 | -0.2373762 |
| N | 0.8796401 | 3.8104762 | -0.2288253 |
| C | -6.1482051 | -0.1973870 | -0.7439700 |
| H | -6.6658063 | -0.9370559 | -0.1183860 |

| | | | |
|---|---|---|---|
| H | -6.5897529 | 0.7913914 | -0.5684582 |
| H | -6.3244352 | -0.4762479 | -1.7958461 |
| H | 5.9759275 | -0.4380970 | -0.4117173 |
| C | 1.1348011 | -4.7037961 | -3.4326350 |
| C | 1.0749452 | -3.3126062 | -3.4642651 |
| C | -1.3040803 | -3.3693953 | -3.4314526 |
| C | -1.2949530 | -4.7623053 | -3.4007872 |
| C | -0.0634005 | -5.4201922 | -3.3971405 |
| H | 2.1043133 | -5.1962276 | -3.4114245 |
| H | -2.2387774 | -5.3026283 | -3.3708528 |
| Pt | -0.1773737 | -0.7015245 | -3.4634129 |
| C | 0.9252419 | 2.0563558 | -3.4862275 |
| C | -1.4014238 | 2.0068696 | -3.4124820 |
| C | 0.9275230 | 3.4360625 | -3.3861018 |
| H | 1.8545473 | 1.4892214 | -3.5530904 |
| C | -1.4515689 | 3.3879005 | -3.3046400 |
| H | -2.3091485 | 1.4020598 | -3.4360508 |
| C | -0.2752397 | 4.1435436 | -3.2621914 |
| H | 1.8846396 | 3.9560730 | -3.3640321 |
| H | -2.4261379 | 3.8709798 | -3.2312858 |
| C | 2.1589121 | -2.3496288 | -3.4878226 |
| N | 3.4836090 | -2.4811114 | -3.4965934 |
| C | -2.4327527 | -2.4588475 | -3.4492146 |
| N | -2.1547491 | -1.1227383 | -3.4803005 |
| N | -0.1306740 | -2.6922023 | -3.4587387 |
| N | -0.2260870 | 1.3399675 | -3.4819922 |
| N | 1.8181447 | -1.0287685 | -3.4893407 |
| N | 2.9627712 | -0.3384286 | -3.4954218 |
| N | -3.3295799 | -0.4885215 | -3.5167928 |
| N | -3.7497330 | -2.6531676 | -3.4636505 |
| N | -4.2840873 | -1.4103858 | -3.5070745 |
| N | 3.9592718 | -1.2135476 | -3.5025660 |
| C | -0.2845807 | 5.6201141 | -3.0234071 |
| H | -0.0296420 | 5.8025448 | -1.9665009 |
| H | 0.4699510 | 6.1327131 | -3.6350949 |
| H | -1.2687140 | 6.0613789 | -3.2240250 |
| H | -0.0354400 | -6.5082474 | -3.3533528 |

**3B dimer 180° rotated**

| | | | |
|---|---|---|---|
| C | -1.2170028 | 3.8909645 | -0.0084693 |
| C | -1.1924113 | 2.5034837 | -0.1551747 |
| C | 1.1924715 | 2.5034685 | -0.1550101 |
| C | 1.2170513 | 3.8909713 | -0.0082722 |
| C | 0.0000171 | 4.5726608 | 0.0736949 |
| H | -2.1748995 | 4.4028004 | 0.0581226 |
| H | 2.1749244 | 4.4027758 | 0.0584788 |
| Pt | 0.0000322 | -0.1311223 | -0.2929402 |
| C | -1.1662910 | -2.8596780 | -0.2568914 |
| C | 1.1663247 | -2.8596969 | -0.2568356 |
| C | -1.1918519 | -4.2449470 | -0.2785641 |
| H | -2.0864713 | -2.2709631 | -0.2591635 |
| C | 1.1918859 | -4.2449585 | -0.2785411 |
| H | 2.0865144 | -2.2709872 | -0.2590912 |
| C | 0.0000066 | -4.9791964 | -0.2924432 |
| H | -2.1600019 | -4.7459973 | -0.2960199 |
| H | 2.1600236 | -4.7460277 | -0.2959385 |
| C | -2.2949080 | 1.5695349 | -0.2205082 |
| N | -3.6170443 | 1.7306819 | -0.2110784 |
| C | 2.2950044 | 1.5695228 | -0.2201998 |
| N | 1.9846782 | 0.2409880 | -0.2669766 |
| N | 0.0000093 | 1.8592648 | -0.2183718 |
| N | 0.0000227 | -2.1711302 | -0.2539970 |
| N | -1.9846377 | 0.2410005 | -0.2672036 |
| N | -3.1425063 | -0.4206296 | -0.2823841 |
| N | 3.1425608 | -0.4206512 | -0.2820632 |
| N | 3.6170744 | 1.7306471 | -0.2106092 |
| N | 4.1210256 | 0.4767504 | -0.2493101 |
| N | -4.1209725 | 0.4767694 | -0.2497817 |
| C | 0.0000034 | -6.4781970 | -0.3029638 |
| H | -0.0000360 | -6.8650921 | 0.7286875 |
| H | -0.8937675 | -6.8755729 | -0.8007242 |

| | | | |
|---|---|---|---|
| H | 0.8937854 | -6.8755847 | -0.8006935 |
| H | 0.0000097 | 5.6536708 | 0.2159094 |
| C | 1.2167578 | -4.8960163 | -3.7158061 |
| C | 1.1924294 | -3.5070150 | -3.5844423 |
| C | -1.1923102 | -3.5070242 | -3.5844193 |
| C | -1.2166047 | -4.8960252 | -3.7157905 |
| C | 0.0000636 | -5.5792470 | -3.7854569 |
| H | 2.1747775 | -5.4084672 | -3.7758429 |
| H | -2.1746401 | -5.4084924 | -3.7757858 |
| Pt | 0.0000581 | -0.8717310 | -3.4498031 |
| C | 1.1661603 | 1.8582859 | -3.4894395 |
| C | -1.1660655 | 1.8582606 | -3.4895679 |
| C | 1.1919165 | 3.2438749 | -3.4882803 |
| H | 2.0865138 | 1.2699640 | -3.4859376 |
| C | -1.1918524 | 3.2438616 | -3.4884305 |
| H | -2.0864193 | 1.2699296 | -3.4861728 |
| C | 0.0000365 | 3.9788033 | -3.4874458 |
| H | 2.1605760 | 3.7442699 | -3.4890656 |
| H | -2.1605056 | 3.7442393 | -3.4893081 |
| C | 2.2948971 | -2.5726789 | -3.5244299 |
| N | 3.6169481 | -2.7337237 | -3.5378312 |
| C | -2.2947525 | -2.5726996 | -3.5244289 |
| N | -1.9845717 | -1.2440573 | -3.4780690 |
| N | 0.0000631 | -2.8623581 | -3.5248634 |
| N | 0.0000436 | 1.1692945 | -3.4850094 |
| N | 1.9846810 | -1.2440432 | -3.4780256 |
| N | 3.1425944 | -0.5823401 | -3.4673407 |
| N | -3.1424871 | -0.5823621 | -3.4674325 |
| N | -3.6168336 | -2.7337556 | -3.5378483 |
| N | -4.1208656 | -1.4797641 | -3.5023451 |
| N | 4.1209785 | -1.4797394 | -3.5022704 |
| C | 0.0000267 | 5.4770103 | -3.4608565 |
| H | 0.0002459 | 5.8338512 | -2.4185196 |
| H | 0.8930266 | 5.8888506 | -3.9478538 |
| H | -0.8931731 | 5.8888497 | -3.9474830 |
| H | 0.0000739 | -6.6630585 | -3.9019569 |

**4A dimer parallel**

| | | | |
|---|---|---|---|
| C | 1.2274170 | -3.9778432 | 0.4738002 |
| C | 1.1982141 | -2.5874132 | 0.3970173 |
| C | -1.1863691 | -2.5823941 | 0.4128181 |
| C | -1.2206072 | -3.9726434 | 0.4894417 |
| C | 0.0022835 | -4.6513721 | 0.5169110 |
| H | 2.1779151 | -4.5058122 | 0.4936500 |
| H | -2.1729150 | -4.4967006 | 0.5209617 |
| Pt | 0.0105942 | 0.0447548 | 0.2916667 |
| C | 1.1753349 | 2.7700734 | 0.0104571 |
| C | -1.1459037 | 2.7750542 | 0.0179648 |
| C | 1.2164735 | 4.1325170 | -0.2042658 |
| H | 2.0975423 | 2.1890945 | 0.0859313 |
| C | -1.1826097 | 4.1375499 | -0.1970699 |
| H | -2.0700648 | 2.1981555 | 0.1007678 |
| C | 0.0180866 | 4.8784255 | -0.3161936 |
| H | 2.1931148 | 4.5987073 | -0.3056386 |
| H | -2.1576888 | 4.6084673 | -0.2917874 |
| C | 2.3038730 | -1.6554863 | 0.3344733 |
| N | 3.6245506 | -1.8190306 | 0.2924813 |
| C | -2.2889300 | -1.6457290 | 0.3670008 |
| N | -1.9727661 | -0.3189975 | 0.3127048 |
| N | 0.0070763 | -1.9396700 | 0.3764007 |
| N | 0.0135570 | 2.0769279 | 0.1150314 |
| N | 1.9923933 | -0.3274260 | 0.2836181 |
| N | 3.1499265 | 0.3320971 | 0.2136212 |
| N | -3.1285922 | 0.3455583 | 0.2631823 |
| N | -3.6107972 | -1.8036925 | 0.3471040 |
| N | 0.0203596 | 6.2291197 | -0.5304248 |
| N | -4.1102619 | -0.5503441 | 0.2823710 |
| N | 4.1280416 | -0.5679418 | 0.2176624 |
| Cl | -0.0009224 | -6.3794027 | 0.6059081 |
| C | 1.2199600 | -5.0286643 | 3.9274230 |
| C | 1.1847192 | -3.6431933 | 3.7856925 |

| | | | |
|---|---|---|---|
| C | -1.1964688 | -3.6468974 | 3.8012400 |
| C | -1.2253968 | -5.0325539 | 3.9430387 |
| C | -0.0012188 | -5.7055574 | 4.0065473 |
| H | 2.1723911 | -5.5502144 | 3.9817468 |
| H | -2.1753947 | -5.5570790 | 4.0097365 |
| Pt | -0.0114102 | -1.0147381 | 3.5820784 |
| C | 1.1455290 | 1.7182692 | 3.4254675 |
| C | -1.1802909 | 1.7130771 | 3.4230450 |
| C | 1.1811712 | 3.0887269 | 3.2852223 |
| H | 2.0695623 | 1.1389991 | 3.4797099 |
| C | -1.2213621 | 3.0834000 | 3.2823891 |
| H | -2.1020568 | 1.1301960 | 3.4769778 |
| C | -0.0216623 | 3.8299090 | 3.1877125 |
| H | 2.1555673 | 3.5688980 | 3.2460775 |
| H | -2.1978201 | 3.5591237 | 3.2397027 |
| C | 2.2875306 | -2.7103938 | 3.7103265 |
| N | 3.6087893 | -2.8689781 | 3.7499996 |
| C | -2.3030337 | -2.7175339 | 3.7414771 |
| N | -1.9952908 | -1.3910820 | 3.6345813 |
| N | -0.0072920 | -2.9980623 | 3.7241661 |
| N | -0.0159260 | 1.0195743 | 3.4863869 |
| N | 1.9743521 | -1.3849571 | 3.6071902 |
| N | 3.1331711 | -0.7227806 | 3.5834225 |
| N | -3.1561472 | -0.7322414 | 3.6285439 |
| N | -3.6230978 | -2.8798747 | 3.8009291 |
| N | -0.0247334 | 5.1803834 | 3.0052473 |
| N | -4.1307325 | -1.6304190 | 3.7287946 |
| N | 4.1117302 | -1.6180451 | 3.6696937 |
| Cl | 0.0027015 | -7.4257508 | 4.1933581 |
| C | 1.2303309 | 5.9137753 | 2.9707127 |
| H | 1.8969315 | 5.5089787 | 2.1936201 |
| H | 1.0241241 | 6.9622890 | 2.7336747 |
| H | 1.7614478 | 5.8736775 | 3.9362903 |
| C | -1.2832832 | 5.9082754 | 2.9794762 |
| H | -1.9471218 | 5.5138009 | 2.1944401 |
| H | -1.8160520 | 5.8489185 | 3.9430481 |
| H | -1.0814599 | 6.9618585 | 2.7620570 |
| C | 1.2715208 | 6.9052762 | -0.8508672 |
| H | 1.0839348 | 7.9809455 | -0.9294993 |
| H | 2.0165053 | 6.7505531 | -0.0581103 |
| H | 1.7035168 | 6.5557313 | -1.8042700 |
| C | -1.2299904 | 6.9109527 | -0.8421007 |
| H | -1.6739714 | 6.5580282 | -1.7887308 |
| H | -1.9674905 | 6.7656595 | -0.0405474 |
| H | -1.0364850 | 7.9849010 | -0.9299897 |

**4A dimer 90° rotated**

| | | | |
|---|---|---|---|
| C | 1.1192304 | -4.6736462 | 0.3451587 |
| C | 1.0601608 | -3.2856653 | 0.2874380 |
| C | -1.3159062 | -3.3294424 | 0.2495431 |
| C | -1.3226779 | -4.7191116 | 0.3072919 |
| C | -0.0894436 | -5.3760404 | 0.3602998 |
| H | 2.0789420 | -5.1800735 | 0.4063910 |
| H | -2.2629202 | -5.2646073 | 0.3182856 |
| Pt | -0.1755363 | -0.6679635 | 0.2301369 |
| C | 0.9491109 | 2.0814893 | 0.2559697 |
| C | -1.3717242 | 2.0538395 | 0.3410165 |
| C | 0.9812540 | 3.4437881 | 0.4358407 |
| H | 1.8751910 | 1.5150337 | 0.1556117 |
| C | -1.4180677 | 3.4159570 | 0.5359467 |
| H | -2.2886893 | 1.4669266 | 0.2831737 |
| C | -0.2206303 | 4.1702949 | 0.6323298 |
| H | 1.9520271 | 3.9282547 | 0.4837276 |
| H | -2.3934994 | 3.8820278 | 0.6457389 |
| C | 2.1508389 | -2.3309708 | 0.2845711 |
| N | 3.4744663 | -2.4713967 | 0.3104776 |
| C | -2.4391739 | -2.4153274 | 0.1743593 |
| N | -2.1542029 | -1.0800626 | 0.1431798 |
| N | -0.1401814 | -2.6558637 | 0.2470762 |
| N | -0.2067948 | 1.3666167 | 0.2298997 |
| N | 1.8175516 | -1.0077193 | 0.2670362 |

| | | | |
|---|---|---|---|
| N | 2.9668906 | -0.3254412 | 0.2860034 |
| N | -3.3232051 | -0.4429999 | 0.0393071 |
| N | -3.7542430 | -2.6059228 | 0.0936043 |
| N | -0.2194324 | 5.4984511 | 0.9100922 |
| N | -4.2802977 | -1.3621816 | 0.0088721 |
| N | 3.9580149 | -1.2075271 | 0.3090457 |
| Cl | -0.0582810 | -7.0995798 | 0.4583721 |
| C | 4.2039795 | 0.8693903 | 3.4479130 |
| C | 2.8134687 | 0.8629096 | 3.4783300 |
| C | 2.7709188 | -1.5134305 | 3.4855547 |
| C | 4.1597558 | -1.5727978 | 3.4563576 |
| C | 4.8620627 | -0.3642512 | 3.4317554 |
| H | 4.7493309 | 1.8096182 | 3.4294871 |
| H | 4.6675279 | -2.5331887 | 3.4237124 |
| Pt | 0.1523690 | -0.2784589 | 3.4672202 |
| C | -2.5668363 | 0.9142575 | 3.2811061 |
| C | -2.5963675 | -1.4045874 | 3.4097097 |
| C | -3.9251873 | 0.9566810 | 3.0605998 |
| H | -1.9808790 | 1.8322496 | 3.3320995 |
| C | -3.9552243 | -1.4403008 | 3.2057455 |
| H | -2.0320302 | -2.3284632 | 3.5381311 |
| C | -4.6778581 | -0.2424664 | 2.9733110 |
| H | -4.3888013 | 1.9297852 | 2.9234034 |
| H | -4.4389971 | -2.4118352 | 3.1676936 |
| C | 1.8978664 | 1.9869268 | 3.5139109 |
| N | 2.0865691 | 3.3034240 | 3.5731568 |
| C | 1.8165892 | -2.6044306 | 3.4908964 |
| N | 0.4931956 | -2.2716366 | 3.4757617 |
| N | 2.1402236 | -0.3129131 | 3.4902462 |
| N | -1.8819114 | -0.2482309 | 3.4270668 |
| N | 0.5623290 | 1.7017993 | 3.5244175 |
| N | -0.0769152 | 2.8721359 | 3.5933110 |
| N | -0.1883736 | -3.4214374 | 3.4659896 |
| N | 1.9577485 | -3.9282431 | 3.4939643 |
| N | -6.0007714 | -0.2492142 | 2.6715072 |
| N | 0.6941665 | -4.4123765 | 3.4799514 |
| N | 0.8412350 | 3.8302384 | 3.6234176 |
| Cl | 6.5871909 | -0.3965482 | 3.3683507 |
| C | -6.6866436 | -1.5169982 | 2.4359926 |
| H | -6.6160094 | -2.1674899 | 3.3195269 |
| H | -7.7464729 | -1.3143098 | 2.2519912 |
| H | -6.2662605 | -2.0386805 | 1.5622743 |
| C | -6.6537312 | 0.9859940 | 2.2608553 |
| H | -6.5644351 | 1.7575377 | 3.0399574 |
| H | -6.2279109 | 1.3762542 | 1.3217428 |
| H | -7.7188008 | 0.7891981 | 2.1042203 |
| C | 1.0437880 | 6.1893047 | 1.1547947 |
| H | 1.7108067 | 6.0995458 | 0.2854754 |
| H | 0.8380702 | 7.2529276 | 1.3116792 |
| H | 1.5488389 | 5.7879913 | 2.0470687 |
| C | -1.4616090 | 6.1576250 | 1.2883839 |
| H | -2.2193619 | 6.0556944 | 0.4974657 |
| H | -1.8681361 | 5.7467794 | 2.2272259 |
| H | -1.2675158 | 7.2251426 | 1.4314146 |

**4A dimer 180° rotated**

| | | | |
|---|---|---|---|
| C | 1.2366582 | -4.3746115 | 0.5314111 |
| C | 1.2025783 | -2.9808489 | 0.5343057 |
| C | -1.1776833 | -2.9839998 | 0.5136841 |
| C | -1.2081665 | -4.3777636 | 0.5092865 |
| C | 0.0151633 | -5.0527918 | 0.5156411 |
| H | 2.1897927 | -4.8975217 | 0.5158398 |
| H | -2.1594950 | -4.9030992 | 0.4762807 |
| Pt | 0.0099394 | -0.3454215 | 0.4044831 |
| C | 1.1719942 | 2.3776189 | 0.1521651 |
| C | -1.1540609 | 2.3760158 | 0.1404500 |
| C | 1.2102881 | 3.7537470 | 0.1065012 |
| H | 2.0955683 | 1.7961238 | 0.1788719 |
| C | -1.1938169 | 3.7521615 | 0.0947658 |
| H | -2.0769212 | 1.7930100 | 0.1569353 |
| C | 0.0077574 | 4.5045514 | 0.1034104 |

| | | | |
|---|---|---|---|
| H | 2.1853134 | 4.2330757 | 0.0845353 |
| H | -2.1693535 | 4.2299524 | 0.0632471 |
| C | 2.3046509 | -2.0468986 | 0.5148733 |
| N | 3.6263781 | -2.2099952 | 0.5327155 |
| C | -2.2817939 | -2.0528977 | 0.4768030 |
| N | -1.9742136 | -0.7241775 | 0.3992462 |
| N | 0.0116026 | -2.3321573 | 0.5212060 |
| N | 0.0092398 | 1.6786449 | 0.1895667 |
| N | 1.9948730 | -0.7190768 | 0.4307793 |
| N | 3.1539619 | -0.0613355 | 0.3923217 |
| N | -3.1343393 | -0.0693192 | 0.3442917 |
| N | -3.6031639 | -2.2194730 | 0.4746026 |
| N | 0.0071197 | 5.8645766 | 0.1222324 |
| N | -4.1106387 | -0.9699673 | 0.3907014 |
| N | 4.1317983 | -0.9594055 | 0.4546767 |
| Cl | 0.0177394 | -6.7886556 | 0.4811118 |
| C | -1.2247569 | 4.7922608 | 3.3762695 |
| C | -1.1962740 | 3.3984716 | 3.3683346 |
| C | 1.1840238 | 3.3918135 | 3.3830621 |
| C | 1.2201019 | 4.7854968 | 3.3903838 |
| C | -0.0004918 | 5.4654400 | 3.3901053 |
| H | -2.1757658 | 5.3188652 | 3.3971024 |
| H | 2.1736651 | 5.3069333 | 3.4219377 |
| Pt | -0.0142601 | 0.7580490 | 3.4908836 |
| C | -1.1864805 | -1.9610911 | 3.7390489 |
| C | 1.1395055 | -1.9668123 | 3.7612205 |
| C | -1.2293473 | -3.3370020 | 3.7877972 |
| H | -2.1080322 | -1.3767053 | 3.7069128 |
| C | 1.1747203 | -3.3428855 | 3.8109480 |
| H | 2.0643120 | -1.3868612 | 3.7459661 |
| C | -0.0291618 | -4.0914928 | 3.7998917 |
| H | -2.2061195 | -3.8129835 | 3.8054600 |
| H | 2.1485521 | -3.8238057 | 3.8468615 |
| C | -2.3021676 | 2.4689697 | 3.3865278 |
| N | -3.6231427 | 2.6374566 | 3.3676551 |
| C | 2.2843539 | 2.4562155 | 3.4170142 |
| N | 1.9714179 | 1.1287383 | 3.4942751 |
| N | -0.0079520 | 2.7448893 | 3.3767765 |
| N | -0.0213663 | -1.2658980 | 3.7058284 |
| N | -1.9977263 | 1.1396965 | 3.4667196 |
| N | -3.1595350 | 0.4864074 | 3.5017716 |
| N | 3.1288643 | 0.4691641 | 3.5483918 |
| N | 3.6064225 | 2.6173723 | 3.4186942 |
| N | -0.0320820 | -5.4515760 | 3.7872574 |
| N | 4.1088210 | 1.3658682 | 3.5018245 |
| N | -4.1336869 | 1.3885350 | 3.4411476 |
| Cl | 0.0040976 | 7.2011687 | 3.4307661 |
| C | 1.2227400 | -6.1857975 | 3.8811140 |
| H | 1.7211515 | -6.0259307 | 4.8517503 |
| H | 1.0190402 | -7.2543540 | 3.7656371 |
| H | 1.9147097 | -5.8854520 | 3.0808910 |
| C | -1.2920055 | -6.1803790 | 3.8495966 |
| H | -1.8088266 | -6.0274048 | 4.8116893 |
| H | -1.9662729 | -5.8684004 | 3.0388350 |
| H | -1.0913336 | -7.2488247 | 3.7272715 |
| C | -1.2501342 | 6.5961274 | 0.0426160 |
| H | -1.0494324 | 7.6633776 | 0.1747894 |
| H | -1.7509143 | 6.4499573 | -0.9290023 |
| H | -1.9385708 | 6.2806929 | 0.8400195 |
| C | 1.2644264 | 6.5971978 | 0.0519503 |
| H | 1.9464226 | 6.2842904 | 0.8558575 |
| H | 1.7735103 | 6.4493591 | -0.9150519 |
| H | 1.0616268 | 7.6645448 | 0.1799760 |

**4B dimer parallel**

| | | | |
|---|---|---|---|
| C | 1.2235297 | -4.5171408 | -0.0866408 |
| C | 1.1918277 | -3.1265477 | -0.1553673 |
| C | -1.1918313 | -3.1265635 | -0.1554097 |
| C | -1.2235138 | -4.5171306 | -0.0866844 |
| C | -0.0000121 | -5.1944782 | -0.0547050 |
| H | 2.1747149 | -5.0426670 | -0.0501489 |

| | | | |
|---|---|---|---|
| H | -2.1747312 | -5.0426765 | -0.0502282 |
| Pt | -0.0000025 | -0.4920354 | -0.2616010 |
| C | 1.1653633 | 2.2381542 | -0.1702614 |
| C | -1.1653675 | 2.2381625 | -0.1701552 |
| C | 1.1909791 | 3.6210916 | -0.0793672 |
| H | 2.0854854 | 1.6496208 | -0.2092266 |
| C | -1.1909784 | 3.6210788 | -0.0792567 |
| H | -2.0855007 | 1.6496124 | -0.2090564 |
| C | -0.0000057 | 4.3544804 | -0.0234821 |
| H | 2.1590371 | 4.1219385 | -0.0501993 |
| H | -2.1590432 | 4.1219352 | -0.0500011 |
| C | 2.2959450 | -2.1916364 | -0.1605295 |
| N | 3.6153634 | -2.3505550 | -0.0819760 |
| C | -2.2959757 | -2.1916337 | -0.1606064 |
| N | -1.9844373 | -0.8632213 | -0.2115006 |
| N | -0.0000026 | -2.4803141 | -0.2024345 |
| N | -0.0000093 | 1.5482925 | -0.2094690 |
| N | 1.9844308 | -0.8632101 | -0.2114356 |
| N | 3.1401297 | -0.1996532 | -0.1654512 |
| N | -3.1401433 | -0.1996551 | -0.1655399 |
| N | -3.6153605 | -2.3505602 | -0.0820867 |
| N | -4.1179355 | -1.0970463 | -0.0872930 |
| N | 4.1179237 | -1.0970362 | -0.0871771 |
| C | 0.0000053 | 5.8480411 | 0.1198360 |
| H | 0.0000528 | 6.1270788 | 1.1853808 |
| H | 0.8945695 | 6.2968128 | -0.3311267 |
| H | -0.8946006 | 6.2968108 | -0.3310488 |
| C | 1.2235653 | -4.5355734 | -3.6323446 |
| C | 1.1919087 | -3.1444269 | -3.5758196 |
| C | -1.1917183 | -3.1444223 | -3.5758608 |
| C | -1.2234123 | -4.5355853 | -3.6323884 |
| C | 0.0001056 | -5.2131397 | -3.6586258 |
| H | 2.1748090 | -5.0613814 | -3.6643916 |
| H | -2.1746174 | -5.0613821 | -3.6644684 |
| Pt | 0.0000804 | -0.5089330 | -3.4963207 |
| C | 1.1655034 | 2.2199095 | -3.6152056 |
| C | -1.1653630 | 2.2198970 | -3.6151044 |
| C | 1.1909249 | 3.6020660 | -3.7158274 |
| H | 2.0855432 | 1.6312751 | -3.5778422 |
| C | -1.1907942 | 3.6020703 | -3.7157225 |
| H | -2.0853900 | 1.6312708 | -3.5776810 |
| C | 0.0000641 | 4.3356905 | -3.7696280 |
| H | 2.1589725 | 4.1021079 | -3.7582333 |
| H | -2.1588400 | 4.1021009 | -3.7580449 |
| C | 2.2960379 | -2.2094919 | -3.5789535 |
| N | 3.6155518 | -2.3692889 | -3.6541601 |
| C | -2.2958800 | -2.2095009 | -3.5790290 |
| N | -1.9843564 | -0.8806677 | -3.5417718 |
| N | 0.0000775 | -2.4977490 | -3.5347535 |
| N | 0.0000772 | 1.5306184 | -3.5674275 |
| N | 1.9845226 | -0.8806571 | -3.5417091 |
| N | 3.1403145 | -0.2176450 | -3.5927873 |
| N | -3.1401516 | -0.2176543 | -3.5928761 |
| N | -3.6153725 | -2.3693020 | -3.6542677 |
| N | -4.1180150 | -1.1158126 | -3.6609126 |
| N | 4.1181805 | -1.1157925 | -3.6607968 |
| C | 0.0000576 | 5.8334425 | -3.8561209 |
| H | 0.0003138 | 6.2739081 | -2.8466252 |
| H | 0.8921670 | 6.2067918 | -4.3750412 |
| H | -0.8922834 | 6.2068173 | -4.3746125 |
| Cl | -0.0000020 | -6.9208773 | 0.0384005 |
| Cl | 0.0000951 | -6.9402740 | -3.7374635 |

**4B dimer 90° rotated**

| | | | |
|---|---|---|---|
| C | 4.2255613 | 0.8086961 | -0.3504361 |
| C | 2.8356356 | 0.8138887 | -0.3053707 |
| C | 2.7731925 | -1.5621217 | -0.2744108 |
| C | 4.1608296 | -1.6332452 | -0.3188915 |
| C | 4.8733690 | -0.4305172 | -0.3627335 |
| H | 4.7777844 | 1.7443605 | -0.3865681 |
| H | 4.6604919 | -2.5979698 | -0.3489883 |

| | | | |
|---|---|---|---|
| Pt | 0.1642100 | -0.3046905 | -0.2688797 |
| C | -2.5400215 | 0.9239683 | -0.3467400 |
| C | -2.5947093 | -1.4017348 | -0.2454040 |
| C | -3.9203857 | 0.9754708 | -0.4625905 |
| H | -1.9336754 | 1.8307331 | -0.3282975 |
| C | -3.9737542 | -1.4024886 | -0.3524646 |
| H | -2.0300878 | -2.3313373 | -0.1664308 |
| C | -4.6781074 | -0.1997901 | -0.4945542 |
| H | -4.4008823 | 1.9501908 | -0.5493264 |
| H | -4.4957048 | -2.3587361 | -0.3644829 |
| C | 1.9300269 | 1.9460218 | -0.2839190 |
| N | 2.1289526 | 3.2620022 | -0.2719272 |
| C | 1.8092801 | -2.6449876 | -0.2528680 |
| N | 0.4888306 | -2.3008888 | -0.2468674 |
| N | 2.1536016 | -0.3562038 | -0.2726855 |
| N | -1.8759333 | -0.2518027 | -0.2600241 |
| N | 0.5926770 | 1.6716463 | -0.2526181 |
| N | -0.0370573 | 2.8484938 | -0.2190747 |
| N | -0.2030844 | -3.4438503 | -0.2564401 |
| N | 1.9383231 | -3.9696590 | -0.2623919 |
| N | 0.6703032 | -4.4427143 | -0.2628450 |
| N | 0.8884512 | 3.8002795 | -0.2300320 |
| Cl | 6.5953539 | -0.4781023 | -0.4481968 |
| C | -6.1533710 | -0.1910332 | -0.7408139 |
| H | -6.6704387 | -0.9342524 | -0.1192123 |
| H | -6.5936275 | 0.7971614 | -0.5591625 |
| H | -6.3318498 | -0.4634591 | -1.7939032 |
| C | 1.1583196 | -4.6894921 | -3.4252166 |
| C | 1.0868449 | -3.3015879 | -3.4635951 |
| C | -1.2891658 | -3.3645762 | -3.4304983 |
| C | -1.2837525 | -4.7546610 | -3.3918914 |
| C | -0.0442465 | -5.4022794 | -3.3841770 |
| H | 2.1233657 | -5.1889153 | -3.3990374 |
| H | -2.2192873 | -5.3073645 | -3.3579421 |
| Pt | -0.1712027 | -0.6925912 | -3.4668452 |
| C | 0.9249664 | 2.0666107 | -3.4903690 |
| C | -1.4015994 | 2.0122461 | -3.4128533 |
| C | 0.9246738 | 3.4462614 | -3.3903954 |
| H | 1.8553462 | 1.5016109 | -3.5587998 |
| C | -1.4542665 | 3.3932111 | -3.3050928 |
| H | -2.3080984 | 1.4056267 | -3.4359830 |
| C | -0.2794406 | 4.1513880 | -3.2647038 |
| H | 1.8809200 | 3.9680396 | -3.3721331 |
| H | -2.4296641 | 3.8744863 | -3.2312830 |
| C | 2.1695341 | -2.3374463 | -3.4836856 |
| N | 3.4942322 | -2.4665034 | -3.4778362 |
| C | -2.4213208 | -2.4588255 | -3.4488822 |
| N | -2.1470476 | -1.1215240 | -3.4832611 |
| N | -0.1190785 | -2.6821958 | -3.4619626 |
| N | -0.2249413 | 1.3477579 | -3.4839313 |
| N | 1.8252918 | -1.0169609 | -3.4869229 |
| N | 2.9684325 | -0.3250427 | -3.4793384 |
| N | -3.3238394 | -0.4917916 | -3.5162708 |
| N | -3.7373511 | -2.6575893 | -3.4580863 |
| N | -4.2756325 | -1.4172073 | -3.5015673 |
| N | 3.9672786 | -1.1985717 | -3.4768101 |
| Cl | 0.0035963 | -7.1250137 | -3.3089386 |
| C | -0.2917608 | 5.6282595 | -3.0282534 |
| H | -0.0310667 | 5.8143487 | -1.9734904 |
| H | 0.4575548 | 6.1418524 | -3.6454283 |
| H | -1.2783586 | 6.0660857 | -3.2239496 |

**4B dimer 180° rotated**

| | | | |
|---|---|---|---|
| C | -1.2235542 | 3.5272292 | -0.1303334 |
| C | -1.1916763 | 2.1364494 | -0.2059401 |
| C | 1.1916635 | 2.1364560 | -0.2058905 |
| C | 1.2235354 | 3.5272236 | -0.1302857 |
| C | -0.0000062 | 4.2012353 | -0.0941925 |
| H | -2.1745107 | 4.0530328 | -0.0964207 |
| H | 2.1744986 | 4.0530370 | -0.0963310 |
| Pt | -0.0000042 | -0.4980819 | -0.2997038 |

| | | | |
|---|---|---|---|
| C | -1.1658014 | -3.2285533 | -0.1836335 |
| C | 1.1657752 | -3.2285710 | -0.1834882 |
| C | -1.1911287 | -4.6102862 | -0.0781747 |
| H | -2.0864198 | -2.6419100 | -0.2336312 |
| C | 1.1910820 | -4.6102643 | -0.0780296 |
| H | 2.0864223 | -2.6418976 | -0.2333866 |
| C | -0.0000078 | -5.3436302 | -0.0117305 |
| H | -2.1591999 | -5.1108145 | -0.0494605 |
| H | 2.1591847 | -5.1108184 | -0.0491908 |
| C | -2.2953288 | 1.2035555 | -0.2427338 |
| N | -3.6173287 | 1.3649617 | -0.2240373 |
| C | 2.2953239 | 1.2035600 | -0.2426278 |
| N | 1.9847306 | -0.1253955 | -0.2711881 |
| N | -0.0000023 | 1.4904941 | -0.2432220 |
| N | -0.0000045 | -2.5390666 | -0.2310100 |
| N | -1.9847373 | -0.1253914 | -0.2712857 |
| N | -3.1422067 | -0.7868714 | -0.2710923 |
| N | 3.1422013 | -0.7868712 | -0.2709256 |
| N | 3.6173216 | 1.3649598 | -0.2238580 |
| N | 4.1212662 | 0.1127301 | -0.2418136 |
| N | -4.1212769 | 0.1127251 | -0.2420273 |
| C | -0.0000300 | -6.8342823 | 0.1490066 |
| H | -0.0001233 | -7.0967644 | 1.2189659 |
| H | -0.8921990 | -7.2874951 | -0.3003994 |
| H | 0.8922304 | -7.2874899 | -0.3002411 |
| C | 1.2233886 | -4.5288446 | -3.5869237 |
| C | 1.1916427 | -3.1370094 | -3.5410717 |
| C | -1.1914657 | -3.1370114 | -3.5411468 |
| C | -1.2232243 | -4.5288576 | -3.5870040 |
| C | 0.0001000 | -5.2042222 | -3.6029837 |
| H | 2.1744254 | -5.0552782 | -3.6059593 |
| H | -2.1742336 | -5.0552851 | -3.6060988 |
| Pt | 0.0000753 | -0.5018146 | -3.4696656 |
| C | 1.1658671 | 2.2285659 | -3.5797826 |
| C | -1.1657289 | 2.2285526 | -3.5797648 |
| C | 1.1912348 | 3.6101252 | -3.6869232 |
| H | 2.0864569 | 1.6413979 | -3.5366841 |
| C | -1.1911161 | 3.6101266 | -3.6869023 |
| H | -2.0863110 | 1.6413908 | -3.5366637 |
| C | 0.0000674 | 4.3435585 | -3.7530421 |
| H | 2.1594638 | 4.1097367 | -3.7272829 |
| H | -2.1593311 | 4.1097254 | -3.7272434 |
| C | 2.2953613 | -2.2038963 | -3.5139722 |
| N | 3.6172823 | -2.3657550 | -3.5304182 |
| C | -2.2952035 | -2.2038968 | -3.5141054 |
| N | -1.9848121 | -0.8747329 | -3.4964827 |
| N | 0.0000786 | -2.4904304 | -3.5155110 |
| N | 0.0000676 | 1.5391217 | -3.5319101 |
| N | 1.9849652 | -0.8747163 | -3.4963731 |
| N | 3.1426747 | -0.2134774 | -3.5016330 |
| N | -3.1425187 | -0.2135000 | -3.5017928 |
| N | -3.6171136 | -2.3657777 | -3.5306161 |
| N | -4.1213462 | -1.1134956 | -3.5232047 |
| N | 4.1215013 | -1.1134754 | -3.5229900 |
| C | 0.0000615 | 5.8384705 | -3.8703904 |
| H | 0.0003407 | 6.2980442 | -2.8699879 |
| H | 0.8926127 | 6.1986583 | -4.3976890 |
| H | -0.8927374 | 6.1987058 | -4.3972254 |
| Cl | -0.0000124 | 5.9325319 | 0.0015916 |
| Cl | 0.0000966 | -6.9364321 | -3.6294156 |
